# A domino reaction for generating β-aryl aldehydes from alkynes by substrate recognition catalysis

**DOI:** 10.1038/s41467-019-12770-w

**Published:** 2019-10-25

**Authors:** Weiwei Fang, Felix Bauer, Yaxi Dong, Bernhard Breit

**Affiliations:** Institut für Organische Chemie, Albert-Ludwigs-Universität Freiburg, Freiburg, Germany

**Keywords:** Catalytic mechanisms, Homogeneous catalysis, Synthetic chemistry methodology, Dynamic combinatorial chemistry

## Abstract

The development of universal catalyst systems that enable efficient, selective, and straightforward chemical transformations is of immense scientific importance. Here we develop a domino process comprising three consecutive reaction steps based on the strategy of supramolecular substrate recognition. This approach provides valuable β-aryl aldehydes from readily accessible α-alkynoic acids and arenes under mild reaction conditions, employing a supramolecular Rh catalyst containing an acylguanidine-bearing phosphine ligand. Furthermore, the synthesis of a key intermediate of Avitriptan using this protocol is accomplished. The first step of the reaction sequence is proved to be the regioselective hydroformylation of α-alkynoic acids. Remarkably, molecular recognition of the ligand and the substrate via hydrogen bonding plays a key role in this step. Control experiments indicate that the reaction further proceeds via 1,4-addition of an arene nucleophile to the unsaturated aldehyde intermediate and subsequent decarboxylation.

## Abstract (web summary)

Supramolecular interactions may be responsible for higher degree of efficiency and selectivity in homogeneous catalysis. Here, the authors exploit the concept of supramolecular catalysis to develop a domino rhodium-catalyzed hydroformylation of α-alkynoic acids followed by Michael addition of nucleophiles.

## Introduction

The pursuit of an ultimate catalytic system, which allows efficient, selective, and straightforward chemical transformations is an area of extreme interest in modern chemical science^1^. In this sense, supramolecular catalysis^2,3^ based on the substrate recognition via specific reversible interactions between substrate and catalyst-mimicking enzymes has emerged as a promising strategy^4–10^. In a pioneering work by Crabtree et al.^11^, molecular recognition enabled high regioselectivity in the oxygenation of saturated C−H bonds. Later, Reek and colleagues^12–19^, Bach and colleagues^20–27^, Zhang and colleagues^28–40^, and our group^41–47^ developed several methodologies resulting in excellent regio- and enantioselectivity, making use of substrate recognition catalysis. Nevertheless, notwithstanding the advances in this field, the scope and applicability of many methodologies^11,12,20,41^ is limited to very specific substrates leading to products with rather low molecular diversity. On the other hand, domino reactions can provide complex molecules in an elegant and efficient way^48–52^. Considering this, we envision that the combination of domino reactivity with supramolecular substrate recognition might overcome this limitation affording complex molecules from different easily available starting materials.

**Fig. 1:**
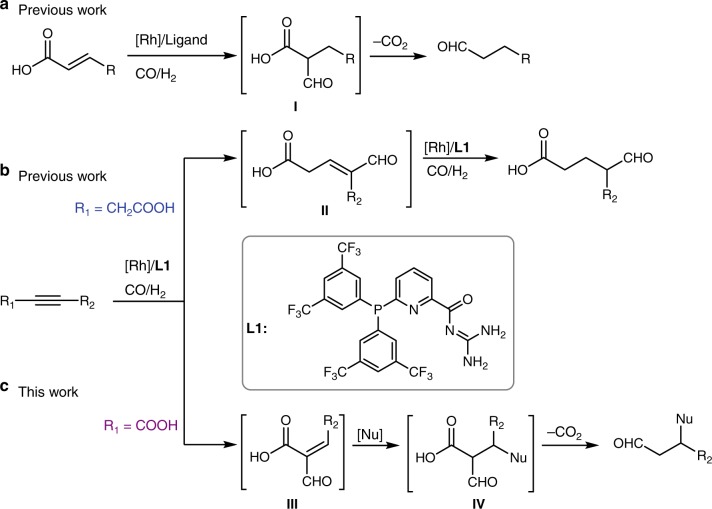
Domino reactions supported by supramolecular Rh catalyst. **a** Rh-catalyzed hydroformylation of α,β-unsaturated carboxylic acids followed by decarboxylation sequence. **b** Rh-catalyzed hydroformylation–hydrogenation of β-alkynoic acids. **c** Rh-catalyzed hydroformylation of α-alkynoic acids followed by Michael addition and decarboxylation

In line with our long-standing research interest in exploring supramolecular concepts in homogeneous catalysis, our group presented several examples of Rh-catalyzed domino reactions enabled by rational-designed supramolecular ligands^42,44–47,53^. In particular, a method for synthesizing terminal aliphatic aldehydes from α,β-unsaturated carboxylic acids involving a hydroformylation (HF)–decarboxylation sequence was developed (Fig. 1a)^42^. Due to the effect of carboxylic group (COOH), high regioselectivity was achieved. Recently, we successfully reported a Rh-catalyzed HF–hydrogenation process by using a rational-designed supramolecular ligand (**L1)**, which efficiently transformed unsymmetrical β-alkynoic acids into aliphatic aldehydes in high regio- and chemoselectivity (Fig. 1b)^47^. Mechanistic studies suggested that the key intermediate in this reaction is the unsaturated aldehyde **II**. In consideration of the success in HF-decarboxylation of α,β-unsaturated carboxylic acids; herein, we attempt to explore the HF of α-alkynoic acids using the supramolecular ligand **L1**. In this approach, the analog intermediate **III** is proposed to be generated based on the previous results (Fig. 1c)^42,47^. Then, due to the activation of the carboxyl group, the Michael addition of suitable arenes (such as indole derivatives) to this unsaturated aldehyde **III** can proceed faster than the competitive hydrogenation of the double bond due to its steric effect. In this way, valuable β-aryl aldehydes^17,54,55^ are generated after the final decarboxylation step^42^ of the proposed intermediate **IV** (Fig. 1c).

## Results

### Optimization of reaction conditions

To investigate the proposed domino reaction, 2-butynoic acid **S1** and 1,3,5-trimethoxybenzene (TMB) were selected as model substrates. The optimization of reaction conditions is shown in Table 1. Initially, the reaction was carried out employing **S1** and TMB (1:1, n/n) in 1,2-dichloroethane (DCE) at 55 °C in the presence of 1 mol% of [Rh(CO)_2_acac] and 5 mol% of **L1** under 6 bar of CO/H_2_ (1:1) (Table 1, entry 1). A nuclear magnetic resonance (NMR) study of the crude reaction mixture indicated that 3-(2,4,6-trimethoxyphenyl)butanal **1** was formed as the main product in 35% yield, similar to the conversion of TMB (36%, Table 1, entry 1). Despite the full conversion of the starting material **S1**, no other side products were identified. When camphorsulfonic acid (CSA) was used as the additive^47^, the yield of **1** was increased to 53% with a moderate conversion of 57% for TMB (Table 1, entry 2). A yield of 65% was obtained by varying the syngas pressure from 6 to 20 bar (see Supplementary Table 1). Other solvents and reaction temperatures were studied with DCE providing the most promising result at 35 °C (72% yield, Table 1, entry 3, and see Supplementary Tables 2 and 3). Slight effects on the yield were observed when changing the ratio of Rh and **L1** (Table 1, entry 3 and 4, and Supplementary Table 4). Increasing the amount of CSA from 6 to 9 mol% (Table 1, entry 5) and decreasing it from 6 to 3 mol% (Table 1, entry 6) led to inferior yields (37% and 64% yield, respectively). This indicates that the quantitative protonation of the acylguanidyl group into the acylguanidinium cation is essential for the performance of the catalyst^47^. Other acids were also tested (see Supplementary Table 5): TsOH and TfOH afforded lower yields, meanwhile TFA gave similar results to CSA (73% vs. 74% yield). Excellent yield was achieved by changing the ratio of **S1** to TMB from 1.0 to 1.5 (90% yield, Table 1, entry 7). Finally, the influence of the electronic properties of the ligand was investigated (Table 1, entries 7–11). We observed that when the σ-donor ability of the phosphine ligand was increased the catalytic activity drops to a moderate level for **L2** and **L3**. The even more electron rich ligands **L4** and **L5** showed no product formation. This confirms that the initially used ligand **L1** is superior to its analogs.

**Table 1:** Optimization of reaction conditions^a^


Entry	L	Ratio (Rh/L/CSA)	Ratio (S1/TMB)	Yield (%)	Conv. (S1/TMB) (%)
1^b^	**L1**	1:5:0	1:1	35	100/36
2^b^	**L1**	1:5:5	1:1	53	100/57
3	**L1**	1:5:5	1:1	72	100/74
4	**L1**	1:6:6	1:1	74	100/75
5	**L1**	1:6:9	1:1	37	100/70
6	**L1**	1:6:3	1:1	64	100/65
7	**L1**	1:6:6	1.5:1	90(90)	95/95
8	**L2**	1:6:6	1.5:1	67	99/71
9	**L3**	1:6:6	1.5:1	66	81/73
10	**L4**	1:6:6	1.5:1	0	10/13
11	**L5**	1:6:6	1.5:1	0	6/9

^a^[Rh(CO)_2_acac]/TMB = 1:100, 1 mmol of TMB, c(**S1**) = 0.5 M, CO/H_2_ (1:1, 20 bar), 35 °C. Yield and conversion (conv.) based on TMB were determined by NMR spectroscopy using dimethylacetamide (DMAc) as the internal standard and isolated yields were shown in parentheses. ^b^55 °C. CO/H_2_ (1:1, 6 bar). *acac* acetylacetonate, *DCE* 1,2-dichloroethane

### Scope of the proposed domino reaction

With the optimized reaction conditions in hand, the general scope of this catalytic protocol was examined. As shown in Table 2, when using TMB as the nucleophile, a variety of α-alkynoic acids could be successfully transformed to the corresponding β-aryl aldehydes in good yields. The length of the alkyl chain of the substrates (from Me to *n*-C_14_H_19_) slightly affected the yields (**1**–**11**, 73–90% yield). Bulkier secondary alkyl substituents such as *i*-Pr and Cy were well tolerated (**12** and **13**, 81% and 74% yield, respectively). Other functional groups such Bn and CN were also tested, affording the corresponding terminal aldehydes in good to excellent yields (**15** and **16**, 77% and 92% yield, respectively). Surprisingly, 3-(2,4,6-trimethoxyphenyl)-propanal **17** was obtained in 51% yield by using 3- (trimethylsilyl)-2-propynoic acid **S17**, whereas 2-propynoic acid cannot be transformed to **17** under the same conditions. This indicates that the TMS-protection of substrate **S17** is essential to achieve the desired transformation under the reaction conditions, whereby the TMS group was cleaved in situ and product **17** was formed.

**Table 2:**
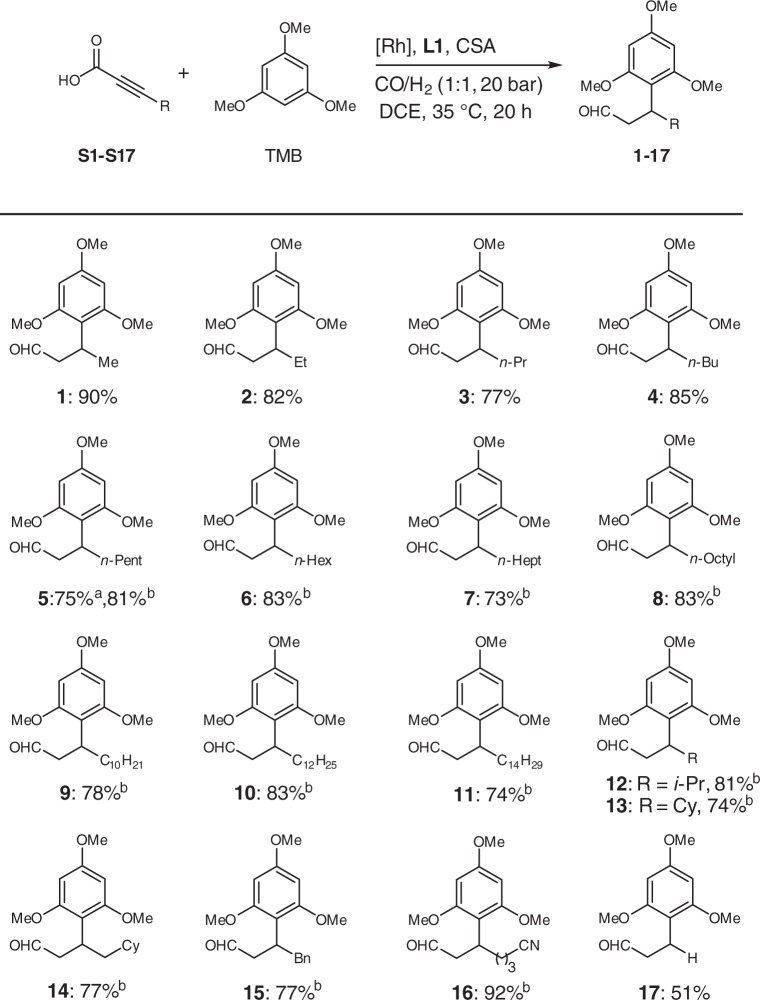
Substrate scope of α-alkynoic acids with TMB^a^

^a^[Rh(CO)_2_acac]/**L1**/CSA/**S**/TMB = 1:6:6:150:100, 1 mmol of TMB, DCE (3 mL). Isolated yield based on TMB. ^b^The ratio of **S** to TMB = 2:1. Bn = Benzyl

As a ubiquitous structural element, the motif of indole is frequently found in bioactive natural products and pharmaceuticals^56,57^. Encouraged by this fact, we decided to evaluate our supramolecular catalytic system in the presence of indole derivatives as nucleophiles (Table 3). Using modified reaction conditions (see Supplementary Table 8), 1-methylindole reacted well with **S1**, leading to the 3-(1-methyl-1H-indol-3-yl)butanal **18** in 81% yield. The chain length (R = *n*-C_14_H_19_) or bulkier substituent (R = *i-*Pr) of α-alkynoic acids slightly affected the reaction efficiency (**19** and **20**, 77% and 76%, respectively). Unfortunately, free indole cannot be transformed under these conditions. However, a variety of protective groups such as Bn, PMB, and TIPS at 1-position of indole were well tolerated (**21**–**23**, 81–90% yield) overcoming this limitation by subsequent deprotection. 1-Pivaloylindole showed no activity under the same reaction conditions. Different substitution patterns at 5-position of the 1-methylindole were evaluated: the electron donating groups Me (**25**) and OMe (**26**) gave just moderate yields (63% and 56% yield, respectively). On the other hand, electron poor substituents such as F (**27**) and Br (**28**) resulted in very good yields (86% and 82% yield, respectively). It should be noticed that indolyl bromide **28** might be further functionalized by transition metal-catalyzed cross-coupling reactions^58^. Substrates with substituents such as Me (**29**, 48% yield) and Ph (**30**, 88% yield) at 2-position of the indole ring were also successfully transformed. Finally, other arene nucleophiles were tested: 1-(dimethylamino)-3-methoxybenzene showed high activity affording product **31** in 90% yield and when dimethylaniline was used, **32** was obtained in 66% yield. However, 1, 3-dimethoxybenzene did not react under these reaction conditions to **33**.

**Table 3:**
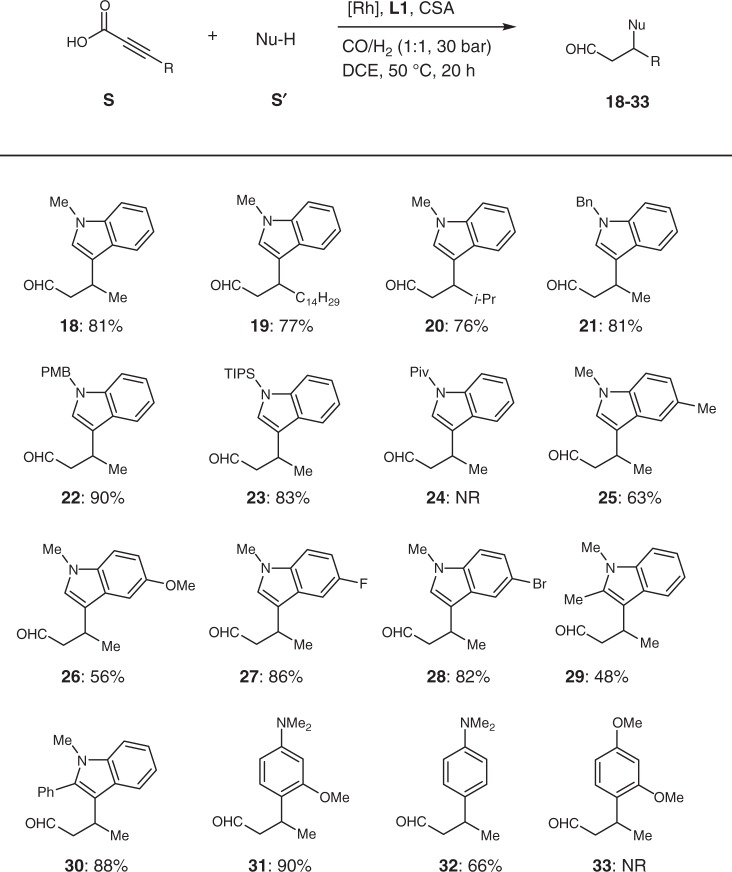
Substrate scope of indole derivatives and other nucleophiles^a^

^a^[Rh(CO)_2_acac]/**L1**/CSA/**S**/Nu-H = 1:6:6:150:100, 0.5 mmol scale of Nu-H, DCE (3 mL). Isolated yield based on used nucleophile. *Piv* Pivaloyl, *PMB* 4-methoxybenzyl, *TIPS* Triisopropylsilyl

**Fig. 2:**
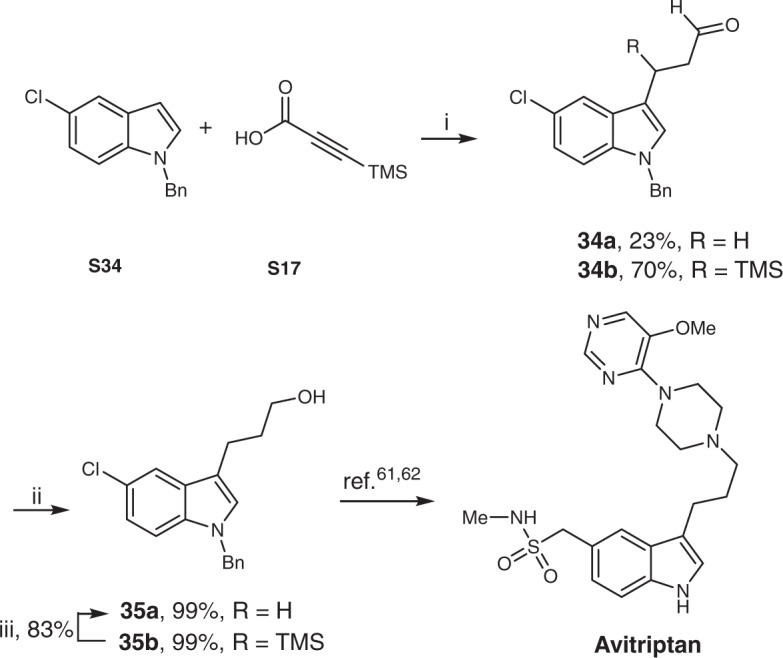
Synthesis of intermediate **35a** for Avitriptan. i: [Rh(CO)_2_acac]/**L1**/CSA/**S17**/**S34** = 1:6:6:150:100, 0.5 mmol scale of **S34**, CO/H_2_ (1:1, 30 bar), DCE (3 mL), 50 °C, 20 h. ii: NaBH_4_ (2 equiv.), MeOH, 0 °C, 30 min. iii: KO*t*Bu (10 mol%), 18-crown-6 (10 mol%), DMSO:H_2_O (19:1, v/v), 80 °C, 5 h. TMS = trimethylsilyl

To further explore the potential application of our protocol in biologically active targets, a concise synthesis of a key intermediate **35a** for Avitriptan^59^, as an attractive candidate for an antimigraine drug, was shown in Fig. 2. Under the optimal reaction conditions, benzyl protected 5-chloroindole **S34** reacted well with 3-(trimethylsilyl)propynoic acid (**S17**), providing the desired β-aryl aldehydes **34** in 93% (*n*(**34a**):*n*(**34b**) = 1:3) yields. The corresponding alcohols **35** were obtained in quantitative yields after simple reduction using NaBH_4_. Notably, **35b** was easily transformed into **35a** with a catalytic amount of KO*t*Bu^60^. Following the literatures known steps^61,62^, Avitriptan could be successfully achieved.

## Discussion

To clarify the role of **L1** in this domino reaction process, several control experiments were undertaken as shown in Table 4. With a phenyl ring analog of **L1**, ligand **L6**, which may also allow for supramolecular substrate–ligand interactions, only poor yields and conversions were observed and it indicated the pyridyl ring of **L1** is key to the catalytic activity (Table 4, entry 2). Furthermore, the combination of 2-pyridinyl-bis-[3’,5’-bis(trifluoromethyl)phenyl]phosphine **L7** and acylguanidine **L8** resulted in no formation of the desired product (Table 4, entry 3). This suggests that the molecular-recognition function and the catalytic unit must be an integral part of the same molecular catalyst, to achieve the unique catalytic activity and selectivity. Using a rhodium catalyst derived from the monodentate ligand **L9**, only very low conversion ( < 2%) of both substrates was observed (Table 4, entry 4). We assumed that the substrate recognition via hydrogen bonding is crucial for our catalytic system. As expected, when MeOH was used as a solvent, the reactivity dramatically dropped, because the essential hydrogen bonding interactions between the catalyst and the substrate were disturbed (Table 4, entry 5). Moreover, using methyl ester **S36**, without the complementary acid functionality, no formation of desired β-aryl aldehyde was observed (Table 4, entry 6). When the reaction was carried out under argon instead of syngas atmosphere, the **L1**-derived catalyst showed basically no activity and the addition reaction of TMB to the triple bond of **S1** was not observed (Table 4, entry 7). This suggests that the HF process is the first step of the reaction sequence rather than the addition reaction. When the reaction of **S1** proceeded without addition of TMB, poor conversion (42%, Table 4, entry 8) was observed and interestingly aldehyde III (Fig. 1c) was observed in 3% yield with poor selectivity (see Supplementary Fig. 3). This indicates that TMB could efficiently trap the intermediate generated from the HF process. Finally, when the isotopically labeled substrate [1–^13^C]-2-nonynoic acid **S37** was subjected to the reaction conditions, product **6** (without isotopic label) was observed (Table 4, entry 9). This is a clear proof that the aldehyde group was not obtained by hydrogenation of the carboxylic acid.

**Table 4:** Control experiments^a^


Entry	Ligand	Substrate (S)	Yield (%)	Conv. (S/TMB) (%)
1	**L1**	**S1**	90(90)	95/95
2	**L6**	**S1**	16	36/36
3	**L7/L8**	**S1**	0	7/9
4	**L9**	**S1**	0	2/2
5^b^	**L1**	**S1**	<4	15/18
6	**L1**	**S36**	0	60/1
7^c^	**L1**	**S1**	0	6/2
8^d^	**L1**	**S1**	-	42/-
9^e^	**L1**	**S37**	78	81/89

^a^1 mmol scale of TMB, [Rh(CO)_2_acac]/**L1**/CSA/**S**/TMB = 1:6:6:150:100, DCE (3 mL). Yield and conversion were determined by NMR spectroscopy using DMAc as the internal standard and isolated yields based on TMB were shown within parentheses. ^b^MeOH was used instead of DCE. ^c^Argon (1 bar) was used instead of syngas. ^d^Without TMB. ^e^[Rh(CO)_2_acac]/**L1**/CSA/**S37**/TMB = 1:6:6:200:100

**Fig. 3:**
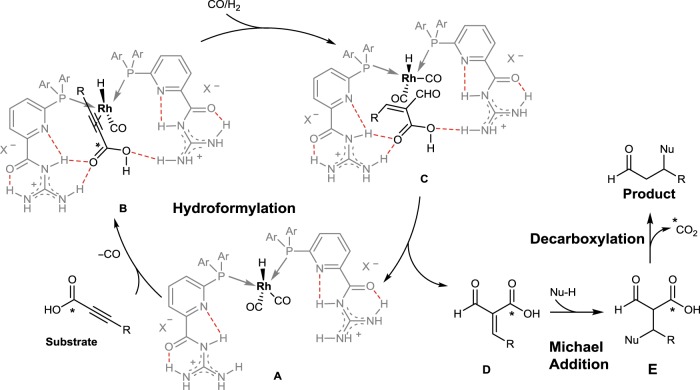
Proposed reaction mechanism. It includes Rh-catalyzed hydroformylation of α-alkynoic acids, Michael addition of arene nucleophiles, and decarboxylation

High-pressure in situ infrared (IR) and in situ NMR experiments were further carried out to study the reaction mechanism. As shown in Supplementary Fig. 17, the formation of characteristic signals (*δ* = − 10.28 p.p.m., *J*_H-P_ = 12.2 Hz) of Rh-hydride species was observed as a triplet peak, which indicated that two **L1** ligands coordinated to the Rh center. The coupling constant of Rh-P (*J*_Rh-P_ = 164.2 Hz, Supplementary Fig. 18) indicated a trigonal bipyramidal structure with an equatorial–equatorial conformation for the related Rh complex^17,63,64^. Furthermore, the formation of product could be easily followed from the high-pressure in situ IR spectroscopy (Supplementary Fig. 9). Based on the generally accepted mechanism of Rh-catalyzed HF in the presence of the triarylphosphine ligand and the presented results, we propose a sequential mechanism consisting of three consecutive steps: HF, Michael addition, and decarboxylation (Fig. 3)^65^. The mechanism might start by the coordination of the substrate to the Rh-complex **A** affording complex **B**, suggested by DFT calculation (see Supplementary Fig. 19), where the substrate is activated via recognition and preorientation to the Rh center. Due to this interaction, an α-selective HF could take place giving rise to intermediate **C**^41^. Then, intermediate **D** could be released regenerating the initial Rh complex **A**. Intermediate **D** might further proceed via Michael addition of an arene nucleophile to intermediate **E**, followed by the decarboxylation process affording the final product.

**Fig. 4:**
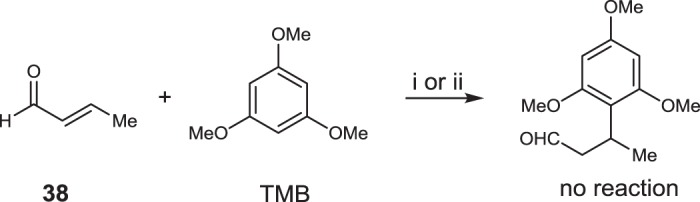
Controlled experiments of the Michael addition. i: [Rh(CO)_2_acac]/**L1**/CSA/**38**/TMB = 1:6:6:150:100, 0.5 mmol scale of TMB, DCE (1.5 mL), 35 °C, 24 h, Argon. ii: *n*(**38**):*n*(TMB) = 1.5:1, 1 mmol scale of TMB, DCE (2 mL), 35 °C, 24 h, Argon

The order of this reaction pathway was further confirmed by the control experiments shown in Fig. 4. The α,β-unsaturated aldehyde (**38**) and TMB were subjected to the optimized reaction conditions with (i) and without (ii) the catalyst. In none of these cases, product formation was observed. This provided a solid support that the Michael addition occurs before the decarboxylation step.

In conclusion, we have successfully developed a domino process from easily accessible α-alkynoic acids, which yields valuable β-aryl aldehydes with high molecular complexity tolerating a variety of functional groups. Furthermore, the synthesis of a key intermediate of Avitriptan using this protocol was accomplished. Based on our study of the reaction mechanism, this domino reaction is triggered by the Rh-catalyzed HF of α-alkynoic acids promoted by hydrogen-bonding interaction between the ligand and the substrate. Consecutive Michael addition of arenes as nucleophiles followed by decarboxylation of the carboxyl function leads to the β-aryl aldehyde products. It should be noticed that the carboxyl function plays a crucial role as a transient and traceless directing group for the introduction of the aldehyde function. This study also highlights the unique advantages of the substrate recognition-directed catalysis in combination with domino reactions. Investigations for an asymmetric version of this protocol are currently being undertaken in our lab.

## Methods

### General procedure *D* for the catalysis reaction

To a flame dried glass liner (Supplementary Fig. 2a) containing a magnetic stirring bar, [Rh(CO)_2_acac], ligand **L1**, 1,3,5-trimethoxybenzene (TMB) (or other nucleophiles), and CSA were added subsequently. The glass liner was sealed by an aluminum crimp cap with silicon septum (Supplementary Fig. 2a) and argon was purged for 5 min via syringes (Supplementary Fig. 2b). DCE was added followed by the liquid substrate (if the substrate was a solid, it was added before purging argon) via a syringe under argon atmosphere. The reaction mixture was stirred for 10 min (a clear reaction solution was obtained; Supplementary Fig. 2c). The glass liner was transferred into the Premex stainless steel autoclave Medimex (100 mL) under argon atmosphere quickly. The autoclave was purged three times with 5 bar of synthesis gas (CO/H_2_, 1:1) and was pressurized to 20 bar (or 30 bar). Then, it was conducted at 35 °C (or 50 °C) for 20 h. Afterwards, the autoclave was cooled down to room temperature and depressurized. Then, 0.5 equiv. of dimethylacetamide (DMAc, 43.6 mg) was added as the internal standard into the crude reaction mixture. Samples were analyzed by NMR analysis after the evaporation of solvent. The corresponding aldehydes were purified by flash chromatography to afford analytically pure products.

Caution: All operations involving carbon monoxide must be carried out in a well-ventilated fume-hood. Use of a gas-leak detector for carbon monoxide is highly recommended.

## Supplementary information

Supplementary Information

## Data Availability

For experimental details and procedures, spectra for all unknown compounds and details from DFT calculation, see Supplementary Files. All data underlying the findings of this study are available from the corresponding author upon reasonable request.
