# Supplementary material for: A domino reaction for generating β-aryl aldehydes from alkynes by substrate recognition catalysis
Source: Nat Commun. 2019 Oct 25;10:4868. doi: 10.1038/s41467-019-12770-w (PMC6814718; doi:10.1038/s41467-019-12770-w)
Supplement: Supplementary file 1 — Supplementary Information [file 41467_2019_12770_MOESM1_ESM.pdf]

Supplementary Information for

A Domino Reaction for Generating β-Aryl Aldehydes from Alkynes by Substrate Recognition Catalysis

Fang et al

Supplementary Methods

General experimental details

All reagents were commercially available unless otherwise noted. Ligand **L1**[1], 1-picolinoylguanidine (**L8**)[2] were synthesized according to literature procedures. All reactions were carried out under argon atmosphere in dried glassware. Air and moisture sensitive liquids and solutions were transferred via syringe. All solvents were dried and distilled by standard procedures.

Nuclear magnetic resonance (NMR) spectra were acquired on a Bruker AMX 300 (300 MHz for ^{1}H and 121 MHz for ^{31}P), a Bruker AMX 400 (400 MHz and 100 MHz for ^{1}H and ^{13}C respectively) and a Bruker DRX 500 (500 MHz, 126 MHz, 202 MHz and 471 MHz for ^{1}H, ^{13}C, ^{31}P and ^{19}F respectively). All ^{1}H NMR spectra are reported in parts per million (ppm) downfield of TMS and were measured relative to the signals at 7.26 ppm ($CDCl_3$). All ^{13}C NMR spectra were reported in ppm relative to $CDCl_3$ (77.16 ppm), and were obtained with ^{1}H-decoupling. Data for ^{1}H-NMR are reported as follows: chemical shift (δ in ppm), multiplicity (s = singlet; brs = broad singlet; d = doublet; t= triplet; q = quartet; quint = quintet; sext = sextet; hept. = heptet, m = multiplet), coupling constant (Hz), integration. Data for ^{13}C-NMR are reported in terms of chemical shift (δ in ppm). High-resolution mass spectra were obtained on a Finnigan MAT 8200 instrument.

Synthesis and characterization of ligands L6 and L7

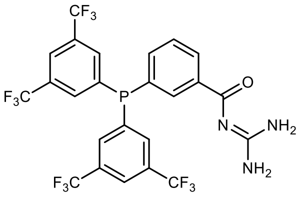

L6
$C_{24}H_{14}F_{12}N_3OP$
Mol. Wt.: 619.3506

***N*-[3-Bis-[3',5'-bis(trifluoromethyl)-phenyl]-phosphanyl-benzoyl]-guanidine (L6)** was synthesized according to literature procedure[2]

BOP (Benzotriazol-1-yloxy)tris(dimethylamino)phosphonium hexafluorophosphate) (155 mg, 0.35 mmol, 1 equiv.) was added to the 3-[bis-[3',5'-bis(trifluoromethyl)phenyl]phosphanyl]benzoic acid (200 mg, 0.35 mmol, 1 equiv.), Boc-protected guanidine (80 mg, 0.52 mmol, 1.5 equiv.) and N-methyl-morpholine (89 mg, 0.88 mmol, 2.5 equiv.) in dry DMF (5 mL) at 0°C. The reaction mixture was stirred at 0°C for 1 h, then for 3 h at room temperature under argon. The conversion was controlled by TLC (CH_2Cl_2/ ethyl acetate = 10:1). After addition of water (40 mL) at 0°C, the product precipitated (stirred 10 min. at 0°C). The precipitate was collected by filtration and washed with water (20 mL). Then it was dissolved in CH_2Cl_2, and filtrated. The filtrate was extracted with water, dried over anhydrous Na_2SO_4 and the solvent was evaporated. The crude product was purified by

column chromatography (CH_2Cl_2/EE = 10:1), which gave a white foam. The foam was triturated with *n*-pentane (2 mL) at -30°C, which gave 160 mg (68%) of a white solid. The white solid was further dissolved in trifluoroacetic acid (2 mL) under argon and the solution was stirred at room temperature for 3 h (TLC control: CH_2Cl_2/MeOH/Et_3N = 30:2:1 / Mo-Ce reagent). The excess of trifluoroacetic acid was removed in vacuum. The residue was dissolved in CH_2Cl_2 (2 mL) and Na_2CO_3 (20% aq., 5 mL) was added at 0 ºC. The two phases mixtures were vigorously stirred at 0 ºC for 15 min. The organic phase was separated and the aqueous phase was extracted with CH_2Cl_2 (2×10 mL) and the combined organic layers were dried over anhydrous Na_2SO_4 and concentrated to give a white foam. This foam was triturated with *n*-pentane (2×10 mL) and dried in vacuum to give the desired product **L6** as a white solid (110 mg, 57%).

Rf (SiO_2, CH_2Cl_2/MeOH/Et_3N = 30:2:1) = 0.25

^{1}H NMR (500 MHz, acetone-D6): δ = 8.42-8.39 (m, 1H), 8.34-8.31 (m,1H), 8.13-8.12 (m, 2H), 8.00-7.98 (m, 4H), 7.53-7.51 (m, 2H), 7.82-6.25 (brs, 4H).

^{13}C NMR (126 MHz, acetone-D6): δ = 176.4, 164.5, 141.5 (d, *J* = 18.8 Hz), 141.3 (d, *J* = 9.3 Hz), 136.6 (d, *J* = 14.7 Hz), 136.2 (d, *J* = 30.3 Hz), 134.4 (dq, *J* = 20.4 Hz, 3.3 Hz), 133.2 (d, *J* = 10.3 Hz), 132.5 (qd, *J* = 33.3 Hz, 6.1 Hz), 132.1, 129.9 (d, *J* = 6.1 Hz), 124.2 (q, *J* = 272.4 Hz), 124.1 (m).

^{31}P NMR (121 MHz, acetone-D6): δ = -3.8.

^{19}F NMR (282.MHz, acetone-D6): δ = -63.5.

HR-MS (ESI, m/z): calcd for $C_{24}H_{15}F_{12}N_3OP$ $[M+H]^+$: 620.0756. Found: 620.0747.

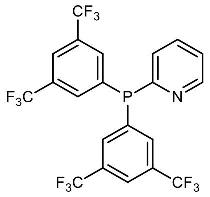

L7
$C_{21}H_{10}F_{12}NP$
Mol. Wt.: 535.2726

2-Pyridyl-bis-[3',5'-bis(trifluoromethyl)phenyl]phosphine (L7) was synthesized according to literature procedure[2]

To a solution of 2-bromopyridine (158 mg, 1 mmol, 1 equiv.) in CH_2Cl_2 (8 mL) under argon was added slowly n-BuLi (0.4 mL, 1 mmol, 2.5 M in hexane, 1.0 equiv.) at -78°C. The mixture was stirred for 30 min., then Ar_2PCl (0.8 equiv.) was added dropwise, and the reaction mixture was stirred for further 30 min. at -78°C. The solution was allowed to warm to r.t. overnight. Then, the reaction was quenched with water (5 mL), and the two phases were separated. The water phase was extracted with CH_2Cl_2 (2×20 mL) and the collected organic phases were dried over anhydrous dry Na_2SO_4 and evaporated, which gave a yellowish oily liquid. The crude product was further

purified by column chromatography (CH_2Cl_2/petroleum ether = 1:5) which gave the desired product **L7** as a colorless oil (90 mg, 21%).

Rf (SiO_2, CH_2Cl_2/petroleum ether = 1:5) = 0.3

^{1}H NMR (500 MHz, acetone-D6): δ = 8.77 (dt, *J* = 4.8 Hz, 1.4 Hz, 1H), 7.91-7.90 (m, 4H), 7.89-7.88 (m, 2H), 7.72 (tdd, *J* = 7.7 Hz, 2.7 Hz, 1.9 Hz, 1H), 7.42 (ddt, *J* = 7.7 Hz, 4.6 Hz, 1.1 Hz, 1H), 7.33 (ddt, *J* = 7.7 Hz, 4.8 Hz, 1.3 Hz, 1H).

^{13}C NMR (126 MHz, acetone-D6): δ = 159.0, 151.5 (d, *J* = 8.7 Hz), 139.1 (d, *J* = 16.8 Hz), 136.7 (d, *J* = 7.8 Hz), 134.0 (dq, *J* = 20.8 Hz, 3.3 Hz), 132.2 (qd, *J* =50.3 Hz, 6.8 Hz), 130.1 (d, *J* = 34.6 Hz), 124.3, 123.7 (m), 123.2 (q, *J* = 273.2 Hz).

^{31}P NMR (121 MHz, acetone-D6): δ = -4.9.

^{19}F NMR (471 MHz, acetone-D6): δ = -63.1.

HR-MS (ESI, m/z): calcd for $C_{21}H_{11}F_{12}NP$ $[M+H]^+$: 536.0432. Found: 536.0439.

Synthesis and characterization of alkynoic acids.

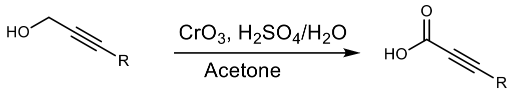

General procedure A[3,4]: Jones reagent was prepared by dissolving 7.5 g of CrO_3 in conc. H_2SO_4 (7.5 mL). This solution was added dropwise to water (22.5 mL) at 0 ºC, which yielded the Jones reagent (2.5 M, 30 mL).

To a solution of the alcohol (20 mmol) in 250 mL of acetone at 0 °C, Jones reagent (2.2 equiv. 2.5 M) was added dropwise, and the obtained reaction mixture was stirred for 3 hours at 0 °C. Then it was quenched by adding *i*-PrOH (80 mL). The Cr salts were filtered off and the organic phase was concentrated by evaporation. Water (100 mL) was added and the aqueous phase was extracted with CH_2Cl_2 (5 × 100 mL). Then the combined organic layers were dried over anhydrous Na_2SO_4 and concentrated in vacuum. Purification by column chromatography (petroleum ether/ethyl acetate/AcOH = 100:10:1) gave the title compounds as yellow oils. The desired products colorless oils could be obtained after further distillation under high vacuum (details of conditions see each substrates).

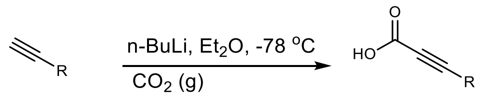

General procedure B[5,6]: To a solution of 1-alkynes (10 mmol, 1 equiv.) in dry Et_2O under argon was added dropwise *n*-BuLi (2.5 M in hexane, 11 mmol, 1.1 equiv.) at -78°C, and the mixture was

stirred at the same temperature for 1 h. Then CO_2 (g) was bubbled through the solution for 1 h at -78 °C. The cooling bath was removed and the temperature was slowly increased to r.t. overnight. The reaction was quenched with 1 M HCl at 0 °C, and extracted with Et_2O (3×). The collected organic phases were dried over anhydrous Na_2SO_4 and evaporated. Purification by column chromatography (petroleum ether/ethyl acetate/AcOH = 100:10:1) gave the title compounds as yellow oils or solids. The desired products as white solids or colorless oils could be obtained after further purification (details of conditions see each substrates).

2-Heptynoic acid (S4)

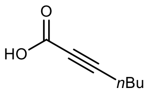

S4
$C_7H_{10}O_2$
Mol. Wt.: 126.1531

Following the **General procedure A**, **2-heptynoic acid (S4)** was obtained as a colorless oil in 85% yield after the distillation under high vacuum (condition: 0.49 mbar, 85-88 ^{o}C).

^{1}H NMR (400 MHz, $CDCl_3$): δ = 11.09 (brs, 1H), 2.36 (t, *J* = 7.1 Hz, 2H), 1.61-1.53 (m, 2H), 1.48-1.39 (m, 2H), 0.92 (t, *J* = 7.3 Hz, 3H).

^{13}C NMR (100 MHz, $CDCl_3$): δ = 158.7, 93.0, 72.8, 29.5, 22.0, 18.6, 13.5.

2-Nonynoic acid (S6)

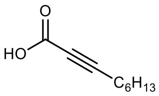

S6
$C_9H_{14}O_2$
Mol. Wt.: 154.2063

Following the **General procedure A**, **2-nonynoic acid (S6)** was obtained as a colorless oil in 85% yield after the distillation under high vacuum (condition: 0.49 mbar, 110-113 ^{o}C).

^{1}H NMR (400 MHz, $CDCl_3$): δ = 11.02 (brs, 1H), 2.35 (t, *J* = 7.1 Hz, 2H), 1.62-1.55 (m, 2H), 1.44-1.35 (m, 2H), 1.34-1.23 (m, 4H), 0.89 (t, *J* = 7.0 Hz, 3H).

^{13}C NMR (100 MHz, $CDCl_3$): δ = 158.7, 93.0, 72.8, 31.3, 28.6, 27.5, 22.5, 18.9, 14.1.

2-Decynoic acid (S7)

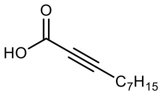

S7
$C_{10}H_{16}O_2$
Mol. Wt.: 168.2328

Following the **General procedure A**, **2-decynoic acid (S7)** was obtained as a colorless oil in 65% yield after the distillation under high vacuum (condition: 0.5 mbar, 105-110 ^{o}C).

^{1}H NMR (400 MHz, $CDCl_3$): δ = 11.11 (brs, 1H), 2.35 (t, *J* = 7.1 Hz, 2H), 1.62-1.55 (m, 2H), 1.43-1.36 (m, 2H), 1.34-1.23 (m, 6H), 0.88 (t, *J* = 6.9 Hz, 3H).

^{13}C NMR (100 MHz, $CDCl_3$): δ = 158.6, 93.0, 72.8, 31.7, 28.9, 28.7, 27.5, 22.7, 18.9, 14.1.

2-Undecynoic acid (S8)

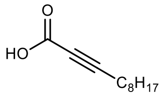

S8
$C_{11}H_{18}O_2$
Mol. Wt.: 182.2594

Following the **General procedure B**, **2-undecynoic acid (S8)** was obtained as a colorless oil in 77% yield after the distillation under high vacuum (condition: 0.49 mbar, 110 ^{o}C).

^{1}H NMR (400 MHz, $CDCl_3$): δ = 10.87 (brs, 1H), 2.34 (t, *J* = 7.1 Hz, 2H), 1.62-1.54 (m, 2H), 1.43-1.35 (m, 2H), 1.31-1.23 (m, 8H), 0.87 (t, *J* = 6.9 Hz, 3H).

^{13}C NMR (100 MHz, $CDCl_3$): δ = 158.8, 93.0, 72.8, 31.9, 29.2, 29.1, 28.9, 27.5, 22.7, 18.9, 14.1.

2-Tridecynoic acid (S9)

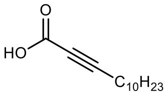

S9
$C_{13}H_{22}O_2$
Mol. Wt.: 210.3126

Following the **General procedure B**, **2-tridecynoic acid (S9)** was obtained as a white solid in 78% yield (condition: 0.5 mbar, 115-120 ^{o}C).

^{1}H NMR (400 MHz, $CDCl_3$): δ = 10.99 (brs, 1H), 2.34 (t, *J* = 7.1 Hz, 2H), 1.62-1.55 (m, 2H), 1.41-1.36 (m, 2H), 1.33-1.23 (m, 12H), 0.87 (t, *J* = 6.9 Hz, 3H).

^{13}C NMR (100 MHz, $CDCl_3$): δ = 158.8, 93.0, 72.8, 32.0, 29.6, 29.5, 29.4, 29.1, 28.9, 27.5, 22.8, 18.9, 14.2.

2-Pentadecynoic acid (S10)

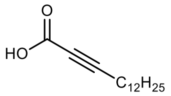

S10
$C_{15}H_{26}O_2$
Mol. Wt.: 238.3657

Following the **General procedure B**, **2-pentadecynoic acid (S10)** was obtained as a white solid in 80% yield after the distillation under high vacuum (condition: 0.5 mbar, 130 ^{o}C).

^{1}H NMR (400 MHz, $CDCl_3$): δ = 11.00 (brs, 1H), 2.35 (t, *J* = 7.1 Hz, 2H), 1.62-1.55 (m, 2H), 1.43-1.36 (m, 2H), 1.33-1.23 (m, 16H), 0.88 (t, *J* = 6.9 Hz, 3H).

^{13}C NMR (100 MHz, $CDCl_3$): δ = 158.8, 93.0, 72.8, 32.0, 29.8, 29.7, 29.5, 29.4, 29.1, 28.9, 27.5, 22.8, 18.9, 14.2.

2-Heptadecynoic acid (S11)

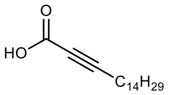

S11
$C_{17}H_{30}O_2$
Mol. Wt.: 266.4189

Following the **General procedure B**, **2-heptadecynoic acid (S11)** was obtained as a white solid in 91% yield (condition: 0.5 mbar, 130-135 ^{o}C).

^{1}H NMR (400 MHz, $CDCl_3$): δ = 10.70 (brs, 1H), 2.35 (t, *J* = 7.1 Hz, 2H), 1.62-1.55 (m, 2H), 1.43-1.36 (m, 2H), 1.33-1.21 (m, 20H), 0.88 (t, *J* = 6.9 Hz, 3H).

^{13}C NMR (100 MHz, $CDCl_3$): δ = 158.7, 93.0, 72.8, 32.1, 29.8(3), 29.7(9), 29.7(7), 29.5(4), 29.4(9), 29.1, 29.0, 27.5, 22.8, 18.9, 14.2.

4-Methyl-2-pentynoic acid (S12)

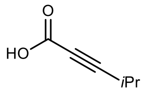

S12
$C_6H_8O_2$
Mol. Wt.: 112.1265

Following the **General procedure B**, **4-methyl-2-pentynoic acid (S12)** was obtained as a white solid in 78% yield (condition: 0.65 mbar, 75-80 ^{o}C).

^{1}H NMR (500 MHz, $CDCl_3$): δ = 11.17 (brs, 1H), 2.71 (hept., *J* = 6.9 Hz, 1H), 1.24 (d, *J* = 6.9 Hz, 6H).

^{13}C NMR (126 MHz, $CDCl_3$): δ = 159.0, 97.4, 71.9, 21.7, 20.7.

3-Cyclohexyl-2-propynoic acid (S13)

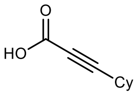

S13
$C_9H_{12}O_2$
Mol. Wt.: 152.1904

Following the **General procedure B**, **3-cyclohexyl-2-propynoic acid (S13)** was obtained as a colorless oil in 90% yield (condition: 0.5 mbar, 120 ^oC).

^{1}H NMR (400 MHz, $CDCl_3$): δ = 10.15 (brs, 1H), 2.57-2.50 (m, 1H), 1.86-1.79 (m, 2H), 1.73-1.66 (m, 2H), 1.56-1.47 (m, 3H), 1.39-1.29 (m, 3H).

^{13}C NMR (100 MHz, $CDCl_3$): δ = 158.8, 96.2, 72.7, 31.4, 29.0, 25.6, 24.6.

4-Cyclohexyl-2-butynoic acid (S14)

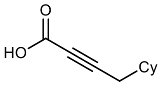

S14
$C_{10}H_{14}O_2$
Mol. Wt.: 166.2170

Following the **General procedure B**, **4-cyclohexyl-2-butynoic acid (S14)** was obtained as a white solid in 92% yield (condition: 0.5 mbar, 130-135 ^oC).

^{1}H NMR (400 MHz, $CDCl_3$): δ = 11.20 (brs, 1H), 2.25 (d, *J* = 6.7 Hz, 2H), 1.83-1.78 (m, 2H), 1.76-1.70 (m, 2H), 1.69-1.62 (m, 1H), 1.61-1.52 (m, 1H), 1.31-1.12 (m, 3H), 1.10-0.97 (m, 2H).

^{13}C NMR (100 MHz, $CDCl_3$): δ = 158.7, 92.1, 73.7, 36.7, 32.8, 26.6, 26.1, 26.0.

4-Phenyl-2-butynoic acid (S15)

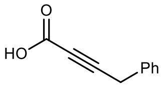

S15
$C_{10}H_8O_2$
Mol. Wt.: 160.1693

Following the **General procedure B**, **4-phenyl-2-butynoic acid (S15)** was obtained as a pale yellow solid in 73% yield (condition: 0.5 mbar, 130-135 ^oC).

^{1}H NMR (400 MHz, $CDCl_3$): δ = 11.17 (brs, 1H), 7.29-7.16 (m, 8H), 3.68 (d, *J* = 0.5 Hz, 2H).

^{13}C NMR (100 MHz, $CDCl_3$): δ = 158.5, 133.7, 129.0, 128.1, 127.4, 89.7, 74.4, 25.2.

6-Cyano-2-hexynoic acid (S16)

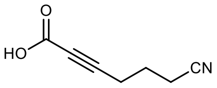

S16
$C_7H_7NO_2$
Mol. Wt.: 137.1360

Following the **General procedure B**, **6-cyano-2-hexynoic acid (S16)** was obtained as a pale yellow solid in 90% yield (condition: 0.5 mbar, 130-135 ^oC).

^{1}H NMR (400 MHz, $CDCl_3$): δ = 10.45 (brs, 1H), 2.55 (t, *J* = 6.9 Hz, 2H), 2.51 (t, *J* = 7.2 Hz, 2H), 1.95 (quint, *J* = 7.0 Hz, 2H).

^{13}C NMR (100 MHz, $CDCl_3$): δ = 157.0, 118.6, 88.7, 74.1, 23.4, 17.9, 16.3.

(1-^{13}C)-2-Nonynoic acid (S37)

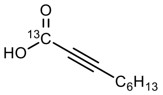

S37
$C_8{}^{13}CH_{14}O_2$
Mol. Wt.: 155.2014

Following the **General procedure B**, using $^{13}CO_2$ (1 L in Sure/Pac cylinder, >99 atom% ^{13}C, < 3 atom%^{18}O) instead of CO_2, **(1-^{13}C)-2-nonynoic acid (S37)** was obtained as a colorless oil in 80% yield (condition: 0.5 mbar, 110-113 ^oC).

^{1}H NMR (400 MHz, $CDCl_3$): δ = 10.40 (brs, 1H), 2.35 (td, *J* = 7.1 Hz, 1.7 Hz, 2H), 1.62-1.55 (m, 2H), 1.44-1.36 (m, 2H), 1.35-1.25 (m, 4H), 0.89 (t, *J* = 7.0 Hz, 3H).

^{13}C NMR (100 MHz, $CDCl_3$): δ = 158.6, 93.0 (d, *J* = 19.8 Hz), 72.7 (d, *J* = 125.0 Hz), 31.3, 28.6, 27.5, 22.5, 18.9 (d, *J* = 1.7 Hz), 14.1.

HR-MS (ESI, m/z): calcd for $C_{13}H_{19}O_4$ $(M-H)^-$: 154.0949. Found: 154.0955.

Synthesis and characterization of indole derivatives.

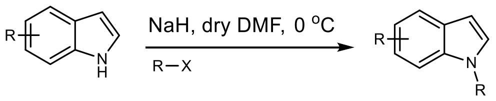

General procedure C[7]: To a solution of indole (5 mmol, 1 equiv.) in dry DMF (15 mL) at 0 ^oC was added NaH (60% dispersion in oil, 10.0 mmol, 1.2 equiv.) portionwise under argon atmosphere and the resulted mixture was stirred for 1 h at the same temperature. Then R-X (6.5 mmol, 1.3 equiv.) was added under 0 ^oC. The reaction mixture was stirred overnight. Then 10 mL saturated

NH_4Cl solution was added slowly and the mixture was extracted with ethyl acetate (3×20 mL). The combined organic layers were dried over anhydrous Na_2SO_4, and concentrated in vacuo. Purification by column chromatography (petroleum ether/ethyl acetate, 30:1 to 15:1, v/v) gave the title compounds.

1-Benzyl-1*H*-indole (S21)

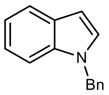

S21
$C_{15}H_{13}N$
Mol. Wt.: 207.2760

Following the **General procedure C**, **1-benzyl-1*H*-indole (S21)** was obtained as a white solid in 80% yields.

^{1}H NMR (400 MHz, $CDCl_3$): δ = 7.76 (ddd, *J* = 7.8 Hz, 1.3 Hz, 0.8 Hz, 1H), 7.39-7.32 (m, 4H), 7.27 (tdd, *J* = 7.0 Hz, 1.3 Hz, 0.3 Hz, 1H), 7.22 (dd, *J* = 7.8 Hz, 1.3 Hz, 1H), 7.20-7.17 (m, 3H), 6.65 (dd, *J* = 3.2 Hz, 0.8 Hz, 1H), 5.36 (s, 2H).

^{13}C NMR (100 MHz, $CDCl_3$): δ = 137.7, 136.5, 128.9, 128.8, 128.3, 127.7, 126.9, 121.8, 121.1, 119.7, 109.8, 101.8, 50.2.

1-(4-Methoxybenzyl-1*H*-indole (S22)

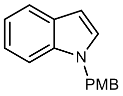

S22
$C_{16}H_{15}NO$
Mol. Wt.: 237.3020

Following the **General procedure C**, **1-(4-methoxybenzyl-1*H*-indole (S22)** was obtained as a white solid in 75 % yields.

^{1}H NMR (500 MHz, $CDCl_3$): δ = 7.64 (dt, *J* = 7.8 Hz, 1.0 Hz, 1H), 7.30 (d, *J* = 8.2 Hz, 1H), 7.17 (ddd, *J* = 8.2 Hz, 7.0 Hz, 1.2 Hz, 1H), 7.12-7.05 (m, 4H), 6.83 (dt, *J* = 6.6 Hz, 2.1 Hz, 1H), 6.53 (d, *J* = 3.1 Hz, 1H), 5.26 (s, 2H), 3.77 (s, 3H).

^{13}C NMR (126 MHz, $CDCl_3$): δ = 159.2, 136.4, 129.7, 128.9, 128.3, 128.2, 121.7, 121.1, 119.6, 114.3, 109.8, 101.7, 55.4, 49.7.

1-(Triisopropylsilyl)-1*H*-indole (S23)

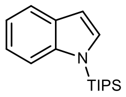

S23
$C_{17}H_{27}NSi$
Mol. Wt.: 273.4950

Following the literature[8], **1-(triisopropylsilyl)-1*H*-indole (S23)** was obtained as a white solid in 33% yield.

^{1}H NMR (400 MHz, $CDCl_3$): δ = 7.64-7.62 (m, 1H), 7.53-7.50 (m, 1H), 7.26 (d, *J* = 3.3 Hz, 1H), 7.16-7.08 (m, 2H), 6.63 (dd, *J* = 3.2 Hz, 0.9 Hz, 1H), 1.77-1.65 (hept., *J* = 7.5 Hz, 3H), 1.15 (d, *J* = 7.5 Hz, 18H).

^{13}C NMR (100 MHz, $CDCl_3$): δ = 141.0, 131.6, 131.3, 121.5, 120.7, 119.9, 114.0, 104.9, 18.3, 13.0.

1-Pivaloyl-1*H*-indole (S24)

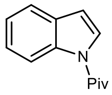

S24
$C_{13}H_{15}NO$
Mol. Wt.: 201.2690

Following procedures in the literature[9], to a flask charged with indole (10.0 mmol, 1 equiv.), 4-(dimethylamino)pyridine (1 mmol, 0.1 equiv.), and triethylamine (15.0 mmol, 1.5 equiv.) was added CH_2Cl_2 (16 mL) and the reaction mixture was cooled at 0 ^oC. Pivaloyl chloride (12 mmol, 1.2 equiv.) was then added dropwise. The reaction mixture was stirred at r.t. for 16 h, filtered through a plug of silica, and concentrated in vacuo. The residue was purified by column chromatography (CH_2Cl_2/MeoH = 24:1) to afford product **1-pivaloyl-1*H*-indole (S24)** as a white solid in 99% yield.

^{1}H NMR (400 MHz, $CDCl_3$): δ = 8.57 (dq, *J* = 8.3 Hz, 0.9 Hz, 1H), 7.75 (d, *J* =3.8 Hz, 1H), 7.59 (dq, *J* = 7.7 Hz, 0.7 Hz, 1H), 7.40-7.36 (m, 1H), 7.31-7.27 (m, 1H), 6.64 (dd, *J* = 3.9 Hz, 0.8 Hz, 1H), 1.54 (s, 9H).

^{13}C NMR (100 MHz, $CDCl_3$): δ = 177.1, 136.9, 129.5, 125.7, 125.2, 123.6, 120.6, 117.4, 108.3, 41.3, 28.8.

1,5-Dimethyl-1*H*-indole (S25)

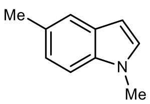

S25
$C_{10}H_{11}N$
Mol. Wt.: 145.2050

Following the **General procedure C**, **1,5-dimethyl-1*H*-indole (S25)** was obtained as a white solid in 66 % yields.

^{1}H NMR (400 MHz, $CDCl_3$): δ = 7.42-7.41 (m, 1H), 7.22 (d, *J* = 8.4 Hz, 1H), 7.06 (ddt, *J* = 8.4 Hz, 1.6 Hz, 0.5 Hz, 1H), 7.01 (d, *J* = 2.9 Hz, 1H), 6.40 (dd, *J* = 3.0 Hz, 0.5 Hz, 1H), 3.77 (s, 3H), 2.46 (s, 3H).

^{13}C NMR (100 MHz, $CDCl_3$): δ = 135.3, 128.9(3), 128.9(1), 128.6, 123.3, 120.6, 109.0, 100.4, 32.9, 21.5.

5-Methoxy-1-methyl-1*H*-indole (S26)

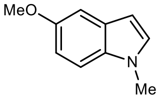

S26
$C_{10}H_{11}NO$
Mol. Wt.: 161.2040

Following the **General procedure C**, **5-methoxy-1-methyl-1*H*-indole (S26)** was obtained as a white solid in 70 % yields.

^{1}H NMR (400 MHz, $CDCl_3$): δ = 7.25 (dt, *J* = 8.8 Hz, *J* = 0.7 Hz,1H), 7.15 (d, *J* = 2.3 Hz, 1H), 7.05 (d, *J* = 3.0 Hz, 1H), 6.95 (ddd, *J* = 8.8 Hz, 2.5 Hz, 0.4 Hz, 1H), 6.45 (dd, *J* = 3.0 Hz, 0.9 Hz, 1H), 3.90 (s, 3H), 3.78 (s, 3H).

^{13}C NMR (100 MHz, $CDCl_3$): δ = 154.2, 132.3, 129.4, 128.9, 112.0, 110.0, 102.7, 100.5, 56.0, 33.0.

5-Fluoro-1-methyl-1*H*-indole (S27)

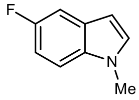

S27
C_9H_8FN
Mol. Wt.: 149.1684

Following the **General procedure C**, **5-fluoro-1-methyl-1*H*-indole (S27)** was obtained as a white solid in 75 % yields

^{1}H NMR (500 MHz, $CDCl_3$): δ = 7.26 (ddd, *J* = 9.7 Hz, 2.5 Hz, 0.3 Hz, 1H), 7.22 (dd, *J* = 8.9 Hz, 4.3 Hz, 1H), 7.08 (d, *J* = 2.9 Hz, 1H), 6.97 (td, *J* = 9.2 Hz, 2.5 Hz, 1H), 6.44 (dd, *J* = 3.1 Hz, 0.5 Hz, 1H), 3.79 (s, 3H).

^{13}C NMR (126 MHz, $CDCl_3$): δ = 158.0 (d, *J* = 234.0 Hz), 133.5, 130.5, 128.7, 110.0 (d, *J* = 34.4 Hz), 105.7 (d, *J* = 23.1 Hz), 101.0 (d, *J* = 4.6 Hz), 33.2.

^{19}F NMR (377 MHz, DMSO-D6): δ = -108 (m).

5-Bromide-1-methyl-1*H*-indole (S28)

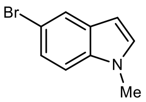

S28
C_9H_8BrN
Mol. Wt.: 210.0740

Following the **General procedure C**, **5-bromide-1-methyl-1*H*-indole (S28)** was obtained as a white solid in 75 % yields.

^{1}H NMR (500 MHz, $CDCl_3$): δ = 7.75 (dd, *J* = 1.9 Hz, 0.6 Hz, 1H), 7.30 (ddd, *J* = 8.7 Hz, 1.9 Hz, 0.3 Hz, 1H), 7.19 (dt, *J* = 8.7 Hz, 0.7 Hz, 1H), 7.04 (d, *J* = 3.1 Hz, 1H), 6.42 (dd, *J* = 3.1 Hz, 0.9 Hz, 1H), 3.77 (s, 3H).

^{13}C NMR (126 MHz, $CDCl_3$): δ = 135.5, 130.3, 130.1, 124.5, 123.4, 112.8, 110.8, 100.7, 33.1.

1,2-Dimethyl-1*H*-indole (S29)

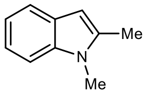

S29
$C_{10}H_{11}N$
Mol. Wt.: 145.2050

Following the **General procedure C**, **1,2-dimethyl-1*H*-indole (S29)** was obtained as a white solid in 65 % yields.

^{1}H NMR (400 MHz, $CDCl_3$): δ = 7.51 (dt, *J* = 7.8 Hz, 1.0 Hz, 1H), 7.25 (dt, *J* = 8.1 Hz, 0.9 Hz, 1H), 7.14 (ddd, *J* = 8.2 Hz, 7.0 Hz, 1.2 Hz, 1H), 7.06 (dt, *J* = 7.8 Hz, 7.0 Hz, 1.1 Hz, 1H), 3.67 (s, 3H), 2.43 (s, 3H).

^{13}C NMR (100 MHz, $CDCl_3$): δ = 137.5, 136.9, 128.1, 120.6, 119.7, 119.4, 108.8, 99.7, 29.5, 12.9.

1-Methyl-2-phenyl-1*H*-indole (S30)

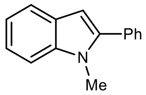

S30
$C_{15}H_{13}N$
Mol. Wt.: 207.2760

Following the **General procedure C**, **1-methyl-2-phenyl-1*H*-indole (S30)** was obtained as a white solid in 86 % yields.

^{1}**H NMR** (400 MHz, $CDCl_3$): δ = 7.76 (ddd, *J* = 7.8 Hz, 1.2 Hz, 0.8 Hz, 1H), 7.63-7.60 (m, 2H), 7.58-7.53 (m, 2H), 7.51-7.45 (m, 2H), 7.36 (ddd, *J* = 8.2 Hz, 7.0 Hz, 1.2 Hz, 1H), 7.27 (ddd, *J* = 7.9 Hz, 7.0 Hz, 1.1 Hz, 1H), 6.68 (s, 1H), 3.82 (s, 3H).

^{13}**C NMR** (100 MHz, $CDCl_3$): δ = 141.7, 138.5, 133.0, 129.5, 128.6, 128.1, 127.9, 121.8, 120.6, 120.0, 109.7, 101.8, 31.2.

1-Benzyl-5-chloro-1*H*-indole (34)

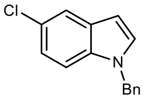

S34
$C_{15}H_{12}ClN$
Mol. Wt.: 241.7180

Following the **General procedure C**, **1-benzyl-5-chloro-1*H*-indole (34)** was obtained as a white solid in 86 % yields.

^{1}**H NMR** (500 MHz, $CDCl_3$): δ = 7.64 (dd, *J* = 2.0 Hz, 0.6 Hz, 1H), 7.34-7.27 (m, 3H), 7.20-7.09 (m, 5H), 6.51 (dd, *J* = 3.2 Hz, 0.8 Hz, 1H), 5.31 (s, 2H).

^{13}**C NMR** (126 MHz, $CDCl_3$): δ = 137.2, 134.8, 129.9, 129.7, 129.0, 127.9, 126.8, 125.4, 122.1, 120.5, 110.9, 101.5, 50.4.

Methyl 2-butynoate (S36)

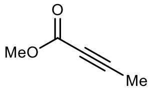

S36
$C_5H_6O_2$
Mol. Wt.: 98.0999

To a solution of **2-butynoic acid** (**S1**, 5 mmol, 420 mg) in 15 mL of MeOH, conc. H_2SO_4 (0.5 mL) was added dropwise, and the resulted reaction mixture was refluxed for 4 hours. Water (20 mL) was added and the aqueous phase was extracted with CH_2Cl_2 (3 × 30 mL). Then the combined organic layers were dried over anhydrous Na_2SO_4 and concentrated in vacuo. Purification by column chromatography (pentane/Et_2O = 40:1) gave the title compound **S36** as a colorless oil (490 mg, yield: >99%).

^{1}**H NMR** (400 MHz, $CDCl_3$): δ = 3.73 (s, 3H), 1.97 (s, 3H).

^{13}**C NMR** (100 MHz, $CDCl_3$): δ = 154.2, 85.8, 72.3, 52.6, 3.8.

Synthesis of key intermediate 35a for Avitriptan

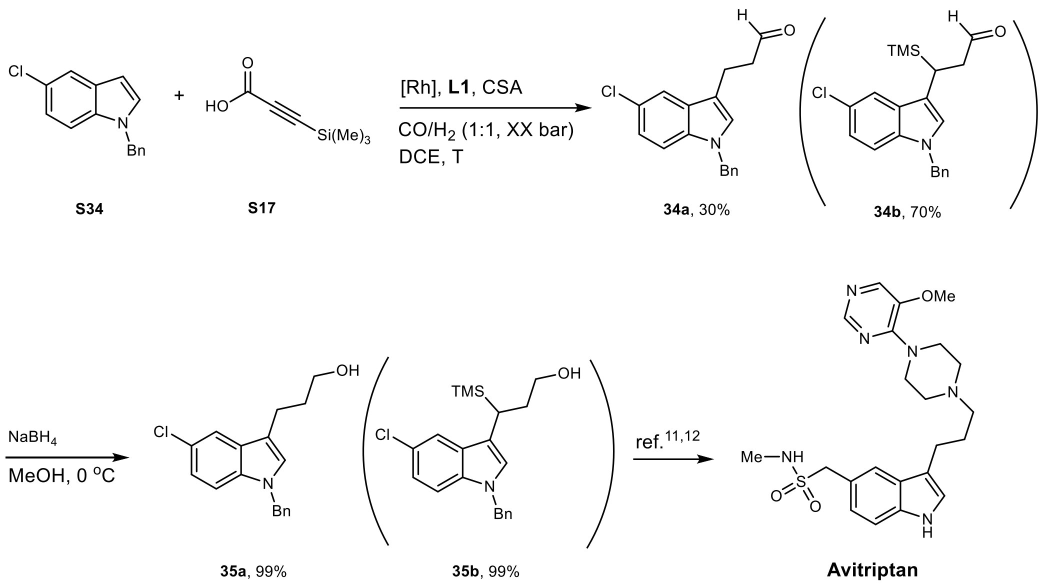

Supplementary Figure 1 Synthesis of key intermediate **35a** for Avitriptan[10-12]

Synthesis of compounds 34: To a dried glass liner (Fig. S1a) containing a magnetic stirring bar, [Rh(CO)$_2$acac] (0.005 mmol, 1.3 mg), ligand **L1** (0.03 mmol, 18.6 mg), CSA (0.03 mmol, 7.0 mg), **S34** (0.5 mmol, 120.9 mg) and 3-(trimethylsilyl)propynoic acid (1.0 mmol, 142.2 mg) were added subsequently. The glass liner was sealed by an aluminum crimp cap with silicon septum (Fig. S1a) and argon was purged for 5 min via syringes (Fig. S1b). DCE (3 mL) was added via a syringe under argon atmosphere. The reaction mixture was stirred for 10 min (a clear reaction solution was obtained, Fig. S1c). The glass liner was transferred into the Premex tainless steel autoclave Medimex (100 mL) under argon atmosphere quickly. The autoclave was purged three times with 5 bar of synthesis gas (CO/H$_2$, 1:1) and was pressurized to 30 bar. Then it was conducted at 50 ^{o}C for 20 h. Afterwards, the autoclave was cooled down to r.t. and depressurized. After the evaporation of solvent, the crude products were purified by flash chromatography (eluted with toluene) to afford analytically pure products (**34a**, 34.6 mg, yield: 23%, **34b**, 130.6 mg, yield: 70%).

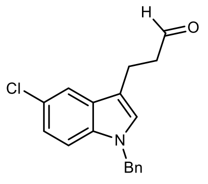

34a
$C_{18}H_{16}ClNO$
Mol. Wt. 297.7820

^{1}H NMR (400 MHz, CDCl$_3$): δ = 9.83 (t, *J* = 1.6 Hz, 1H), 7.55 (dd, *J* = 1.9 Hz, 0.7 Hz, 1H), 7.33-7.27 (m, 3H), 7.17-7.10 (m, 2H), 7.09-7.06 (m, 2H), 6.95 (t, *J* = 0.8 Hz, 1H), 5.24 (s, 2H), 3.09-3.05 (m, 2H), 2.83 (tdd, *J* = 7.26 Hz,1.6 Hz, 0.5 Hz, 2H).

^{13}C NMR (100 MHz, $CDCl_3$): δ = 202.1, 137.3, 135.3, 129.0, 127.9, 127.3, 126.8, 125.2, 122.4, 118.5, 113.7, 111.0, 50.3, 44.1, 17.8.

HR-MS (ESI, m/z): calcd for $C_{18}H_{17}ONCl$ $(M+H)^+$: 298.0993. Found: 298.0995.

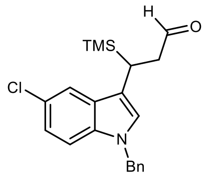

34b
$C_{21}H_{24}ClNOSi$
Mol. Wt.: 369.9640

^{1}H NMR (400 MHz, $CDCl_3$): δ = 9.59 (dd, *J* = 2.8 Hz, 2.0 Hz, 1H), 7.53 (dd, *J* = 1.8 Hz, 0.8 Hz, 1H), 7.31-7.25 (m, 3H), 7.12-7.07 (m, 2H), 7.01-6.98 (m, 2H), 6.81 (s, 1H), 5.25 (d, *J* = 3.0 Hz, 2H), 2.89-2.85 (m, 1H), 2.73 (ddd, *J* = 9.9 Hz, 5.2 Hz, 3.0 Hz, 2H), 0.01 (s, 9H).

^{13}C NMR (100 MHz, $CDCl_3$): δ = 202.3, 137.4, 135.0, 129.3, 129.0, 127.8, 126.5, 226.1, 125.0, 122.3, 119.1, 115.6, 110.9, 50.3, 44.3, 19.7, -2.6.

HR-MS (ESI, m/z): calcd for $C_{21}H_{25}ONClSi$ $(M+H)^+$: 370.1388. Found: 370.1392.

Caution: All operations involving carbon monoxide must be carried out in a well-ventilated fume-hood. Use of a gas-leak detector for carbon monoxide is highly recommended.

Synthesis of compounds 35: The aldehyde product **34a** (or **34b**) was dissolved in MeOH (0.1 M) in a 20 mL round-bottomed flask equipped with a stir bar. The mixture was cooled to 0 °C. Sodium borohydride (2.0 equiv.) was added, and the resulting mixture was stirred for 30 min at 0 °C. The solvent was removed under reduced pressure, and the resulting residue was transferred to a separatory funnel using diethyl ether (20 mL) and water (20 mL). The aqueous layer was extracted with diethyl ether (2 x 20 mL), and the combined organic layers were washed with water (20 mL), and brine (20 mL). The organic layer was then dried over anhydrous Na_2SO_4, and concentrated under reduced pressure. The resulting mixture was purified by flash chromatography (eluted with CH_2Cl_2) to afford analytically pure products (**35a**, 20.1 mg, yield: >99%; **35b**, 97.8 mg, yield: >99%).

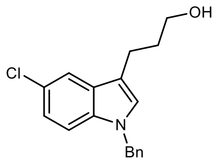

35a
$C_{18}H_{18}ClNO$
Mol. Wt.: 299.7980

^{1}H NMR (400 MHz, $CDCl_3$): δ = 7.58 (dd, *J* = 2.0 Hz, 0.7 Hz, 1H), 7.32-7.26 (m, 3H), 7.16-7.11 (m, 2H), 7.09-7.06 (m, 2H), 6.95 (t, *J* = 1.0 Hz, 2H), 5.24 (s, 2H), 3.72 (t, *J* = 6.4 Hz, 2H), 2.84-2.80 (m, 2H), 2.00-1.93 (m, 2H), 1.44 (brs, 1H).

^{13}C NMR (100 MHz, $CDCl_3$): δ = 137.5, 135.3, 129.4, 128.9, 127.9, 127.0, 126.8, 125.0, 122.1, 118.8, 115.1, 110.8, 62.6, 50.2, 33.1, 21.3.

HR-MS (ESI, m/z): calcd for $C_{18}H_{19}ONCl$ $(M+H)^+$: 300.1150. Found: 300.1148.

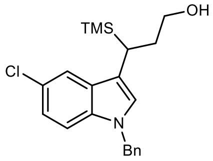

35b
$C_{21}H_{26}ClNOSi$
Mol. Wt.: 371.9800

^{1}H NMR (400 MHz, $CDCl_3$): δ = 7.51 (dd, *J* = 1.9 Hz, 0.6 Hz, 1H), 7.31-7.22 (m, 3H), 7.11 (dd, *J* = 8.7 Hz, 0.6 Hz, 1H), 7.07 (dd, *J* = 8.7 Hz, 1.9 Hz, 1H), 7.01-6.99 (m, 2H), 6.82 (s, 2H), 3.65-3.52 (m, 2H), 2.36 (dd, *J* = 12.5 Hz, 3.5 Hz, 1H), 2.09-2.01 (m, 1H), 1.99-1.89 (m, 1H), 1.32 (brs, 1H), -0.02 (s, 9H).

^{13}C NMR (100 MHz, $CDCl_3$): δ = 137.7, 135.1, 129.9, 128.9, 127.8, 126.5, 125.7, 124.7, 122.1, 119.1, 116.5, 110.7, 62.6, 50.2, 33.3, 21.6, -2.6.

HR-MS (ESI, m/z): calcd for $C_{21}H_{27}ONClSi$ $(M+H)^+$: 372.1545. Found: 372.1549.

Transformation from 35b into 35a:[13] **35b** (60 mg, 0.16 mmol) was dissolved in the mixture of DMSO:H_2O (2 mL, 19:1, v/v). Then 10 mol% of KO*t*Bu (1.8 mg) and 10 mol% 18-crown-6 (4.2 mg) were added, and the resulted mixture was heated at 80 ^oC for 5 h. After cooling to r.t., diethyl ether (20 mL) and water (20 mL) were added. The organic phase was separated and the aqueous layer was extracted with diethyl ether (3 x 20 mL), and the combined organic layers were dried over anhydrous Na_2SO_4, and concentrated under reduced pressure. The residue was purified by flash chromatography (eluted with CH_2Cl_2) to afford analytically pure product (40.3 mg, 83%).

Data of obtained products

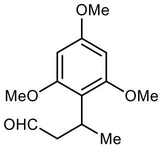

1
$C_{13}H_{18}O_4$
Mol. Wt.: 238.2796

^{1}H NMR (400 MHz, $CDCl_3$): δ = 9.63 (t, *J* = 2.5 Hz, 1H), 6.11 (s, 2H), 3.99-3.90 (m, 1H), 3.79 (s, 3H), 3.78 (s, 6H), 2.81 (ddd, *J* = 16.2 Hz, 7.8 Hz, 2.4 Hz, 1H), 2.72 (ddd, *J* = 16.3 Hz, 7.4 Hz, 2.7 Hz,1H), 1.28 (d, *J* = 7.1 Hz, 3H).

^{13}C NMR (100 MHz, $CDCl_3$): δ = 204.4, 159.8, 159.2, 113.4, 91.2, 55.7, 55.3, 49.2, 24.1, 19.4.

HR-MS (ESI, m/z): calcd for $C_{13}H_{19}O_4$ $(M+H)^+$: 239.1283. Found: 239.1279.

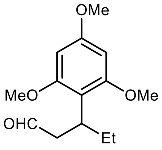

2
$C_{14}H_{20}O_4$
Mol. Wt.: 252.3062

^{1}H NMR (400 MHz, $CDCl_3$): δ = 9.59 (t, *J* = 2.6 Hz, 1H), 6.11 (s, 2H), 3.79 (s, 3H), 3.77 (s, 6H), 3.79-3.69 (m, 1H), 2.82 (ddd, *J* = 16.1 Hz, 8.6 Hz, 2.6 Hz, 1H), 2.69 (ddd, *J* = 16.1 Hz, 6.6 Hz, 2.7 Hz, 1H), 1.86-1.75 (m, 1H), 1.69-1.59 (m, 1H), 0.77 (t, *J* = 7.4 Hz, 3H).

^{13}C NMR (100 MHz, $CDCl_3$): δ = 204.4, 159.8, 159.5, 111.6, 91.1, 55.7, 55.3, 47.9, 31.3, 26.6, 12.5.

HR-MS (ESI, m/z): calcd for $C_{14}H_{21}O_4$ $(M+H)^+$: 253.1440. Found: 253.1433.

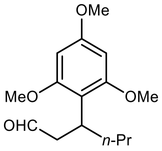

3
$C_{15}H_{22}O_4$
Mol. Wt.: 266.3328

^{1}H NMR (400 MHz, $CDCl_3$): δ = 9.59 (t, *J* = 2.7 Hz, 1H), 6.11 (s, 2H), 3.78 (s, 3H), 3.77 (s, 6H), 3.87-3.75 (m, 1H), 2.81 (ddd, *J* = 16.0 Hz, 8.6 Hz, 2.7 Hz, 1H), 2.67 (ddd, *J* = 16.0 Hz, 6.6 Hz, 2.7 Hz, 1H), 1.86-1.77 (m, 1H), 1.59-1.50 (m, 1H), 1.26-1.05 (m, 2H), 0.84 (t, *J* = 7.3 Hz, 3H).

^{13}C NMR (100 MHz, $CDCl_3$): δ = 204.4, 159.8, 159.5, 111.8, 91.1, 55.7, 55.3, 48.2, 36.0, 29.2, 21.1, 14.1.

HR-MS (ESI, m/z): calcd for $C_{15}H_{23}O_4$ $(M+H)^+$: 267.1596. Found: 267.1593.

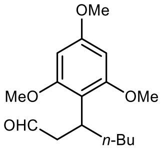

4
$C_{16}H_{24}O_4$
Mol. Wt.: 280.3594

^{1}H NMR (400 MHz, $CDCl_3$): δ = 9.59 (t, *J* = 2.7 Hz, 1H), 6.11 (s, 2H), 3.79 (s, 3H), 3.77 (s, 6H), 3.84-3.76 (m, 1H), 2.81 (ddd, *J* = 16.0 Hz, 8.6 Hz, 2.6 Hz, 1H), 2.67 (ddd, *J* = 16.0 Hz, 6.6 Hz, 2.7 Hz, 1H), 1.87-1.77 (m, 1H), 1.62-1.53 (m, 1H), 1.35-1.01 (m, 4H), 0.82 (t, *J* = 7.2 Hz, 3H).

^{13}C NMR (100 MHz, $CDCl_3$): δ = 204.4, 159.8, 159.5, 111.9, 91.1, 55.7, 55.3, 48.2, 33.4, 30.2, 29.5, 22.7, 14.1.

HR-MS (ESI, m/z): calcd for $C_{17}H_{27}O_4$ $(M+H)^+$: 281.1753. Found: 281.1750.

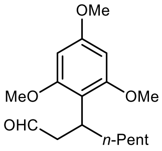

5
$C_{17}H_{26}O_4$
Mol. Wt.: 294.3859

^{1}H NMR (400 MHz, $CDCl_3$): δ = 9.59 (t, *J* = 2.7 Hz, 1H), 6.11 (s, 2H), 3.78 (s, 3H), 3.77 (s, 6H), 3.84-3.75 (m, 1H), 2.81 (ddd, *J* = 16.0 Hz, 8.6 Hz, 2.7 Hz, 1H), 2.67 (ddd, *J* = 16.0 Hz, 6.6 Hz, 2.7 Hz, 1H), 1.86-1.77 (m, 1H), 1.61-1.52 (m, 1H), 1.29-1.05 (m, 6H), 0.83 (t, *J* = 6.8 Hz, 3H).

^{13}C NMR (100 MHz, $CDCl_3$): δ = 204.4, 159.8, 159.5, 111.9, 91.1, 55.7, 55.3, 48.2, 33.6, 31.9, 29.5, 27.6, 22.7, 14.1.

HR-MS (ESI, m/z): calcd for $C_{17}H_{27}O_4$ $(M+H)^+$: 295.1909. Found: 295.1906.

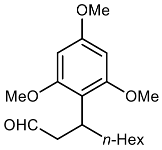

6
$C_{18}H_{28}O_4$
Mol. Wt.: 308.4125

^{1}H NMR (400 MHz, $CDCl_3$): δ = 9.59 (t, *J* = 2.7 Hz, 1H), 6.11 (s, 2H), 3.79 (s, 3H), 3.77 (s, 6H), 3.84-3.75 (m, 1H), 2.81 (ddd, *J* = 16.0 Hz, 8.6 Hz, 2.7 Hz, 1H), 2.67 (ddd, *J* = 16.0 Hz, 6.6 Hz, 2.7 Hz, 1H), 1.87-1.77 (m, 1H), 1.61-1.52 (m, 1H), 1.34-1.03 (m, 8H), 0.84 (t, *J* = 6.9 Hz, 3H).

^{13}C NMR (100 MHz, $CDCl_3$): δ = 204.4, 159.8, 159.5, 111.9, 91.1, 55.7, 55.3, 48.2, 33.6, 31.9, 29.5, 27.6, 22.7, 14.1.

HR-MS (ESI, m/z): calcd for $C_{18}H_{29}O_4$ $(M+H)^+$: 309.2066. Found: 309.2063.

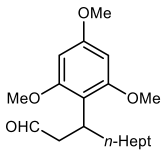

7
$C_{19}H_{30}O_4$
Mol. Wt.: 322.4391

^{1}H NMR (400 MHz, $CDCl_3$): δ = 9.59 (t, *J* = 2.7 Hz, 1H), 6.11 (s, 2H), 3.79 (s, 3H), 3.77 (s, 6H), 3.84-3.73 (m, 1H), 2.81 (ddd, *J* = 16.0 Hz, 8.6 Hz, 2.7 Hz, 1H), 2.67 (ddd, *J* = 16.0 Hz, 6.6 Hz, 2.7 Hz, 1H), 1.87-1.77 (m, 1H), 1.61-1.52 (m, 1H), 1.32-1.05 (m, 10H), 0.85 (t, *J* = 7.0 Hz, 3H).

^{13}C NMR (100 MHz, $CDCl_3$): δ = 204.1, 159.8, 159.5, 111.9, 91.1, 55.7, 55.3, 48.2, 33.7, 32.0, 29.7, 29.5, 29.3, 28.0, 22.8, 14.2.

HR-MS (ESI, m/z): calcd for $C_{19}H_{31}O_4$ $(M+H)^+$: 323.2222. Found: 323.2215.

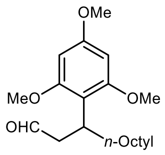

8
$C_{20}H_{32}O_4$
Mol. Wt.: 336.4720

^{1}H NMR (500 MHz, $CDCl_3$): δ = 9.59 (t, *J* = 2.7 Hz, 1H), 6.10 (s, 2H), 3.79 (s, 3H), 3.77 (s, 6H), 3.83-3.75 (m, 1H), 2.81 (ddd, *J* = 16.0 Hz, 8.6 Hz, 2.7 Hz, 1H), 2.67 (ddd, *J* = 16.1 Hz, 6.5 Hz, 2.7 Hz, 1H), 1.85-1.78 (m, 1H), 1.59-1.51 (m, 2H), 1.28-1.13 (m, 11H), 0.86 (t, *J* = 7.1 Hz, 3H).

^{13}C NMR (126 MHz, $CDCl_3$): δ = 204.7, 159.8, 159.5, 111.8, 91.0, 55.7, 55.4, 48.2, 33.7, 32.0, 29.7(2), 29.6(7), 29.5, 28.0, 22.8, 14.3.

HR-MS (ESI, m/z): calcd for $C_{20}H_{33}O_4$ $(M+H)^+$: 337.2373. Found: 337.2374.

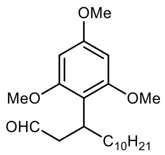

9
$C_{22}H_{36}O_4$
Mol. Wt.: 364.5260

^{1}H NMR (500 MHz, $CDCl_3$): δ = 9.59 (t, *J* = 2.7 Hz, 1H), 6.11 (s, 2H), 3.79 (s, 3H), 3.77 (s, 6H), 3.83-3.75 (m, 1H), 2.81 (ddd, *J* = 16.1 Hz, 8.7 Hz, 2.7 Hz, 1H), 2.67 (ddd, *J* = 16.0 Hz, 6.5 Hz, 2.7 Hz, 1H), 1.85-1.78 (m, 1H), 1.59-1.51 (m, 2H), 1.30-1.14 (m, 15H), 0.87 (t, *J* = 7.1 Hz, 3H).

^{13}C NMR (126 MHz, $CDCl_3$): δ = 204.7, 159.7, 159.5, 111.8, 91.0, 55.7, 55.4, 48.2, 33.7, 32.1, 29.8(1), 29.7(6), 29.7, 29.4(8), 29.4(7), 28.0, 22.8, 14.3.

HR-MS (ESI, m/z): calcd for $C_{22}H_{37}O_4$ $(M+H)^+$: 365.2686. Found: 365.2688.

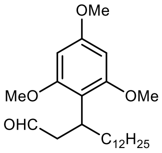

10
$C_{24}H_{40}O_4$
Mol. Wt.: 392.5800

^{1}H NMR (400 MHz, $CDCl_3$): δ = 9.59 (t, *J* = 2.7 Hz, 1H), 6.11 (s, 2H), 3.79 (s, 3H), 3.77 (s, 6H), 3.84-3.76 (m, 1H), 2.81 (ddd, *J* = 16.0 Hz, 8.6 Hz, 2.7 Hz, 1H), 2.67 (ddd, *J* = 16.0 Hz, 6.6 Hz, 2.7 Hz, 1H), 1.86-1.77 (m, 1H), 1.61-1.51 (m, 1H), 1.31-1.15 (m, 20H), 0.88 (t, *J* = 6.9 Hz, 3H).

^{13}C NMR (100 MHz, $CDCl_3$): δ = 204.5, 159.8, 159.5, 111.9, 91.1, 55.7, 55.3, 48.2, 33.7, 32.1, 29.8(2), 29.8(0), 29.7(8), 29.7, 29.5, 29.4(8), 28.0, 22.8, 14.2.

HR-MS (ESI, m/z): calcd for $C_{24}H_{41}O_4$ $(M+H)^+$: 393.3005. Found: 393.3006.

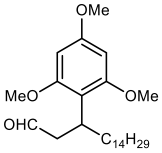

11
$C_{26}H_{44}O_4$
Mol. Wt.: 420.6340

^{1}H NMR (500 MHz, $CDCl_3$): δ = 9.59 (t, *J* = 2.7 Hz, 1H), 6.10 (s, 2H), 3.79 (s, 3H), 3.77 (s, 6H), 3.83-3.75 (m, 1H), 2.81 (ddd, *J* = 16.1 Hz, 8.7 Hz, 2.7 Hz, 1H), 2.67 (ddd, *J* = 16.0 Hz, 6.5 Hz, 2.7 Hz, 1H), 1.85-1.78 (m, 1H), 1.59-1.51 (m, 2H), 1.31-1.14 (m, 23H), 0.88 (t, *J* = 7.0 Hz, 3H).

^{13}C NMR (126 MHz, $CDCl_3$): δ = 204.7, 159.8, 159.5, 111.8, 91.0, 55.7, 55.4, 48.2, 33.7, 32.1, 29.9, 29.8(2), 29.8(0), 29.7, 29.5(1), 29.4(8), 28.0, 22.8, 14.3.

HR-MS (ESI, m/z): calcd for $C_{26}H_{45}O_4$ $(M+H)^+$: 421.3312. Found: 421.3312.

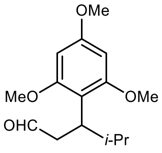

12
$C_{15}H_{22}O_4$
Mol. Wt.: 266.3328

^{1}H NMR (400 MHz, $CDCl_3$): δ = 9.49 (dd, *J* = 3.3 Hz, 2.4 Hz, 1H), 6.10 (s, 2H), 3.78 (s, 3H), 3.76 (s, 6H), 3.42 (td, *J* = 10.0 Hz, 5.3 Hz, 1H), 2.86 (ddd, *J* = 16.0 Hz, 10.1 Hz, 3.3 Hz, 1H), 2.68 (ddd,

J = 15.8 Hz, 5.3 Hz, 2.4 Hz, 1H), 2.22-2.01 (m, 1H), 0.98 (d, *J* = 6.6 Hz, 3H), 0.67 (d, *J* = 6.7 Hz, 3H).

^{13}C NMR (100 MHz, $CDCl_3$): δ = 204.9, 159.8, 159.3, 111.8, 91.0, 55.6, 55.3, 46.1, 37.1, 30.7, 21.7, 21.4.

HR-MS (ESI, m/z): calcd for $C_{15}H_{23}O_4$ $(M+H)^+$: 267.1596. Found: 267.1588.

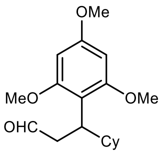

13
$C_{18}H_{26}O_4$
Mol. Wt.: 306.3966

^{1}H NMR (400 MHz, $CDCl_3$): δ = 9.50 (dd, *J* = 3.3 Hz, 2.3 Hz, 1H), 6.09 (s, 2H), 3.78 (s, 3H), 3.76 (s, 6H), 3.50 (td, *J* = 9.8 Hz, 5.5 Hz, 1H), 2.81 (ddd, *J* = 15.9 Hz, 9.8 Hz, 3.3 Hz, 1H), 2.70 (ddd, *J* = 15.8 Hz, 5.5 Hz, 2.4 Hz, 1H), 1.91-1.71 (m, 3H), 1.63-1.55 (m, 2H), 1.35-1.29 (m, 1H), 1.28-1.02 (m, 3H), 0.98-0.88 (m, 1H), 0.82-0.72 (m, 1H).

^{13}C NMR (100 MHz, $CDCl_3$): δ = 204.9, 159.8, 159.4, 111.5, 91.1, 55.7, 55.3, 45.7, 10.0, 35.5, 32.1, 31.4, 26.7, 26.6, 26.5.

HR-MS (ESI, m/z): calcd for $C_{18}H_{27}O_4$ $(M+H)^+$: 307.1909. Found: 307.1904.

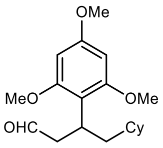

14
$C_{19}H_{28}O_4$
Mol. Wt.: 320.4232

^{1}H NMR (400 MHz, $CDCl_3$): δ = 9.57 (t, *J* = 2.7 Hz, 1H), 6.10 (s, 2H), 3.96-3.89 (m, 1H), 3.79 (s, 3H), 3.77 (s, 6H), 2.80 (ddd, *J* = 15.9 Hz, 8.8 Hz, 2.7 Hz, 1H), 2.61 (ddd, *J* = 15.9 Hz, 6.5 Hz, 2.7 Hz, 1H), 1.87-1.82 (m, 1H), 1.82-1.75 (m, 1H), 1.66-1.52 (m, 4H), 1.41-1.34 (m, 1H), 1.15-1.05 (m, 3H), 1.01-0.94 (m, 1H), 0.88-0.82 (m, 2H).

^{13}C NMR (100 MHz, $CDCl_3$): δ = 204.5, 159.7, 159.4, 112.0, 91.9, 55.6, 55.3, 48.3, 41.6, 35.8, 34.1, 33.2, 26.8, 26.5, 26.4.

HR-MS (ESI, m/z): calcd for $C_{19}H_{29}O_4$ $(M+H)^+$: 321.2060. Found: 321.2051.

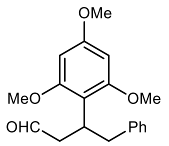

15
$C_{19}H_{22}O_4$
Mol. Wt.: 314.3756

^{1}H NMR (400 MHz, $CDCl_3$): δ = 9.52 (dd, *J* = 2.7 Hz, 2.4 Hz, 1H), 7.22-7.17 (m ,2H), 7.14-7.08 (m, 3H), 6.07 (s, 2H), 4.15-4.07 (m, 1H), 3.78 (s, 3H), 3.72 (s, 6H), 3.04-2.94 (m, 2H), 2.92 (ddd, *J* = 16.2 Hz, 9.0 Hz, 2.8 Hz, 1H), 2.68 (ddd, *J* = 16.2 Hz, 6.2 Hz, 2.3 Hz, 1H).

^{13}C NMR (100 MHz, $CDCl_3$): δ = 203.9, 160.0, 159.4, 141.1, 129.3, 128.1, 125.9, 111.4, 91.2, 55.8, 55.3, 47.1, 40.0, 31.8.

HR-MS (ESI, m/z): calcd for $C_{19}H_{23}O_4$ $(M+H)^+$: 315.1591. Found: 315.1591.

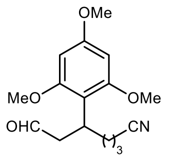

16
$C_{16}H_{21}NO_4$
Mol. Wt.: 291.3422

^{1}H NMR (400 MHz, $CDCl_3$): δ = 9.58 (t, *J* = 2.3 Hz, 1H), 6.10 (s, 2H), 3.88-3.75 (m, 1H), 3.78 (s, 3H), 3.77 (s, 6H), 2.83 (ddd, *J* = 16.5 Hz, 8.0 Hz, 2.4 Hz, 1H), 2.72 (ddd, *J* = 16.5 Hz, 6.9 Hz, 2.3 Hz, 1H), 2.28-2.23 (m, 2H), 2.08-1.99 (m, 1H), 1.69-1.62 (m, 1H), 1.51-1.42 (m, 2H).

^{13}C NMR (100 MHz, $CDCl_3$): δ = 203.2, 160.1, 159.4, 119.9, 109.9, 91.0, 55.6, 55.3, 48.1, 32.1, 28.2, 23.7, 16.8.

HR-MS (ESI, m/z): calcd for $C_{16}H_{22}NO_4$ $(M+H)^+$: 292.1543. Found: 292.1542.

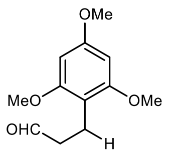

17
$C_{12}H_{16}O_4$
Mol. Wt.: 224.2530

^{1}H NMR (500 MHz, $CDCl_3$): δ = 9.74 (t, *J* = 2.3 Hz, 1H), 6.12 (s, 2H), 3.80 (s, 3H), 3.78 (s, 6H), 2.91 (t, *J* = 7.4 Hz, 2H), 2.53 (dt, *J* = 7.4 Hz, 2.4 Hz, 2H).

^{13}C NMR (126 MHz, $CDCl_3$): δ = 204.1, 159.9, 158.8, 108.9, 90.6, 55.6, 55.4, 43.7, 16.0.

HR-MS (ESI, m/z): calcd for $C_{12}H_{17}O_4$ $(M+H)^+$: 225.1121. Found: 225.1116.

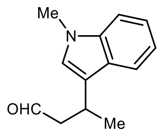

18
$C_{13}H_{15}NO$
Mol. Wt.: 201.2690

^{1}H NMR (400 MHz, $CDCl_3$): δ = 9.77 (t, *J* = 2.3 Hz, 1H), 7.66 (dt, *J* = 7.9 Hz, 1.0 Hz, 1H), 7.32 (dt, *J* = 8.2 Hz, 1.0 Hz, 1H), 7.28-7.24 (m, 1H), 7.15 (ddd, *J* = 8.0 Hz, 6.9 Hz, 1.1 Hz, 1H), 6.85 (d, *J* = 0.6 Hz, 1H), 3.76 (s, 3H), 3.74-3.66 (m, 1H), 2.89 (ddd, *J* = 16.3 Hz, 6.8 Hz, 2.4 Hz, 1H), 2.72 (ddd, *J* = 16.3 Hz, 7.5 Hz, 2.3 Hz, 1H), 1.46 (d, *J* = 6.9 Hz, 3H).

^{13}C NMR (100 MHz, $CDCl_3$): δ = 202.8, 137.4, 126.7, 125.3, 121.9, 126.8, 125.3, 121.9, 119.2, 119.0, 118.9(6), 109.5, 51.2, 32.7, 26.0, 21.8.

HR-MS (ESI, m/z): calcd for $C_{13}H_{16}ON$ $(M+H)^+$: 202.1226. Found: 202.1227.

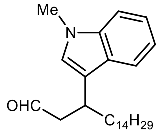

19
$C_{26}H_{41}NO$
Mol. Wt.: 383.6200

^{1}H NMR (400 MHz, $CDCl_3$): δ = 9.70 (t, *J* = 2.4 Hz, 1H), 7.65 (dt, *J* = 8.0 Hz, 1.0 Hz, 1H), 7.32 (dt, *J* = 8.2 Hz, 1.0 Hz, 1H), 7.25 (ddd, *J* = 8.2 Hz, 6.9 Hz, 1.2 Hz, 1H), 7.14 (ddd, *J* = 8.0 Hz, 6.9 Hz, 1.1 Hz, 1H), 6.85 (s, 1H), 3.75 (s, 3H), 3.56-3.48 (m, 1H), 2.84 (ddd, *J* = 16.1 Hz, 8.1 Hz, 2.5 Hz, 1H), 2.78 (ddd, *J* = 16.1 Hz, 6.6 Hz, 2.3 Hz, 1H), 1.89-1.81 (m, 1H), 1.79-1.71 (m, 1H), 1.36-1.26 (m, 24H), 0.92 (d, *J* = 6.9 Hz, 3H).

^{13}C NMR (100 MHz, $CDCl_3$): δ = 203.1, 137.4, 127.1, 126.1, 121.8, 119.3, 118.9, 117.3, 109.5, 49.7, 36.3, 32.7, 32.1, 31.7, 29.8(1), 29.8(0), 29.7(8), 29.7(5), 29.7, 29.5, 27.6, 22.8, 14.2.

HR-MS (ESI, m/z): calcd for $C_{26}H_{42}ON$ $(M+H)^+$: 384.3261. Found: 384.3265.

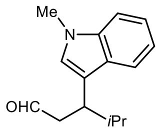

20
$C_{15}H_{19}NO$
Mol. Wt.: 229.3230

^{1}H NMR (400 MHz, $CDCl_3$): δ = 9.60 (t, *J* = 2.5 Hz, 1H), 7.64 (dt, *J* = 8.0 Hz, 1.0 Hz, 1H), 7.30 (dt, *J* = 8.2 Hz, 1.0 Hz, 1H), 7.23 (ddd, *J* = 8.2 Hz, 7.0 Hz, 1.2 Hz, 1H), 7.12 (ddd, *J* = 8.0 Hz, 6.9 Hz,

1.1 Hz, 1H), 6.82 (s, 1H), 3.75 (s, 3H), 3.41-3.35 (m, 1H), 2.81-2.78 (m, 2H), 2.11 (hept., *J* = 6.7 Hz, 1H), 0.94 (df, *J* = 6.8 Hz, 4.4 Hz, 6H).

^{13}C NMR (100 MHz, $CDCl_3$): δ = 203.5, 137.3, 127.7, 126.8, 121.7, 119.5, 118.9, 115.9, 109.4, 46.2, 38.1, 32.9, 32.8, 20.5, 20.3.

HR-MS (ESI, m/z): calcd for $C_{15}H_{20}ON$ $(M+H)^+$: 230.1545. Found: 230.1541.

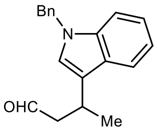

21

$C_{19}H_{19}NO$

Mol. Wt.: 277.3670

^{1}H NMR (500 MHz, $CDCl_3$): δ = 9.76 (t, *J* = 2.3 Hz, 1H), 7.65 (dt, *J* =7.9 Hz, 1.0 Hz, 1H), 7.32-7.24 (m, 4H), 7.18 (ddd, *J* = 8.2 Hz, 7.0 Hz, 1.2 Hz, 1H), 7.14-7.08 (m, 3H), 6.92 (s, 1H), 5.27 (s, 2H), 3.70 (sext., *J* = 6.9 Hz, 1H), 2.88 (ddd, *J* = 16.3 Hz, 6.7 Hz, 2.3 Hz, 1H), 2.72 (ddd, *J* = 16.3 Hz, 7.6 Hz, 2.3 Hz, 1H), 1.45 (d, *J* = 7.0 Hz, 3H).

^{13}C NMR (126 MHz, $CDCl_3$): δ = 202.9, 137.7, 137.1, 128.9, 127.7, 127.0, 126.9, 124.7, 122.1, 119.7, 119.3, 119.2, 110.0, 51.1, 50.1, 26.1, 21.7.

HR-MS (ESI, m/z): calcd for $C_{19}H_{20}ON$ $(M+H)^+$: 278.1539. Found: 278.1539.

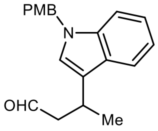

22

$C_{20}H_{21}NO_2$

Mol. Wt.: 307.3930

^{1}H NMR (500 MHz, $CDCl_3$): δ = 9.76 (t, *J* = 2.3 Hz, 1H), 7.64 (dt, *J* = 7.9 Hz, 0.9 Hz, 1H), 7.28 (dt, *J* = 8.2 Hz, 0.8 Hz, 1H), 7.19 (ddd, *J* = 8.2 Hz, 7.0 Hz, 1.2 Hz, 1H), 7.12 (ddd, *J* = 8.0 Hz, 7.0 Hz, 1.0 Hz, 1H), 7.06 (dt, *J* = 6.6 Hz, 2.2 Hz, 2H), 6.89 (s, 1H), 6.84 (dt, *J* = 7.9 Hz, 0.9 Hz, 2H), 5.20 (s, 2H), 3.78 (s, 3H), 3.72-3.65 (m, 1H), 2.87 (ddd, *J* = 16.3 Hz, 6.7 Hz, 2.3 Hz, 1H), 2.71 (ddd, *J* = 16.3 Hz, 7.6 Hz, 2.3 Hz, 1H), 1.44 (d, *J* = 7.0 Hz, 3H).

^{13}C NMR (126 MHz, $CDCl_3$): δ = 202.9, 159.2, 137.0, 129.6, 128.3, 127.0, 124.5, 122.0, 119.6, 119.3, 119.2, 114.3, 110.0, 55.4, 51.1, 49.6, 26.1, 21.7.

HR-MS (ESI, m/z): calcd for $C_{20}H_{22}O_2N$ $(M+H)^+$: 308.1642. Found: 308.1645.

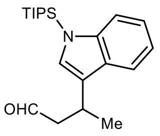

23
$C_{21}H_{33}NOSi$
Mol. Wt.: 343.5860

^{1}H NMR (500 MHz, $CDCl_3$): δ = 9.74 (t, *J* = 2.4 Hz, 1H), 7.62-7.61 (m, 1H), 7.50-7.48 (m, 1H), 7.17-7.11 (m, 2H), 6.99 (s, 1H), 3.67 (sext., *J* = 7.0 Hz, 1H), 2.88 (ddd, *J* = 16.2 Hz, 6.8 Hz, 2.5 Hz, 1H), 2.72 (ddd, *J* = 16.2 Hz, 7.5 Hz, 2.3 Hz, 1H), 3.67 (sext., *J* = 7.0 Hz, 1H), 1.68 (hept., *J* = 7.5 Hz, 3H), 1.45 (d, *J* = 7.0 Hz, 3H), 1.13 (d, *J* = 7.5 Hz, 18H)

^{13}C NMR (126 MHz, $CDCl_3$): δ = 203.0, 141.8, 129.9, 127.3, 122.0, 121.7, 119.5, 118.9, 114.3 50.9, 26.1, 21.5, 18.3, 13.0.

HR-MS (ESI, m/z): calcd for $C_{21}H_{34}ONSi$ $(M+H)^+$: 344.2404. Found: 344.2401.

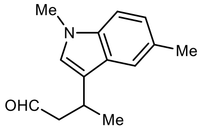

25
$C_{14}H_{17}NO$
Mol. Wt.: 215.2960

^{1}H NMR (400 MHz, $CDCl_3$): δ = 9.75 (t, *J* = 2.3 Hz, 1H), 7.40-7.39 (m, 1H), 7.18 (d, *J* = 8.3 Hz, 1H), 7.06 (ddt, *J* = 8.3 Hz, 1.6 Hz, 0.5 Hz, 1H), 6.79 (s, 1H), 3.71 (s, 3H), 3.67-3.60 (m, 1H), 2.86 (ddd, *J* = 16.3 Hz, 6.8 Hz, 2.4 Hz, 1H), 2.69 (ddd, *J* = 16.3 Hz, 7.5 Hz, 2.3 Hz, 1H), 2.47 (s, 3H), 1.43 (d, *J* = 7.0 Hz, 3H).

^{13}C NMR (100 MHz, $CDCl_3$): δ = 203.0, 135.9, 128.2, 127.0, 125.3, 123.5, 118.9, 118.5, 109.3, 51.2, 32.8, 26.1, 21.8, 21.6.

HR-MS (ESI, m/z): calcd for $C_{14}H_{18}ON$ $(M+H)^+$: 216.1383. Found: 216.1383.

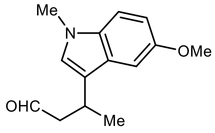

26
$C_{14}H_{17}NO_2$
Mol. Wt.: 231.2950

^{1}H NMR (500 MHz, $CDCl_3$): δ = 9.76 (t, *J* = 2.3 Hz, 1H), 7.19 (dd, *J* = 8.8 Hz, 0.5 Hz, 1H), 7.07 (dd, *J* = 2.4 Hz, 0.4 Hz, 1H), 6.91 (ddd, *J* = 6.0 Hz, 2.4 Hz, 0.4 Hz, 1H), 6.81 (s, 1H), 3.88 (s, 3H), 3.71 (s, 3H), 3.68-3.59 (m, 1H), 2.85 (ddd, *J* = 16.3 Hz, 6.8 Hz, 2.3 Hz, 1H), 2.70 (ddd, *J* = 16.3 Hz, 7.3 Hz, 2.3 Hz, 1H), 1.43 (d, *J* = 7.0 Hz, 3H).

^{13}C NMR (100 MHz, $CDCl_3$): δ = 202.9, 153.9, 132.8, 127.0, 125.9, 118.5, 111.9, 110.3, 101.4, 56.2, 51.1, 32.9, 25.9, 21.7.

HR-MS (ESI, m/z): calcd for $C_{14}H_{18}O_2N$ $(M+H)^+$: 232.1332. Found: 232.1329.

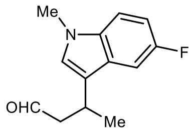

27
$C_{13}H_{14}FNO$
Mol. Wt.: 219.2594

^{1}H NMR (500 MHz, $CDCl_3$): δ = 9.74 (t, *J* = 2.3 Hz, 1H), 7.26 (dd, *J* = 9.7 Hz, 2.5 Hz, 1H), 7.19 (dd, *J* = 8.9 Hz, 4.3 Hz, 1H), 6.97 (td, *J* = 9.0 Hz, 2.5 Hz, 1H), 6.87 (s, 1H), 3.73 (s, 3H), 3.60 (sext., *J* = 7.0 Hz, 1H), 2.83 (ddd, *J* = 16.4 Hz, 7.0 Hz, 2.3 Hz, 1H), 2.69 (ddd, *J* = 16.4 Hz, 7.4 Hz, 2.2 Hz, 1H), 1.41 (d, *J* = 7.0 Hz, 3H).

^{13}C NMR (126 MHz, $CDCl_3$): δ = 202.6, 157.6 (d, *J* = 234.3 Hz), 134.0, 126.9, 126.8 (d, *J* = 9.7 Hz), 118.9, (d, *J* = 4.9 Hz), 110.3 (d, *J* = 15.7 Hz), 110.1, 104.1 (d, *J* = 23.7 Hz), 51.0, 33.1, 25.9, 21.7.

^{19}F NMR (470 MHz, $CDCl_3$): δ = 125.3 (m).

HR-MS (ESI, m/z): calcd for $C_{13}H_{15}ONF$ $(M+H)^+$: 220.1132. Found: 220.1133.

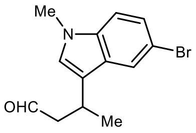

28
$C_{13}H_{14}BrNO$
Mol. Wt.: 280.1650

^{1}H NMR (500 MHz, $CDCl_3$): δ = 9.74 (t, *J* = 2.2 Hz, 1H), 7.73 (dd, *J* = 1.9 Hz, 0.4 Hz, 1H), 7.30 (dd, *J* = 8.7 Hz, 1.9 Hz, 1H), 7.15 (d, *J* = 8.7 Hz, 1H), 6.83 (s, 1H), 3.72 (s, 3H), 3.61 (sext., *J* = 6.9 Hz, 1H), 2.84 (ddd, *J* = 16.5 Hz, 6.9 Hz, 2.3 Hz, 1H), 2.69 (ddd, *J* = 16.5 Hz, 7.4 Hz, 2.1 Hz, 1H), 1.41 (d, *J* = 7.0 Hz, 3H).

^{13}C NMR (126 MHz, $CDCl_3$): δ = 202.5, 136.0, 128.4, 126.5, 124.7, 121.7, 118.7, 112.4, 111.0, 51.1, 33.0, 25.8, 21.8.

HR-MS (ESI, m/z): calcd for $C_{13}H_{18}ON_2Br$ $(M+H)^+$: 280.0332. Found: 280.0329.

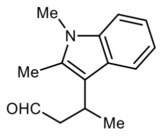

29
$C_{14}H_{17}NO$
Mol. Wt.: 215.2960

^{1}H NMR (500 MHz, $CDCl_3$): δ = 9.67 (t, *J* = 2.1 Hz, 1H), 7.63 (dt, *J* = 7.9 Hz, 0.9 Hz, 1H), 7.26 (dt, *J* = 8.2 Hz, 0.8 Hz, 1H), 7.16 (ddd, *J* = 8.2 Hz, 7.0 Hz, 1.1 Hz, 1H), 7.07 (ddd, *J* = 8.0 Hz, 7.0 Hz, 1.1 Hz, 1H), 3.68-3.61 (m, 1H), 3.64 (s, 3H), 3.01 (ddd, *J* = 16.5 Hz, 8.2 Hz, 1.9 Hz, 1H), 2.85 (ddd, *J* = 16.5 Hz, 6.8 Hz, 2.2 Hz, 1H), 2.4 (s, 3H), 1.48 (d *J* = 7.2 Hz, 3H).

^{13}C NMR (126 MHz, $CDCl_3$): δ = 202.9, 137.1, 132.3, 126.0, 120.6, 119.0, 118.8, 114.0, 109.0, 50.8, 29.6, 26.5, 21.6, 10.6.

HR-MS (ESI, m/z): calcd for $C_{14}H_{18}ON$ $(M+H)^+$: 216.1383. Found: 216.1384.

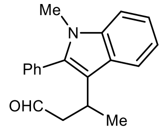

30
$C_{19}H_{19}NO$
Mol. Wt.: 277.3670

^{1}H NMR (400 MHz, $CDCl_3$): δ = 9.60 (t, *J* = 2.4 Hz, 1H), 7.78 (ddd, *J* = 7.9 Hz, 1.1 Hz, 0.9 Hz, 1H), 7.57-7.48 (m, 3H), 7.41-7.38 (m, 3H), 7.30 (ddd, *J* = 8.2 Hz, 7.0 Hz, 1.2 Hz, 1H), 7.19 (ddd, *J* = 8.0 Hz, 7.0 Hz, 1.1 Hz, 1H), 3.55 (s, 3H), 3.61-3.52 (m, 1H), 2.95 (ddd, *J* = 16.1 Hz, 7.9 Hz, 2.1 Hz, 1H), 2.78 (ddd, *J* = 16.1 Hz, 7.3 Hz, 2.6 Hz, 1H), 1.50 (d, *J* = 7.1 Hz, 3H).

^{13}C NMR (100 MHz, $CDCl_3$): δ = 202.8, 137.5(3), 137.4(9), 132.2, 130.9, 128.6, 128.5, 125.9, 121.8, 119.9, 119.3, 115.9, 109.8, 50.5, 30.7, 26.8, 22.0.

HR-MS (ESI, m/z): calcd for $C_{19}H_{20}ON$ $(M+H)^+$: 278.1539. Found: 278.1541.

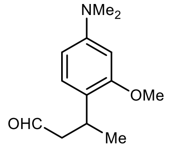

31
$C_{13}H_{19}NO_2$
Mol. Wt.: 221.3000

^{1}H NMR (400 MHz, $CDCl_3$): δ = 9.69 (t, *J* = 2.5 Hz, 1H), 7.03 (d, *J* = 8.4 Hz, 1H), 6.32 (dd, *J* = 8.4 Hz, 2.5 Hz, 1H), 6.28 (d, *J* = 2.5 Hz, 1H), 3.84 (s, 3H), 3.63 (sext., *J* = 7.1 Hz, 1H), 2.95 (s, 6H),

2.69 (ddd, J = 5.9 Hz, 6.8 Hz, 2.3 Hz, 1H), 2.57 (ddd, J = 15.9 Hz, 7.7 Hz, 2.7 Hz, 1H), 1.29 (d, J = 7.0 Hz, 3H).

^{13}C NMR (100 MHz, $CDCl_3$): δ = 203.3, 157.6, 150.7, 127.4, 121.7, 105.0, 96.5, 55.2, 51.1, 40.9, 27.5, 20.7.

HR-MS (ESI, m/z): calcd for $C_{13}H_{20}O_2N$ $(M+H)^+$: 222.1489. Found: 222.1492.

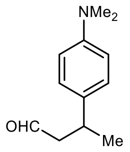

32
$C_{12}H_{17}NO$
Mol. Wt.: 191.2740

^{1}H NMR (500 MHz, $CDCl_3$): δ = 9.70 (t, J = 2.2 Hz, 1H), 7.11 (d, J = 8.5 Hz, 1H), 6.72 (brs, 2H), 3.28 (sext., J = 7.1 Hz, 1H), 2.93 (s, 6H), 2.70 (ddd, J = 16.3 Hz, 7.0 Hz, 2.0 Hz, 1H), 2.61 (ddd, J = 16.4 Hz, 7.6 Hz, 2.5 Hz, 1H), 1.29 (d, J = 6.9 Hz, 3H).

^{13}C NMR (126 MHz, $CDCl_3$): δ = 202.6, 149.5, 133.5, 127.6, 113.3, 52.1, 41.0, 33.7, 22.5.

HR-MS (ESI, m/z): calcd for $C_{12}H_{18}ON$ $(M+H)^+$: 192.1383. Found: 192.1385.

Supplementary Figures

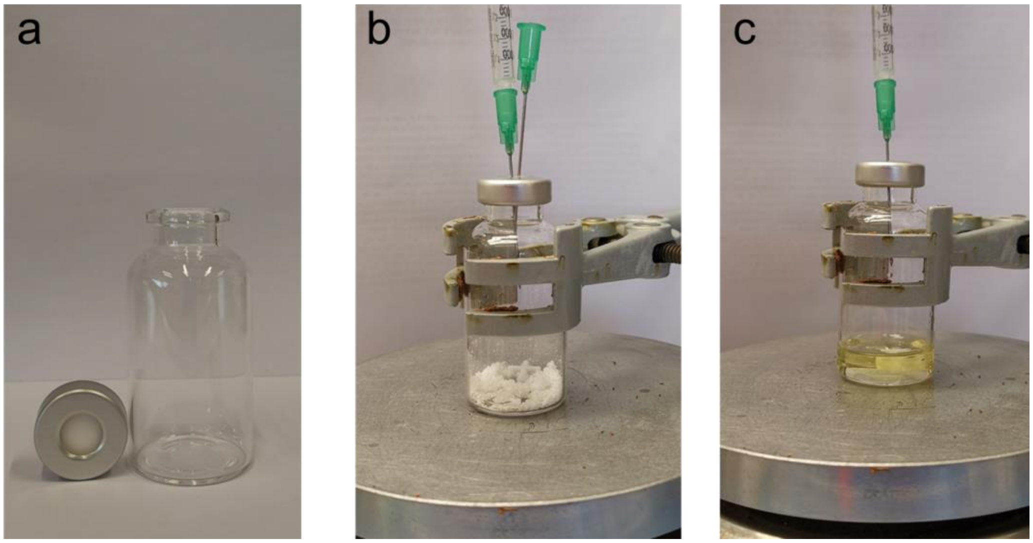

Supplementary Figure 2 Glass liner used in the catalytic reaction. **a** Glass liner and an aluminum crimp cap. **b** Glass liner under argon. **c** Glass liner after adding all the substrates

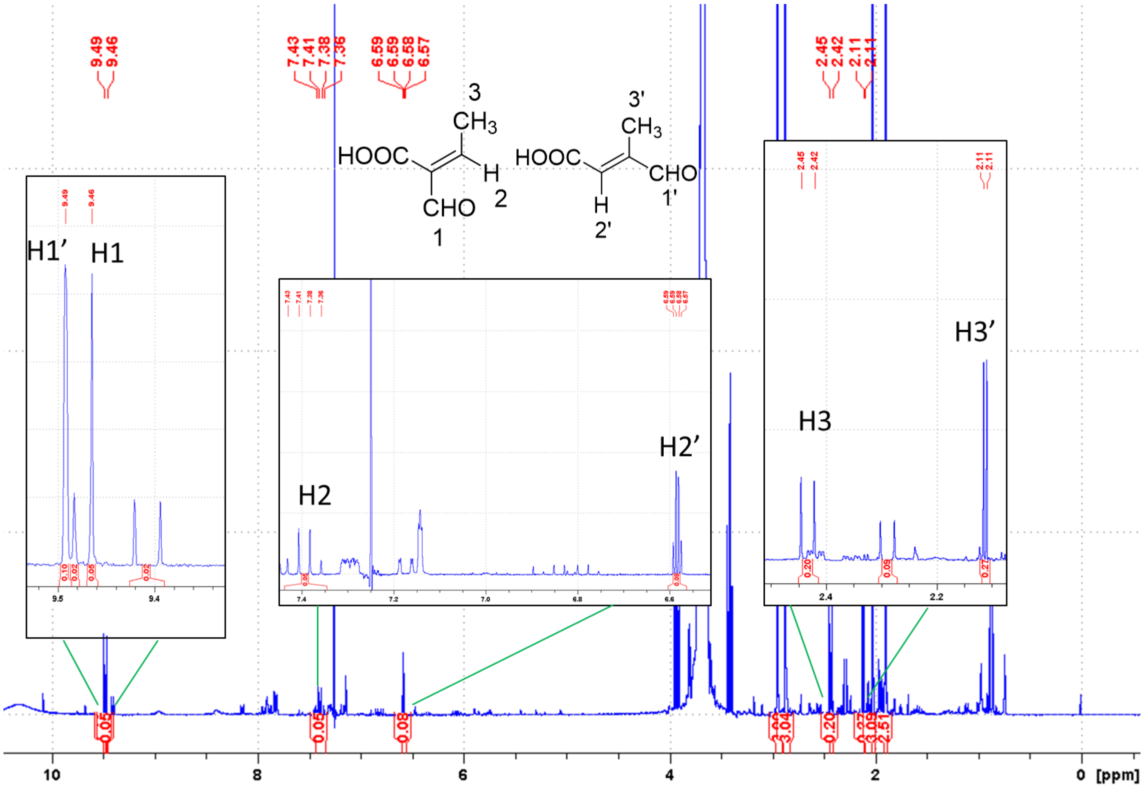

Supplementary Figure 3 ^{1}H NMR ($CDCl_3$, 300 MHz) spectrum: crude results for controlled catalysis of **S1** without TMB (without evaporation of DCE after reaction ended)

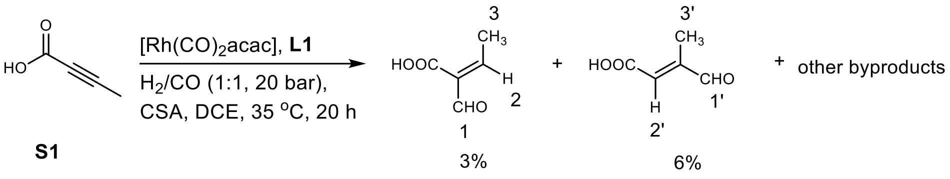

Supplementary Figure 4 Controlled catalysis of **S1** without TMB

Supplementary Tables

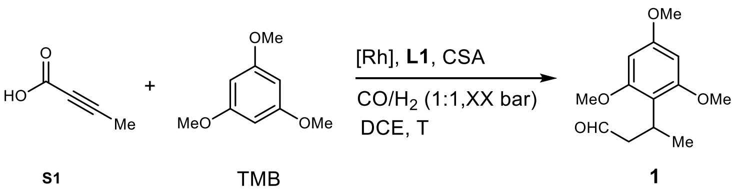

Supplementary Figure 5 Supramolecular domino reaction of 2-butynoic acid (**S1**) and TMB

Supplementary Table 1 Effect of pressure[a]

Entry	P (bar)	Yield (**1**) (%)	Conv. (**S1**) (%)	Conv. (TMB) (%)
1[b]	6	35	100	36
2	6	53	100	57
3	10	58	100	64
4	16	60	100	67
5	20	65	100	68
6	26	64	100	68
7	30	65	100	68

[a]Reaction condition: 1 mmol scale of TMB, [Rh(CO)$_2$acac]/**L1**/CSA/**S1**/TMB = 1:5:5:100:100, c(**S1**) = 0.5 M, DCE (2 mL), CO/H_2 (1:1, XX bar), 55 ^{o}C, 20 h. Yield and conversion were determined by NMR spectroscopy using DMAc as the internal standard. [b]Without CSA. CSA = camphorsulfonic acid. DMAc = dimethylacetamide

Supplementary Table 2 Effect of solvents[a]

Entry	Solvent	Yield (**1**) (%)	Conv. (**S1**) (%)	Conv. (TMB) (%)
1	DCE	65	100	68
2[b]	DCE	72	100	74
3	Toluene	52	98	59
4	THF	3	46	7
5[b]	CH_2Cl_2	71	100	73

[a]Reaction condition: 1 mmol scale of TMB, [Rh(CO)$_2$acac]/**L1**/CSA/**S1**/TMB = 1:5:5:100:100, c(**S1**) = 0.5 M, Solvent (2 mL), CO/H_2 (1:1, 20 bar), 55 ^{o}C, 20 h. Yield and conversion were determined by NMR spectroscopy using DMAc as the internal standard. [b]35 ^{o}C

Supplementary Table 3 Effect of temperature[a]

Entry	T (^{o}C)	Yield (**1**) (%)	Conv. (**S1**) (%)	Conv. (TMB) (%)
1	55	65	100	68
2	65	59	100	61
3	45	68	100	72
4	40	71	100	72
5	35	72	100	74
6	30	66	100	71
7	25	53	100	63

[a]Reaction condition: 1 mmol scale of TMB, [Rh(CO)$_2$acac]/**L1**/CSA/**S1**/TMB = 1:5:5:100:100, c(**S1**) = 0.5 M, DCE (2 mL), CO/H_2 (1:1, 20 bar), XX ^{o}C, 20 h. Yield and conversion were determined by NMR spectroscopy using DMAc as the internal standard

Supplementary Table 4 Effect of the ratio of [Rh]/**L1**/CSA[a]

Entry	Ratio of [Rh]/**L1**/CSA	Yield (**1**) (%)	Conv. (**S1**) (%)	Conv. (TMB) (%)
1	1/5/5	72	100	74
2	1/3/3	65	100	71
3	1/6/6	74	100	75
4	1/7/7	74	100	74
5	1/10/10	73	100	73
6	1/6/3	64	100	65
7	1/6/9	37	100	70

[a]Reaction condition: 1 mmol scale of TMB, $[Rh(CO)_2acac]$/**L1**/CSA/**S1**/TMB = 1:XX:XX:100:100, c(**S1**) = 0.5 M, DCE (2 mL), CO/H_2 (1:1, 20 bar), 35 ^oC, 20 h. Yield and conversion were determined by NMR spectroscopy using DMAc as the internal standard

Supplementary Table 5 Effect of additive[a]

Entry	Additive	Yield (**1**) (%)	Conv. (**S1**) (%)	Conv. (TMB) (%)
1	CSA	74	100	75
2	TFA	73	100	77
3	TsOH	60	100	65
4	TfOH	29	59	49

[a]Reaction condition: 1 mmol scale of TMB, $[Rh(CO)_2acac]$/**L1**/XX/**S1**/TMB = 1:6:6:100:100, c(**S1**) = 0.5 M, DCE (2 mL), CO/H_2 (1:1, 20 bar), 35 ^oC, 20 h. Yield and conversion were determined by NMR spectroscopy using DMAc as the internal standard. TsOH = *p*-toluenesulfonic acid, TfOH = trifluoromethanesulfonic acid, TFA = trifluoroacetic acid

Supplementary Table 6 Effect of concentration, and ratio of **S1** and TMB[a]

Entry	Ratio of **S1**/TMB	Con. (**S1**)	Yield (**1**) (%)	Conv. (**S1**) (%)	Conv. (TMB) (%)
1	100/100	0.5 M	74	100	75
2	100/100	1.0 M	68	100	74
3	100/100	0.25 M	70	100	71
4	130/100	0.65 M	85	100	89
5	140/100	0.5 M	89	98	93
6	150/100	0.5 M	90(90)	95	95

[a]Reaction condition: 1 mmol scale of TMB, $[Rh(CO)_2acac]$/**L1**/CSA/**S1**/TMB = 1:6:6:XX:100, c(**S1**) = XX M, DCE, CO/H_2 (1:1, 20 bar), 35 ^oC, 20 h. Yield and conversion were determined by NMR spectroscopy using DMAc as the internal standard. Isolated yield was shown within parentheses

Supplementary Table 7 Effect of ligands[a]

Entry	Ligand	Yield (**1**) (%)	Conv. (**S1**) (%)	Conv. (TMB) (%)
1	**L1**	90(90)	95	95
2	**L2**	67	99	71
3	**L3**	66	81	73
4	**L4**	0	10	13
5	**L5**	0	6	9

[a]Reaction condition: 1 mmol scale of TMB, $[Rh(CO)_2acac]$/**L**/CSA/**S1**/TMB = 1:6:6:150:100, c(**S1**) = 0.5 M, DCE (3 mL), CO/H_2 (1:1, 20 bar), 35 °C, 20 h. Yield and conversion were determined by NMR spectroscopy using DMAc as the internal standard. Isolated yield was shown within parentheses

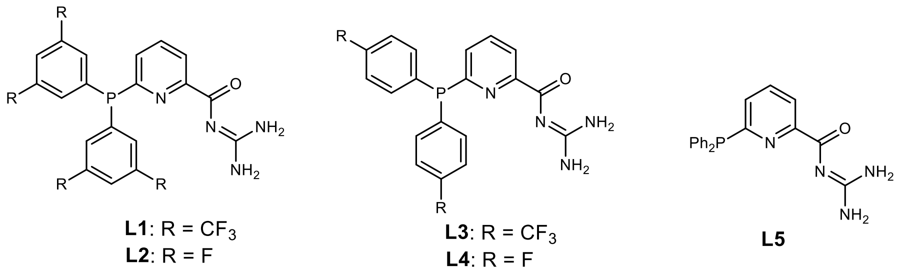

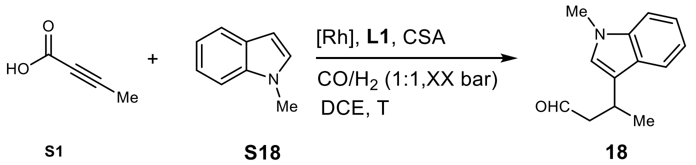

Supplementary Figure 6 Supramolecular domino reaction of **S1** and **S18**

Supplementary Table 8 Reaction optimization of 1-methylindole and 2-butynoic acid[a]

Entry	P (bar)	T (°C)	c(**S1**) (M)	Yield (**18**) (%)	Conv. (**S1**) (%)	Conv. (**S18**) (%)
1	20	35	0.5	54	95	85
2	10	35	0.5	36	70	80
3	30	35	0.5	61	100	83
4	40	35	0.5	57	95	82
5	30	35	0.375	65	97	87
6[b]	30	35	0.25	64	91	89
7[b]	30	40	0.25	74	96	88
8[b]	30	45	0.25	81	100	86
9[b]	30	50	0.25	81(81)	100	85

[a]Reaction condition: 1 mmol scale of 1-methylindole, $[Rh(CO)_2acac]$/**L1**/CSA/**S1**/**S18** = 1:6:6:150:100, DCE (XX mL), CO/H_2 (1:1, XX bar), XX °C, 20 h. Yield and conversion were

determined by NMR spectroscopy using DMAc as the internal standard. Isolated yield was shown within parentheses. [b]0.5 mmol scale of 1-methylindole

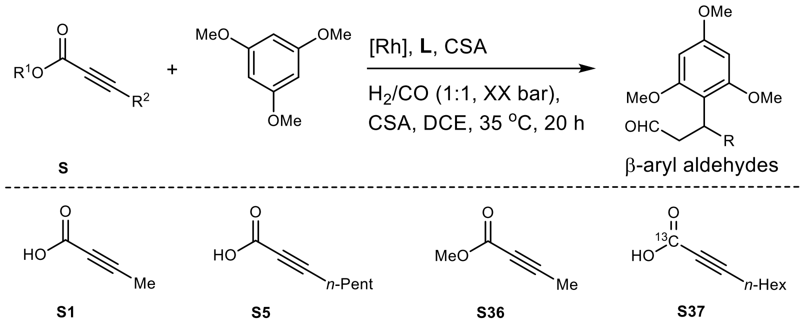

Supplementary Figure 7 Control experiments

Supplementary Table 9 Control experiments[a]

Entry	Ligand	Substrate (**S**)	Yield of (β-aryl aldehydes) (%)	Conv. (**S**) (%)	Conv. (TMB) (%)
1	**L1**	**S1**	90(90)	95	95
2	**L6**	**S1**	16	36	36
3	**L7/L8**	**S1**	0	7	9
4	**L9**	**S1**	0	2	2
5[b]	**L1**	**S1**	<4	15	18
6	**L1**	**S36**	0	60	1
7[c]	**L1**	**S1**	-	6	2
8[d]	**L1**	**S1**	-	42	-
9	**L1**	**S5**	82(81)	90	89
10[d]	**L1**	**S5**	-	95	-
11[e]	**L1**	**S37**	78	81	89

[a]Reaction condition: 1 mmol scale of TMB, $[Rh(CO)_2acac]$/**L**/CSA/**S1**/TMB = 1:6:6:150:100, c(**S1**) = 0.5 M, DCE (3 mL), CO/H_2 (1:1, 20 bar), 35 °C, 20 h. Yield and conversion were determined by NMR spectroscopy using DMAc as the internal standard. Isolated yield was shown within parentheses. [b]MeOH was used instead of DCE. [c]Argon was used instead of syngas. [d]Without TMB. [e]$(Rh(CO)_2acac)$/**L1**/CSA/**S37**/TMB = 1:6:6:200:100

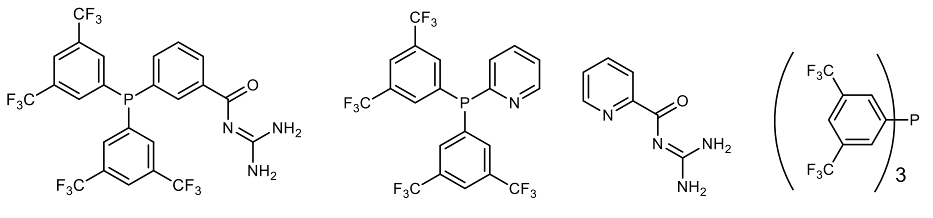

L6 **L7** **L8** **L9**

Supplementary Discussion

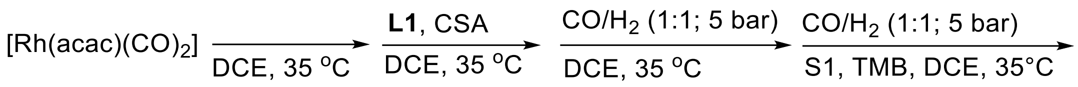

Supplementary Figure 8 General procedure for catalytic reaction performed in high pressure in-situ-IR experiments

High pressure in-situ-IR. Procedure: In a in-situ-IR experiment, the autoclave equipped with a glass inlet and magnetic stirrer was connected to a Sicomb (9 mm) probe of an in-situ-IR TM 45 (Mettler Toledo) and a reservoir. The autoclave was evacuated for 60 min and purged with argon. DCE (4 mL) was added and the autoclave heated to 35°C. A spectra was recorded every 60 seconds. After 20 min the solvent spectrum was subtracted. Then [Rh(acac)(CO)$_2$] (13.0 mg, 50.0 µmol) in DCE (1 mL) was added to the reaction mixture. After stirring for 30 min, a solution of **L1** (188 mg, 300 µmol, 6 eq.) and CSA (70.2 mg, 300 µmol, 6 eq.) in DCE (3 mL) was added to the reaction mixture. The autoclave was purged twice with 1 bar of synthesis gas (CO/H$_2$, 1:1) and was pressurized to 5 bar. After 1 h the pressure was released and a solution of S1 (190 mg, 2.27 mmol, 45 eq.) and TMB (254 mg, 1.51 mmol, 30 eq.) in DCE (2 mL) was added. Then the autoclave was repressurized with CO/H$_2$ (1:1, 5 bar) and the reaction stirred for 30 h at 35°C.

Conclusion: As shown in Fig S3, we could easily follow the formation of the product **1** and consumption of substrate **S1**. Furthermore, a change of IR bands between 2150 and 1950 cm^{-1} was observed, however, detailed characterization of each IR band to related structure was very difficult to continue.

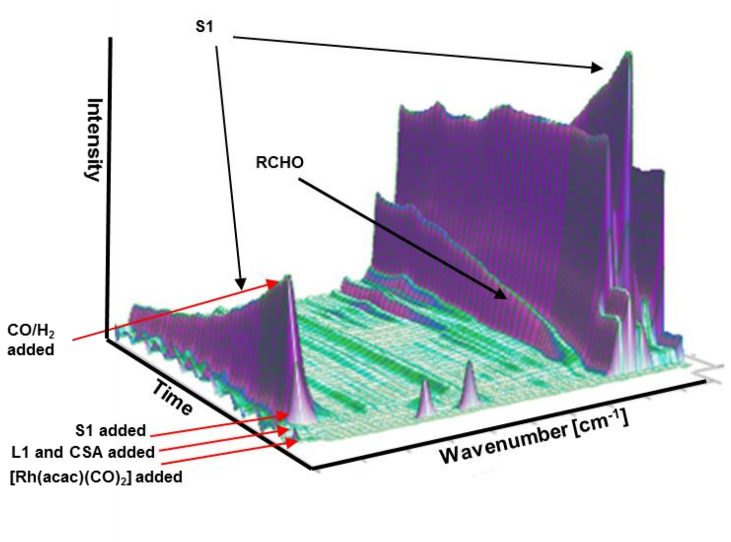

Supplementary Figure 9 3D-in-situ-IR spectrum

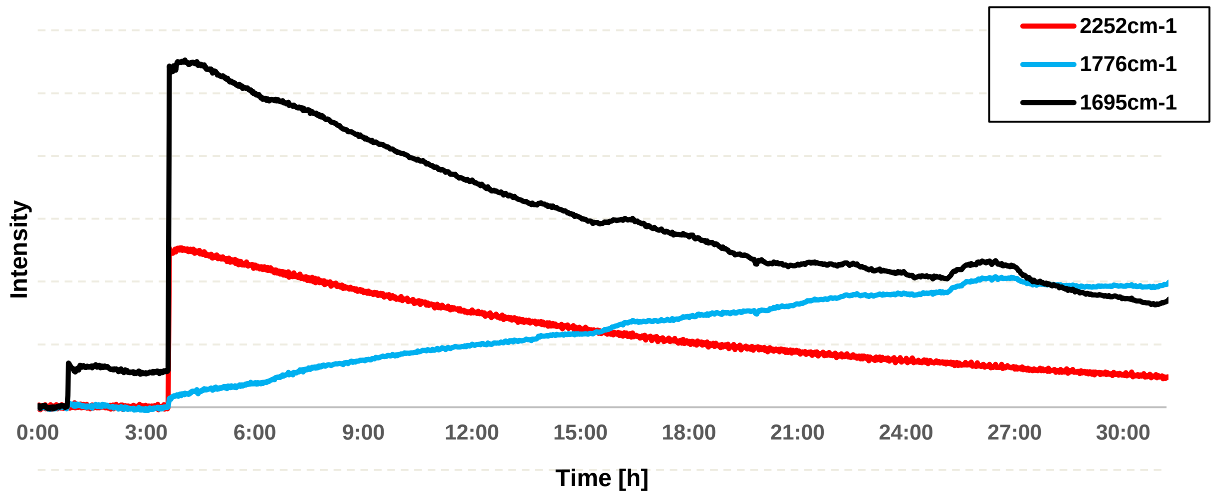

Supplementary Figure 10 Develoment of Intensity of Peaks related to S1 and product

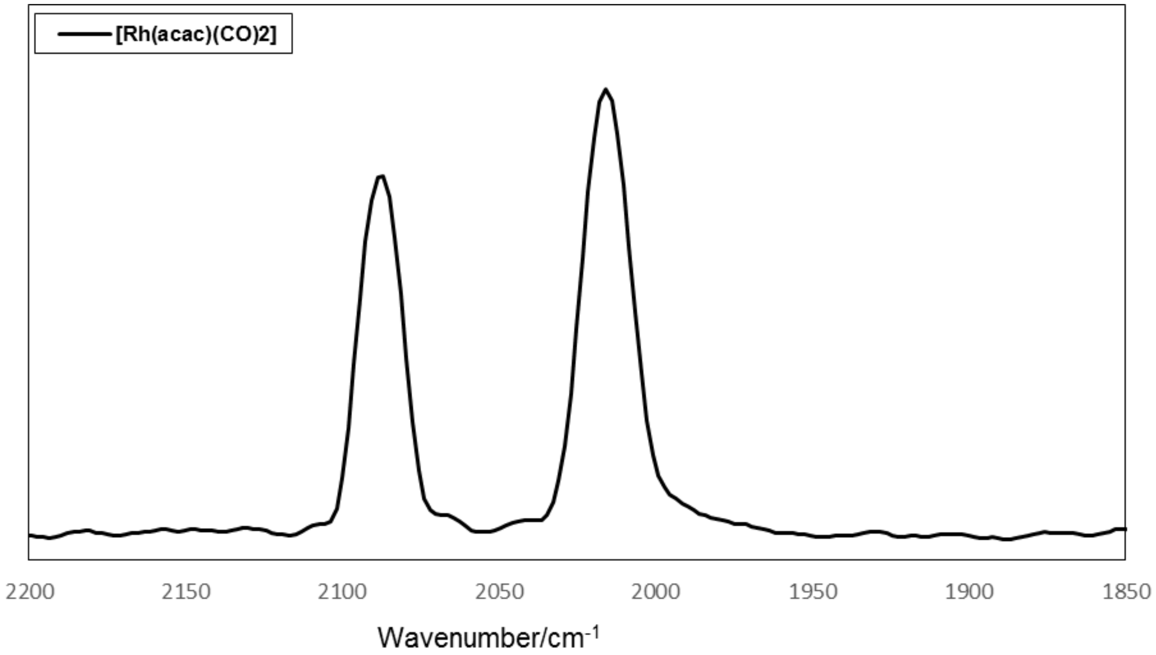

Supplementary Figure 11 IR spectra of [Rh(acac)$(CO)_2$] (black)

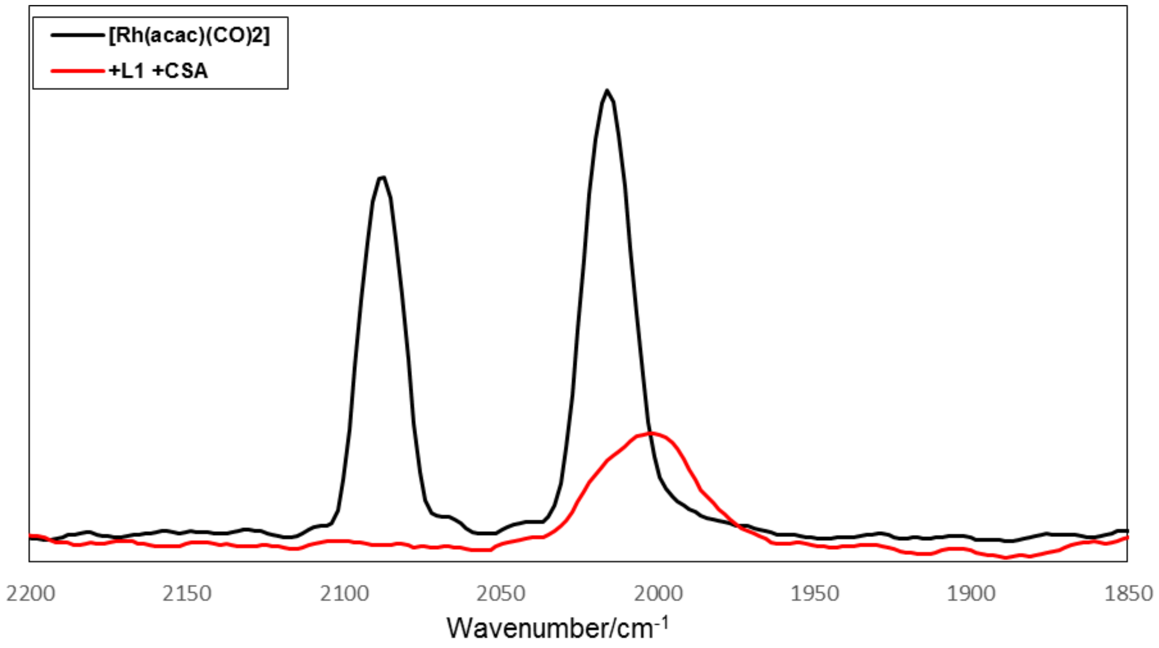

Supplementary Figure 12 IR spectra of [Rh(acac)(CO)$_2$] (black) and after addition of **L1** and CSA (red)

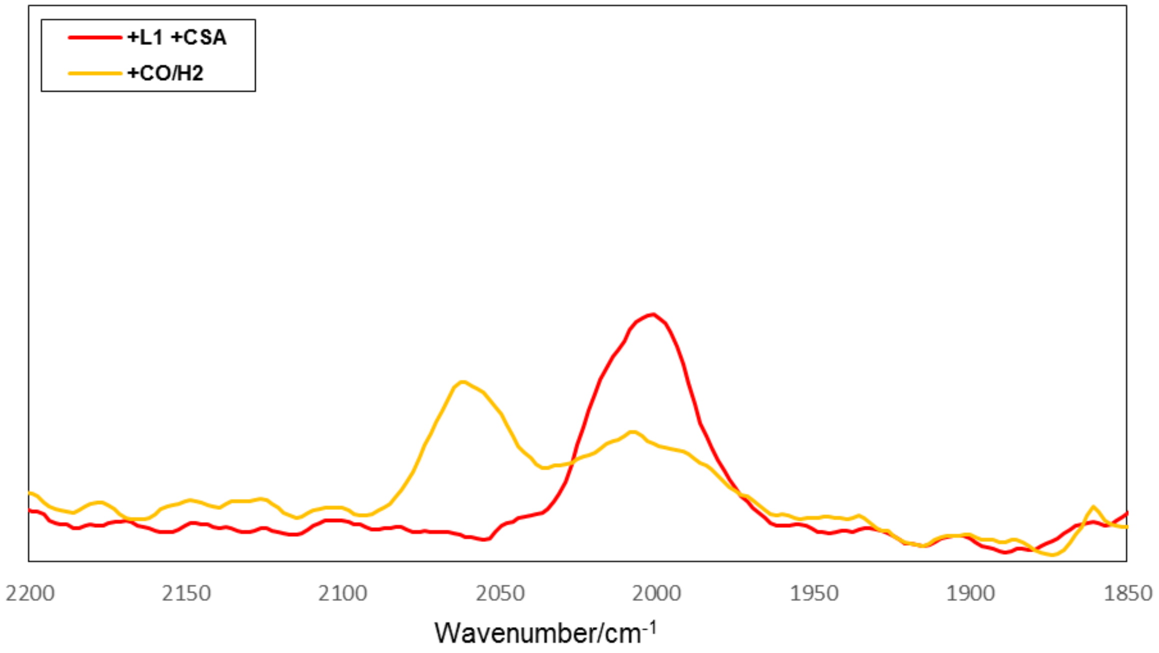

Supplementary Figure 13 IR spectra after addition of **L1** and CSA (red) and then after pressurizing CO/H_2 (1:1, 5 bar) (orange)

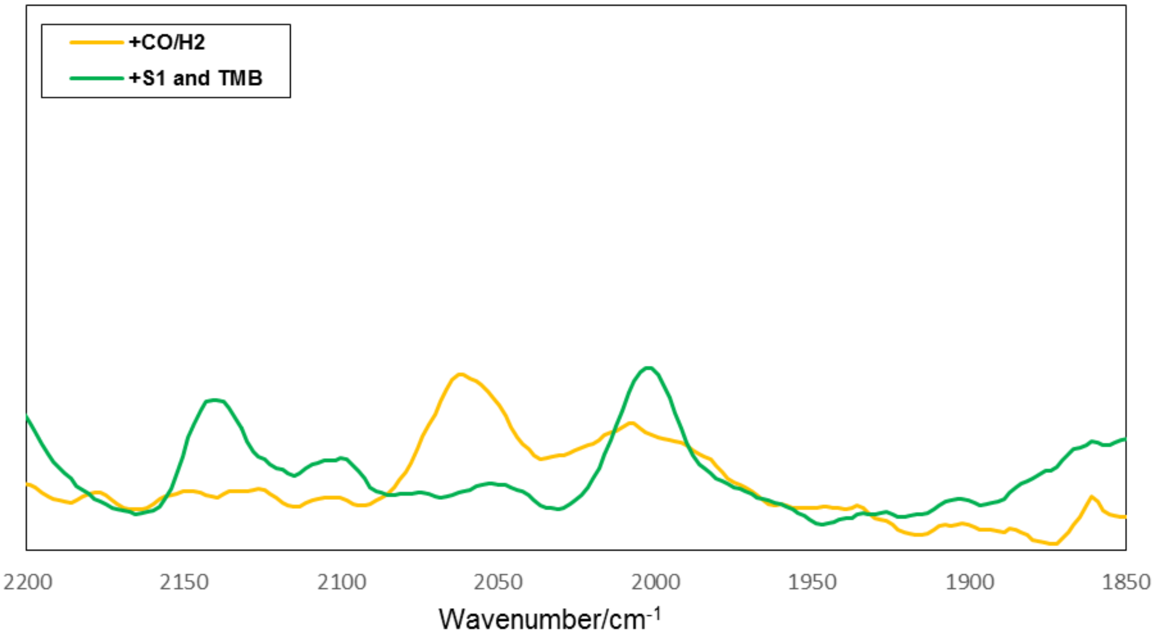

Supplementary Figure 14 IR spectra after pressurizing CO/H_2 (1:1, 5 bar) (organge) and then after adding S1 and TMB (green)

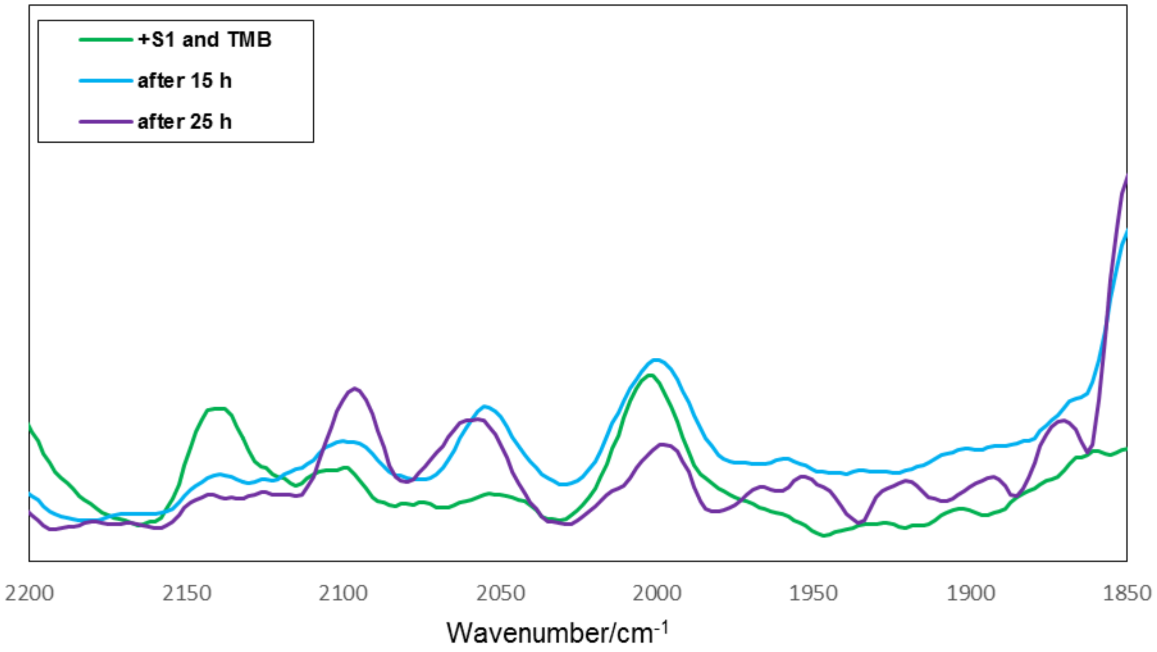

Supplementary Figure 15 IR spectra after adding S1 and TMB (green), then after repressurizing with CO/H_2 (1:1, 5 bar) and waiting for 15 h (blue) and 25 h (purple).

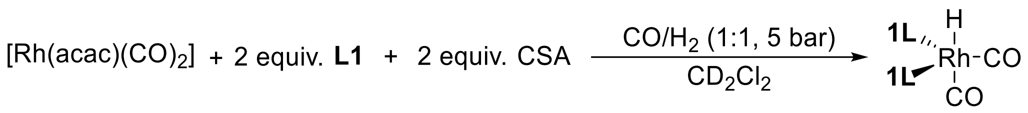

Supplementary Figure 16 Rh complex formed in the catalysis

High pressure in-situ-NMR. Procedure: In a flame heated Schlenk flask $[Rh(acac)(CO)_2]$ (2.30 mg, 8.90 µmol), **L1** (11.6 mg, 12.7 µmol, 2.1 eq.) and CSA (4.40 mg, 12.7 µmol, 2.1 eq.) was dissolved in CD_2Cl_2 (0.7 ml).After stirring at r.t. for 5 min, the solution was transferred to a sapphire-high-pressure NMR tube connected to a special tube. The NMR tube was purged three times with 5 bar of synthesis gas (CO/H_2, 1:1) and was pressurized to 5 bar. After 24 h at r.t., the formed Rh complex was observed by NMR-spectroscopy under CO/H_2 pressure using a Bruker Avance 500 instrument.

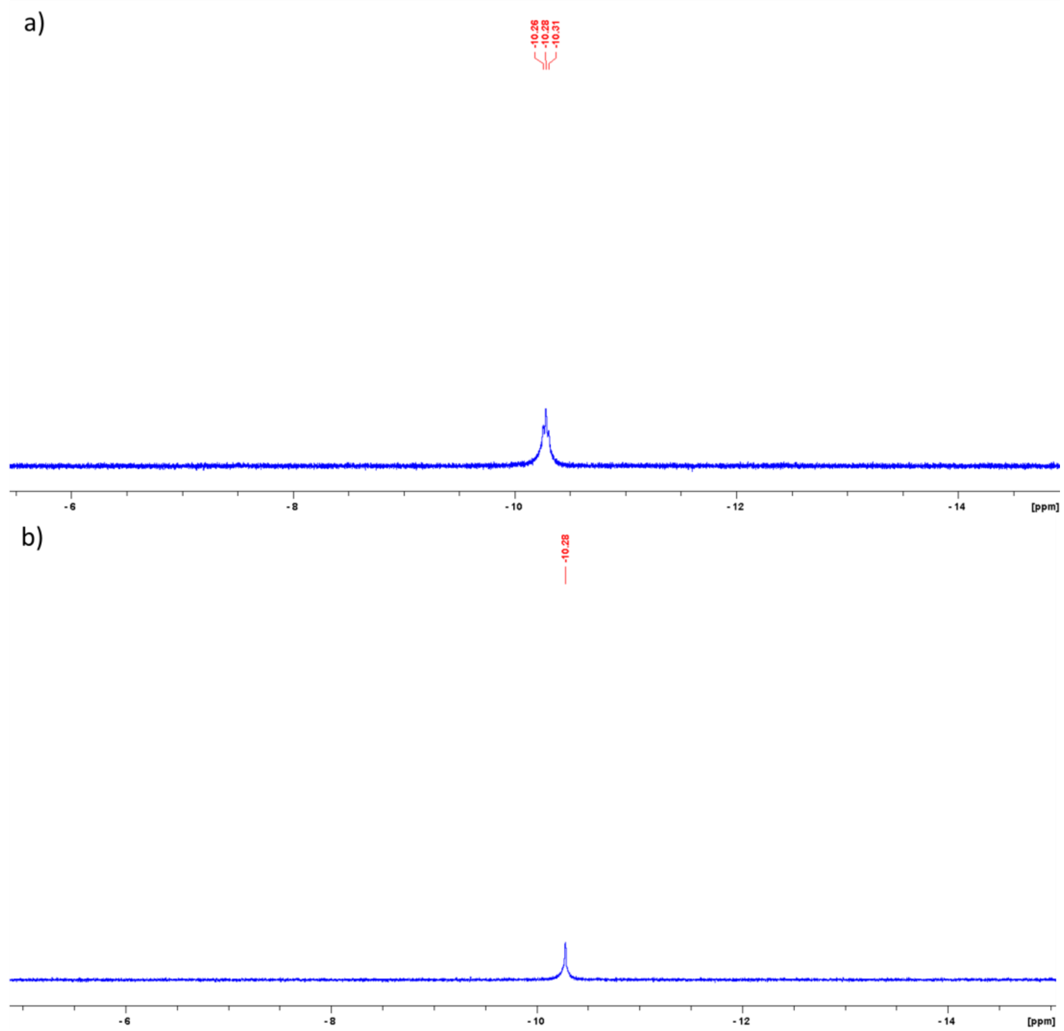

Supplementary Figure 17 ^{1}H-NMR-spectrum with (a, $J_{H\text{-}P}$ = 12.2 Hz) and without (b) coupling with P atom.

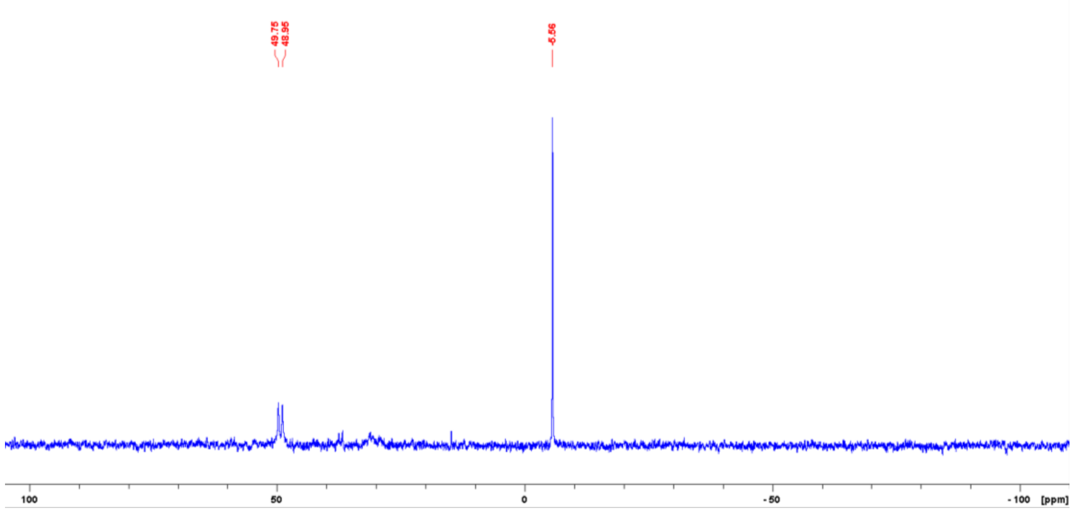

Supplementary Figure 18 *^{31}P-NMR spectrum; $J_{Rh\text{-}P}$ = 164.2 Hz*

Conclusion: As seen in Fig. S10, the formation of characteristic signals (δ = 7.64 ppm) of rhodium hydride species was observed, which showed a triplett peak with a coupling constant of $J_{H\text{-}P}$ = 12.2 Hz and it meant that two **L1** ligands coordinated to the Rh center. The coupling constant of Rh-P ($J_{Rh\text{-}P}$ = 164.2 Hz, Fig. S11) indicated a trigonal bipyramidal structure with an equatorial-equatorial conformation for the Rh complex.

DFT-Calculation

In order to understand the binding mode of the found catalytic system we optimized the geometry of a [(**S1**)HRh(**L1**+H^+)$_2$(CO)] pi-complex by DFT using Gaussian 09.[14] The structure was optimized by the BP86-functional[15,16] in combination with the def2SVP[17] basic set. The solvent DCE was considered by the IEFPCM model.

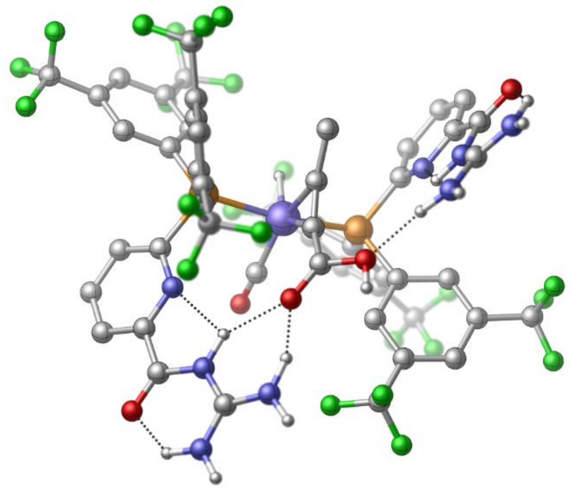

Supplementary Figure 19 DFT optimized structures of [(**S1**)HRh(**L1**+H^+)$_2$(CO)]

Conclusion: The nitrogen atoms of the two pyridine rings form hydrogen bonds with the acylguanidinium groups and thereby fix their orientation. The carbonyl oxygen of the carboxyl group of **S1** is coordinated by two hydrogen bonds from one of the acylguanidinium groups and the OH group by only one hydrogen bond from the other ligand. These interactions between the carboxyl group of **S1** and the ligands seem to control the orientation of the substrate effectively and only allow one possible hydrometalation. This is in perfect agreement with our experimental observations.

Supplementary Table 14 Cartesian coordinates (Å)

P	-2.4508819	0.5136169	-0.6061368	N	1.8528961	-4.2378271	3.4752822
P	2.0826721	1.1503409	0.3864392	H	0.8032571	-5.6658891	-3.6592128
C	-3.5347299	1.9536889	-0.1593418	H	1.1972991	-4.4420811	0.1716712
C	-3.3294139	-0.9158161	0.1937602	N	-0.4856829	-4.6340141	-5.6615398
C	3.3598981	-0.1151821	-0.0803728	H	-1.0473749	-4.0652011	-6.3191778
C	2.4290071	1.3859449	2.2053322	C	0.0663011	-1.4664091	0.3653842
C	-1.8241329	-2.2459911	-4.9324128	C	-0.3087559	-0.7636111	1.4023282
C	-5.1841869	4.1545879	0.4918062	N	2.5507861	-3.7777311	5.6705632
C	-2.9706149	3.1436929	0.3307022	H	2.4243101	-4.7427141	5.9814112
C	-4.9363409	1.8678369	-0.3248678	H	2.8358811	-3.0347751	6.3309842
C	-5.7518569	2.9627299	0.0054922	H	1.7353511	-5.2338881	3.6728352
C	-3.7918409	4.2404829	0.6500082	C	-0.7971509	-0.8035851	2.7978162
H	-1.8796039	3.2007099	0.4742482	H	-1.8745789	-0.5350221	2.8355832
H	-5.4055869	0.9509729	-0.7117488	H	-0.6776959	-1.8067271	3.2598982
H	-5.8268239	5.0098579	0.7472072	H	-0.2772349	-0.0495431	3.4234242
C	-4.4959439	-3.1276091	1.5223502	C	-2.4286559	-1.0112681	-4.3163498
C	-3.1540619	-2.2220551	-0.3078658	C	-3.1108789	-0.1115211	-5.1585248
C	-4.0748159	-0.7266341	1.3751122	N	-2.2724659	-0.8165411	-2.9938418
C	-4.6595559	-1.8288621	2.0276202	C	-3.6383849	1.0512139	-4.5838638
C	-3.7350549	-3.3160831	0.3551702	H	-3.1989809	-0.3301231	-6.2319178
H	-2.5643429	-2.3847191	-1.2200578	C	-2.8025969	0.2886759	-2.4391678
H	-4.2075169	0.2783789	1.8023372	C	-3.4832729	1.2619909	-3.2036528
H	-4.9523699	-3.9848391	2.0363592	H	-4.1641739	1.7947389	-5.2015858
C	5.2792801	-2.0169831	-0.9161858	H	-3.8815989	2.1736989	-2.7384568
C	4.5819471	-0.2439611	0.6107692	Rh	-0.1266489	0.6184009	-0.2017738
C	3.1017171	-0.9483451	-1.1881468	H	-0.3956129	2.0422009	0.5275472
C	4.0564091	-1.8938331	-1.5994118	C	0.1988081	1.1782259	-2.0059448
C	5.5348851	-1.1906711	0.1892052	O	0.3842091	1.5673689	-3.0875898
H	4.8122651	0.3936189	1.4765132	H	1.5760041	-3.8970051	2.5406492
H	2.1465421	-0.8649191	-1.7271888	H	-0.0232329	-5.4980221	-5.9498758
H	6.0316221	-2.7479821	-1.2445978	C	-7.2610079	2.8495959	-0.1112958
C	2.9062131	1.5062649	4.9333062	C	-3.1716439	5.5420589	1.1236272
C	2.6133531	2.6479589	2.8128362	C	-5.4864979	-1.5834421	3.2747352
N	2.4584571	0.2460919	2.9127672	C	-3.4684059	-4.7172901	-0.1561878
C	2.6949221	0.3031069	4.2382192	C	3.7492651	-2.8329521	-2.7492118

C	2.8540921	2.7003989	4.1962772	C	6.8265331	-1.3345661	0.9724202
H	2.5793551	3.5713589	2.2170052	C	5.9676521	4.2461629	-1.6986618
C	2.7130021	-1.0217411	4.9559352	C	1.2931501	6.1488039	-1.3042468
H	3.0079041	3.6699729	4.6933322	F	6.6770571	4.7727979	-0.6667618
H	3.1017501	1.4917679	6.0149852	F	6.1251861	5.0759379	-2.7566668
C	0.3277131	-2.7758551	-0.1580828	F	6.5428471	3.0599599	-2.0138268
O	-0.0145529	-3.1801391	-1.2834518	F	1.7054901	7.0312929	-2.2427418
O	1.0545291	-3.5984551	0.6608802	F	0.0640111	5.7025319	-1.6682338
O	-2.0040959	-2.5299671	-6.1196708	F	1.1381961	6.8356299	-0.1431178
N	-1.0520759	-3.0144611	-4.0658098	F	6.6249691	-2.0381491	2.1185712
C	-0.4015629	-4.1887481	-4.4101338	F	7.3238501	-0.1260861	1.3328832
H	-0.9425299	-2.6709711	-3.0912038	F	7.7803021	-1.9811181	0.2651672
N	0.2765291	-4.8160351	-3.4518898	F	4.8491371	-3.0984641	-3.4901888
H	0.3142521	-4.4004361	-2.4992498	F	3.2853371	-4.0312271	-2.2879078
C	2.7148431	2.7239249	-0.3738788	F	2.8013311	-2.3332101	-3.5768008
C	3.6124931	5.1487049	-1.5215108	F	-3.3500219	-4.7481541	-1.5063348
C	4.0635741	2.8725639	-0.7601338	F	-4.4481129	-5.5823001	0.1927182
C	1.8197241	3.7946709	-0.5704558	F	-2.3029149	-5.2084991	0.3536552
C	2.2706481	5.0006419	-1.1359958	F	-5.7073979	-2.7217311	3.9716912
C	4.5051501	4.0802929	-1.3281088	F	-6.6969249	-1.0510851	2.9706872
H	4.7810321	2.0485839	-0.6411018	F	-4.8663149	-0.7052261	4.1070492
H	0.7602841	3.6754419	-0.2975108	F	-7.6278389	1.8839389	-0.9875968
H	3.9565791	6.0837559	-1.9854688	F	-7.8222049	2.5414889	1.0861302
N	2.4088211	-2.0729221	4.0908232	F	-7.8154069	4.0145029	-0.5240478
O	2.9556211	-1.1542171	6.1541682	F	-3.9402799	6.1481059	2.0602202
H	2.2598021	-1.7518731	3.1158902	F	-1.9429039	5.3470989	1.6619892
C	2.2680791	-3.3997191	4.4270112	F	-3.0270389	6.4186439	0.0975022

Copies of NMR and HR-MS (ESI) spectra

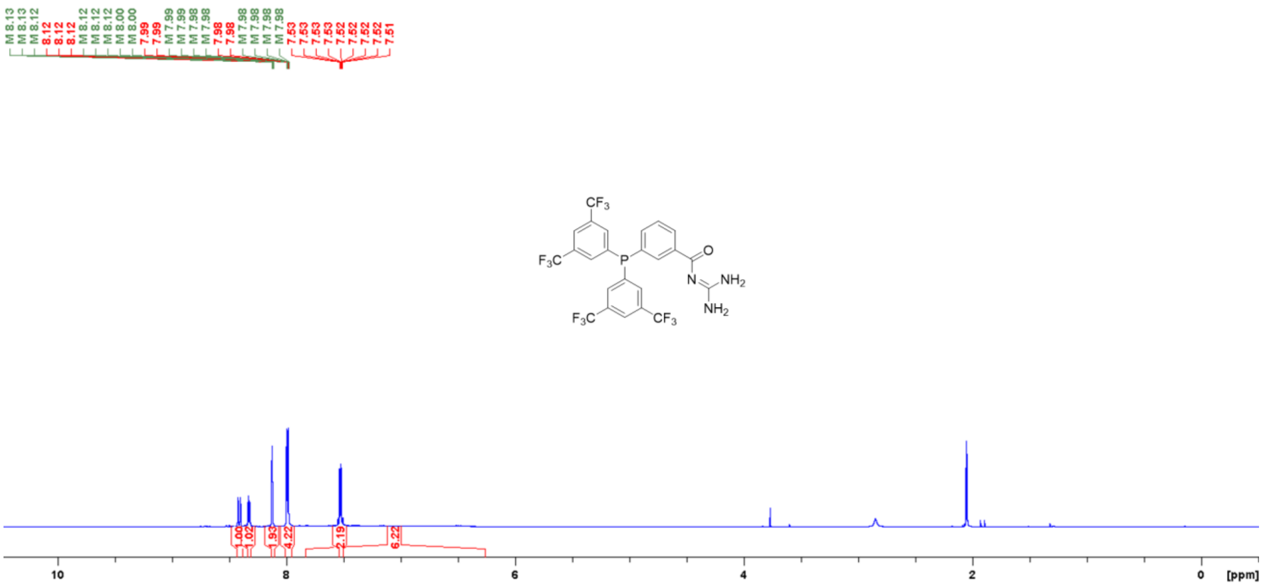

Supplementary Figure 20 ^{1}H NMR (acetone-D6, 500 MHz) spectrum of **L6**

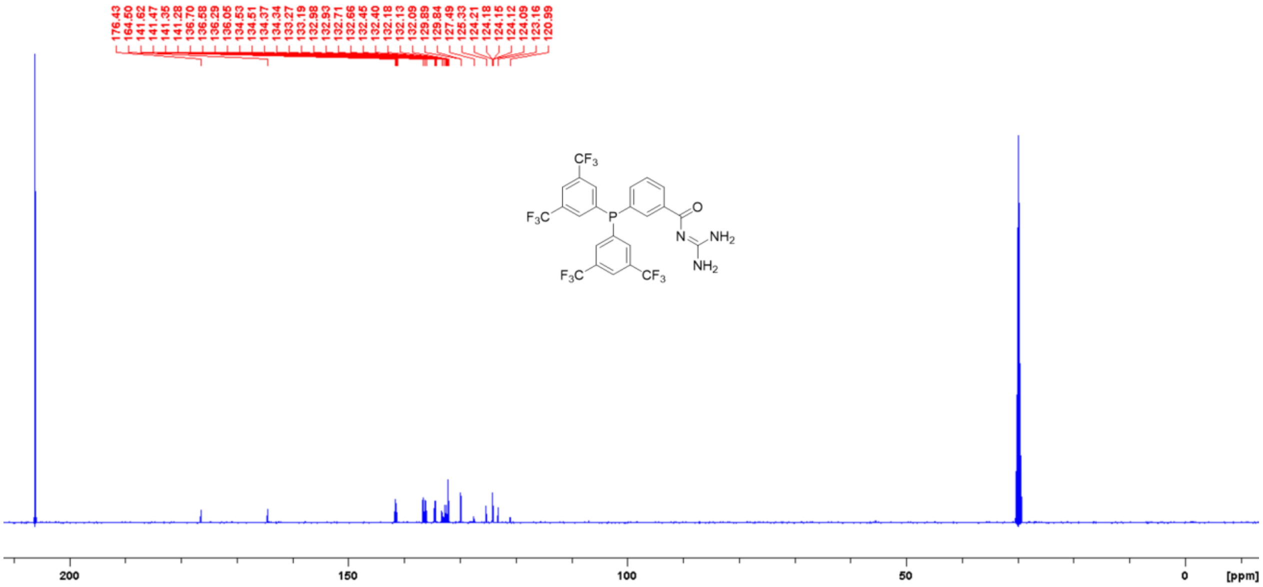

Supplementary Figure 21 ^{13}C NMR (acetone-D6, 126 MHz) spectrum of **L6**

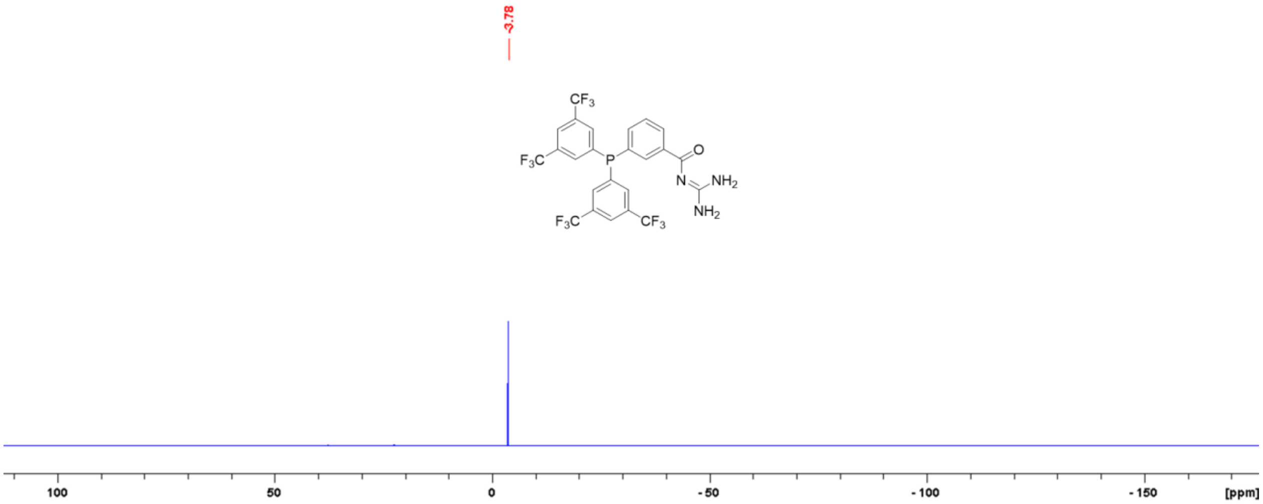

Supplementary Figure 22 ^{31}P NMR (acetone-D6, 202 MHz) spectrum of **L6**

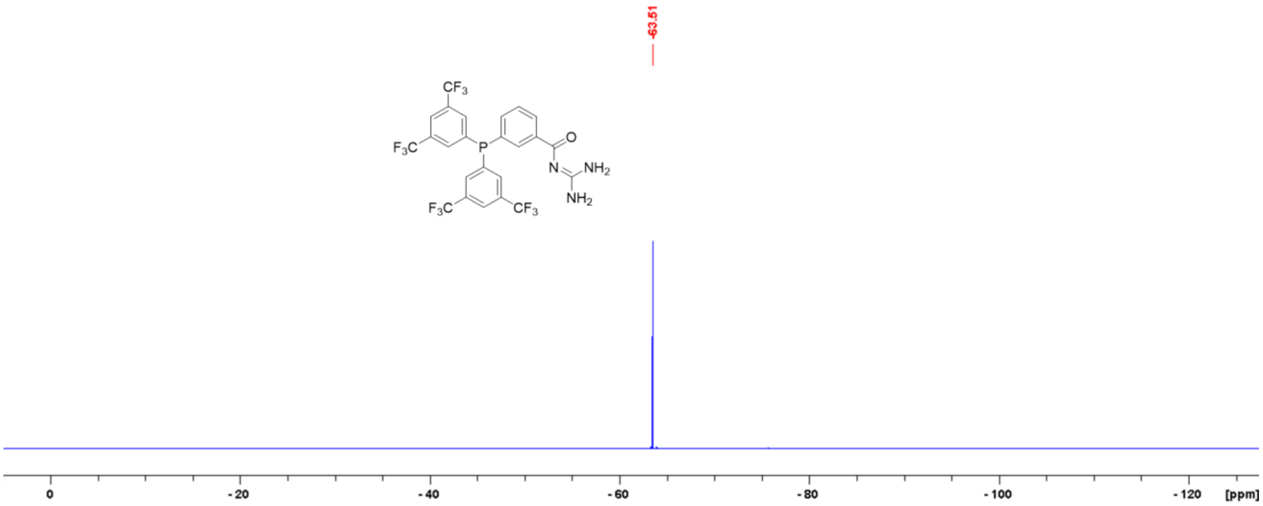

Supplementary Figure 23 ^{19}F NMR (acetone-D6, 471 MHz) spectrum of **L6**

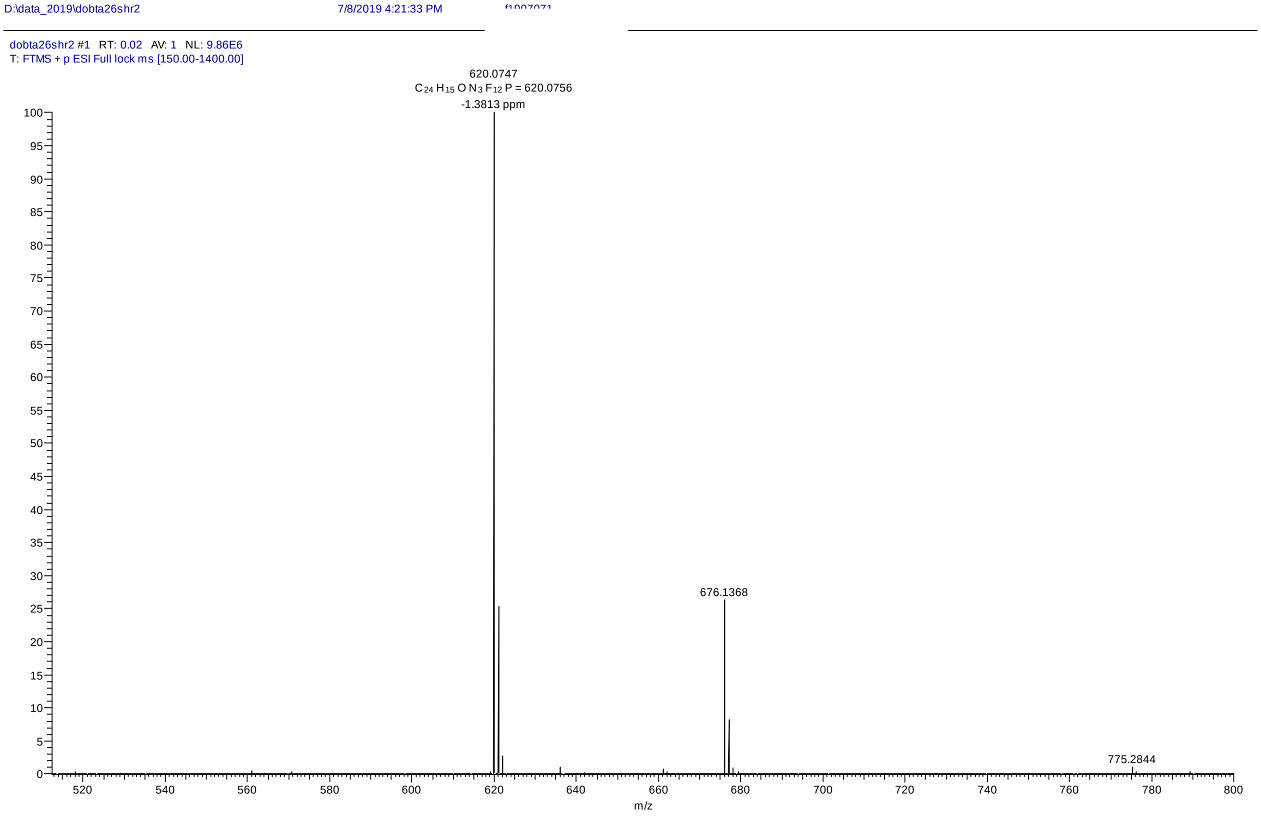

Supplementary Figure 24 ESI-MS spectra of **L6**

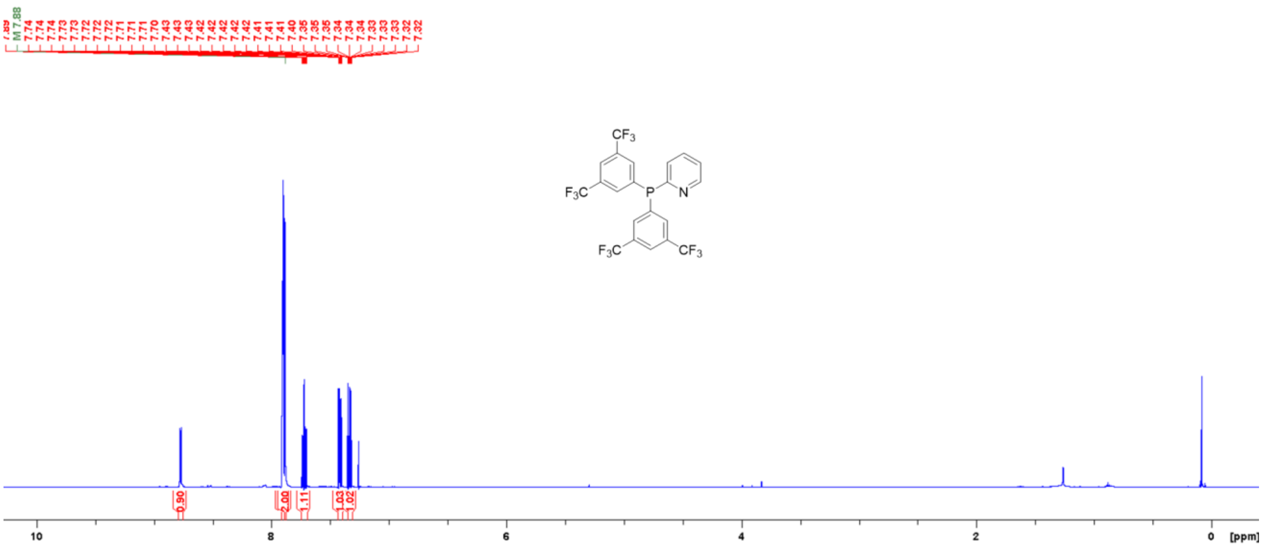

Supplementary Figure 25 ^{1}H NMR (acetone-D6, 500 MHz) spectrum of **L7**

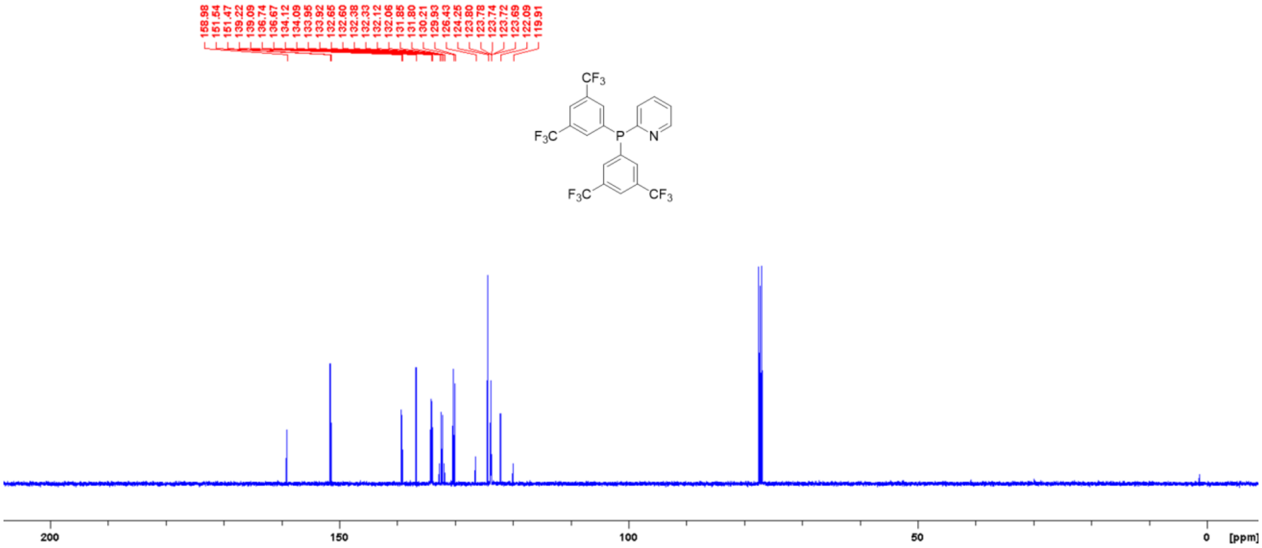

Supplementary Figure 26 ^{13}C NMR (acetone-D6, 126 MHz) spectrum of **L7**

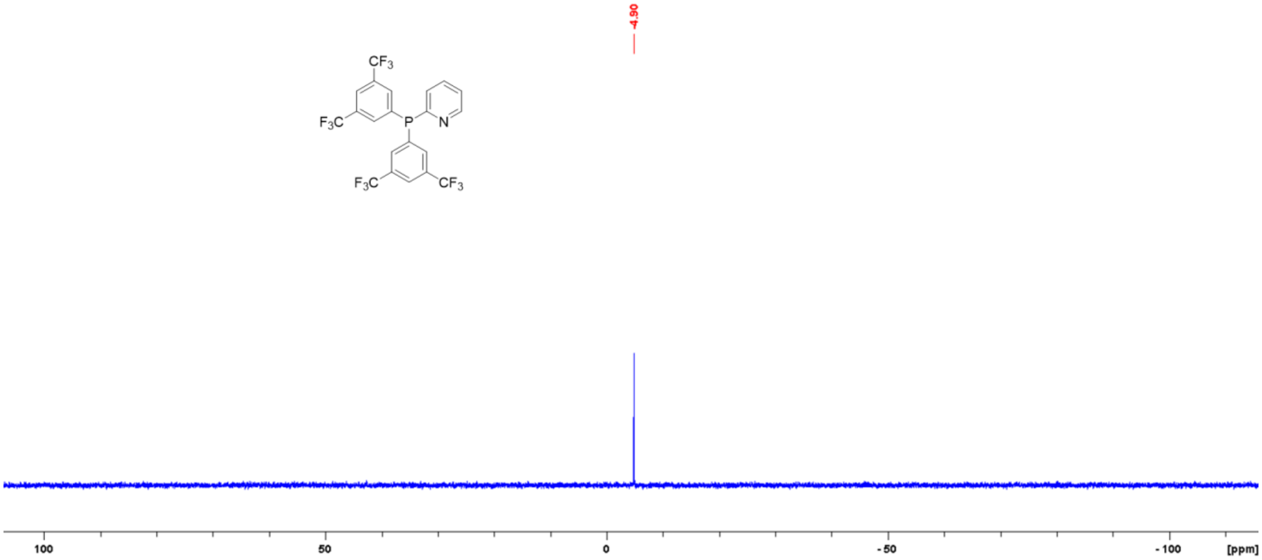

Supplementary Figure 27 ^{31}P NMR (acetone-D6, 121 MHz) spectrum of **L7**

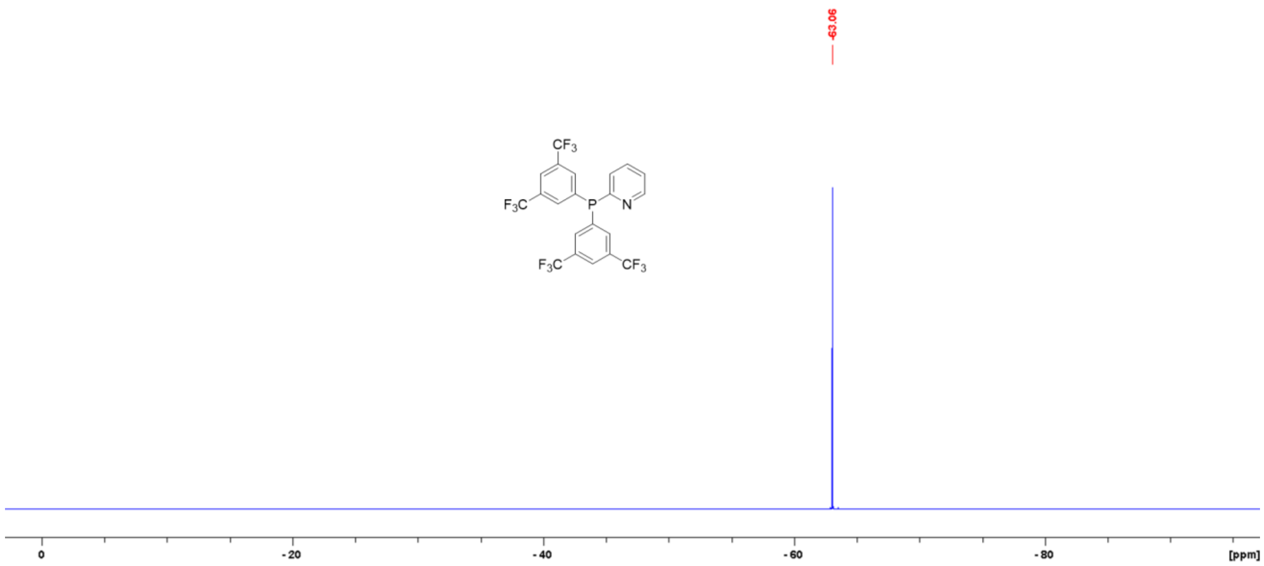

Supplementary Figure 28 ^{19}F NMR (acetone-D6, 471 MHz) spectrum of **L7**

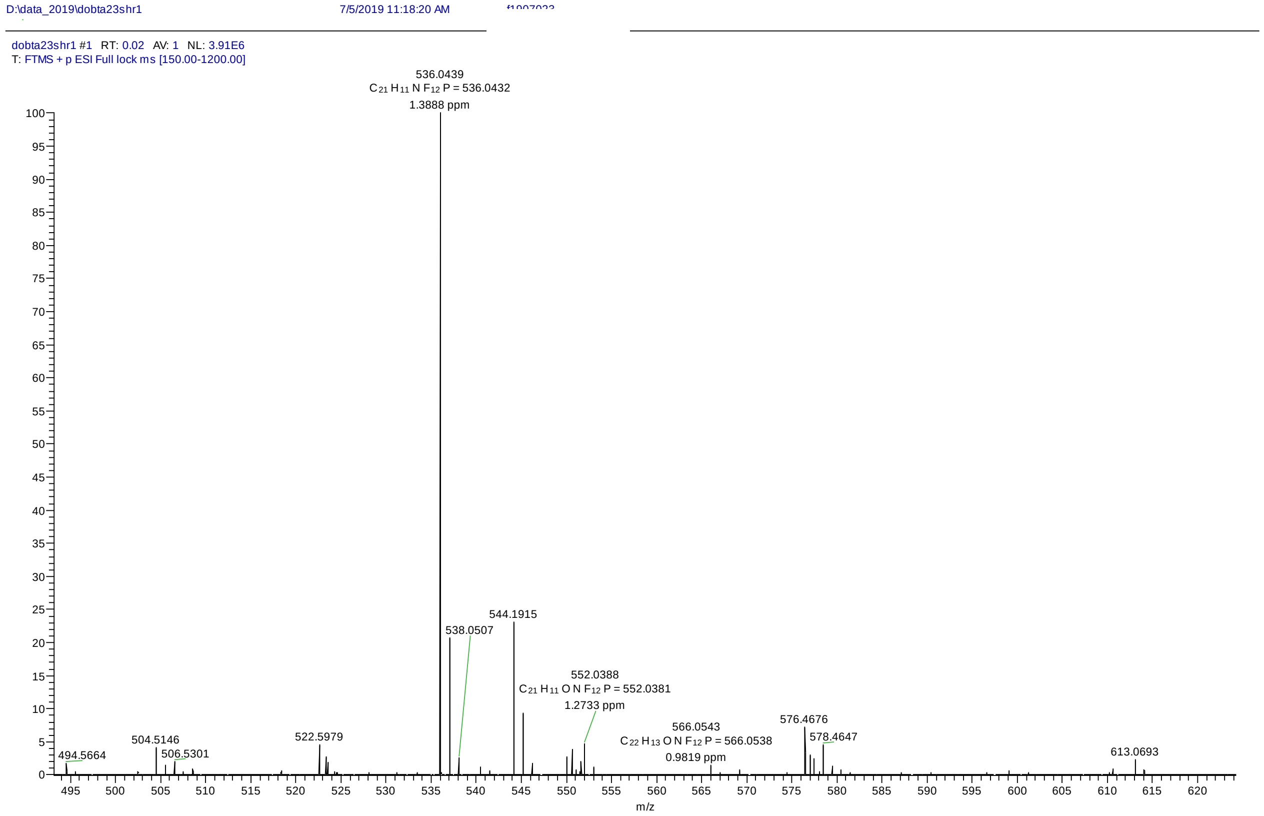

Supplementary Figure 29 ESI-MS spectra of **L7**

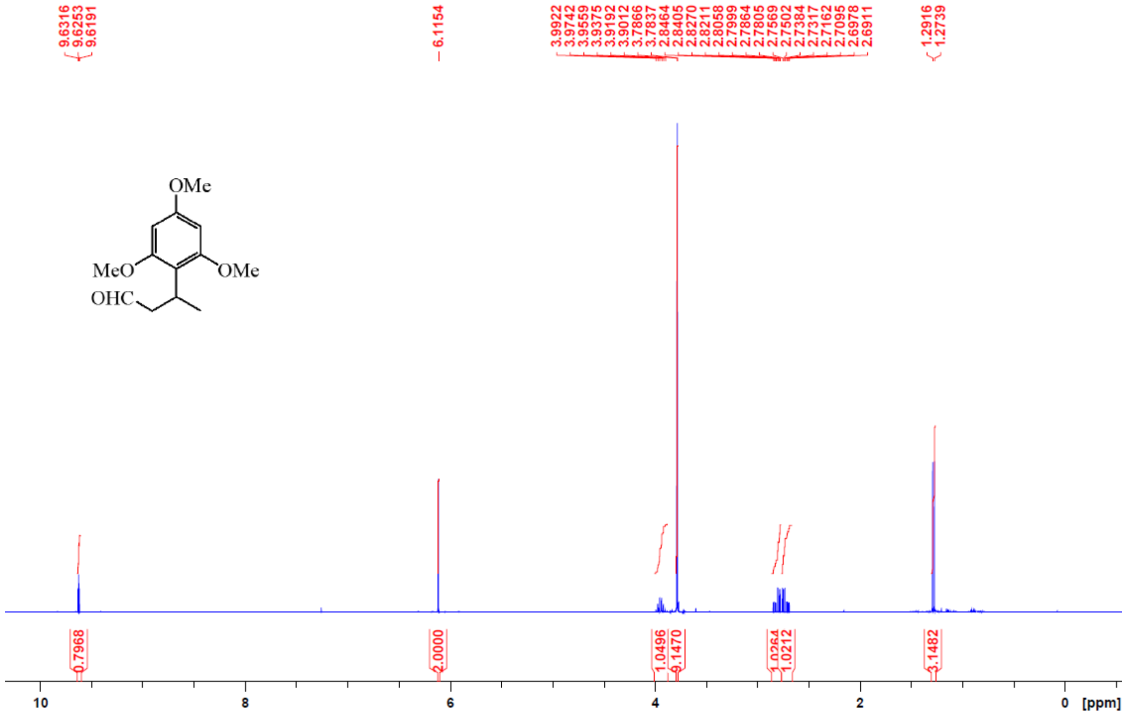

Supplementary Figure 30 ^{1}H NMR ($CDCl_3$, 400 MHz) spectrum of **1**

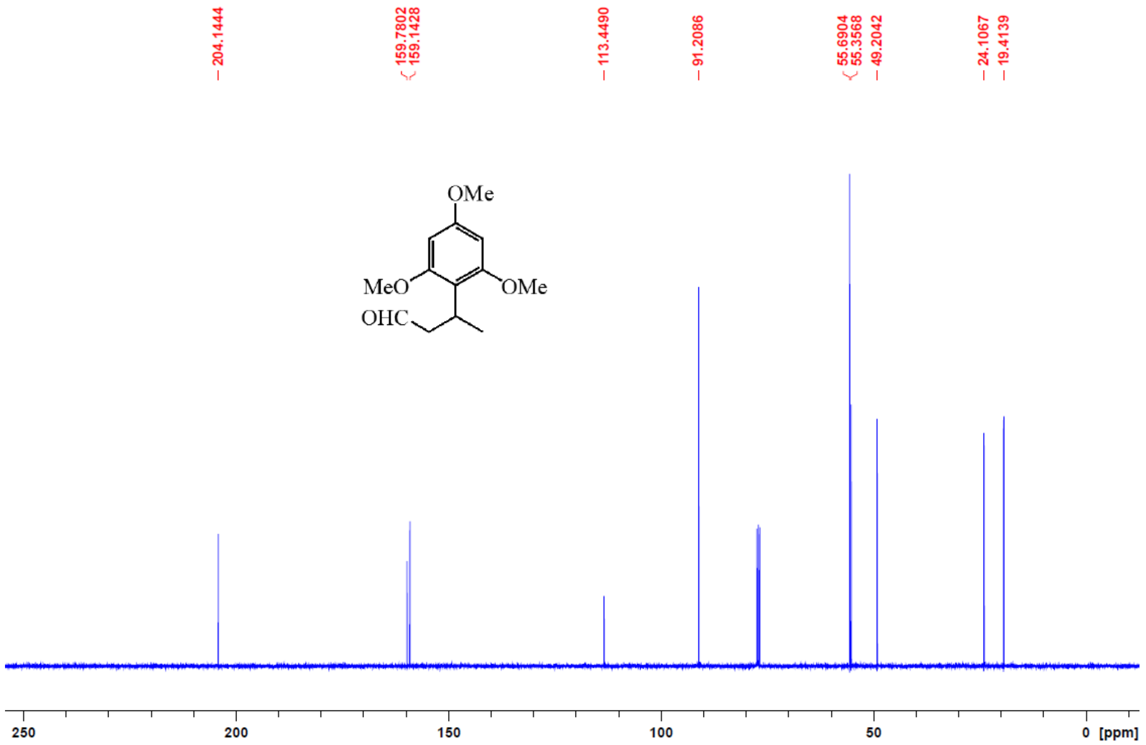

Supplementary Figure 31 ^{13}C NMR ($CDCl_3$, 100 MHz) spectrum of **1**

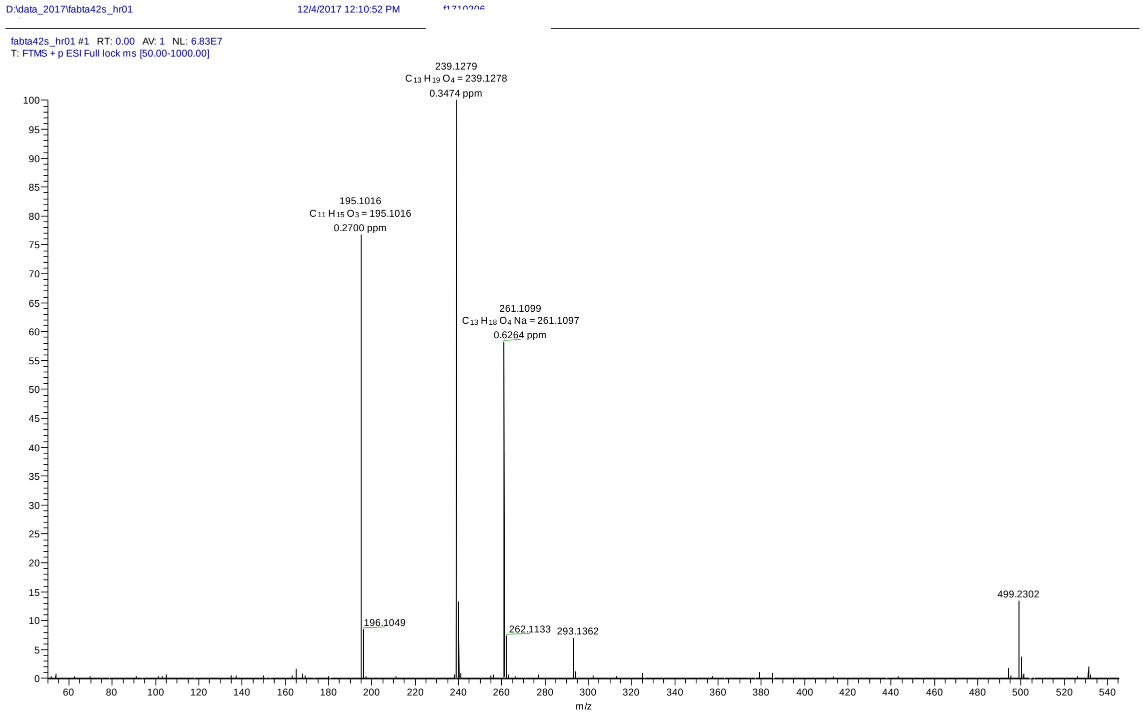

Supplementary Figure 32 ESI-MS spectra of **1**

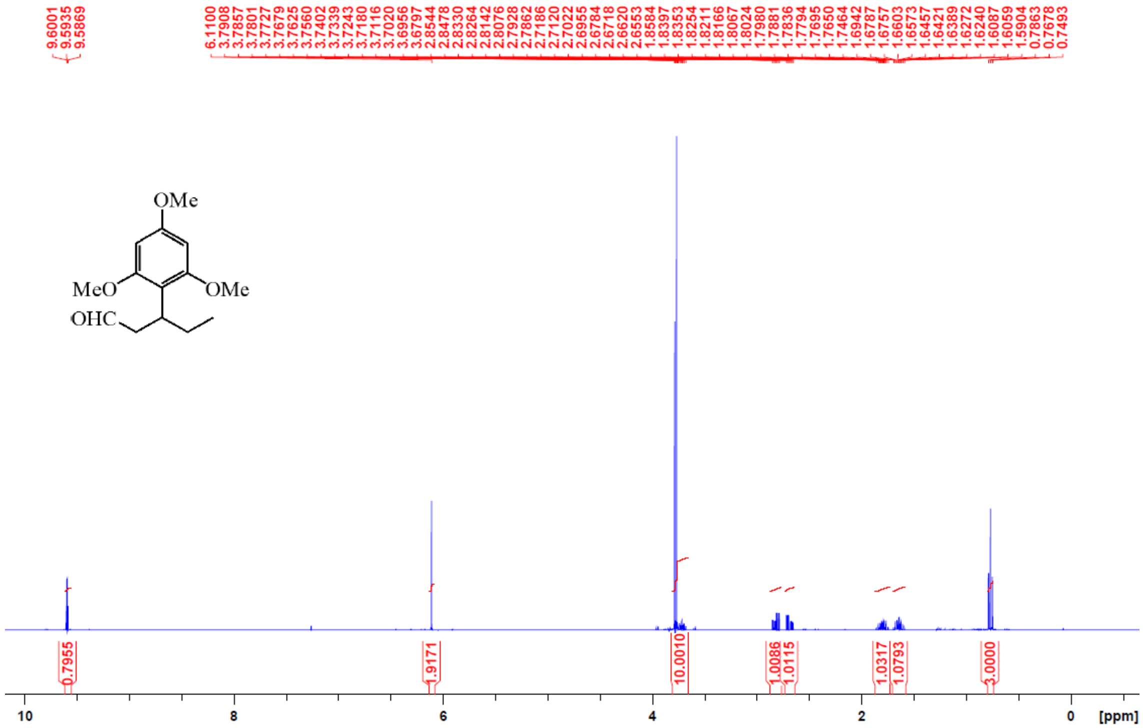

Supplementary Figure 33 ^{1}H NMR ($CDCl_3$, 400 MHz) spectrum of **2**

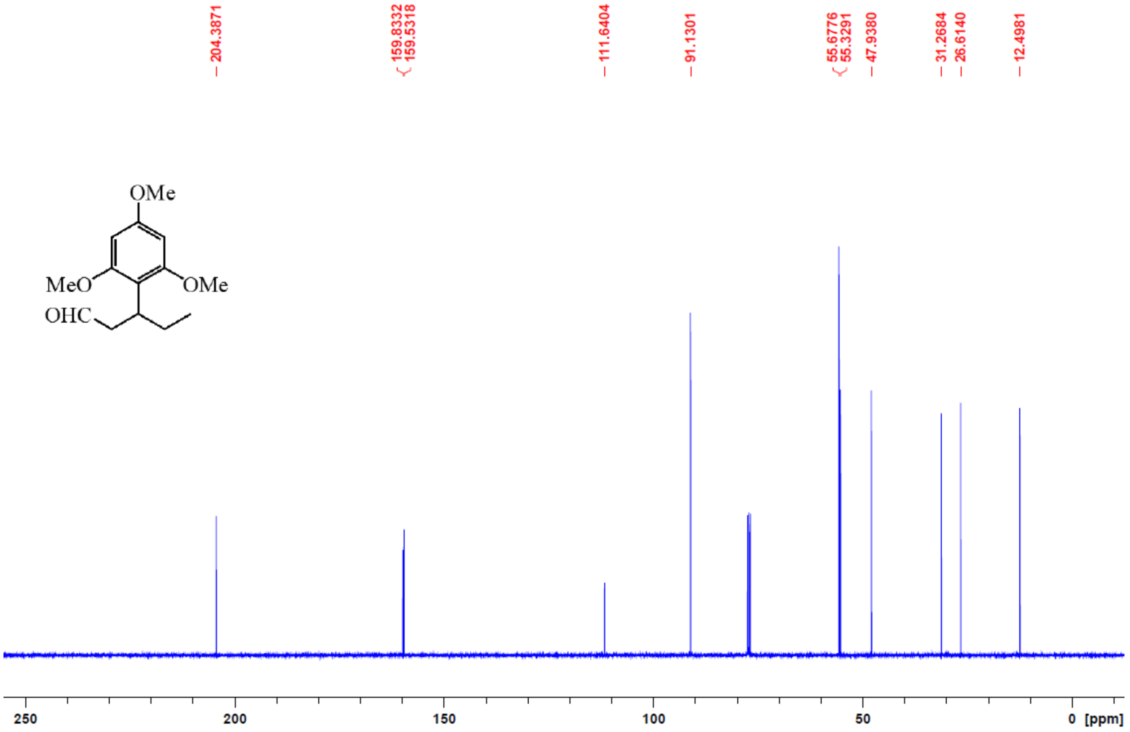

Supplementary Figure 34 ^{13}C NMR ($CDCl_3$, 100 MHz) spectrum of **2**

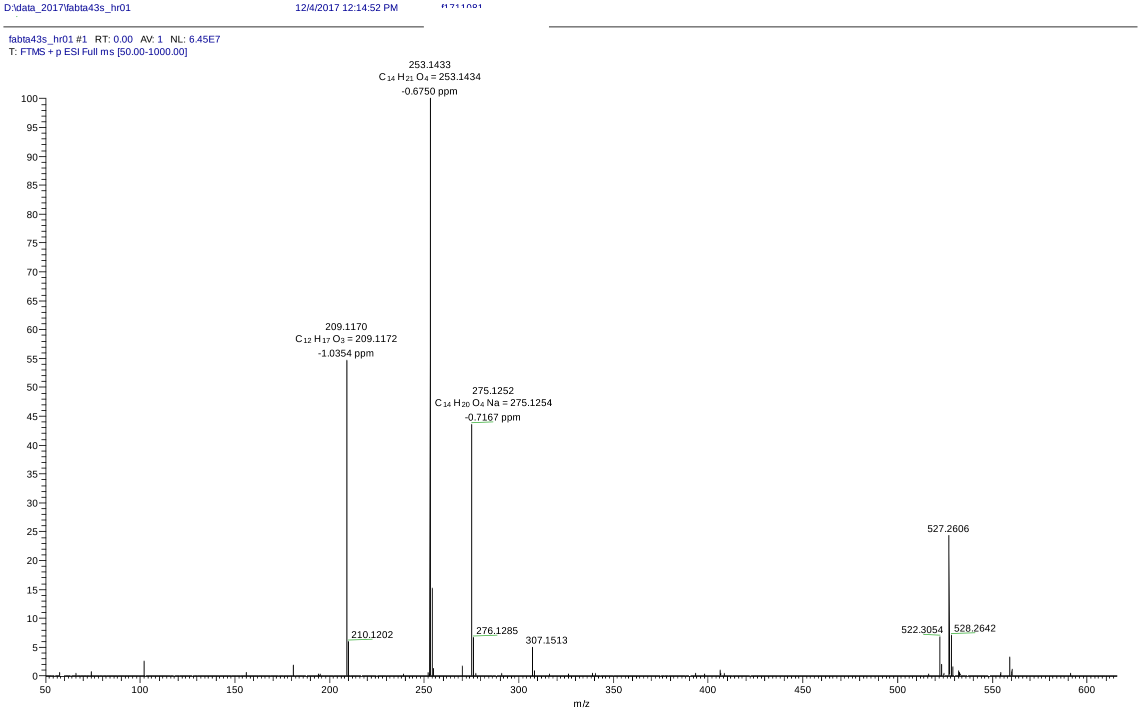

Supplementary Figure 35 ESI-MS spectra of **2**

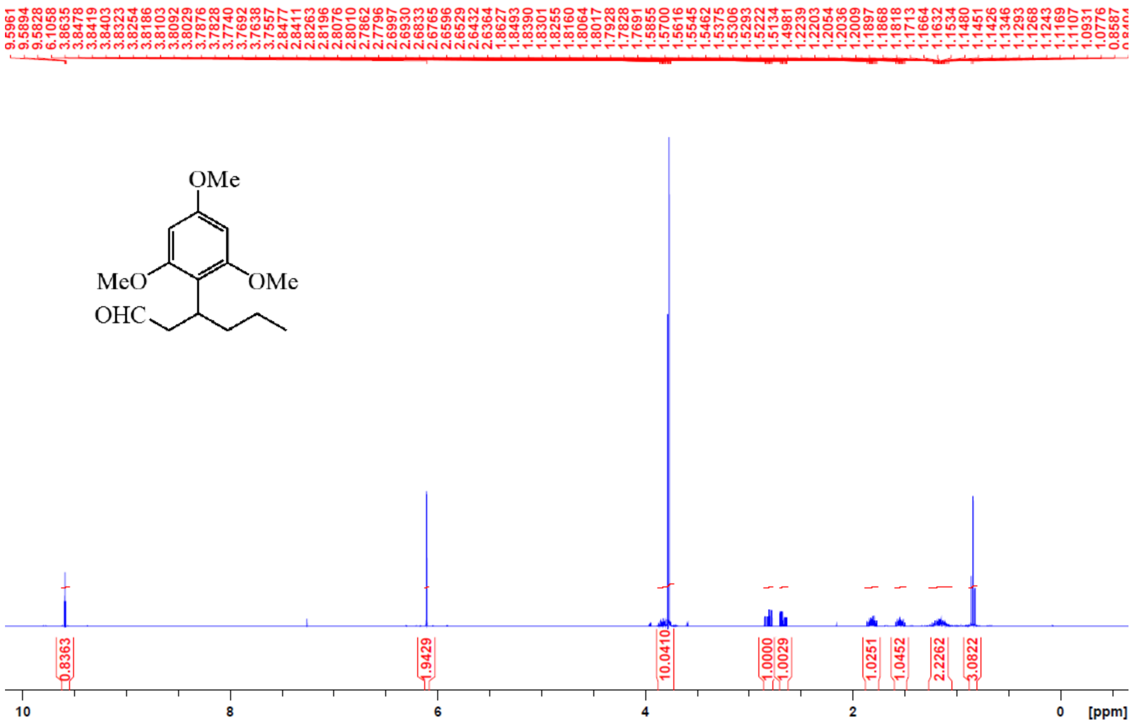

Supplementary Figure 36 ^{1}H NMR ($CDCl_3$, 400 MHz) spectrum of **3**

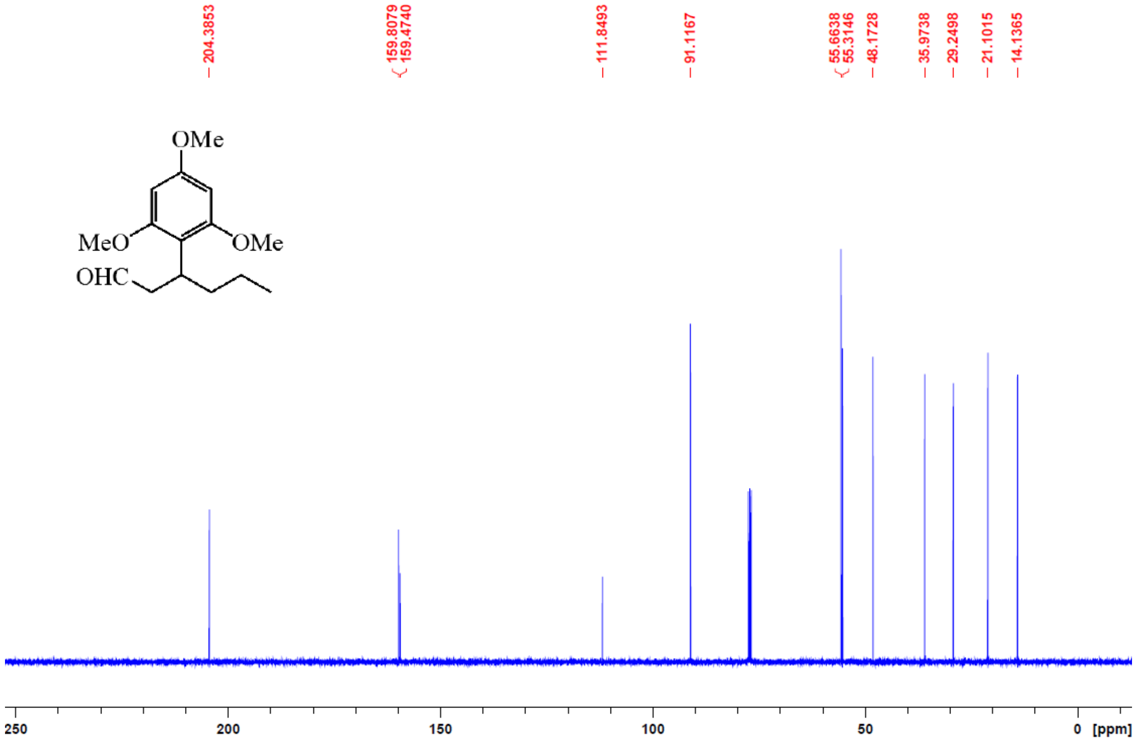

Supplementary Figure 37 ^{13}C NMR ($CDCl_3$, 100 MHz) spectrum of **3**

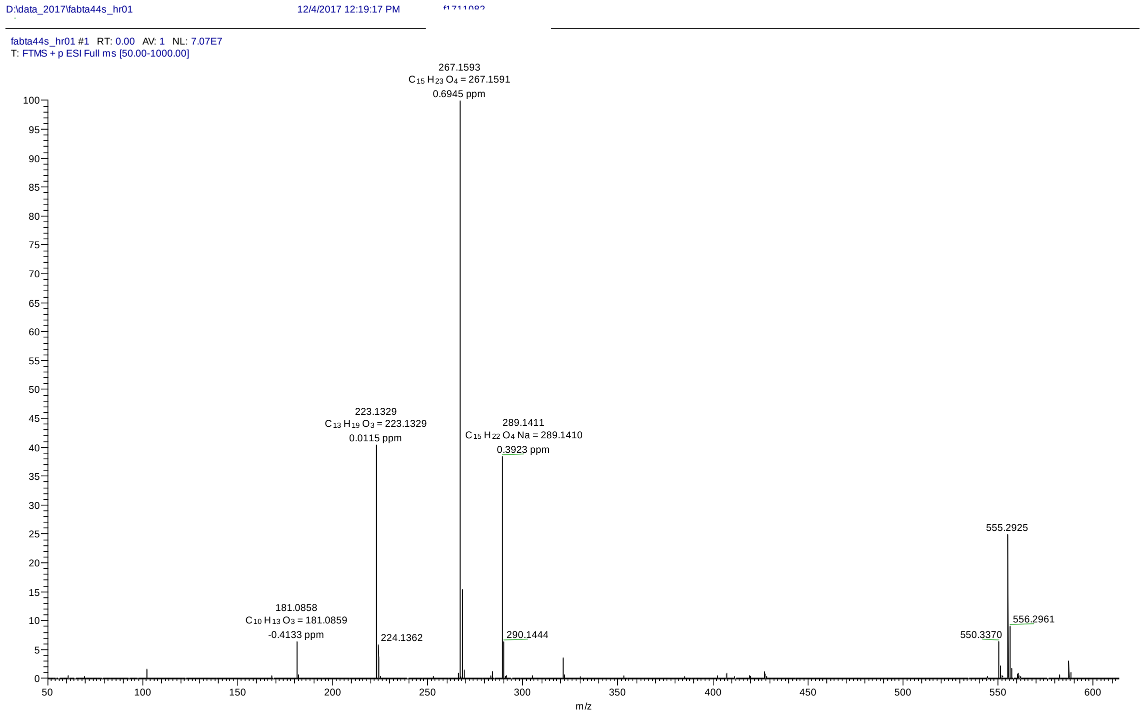

Supplementary Figure 38 ESI-MS spectra of **3**

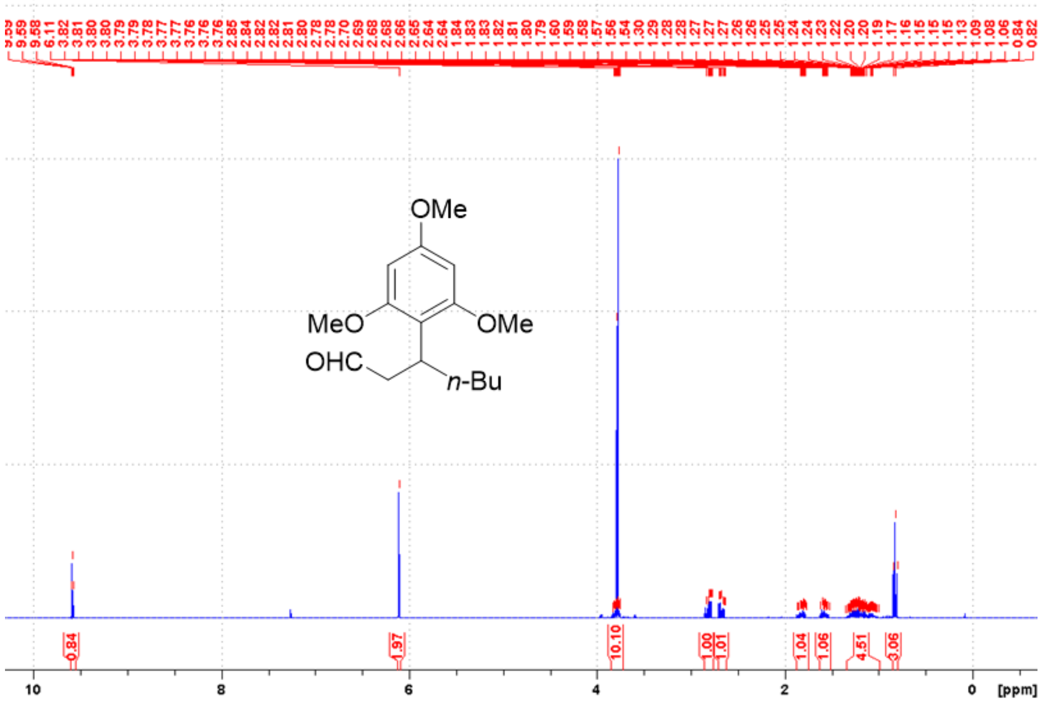

Supplementary Figure 39 ^{1}H NMR ($CDCl_3$, 400 MHz) spectrum of **4**

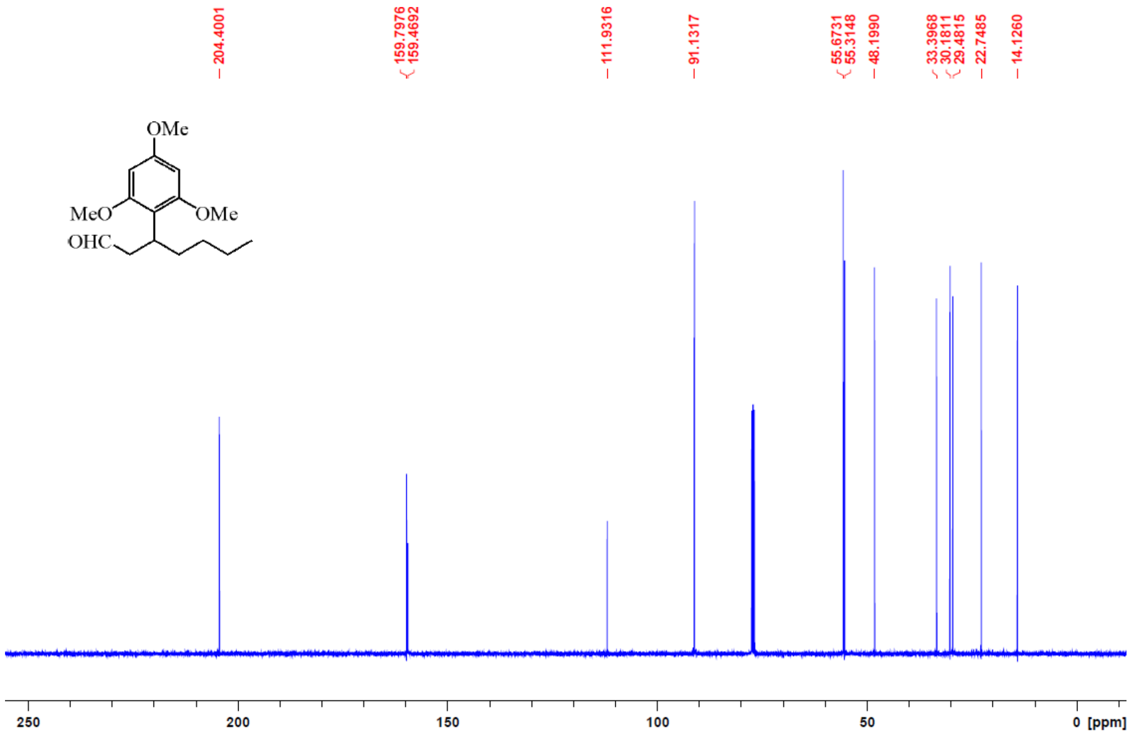

Supplementary Figure 40 ^{13}C NMR ($CDCl_3$, 100 MHz) spectrum of **4**

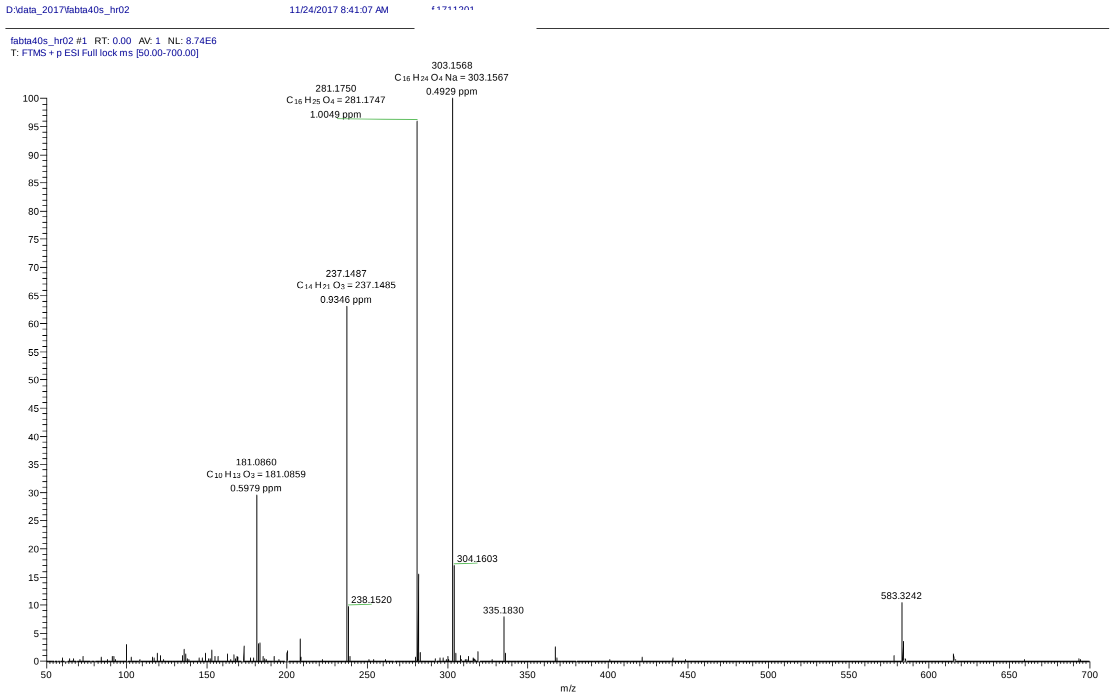

Supplementary Figure 41 ESI-MS spectra of **4**

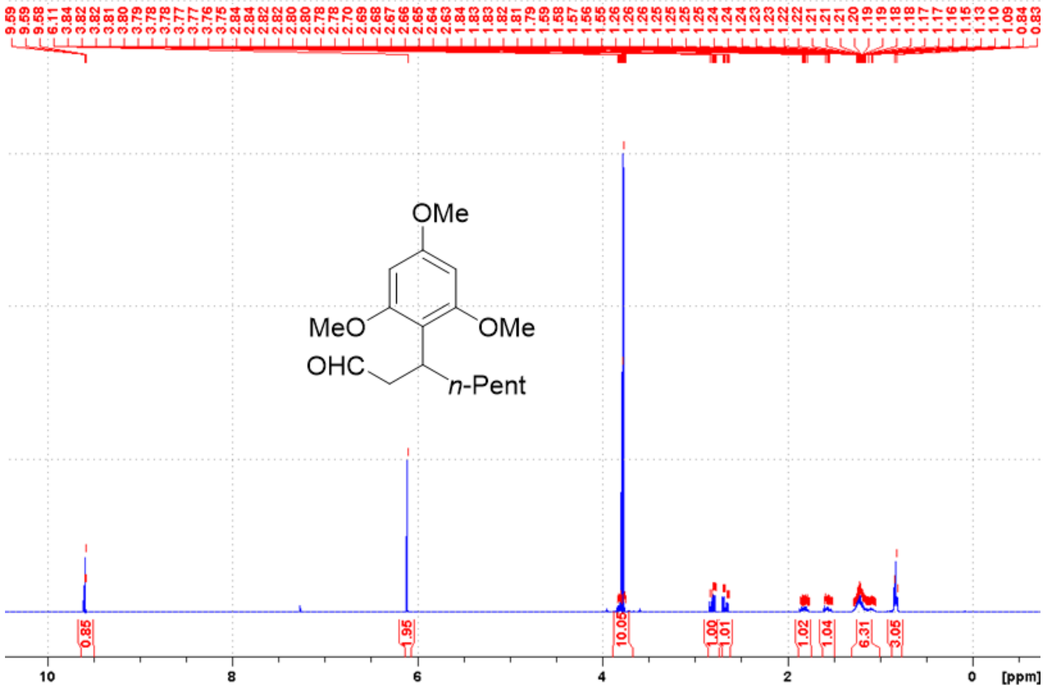

Supplementary Figure 42 ^{1}H NMR ($CDCl_3$, 400 MHz) spectrum of **5**

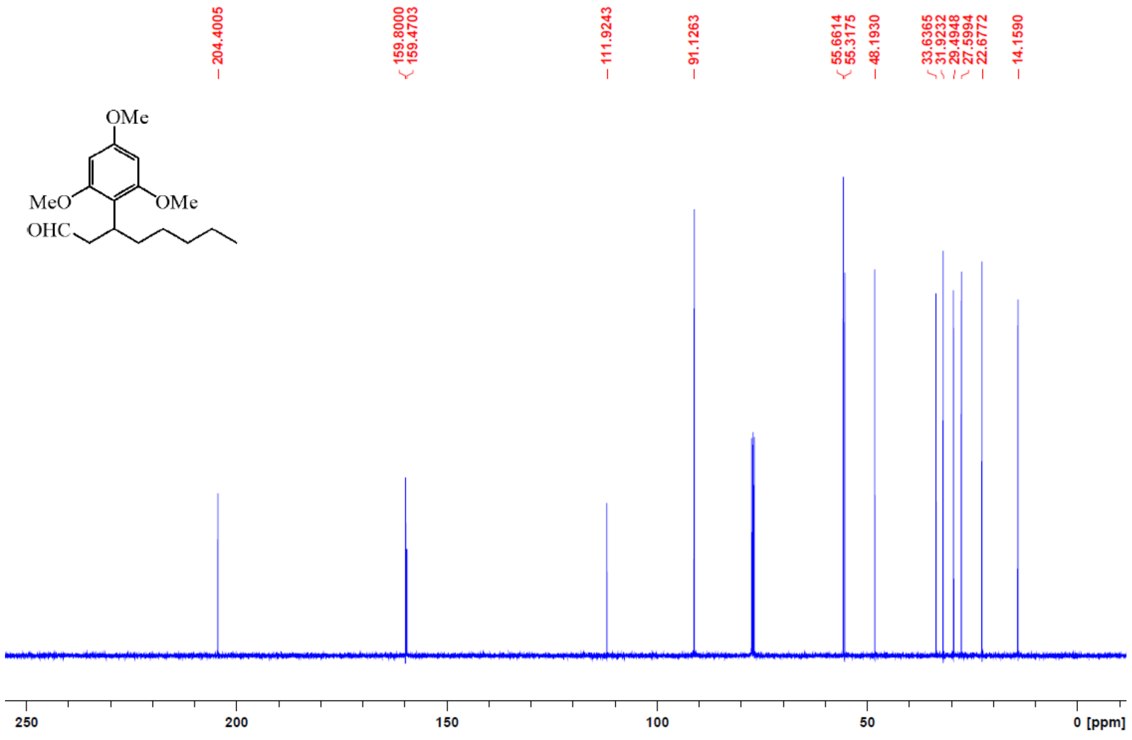

Supplementary Figure 43 ^{13}C NMR ($CDCl_3$, 100 MHz) spectrum of **5**

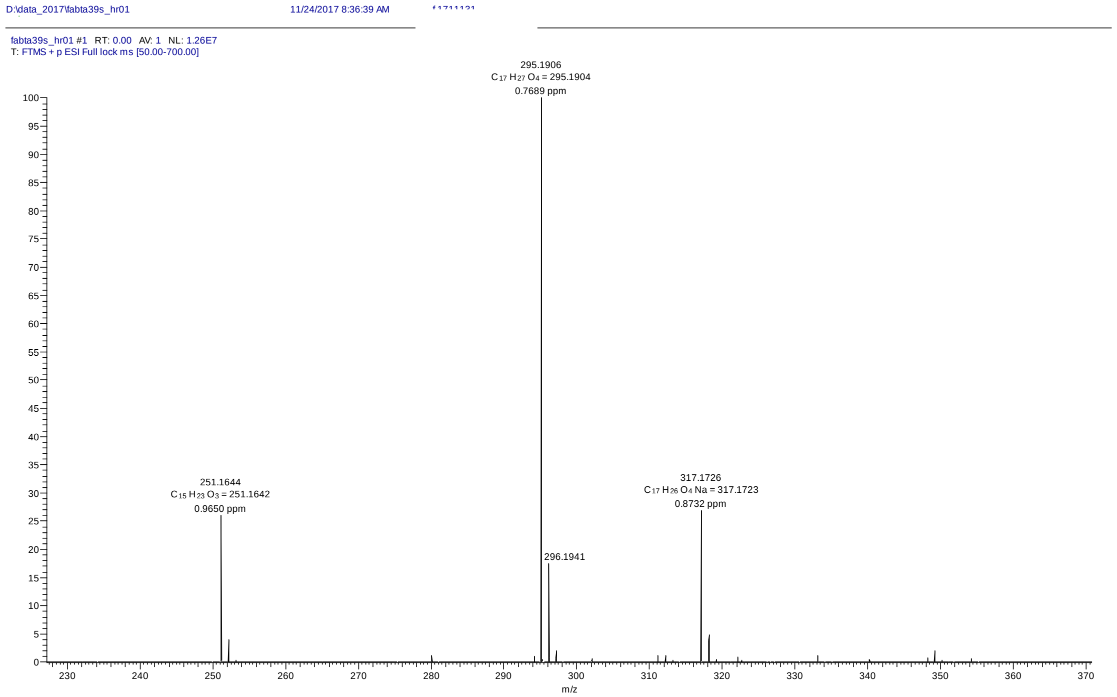

Supplementary Figure 44 ESI-MS spectra of **5**

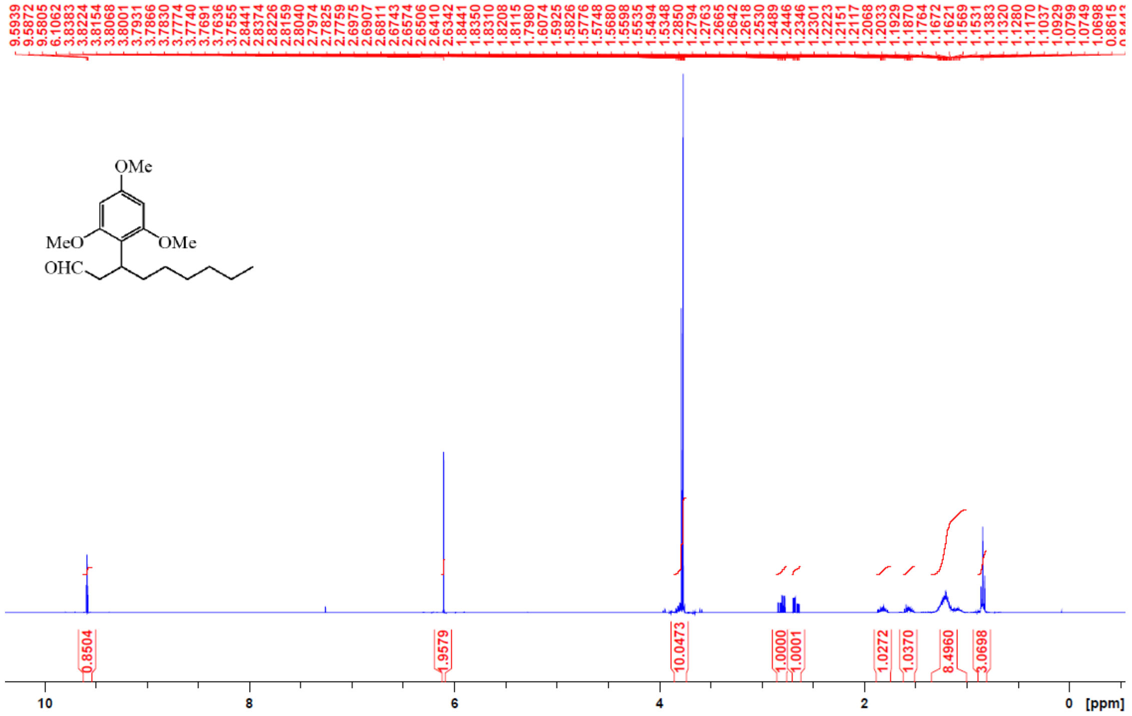

Supplementary Figure 45 ^{1}H NMR ($CDCl_3$, 400 MHz) spectrum of **6**

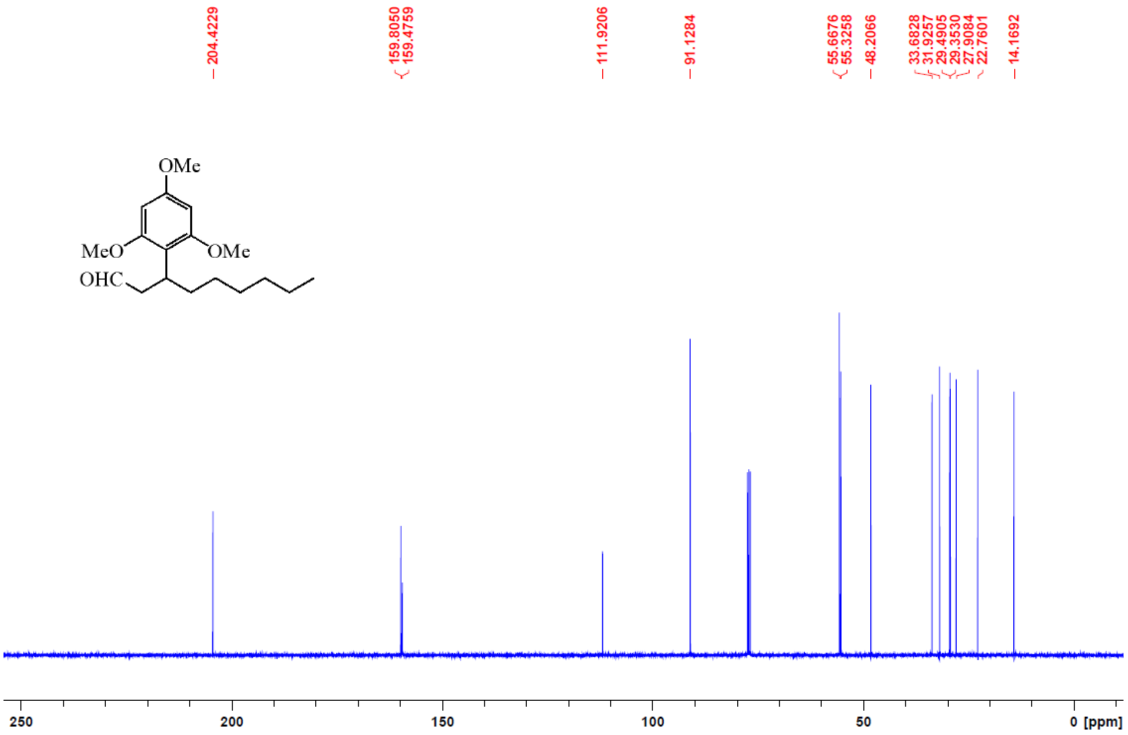

Supplementary Figure 46 ^{13}C NMR ($CDCl_3$, 100 MHz) spectrum of **6**

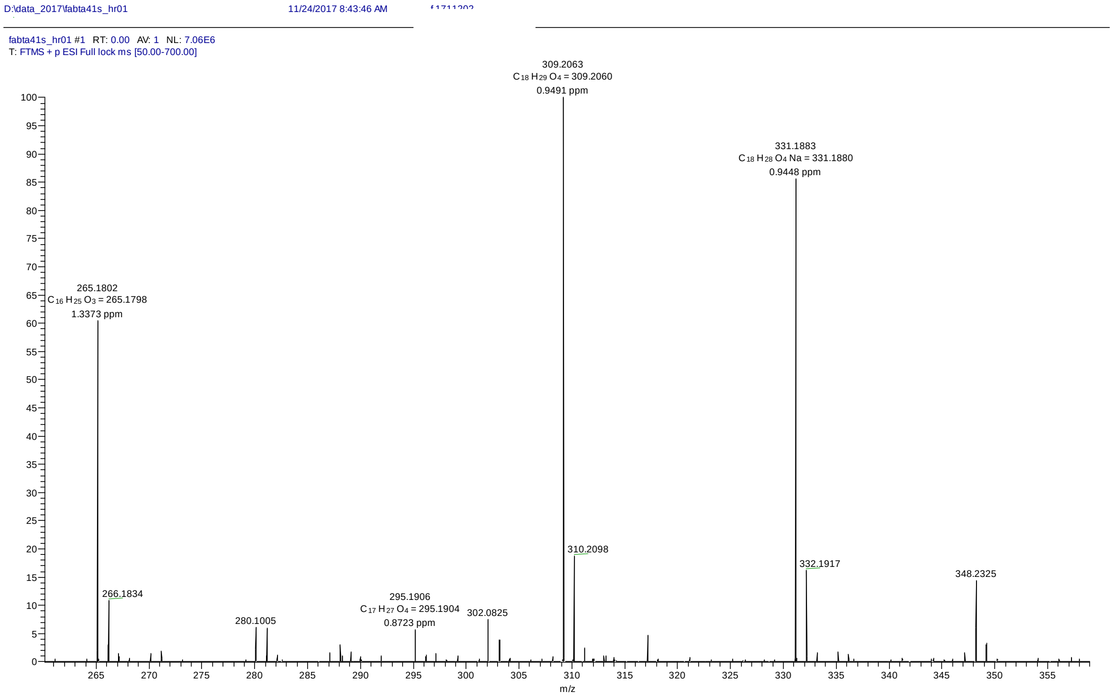

Supplementary Figure 47 ESI-MS spectra of **6**

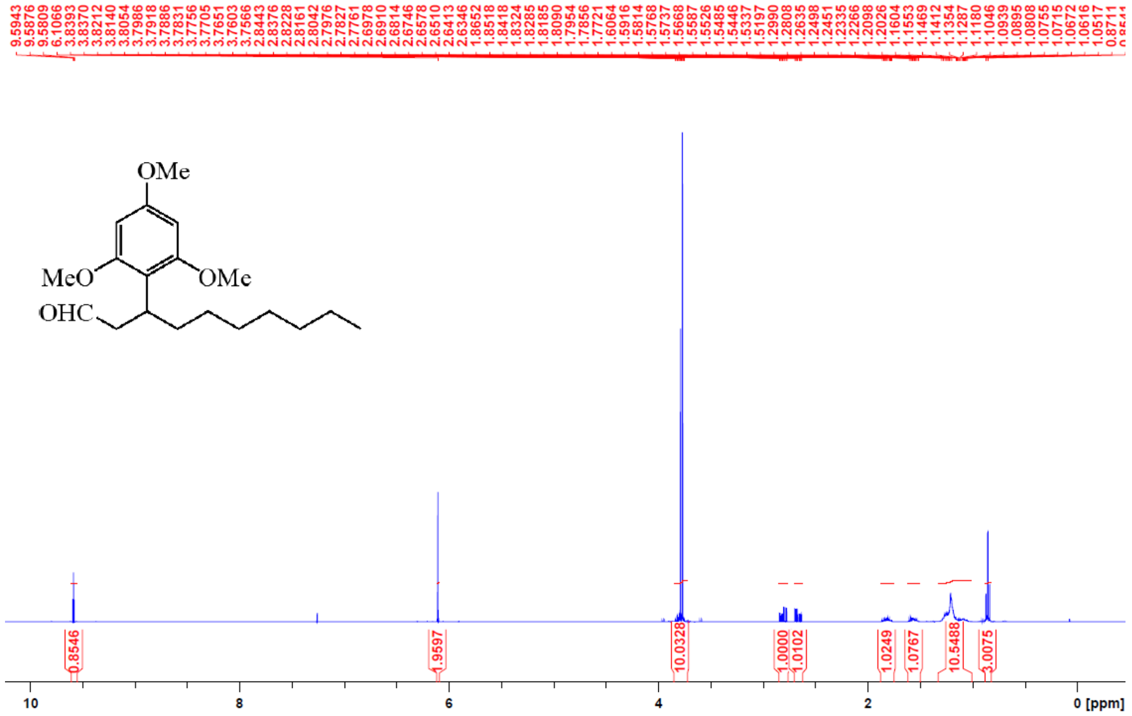

Supplementary Figure 48 ^{1}H NMR ($CDCl_3$, 400 MHz) spectrum of **7**

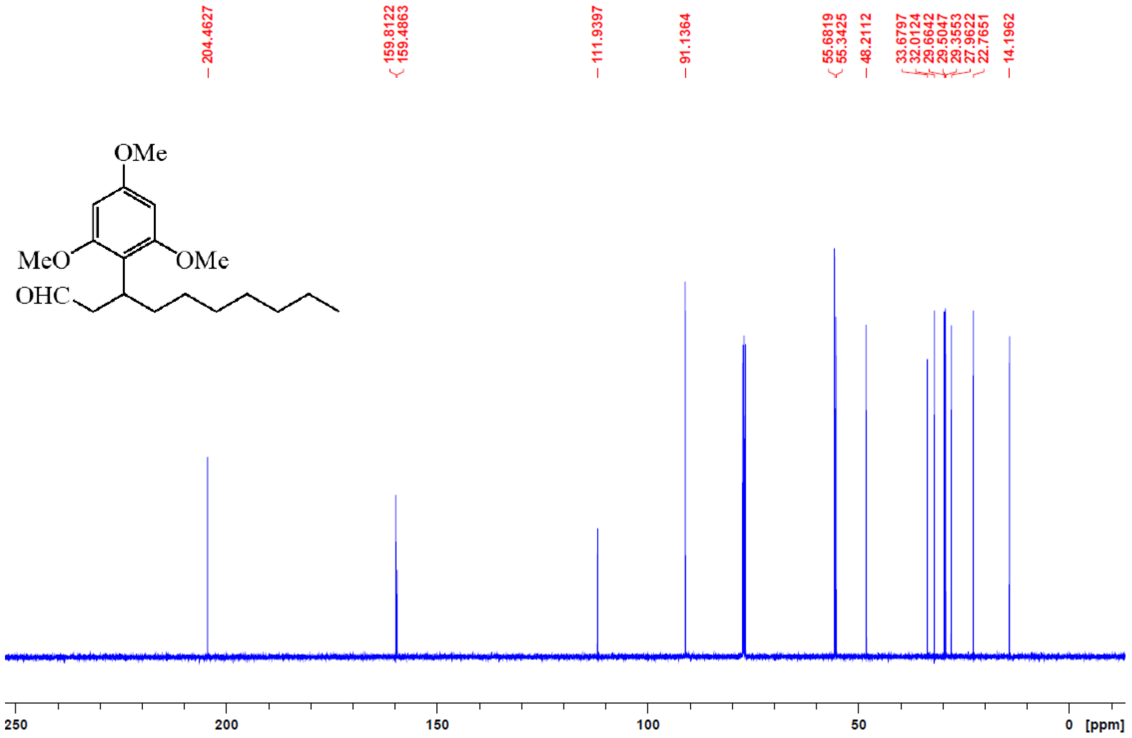

Supplementary Figure 49 ^{13}C NMR ($CDCl_3$, 100 MHz) spectrum of **7**

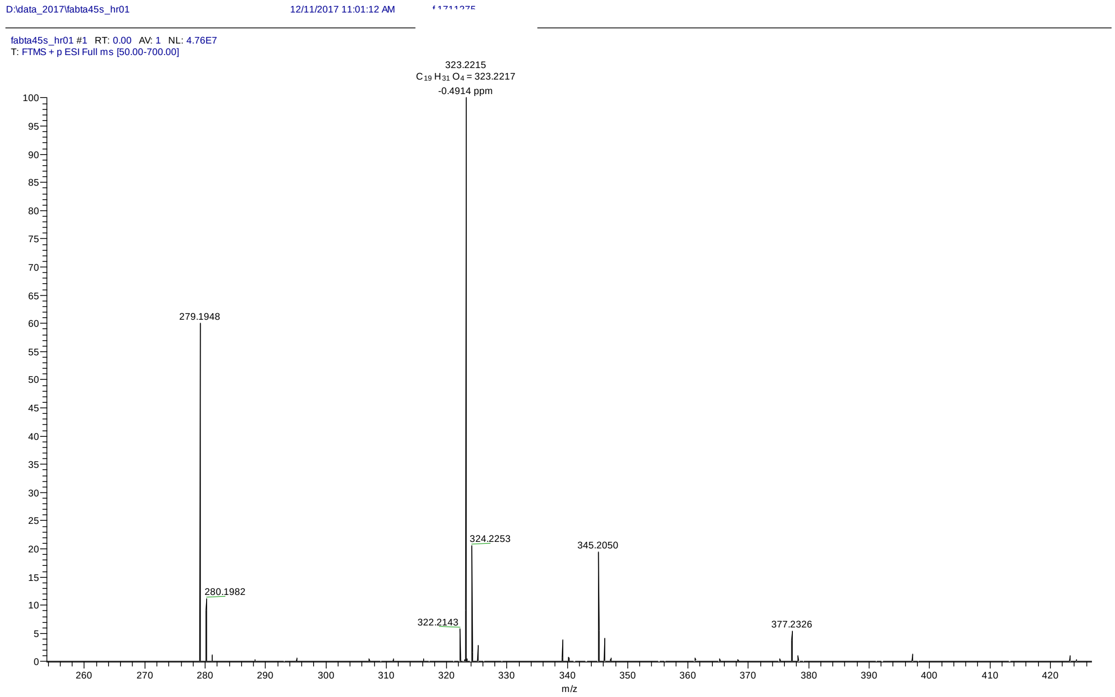

Supplementary Figure 50 ESI-MS spectra of **7**

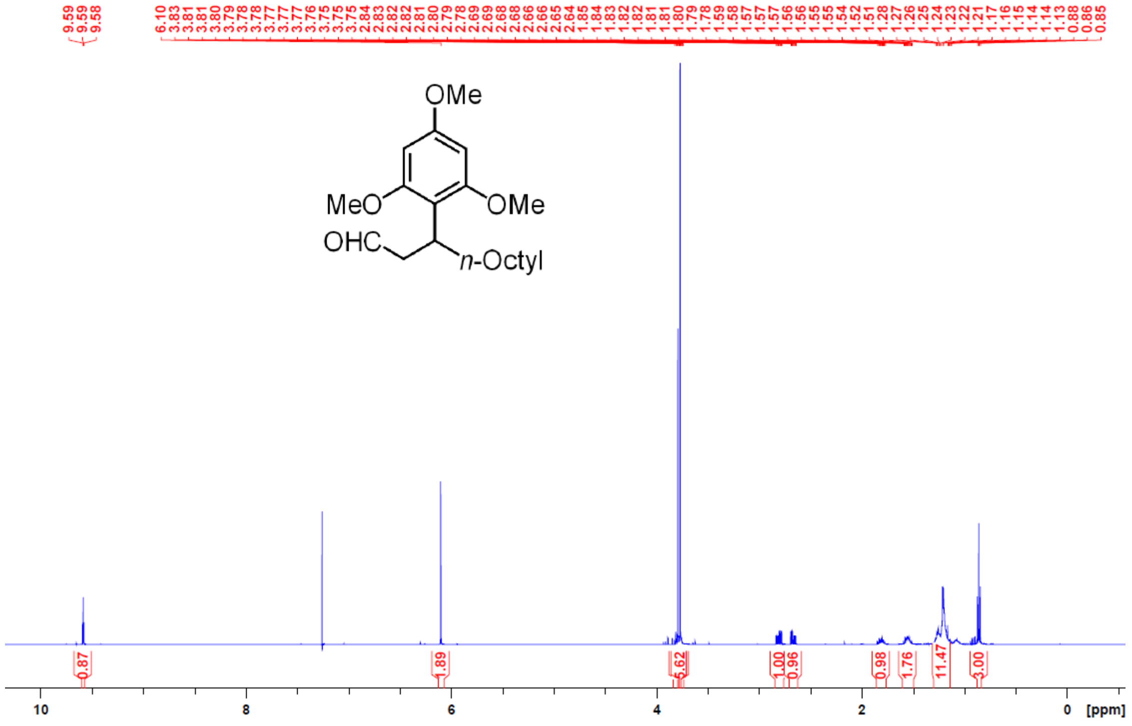

Supplementary Figure 51 ^{1}H NMR ($CDCl_3$, 500 MHz) spectrum of **8**

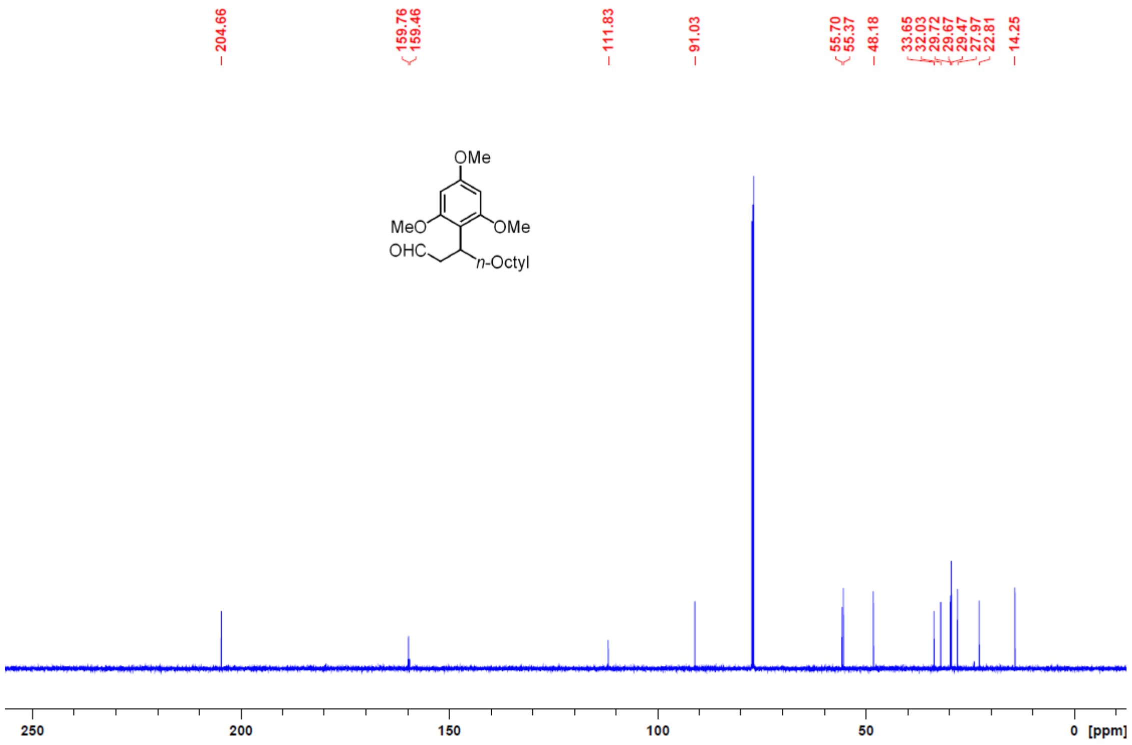

Supplementary Figure 52 ^{13}C NMR ($CDCl_3$, 126 MHz) spectrum of **8**

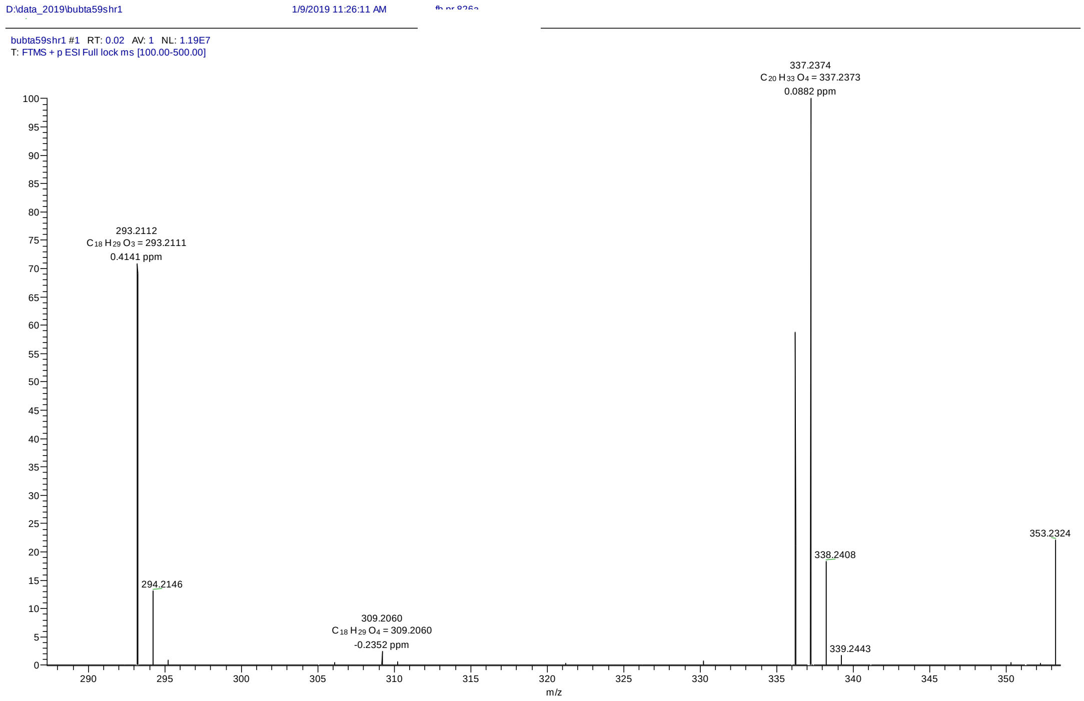

Supplementary Figure 53 ESI-MS spectra of **8**

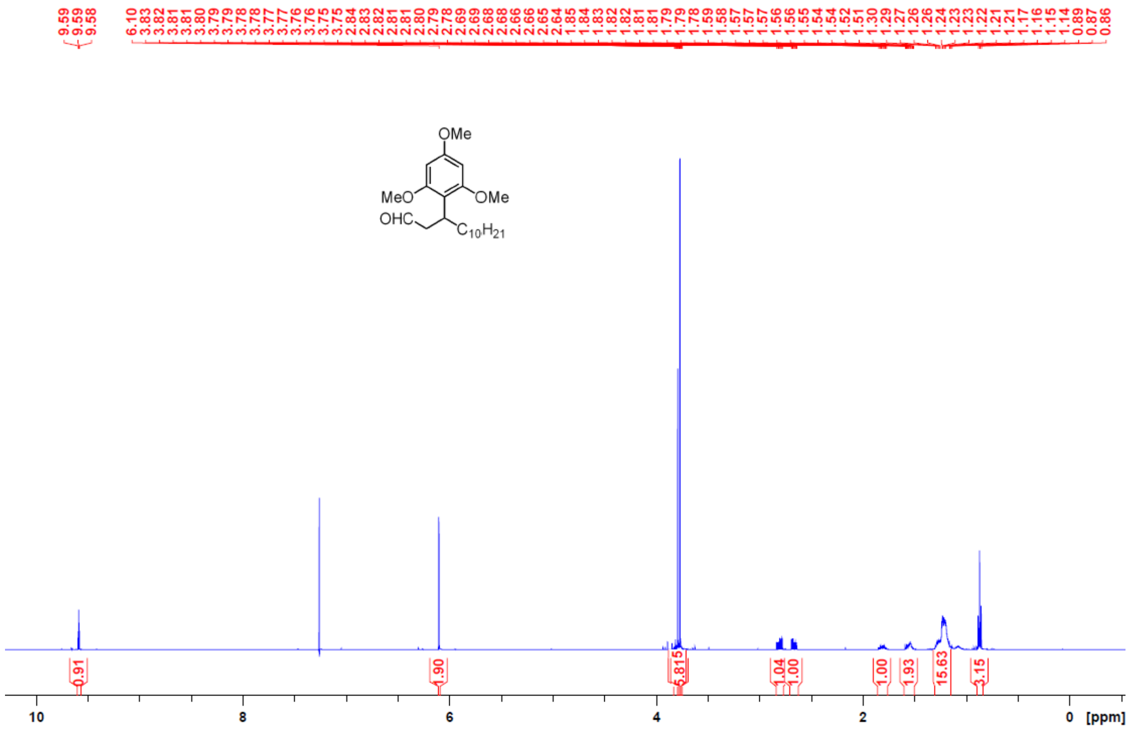

Supplementary Figure 54 ^{1}H NMR ($CDCl_3$, 500 MHz) spectrum of **9**

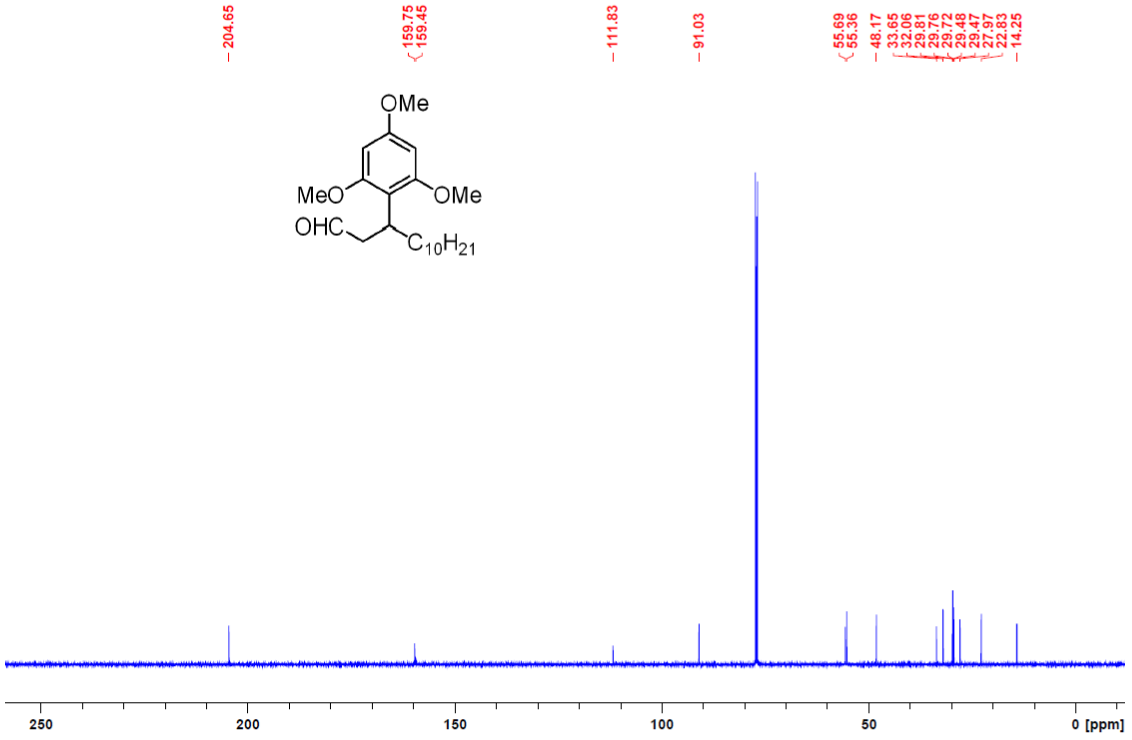

Supplementary Figure 55 ^{13}C NMR ($CDCl_3$, 126 MHz) spectrum of **9**

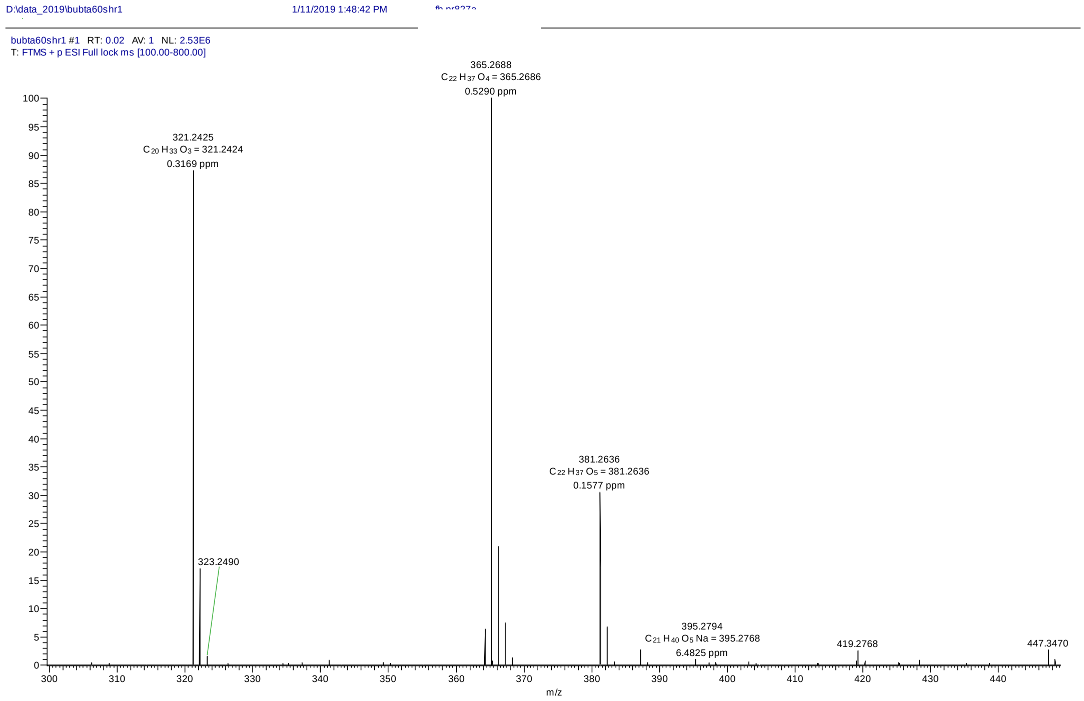

Supplementary Figure 56 ESI-MS spectra of **9**

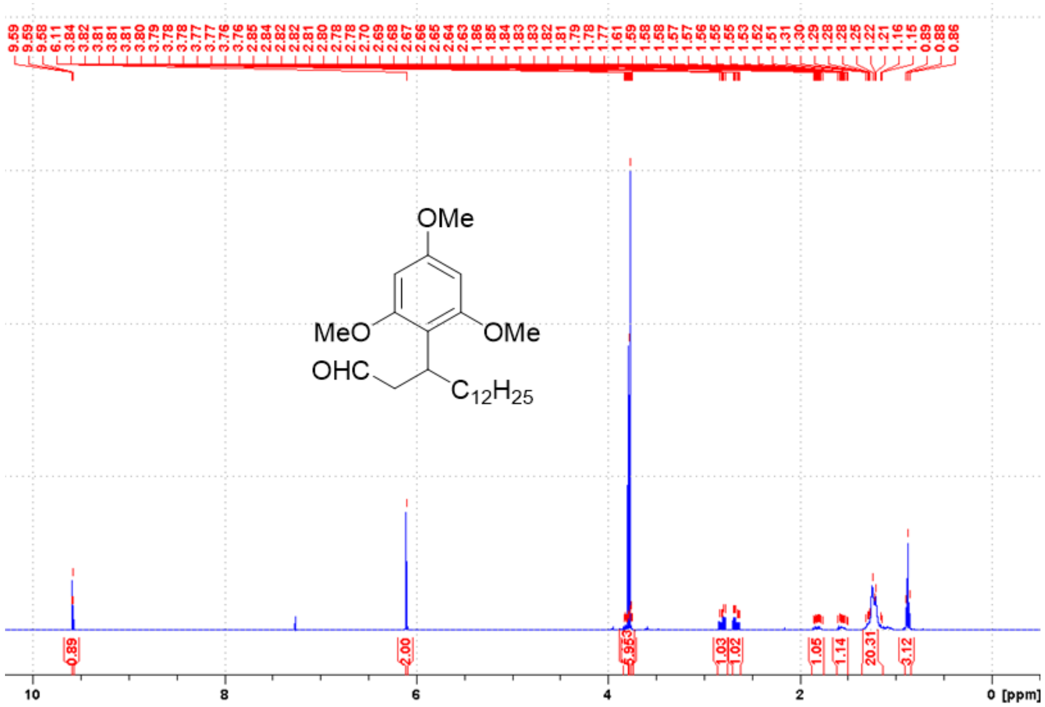

Supplementary Figure 57 ^{1}H NMR ($CDCl_3$, 400 MHz) spectrum of **10**

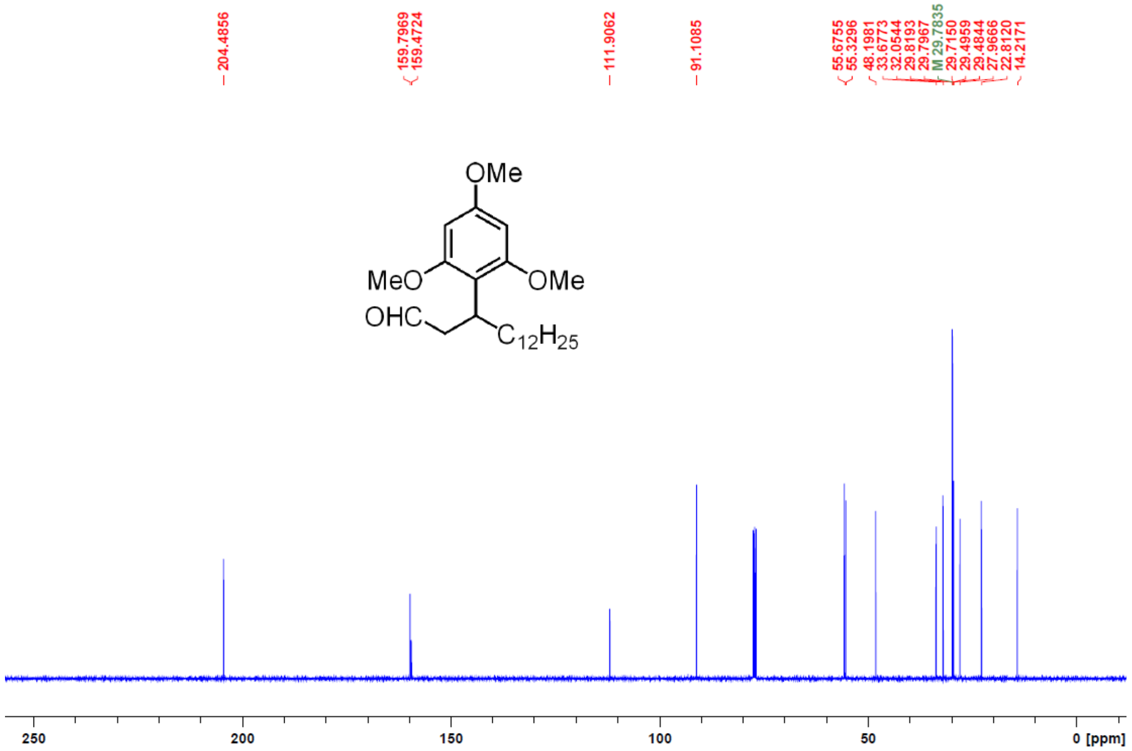

Supplementary Figure 58 ^{13}C NMR ($CDCl_3$, 100 MHz) spectrum of **10**

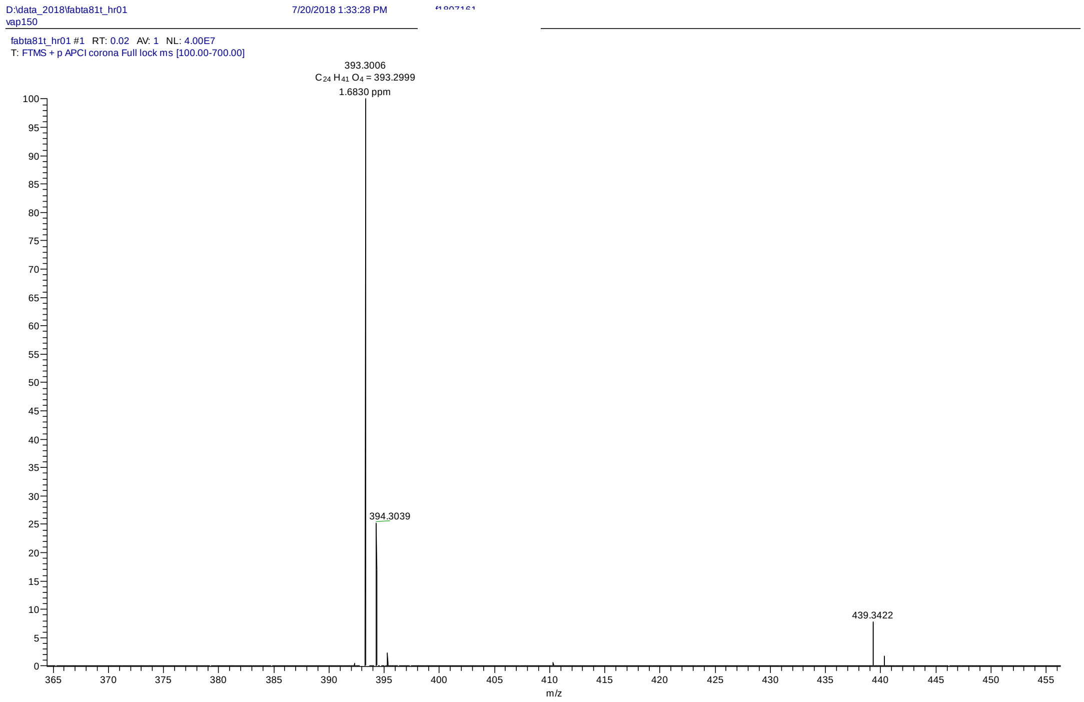

Supplementary Figure 59 ESI-MS spectra of **10**

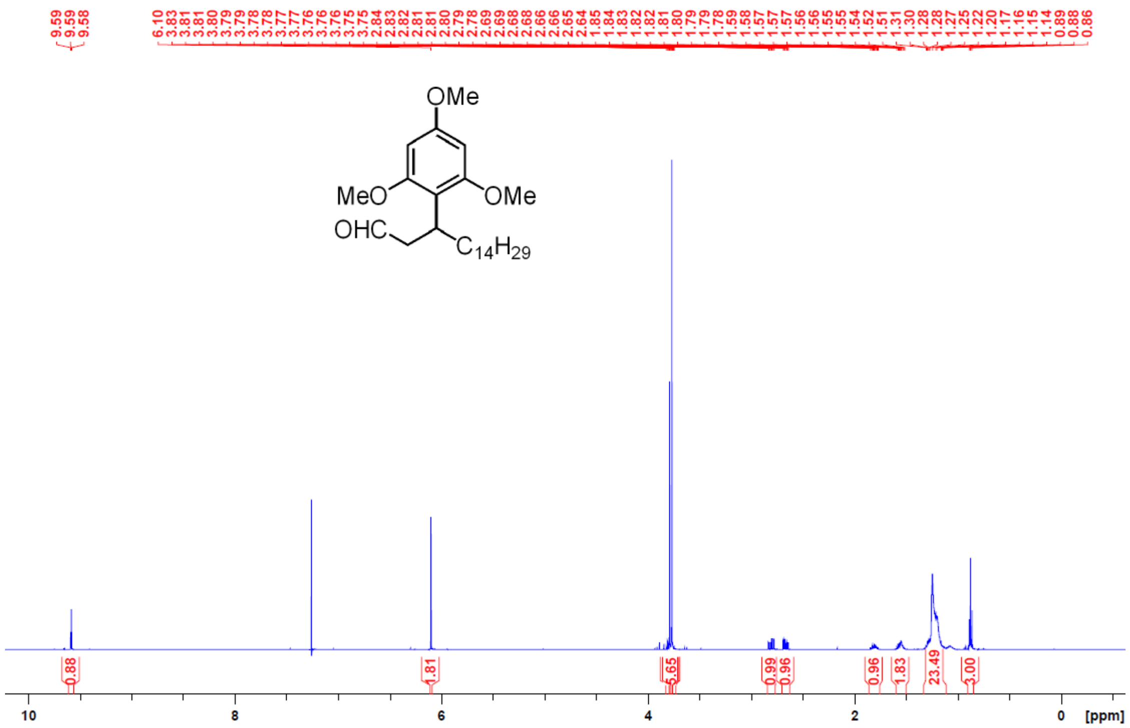

Supplementary Figure 60 ^{1}H NMR ($CDCl_3$, 500 MHz) spectrum of **11**

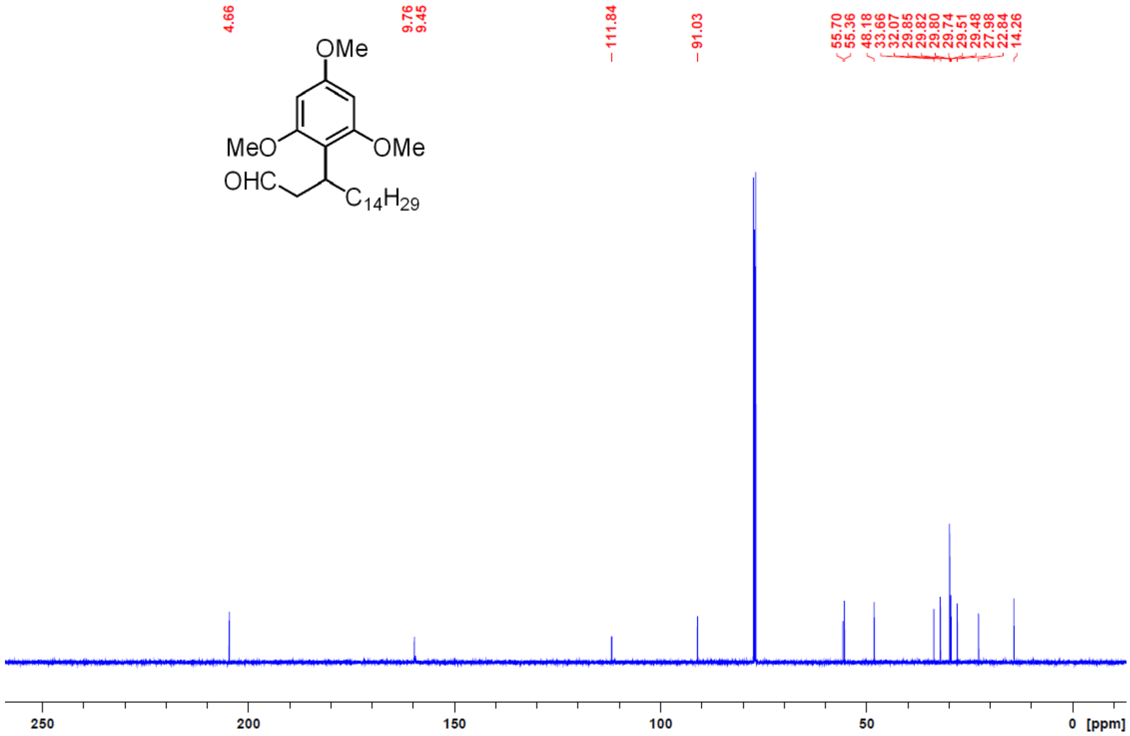

Supplementary Figure 61 ^{13}C NMR ($CDCl_3$, 126 MHz) spectrum of **11**

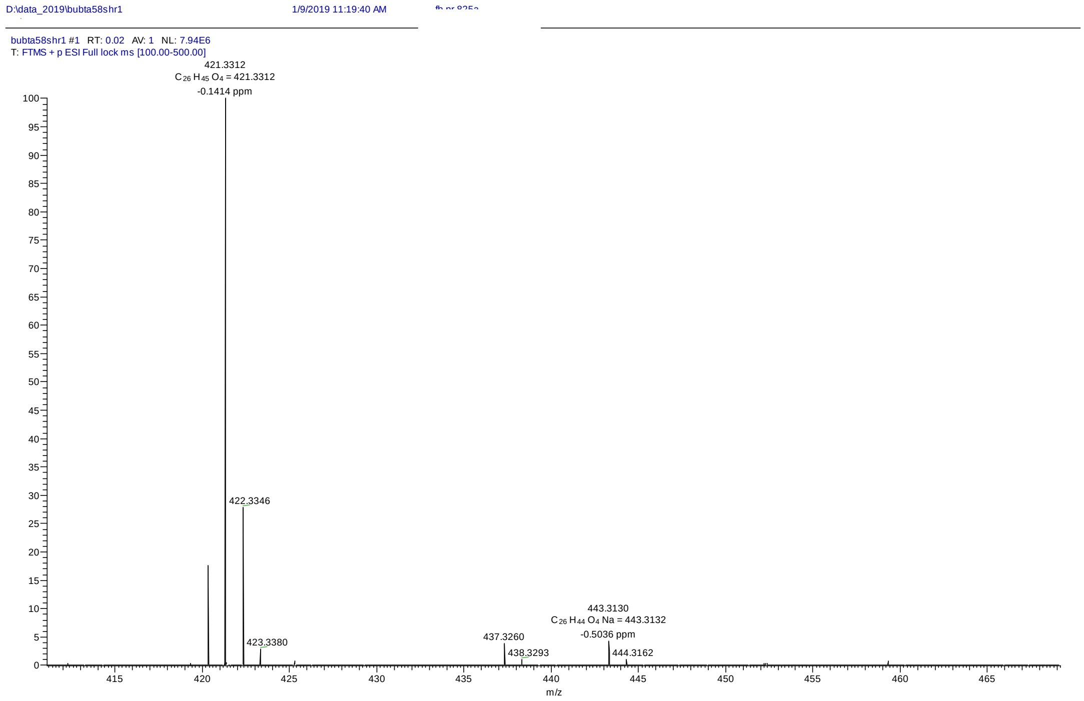

Supplementary Figure 62 ESI-MS spectra of **11**

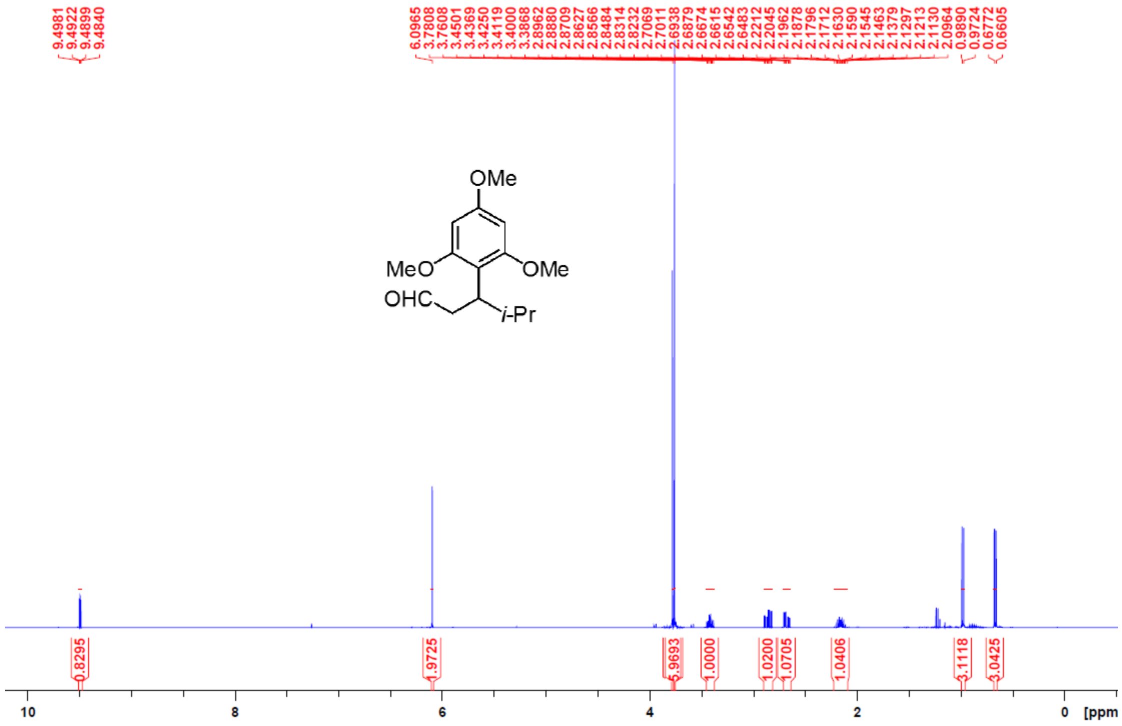

Supplementary Figure 63 ^{1}H NMR ($CDCl_3$, 400 MHz) spectrum of **12**

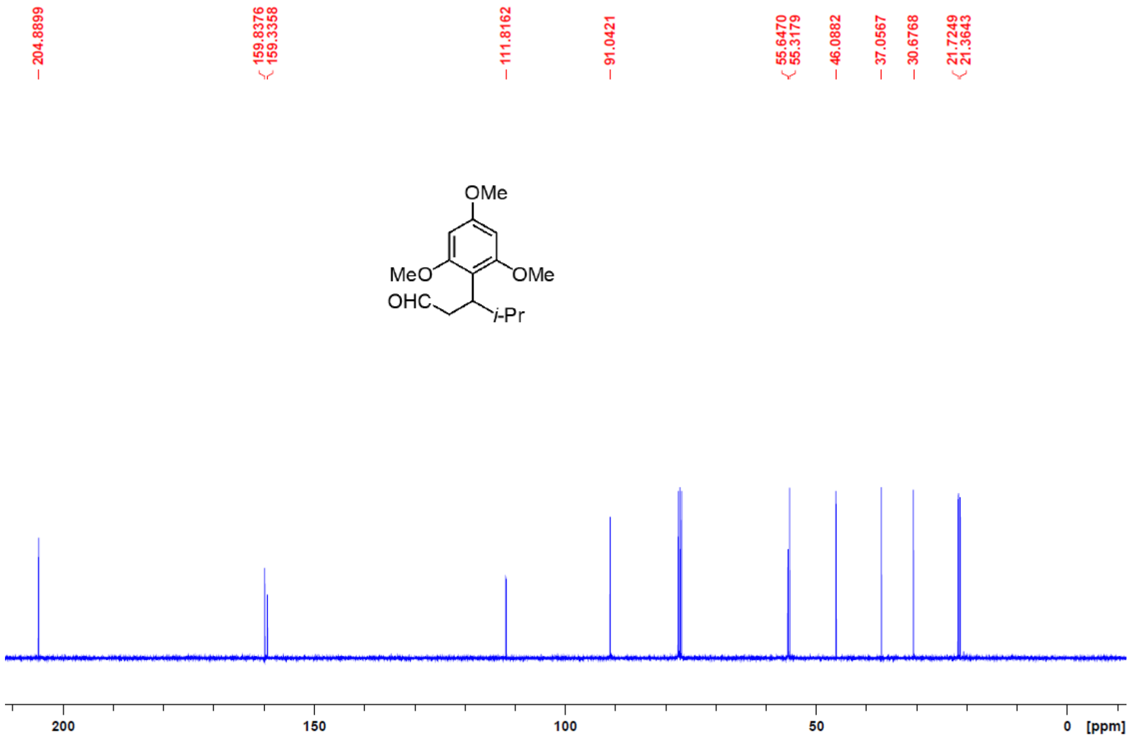

Supplementary Figure 64 ^{13}C NMR ($CDCl_3$, 100 MHz) spectrum of **12**

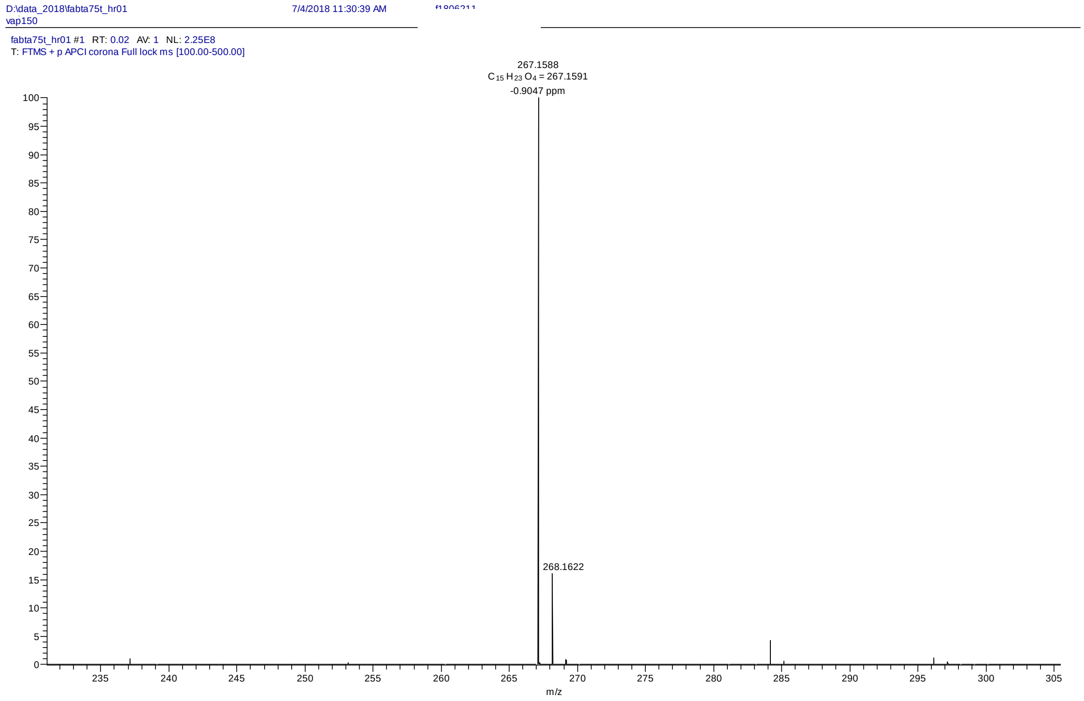

Supplementary Figure 65 ESI-MS spectra of **12**

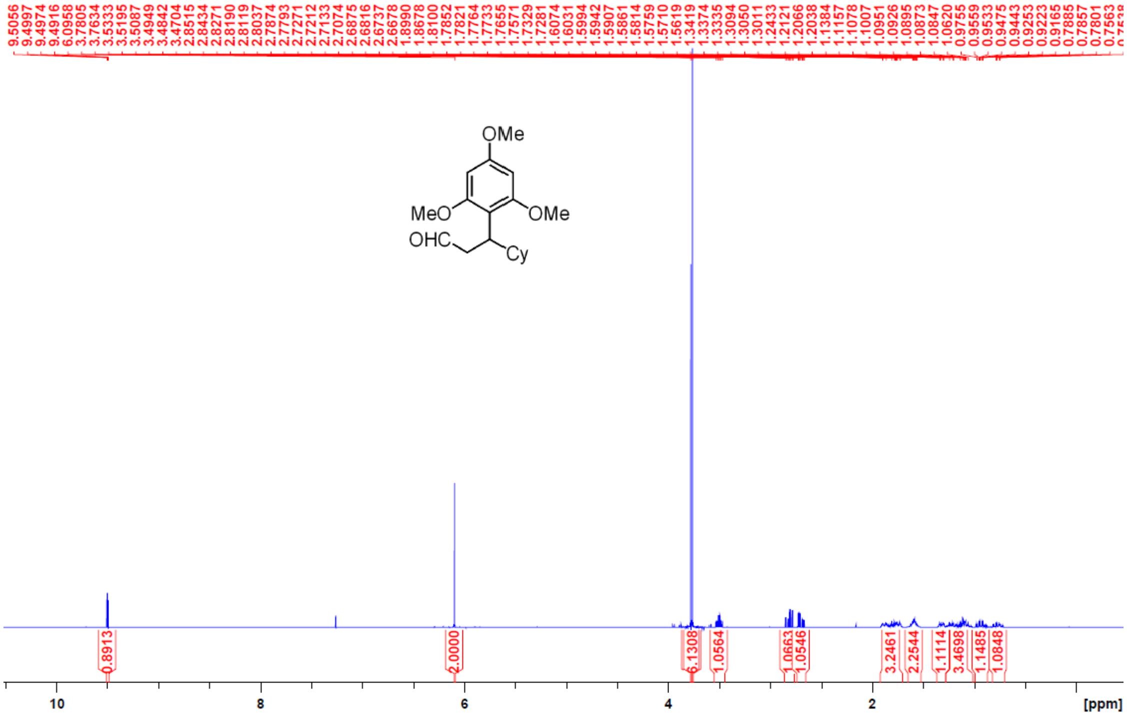

Supplementary Figure 66 ^{1}H NMR ($CDCl_3$, 400 MHz) spectrum of **13**

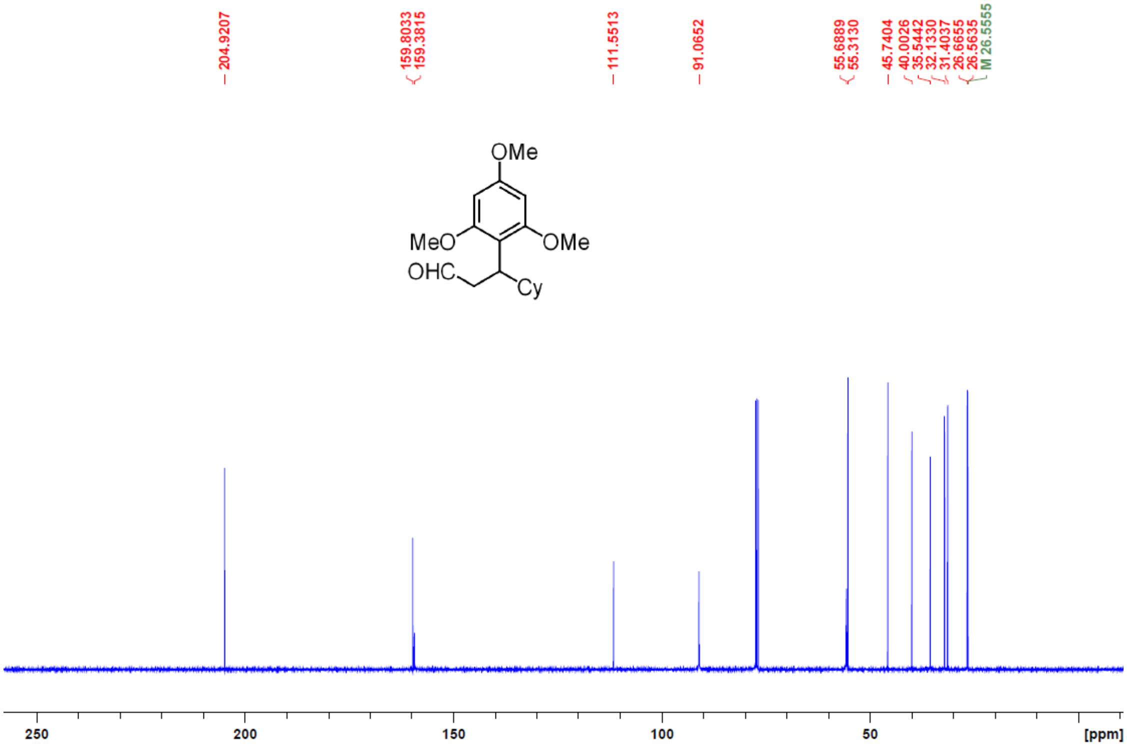

Supplementary Figure 67 ^{13}C NMR ($CDCl_3$, 100 MHz) spectrum of **13**

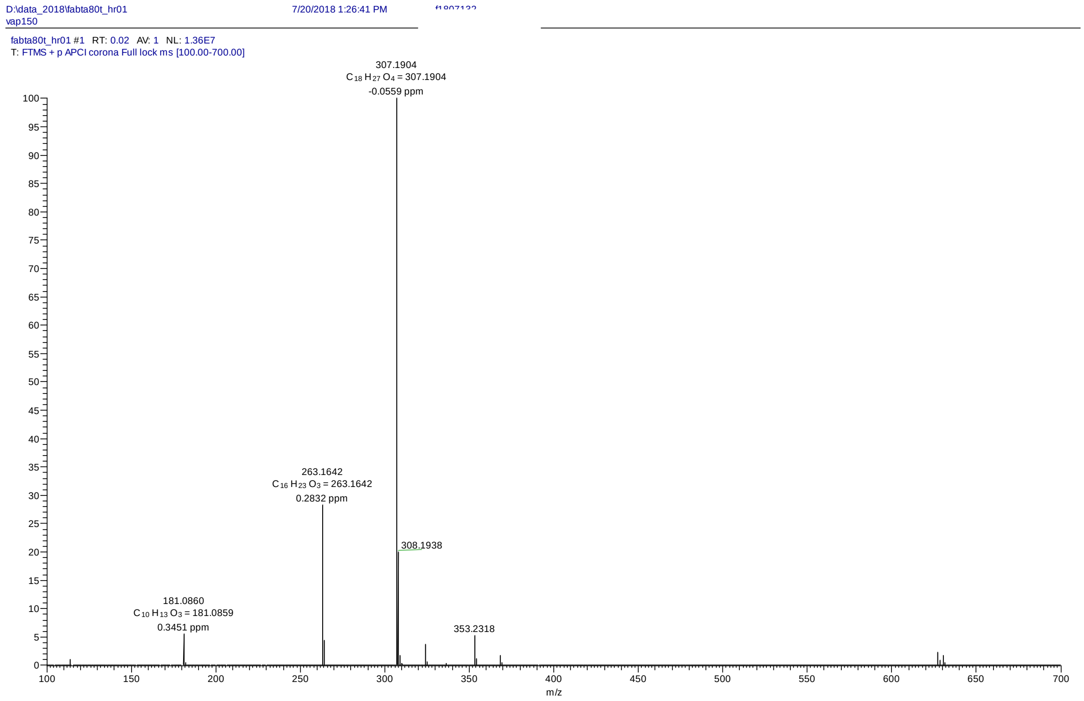

Supplementary Figure 68 ESI-MS spectra of **13**

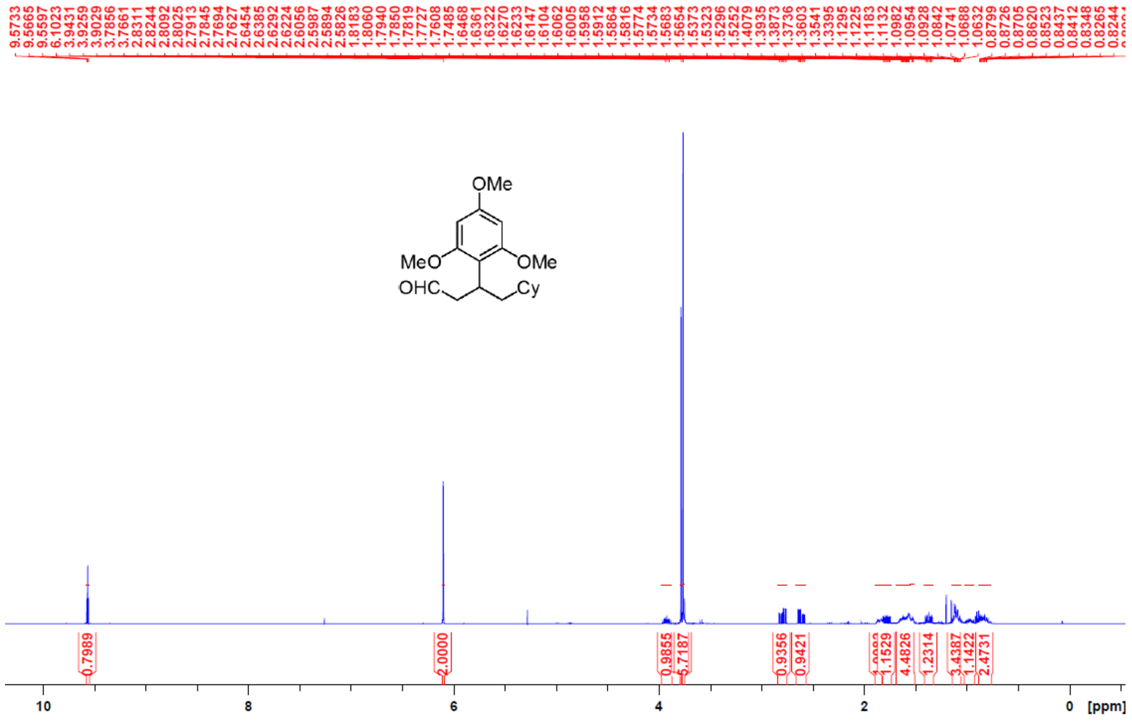

Supplementary Figure 69 ^{1}H NMR ($CDCl_3$, 400 MHz) spectrum of **14**

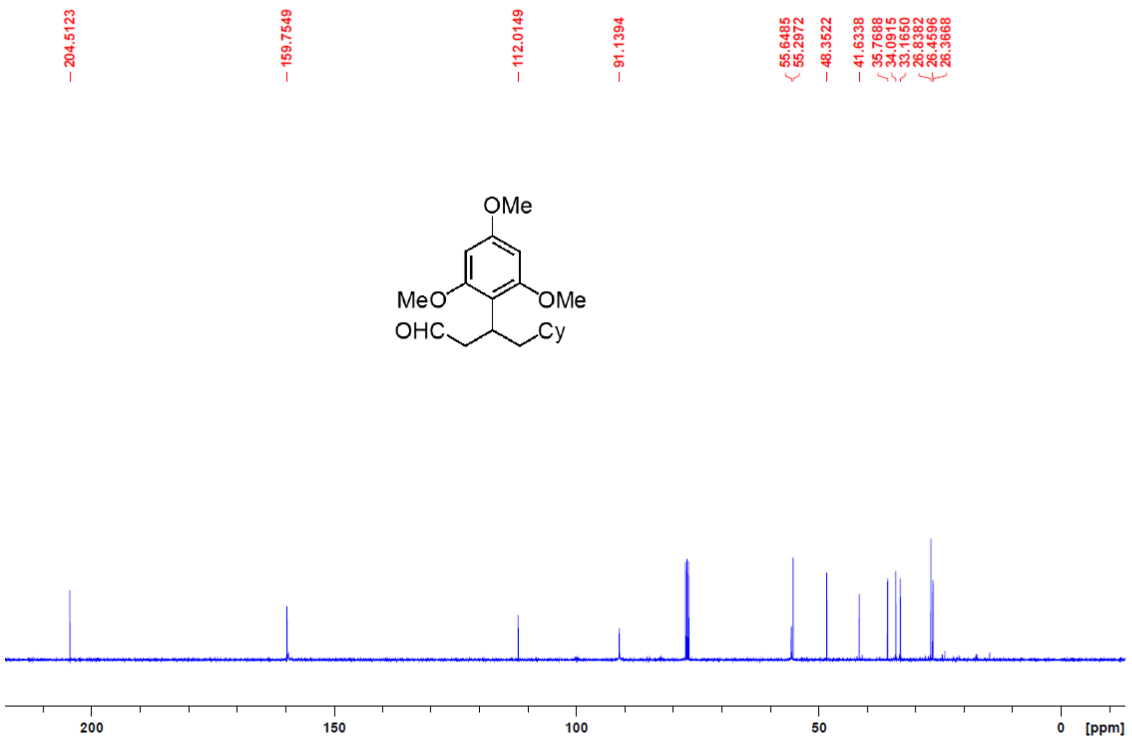

Supplementary Figure 70 ^{13}C NMR ($CDCl_3$, 100 MHz) spectrum of **14**

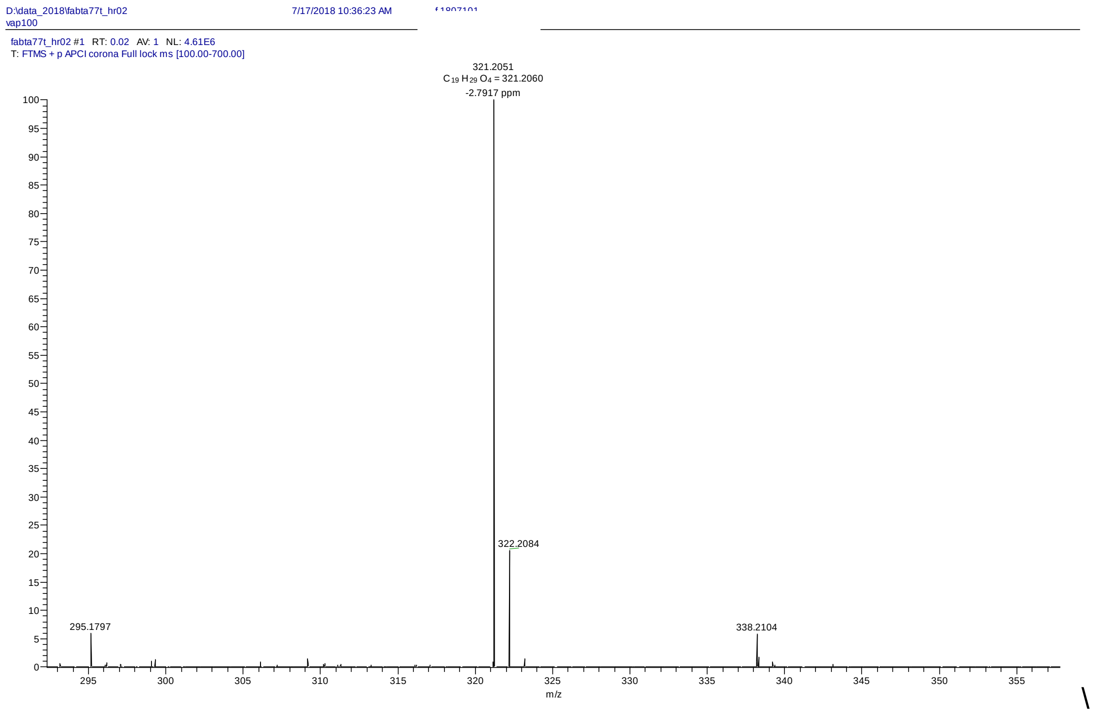

Supplementary Figure 71 ESI-MS spectra of **14**

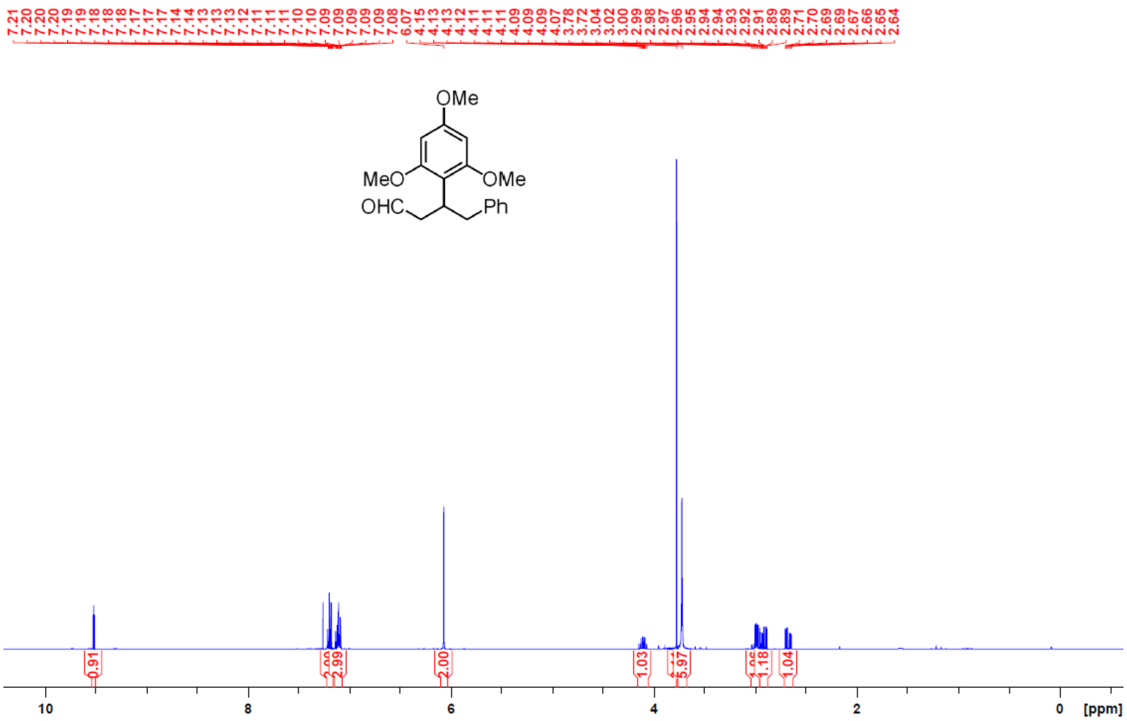

Supplementary Figure 72 ^{1}H NMR ($CDCl_3$, 400 MHz) spectrum of **15**

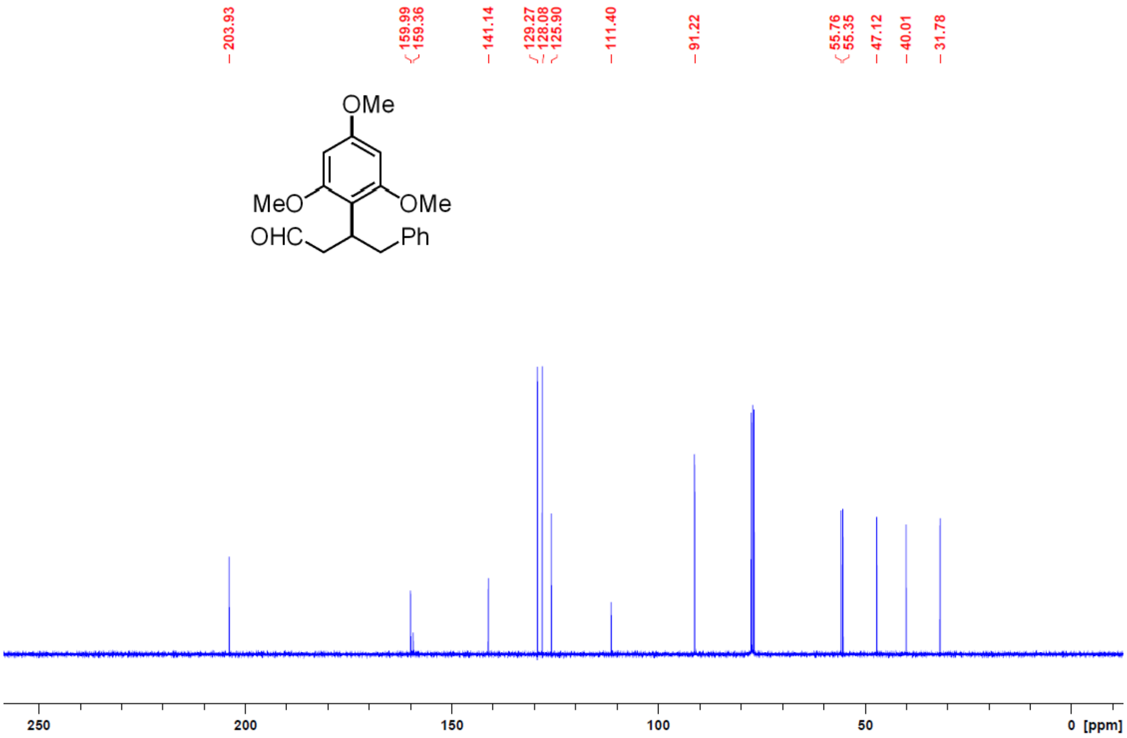

Supplementary Figure 73 ^{13}C NMR ($CDCl_3$, 100 MHz) spectrum of **15**

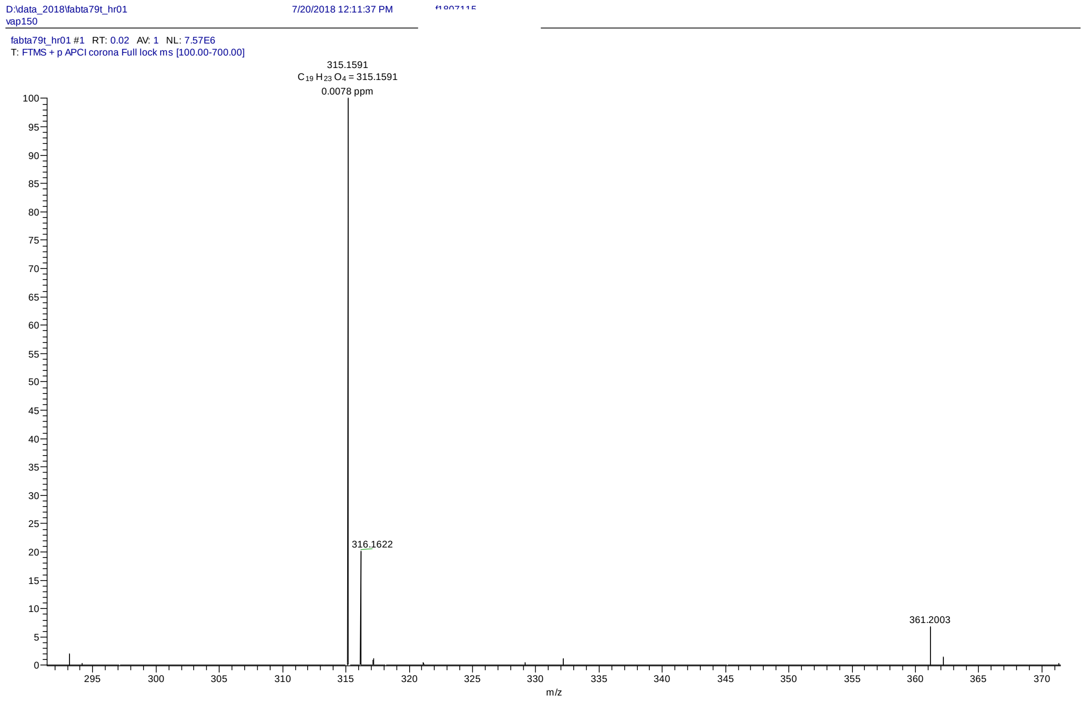

Supplementary Figure 74 ESI-MS spectra of **15**

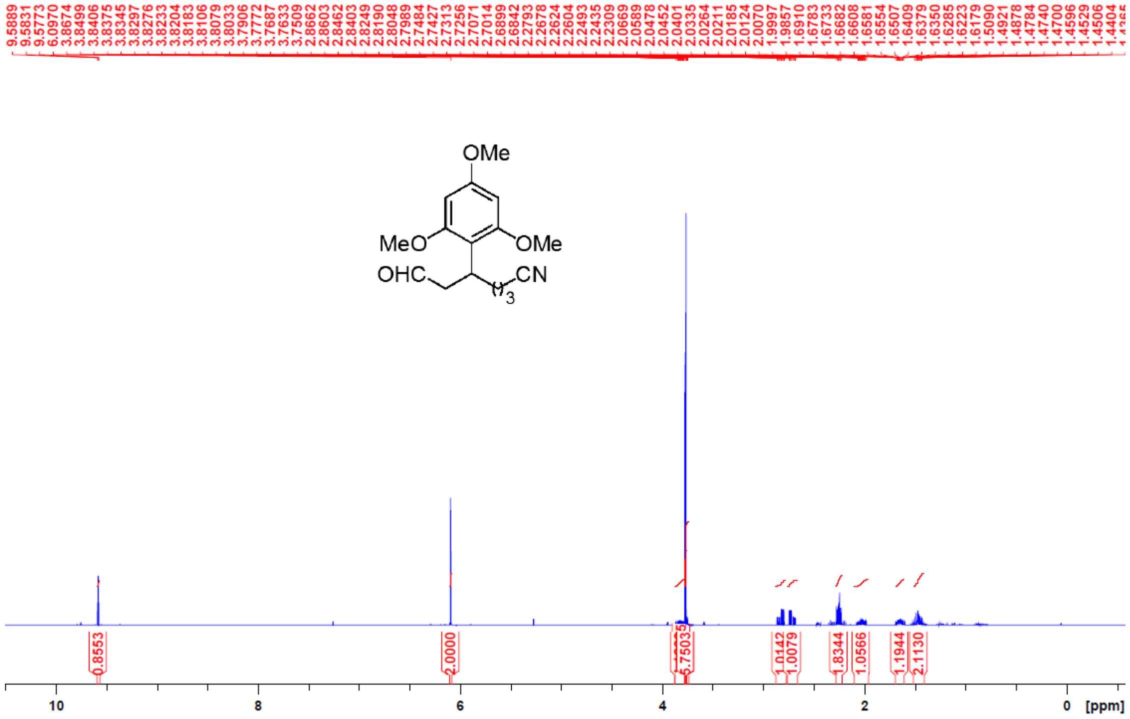

Supplementary Figure 75 ^{1}H NMR ($CDCl_3$, 400 MHz) spectrum of **16**

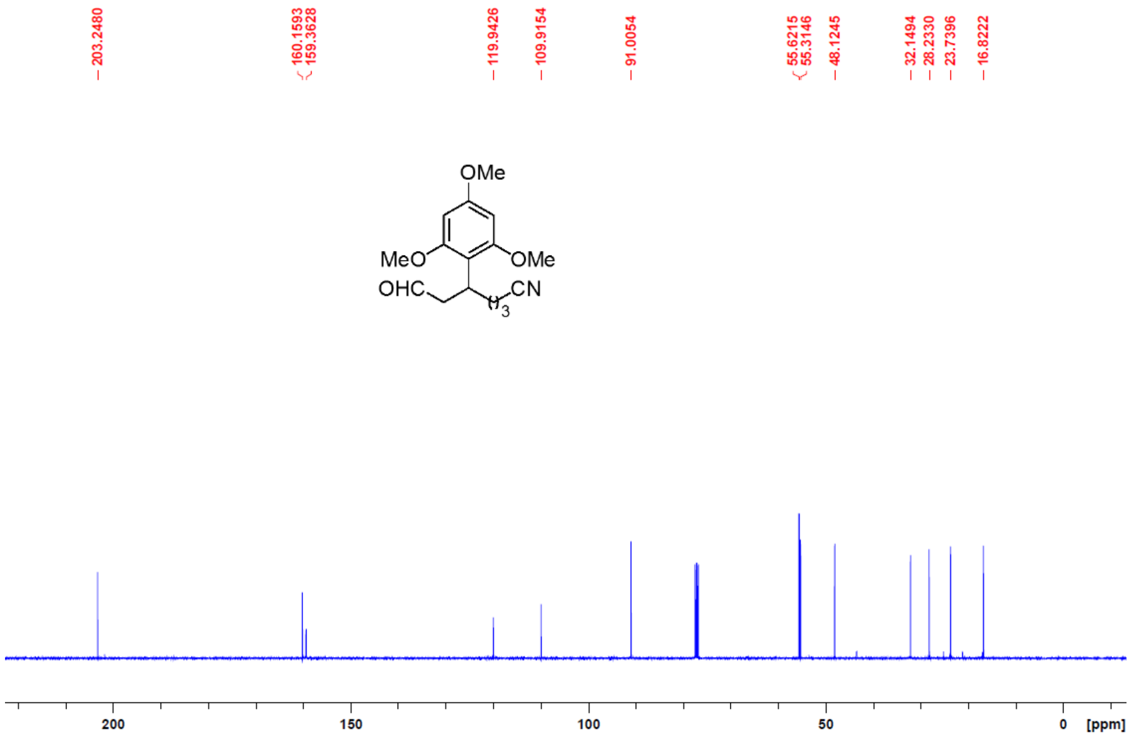

Supplementary Figure 76 ^{13}C NMR ($CDCl_3$, 100 MHz) spectrum of **16**

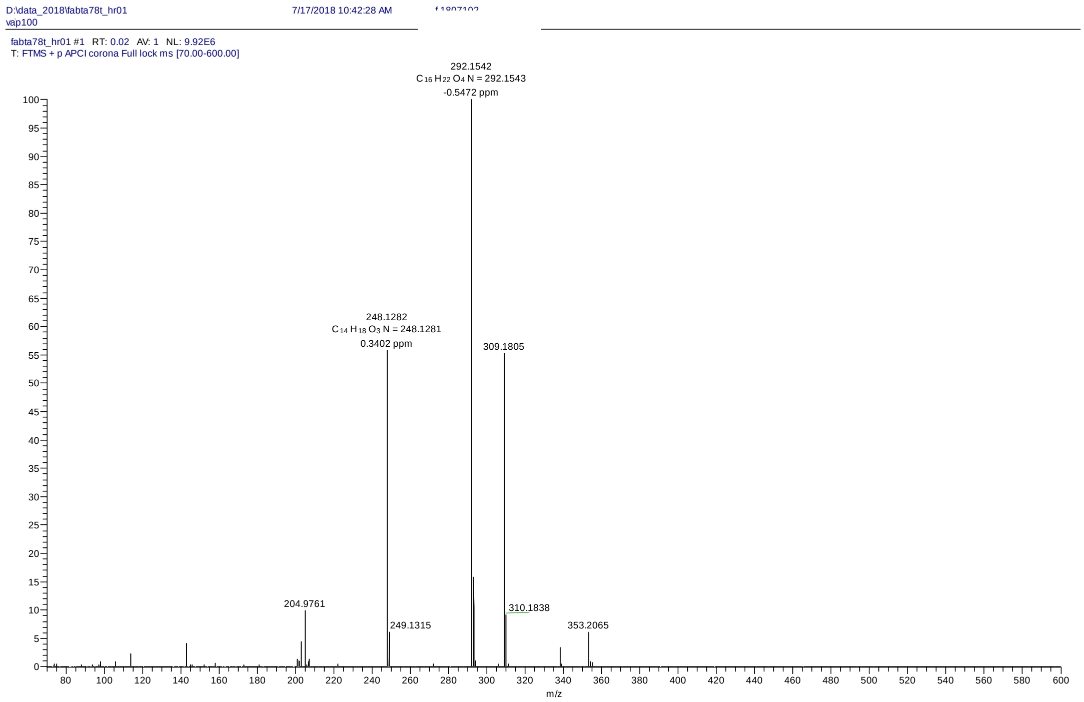

Supplementary Figure 77 ESI-MS spectra of **16**

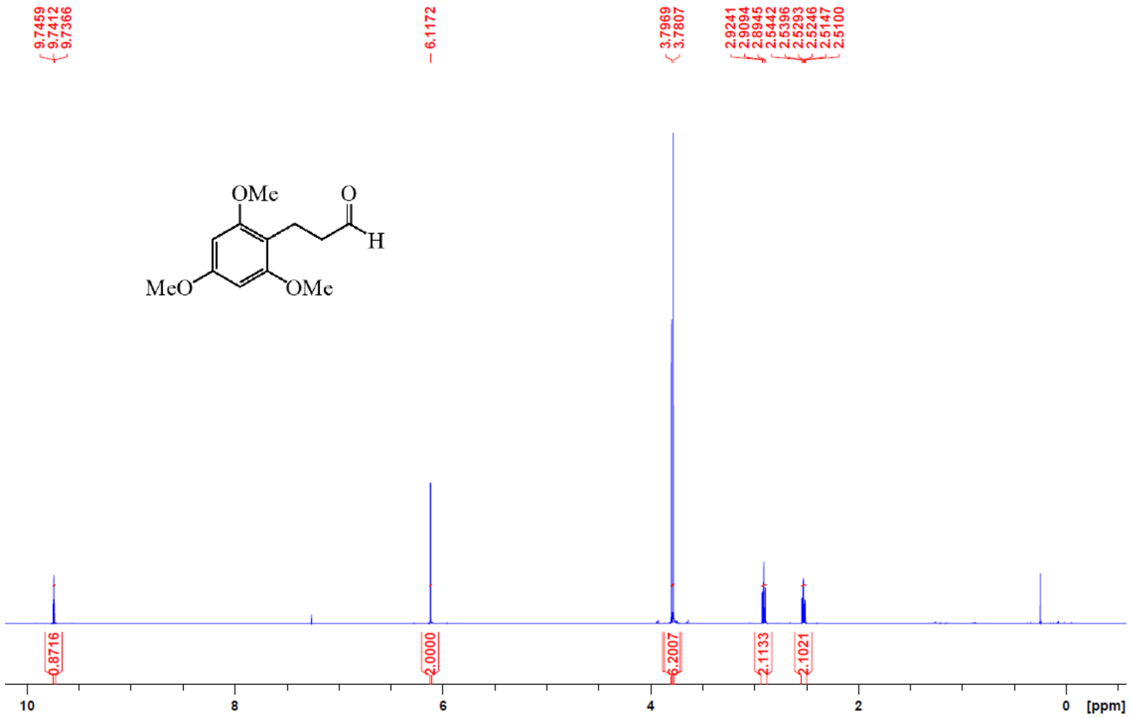

Supplementary Figure 78 ^{1}H NMR ($CDCl_3$, 500 MHz) spectrum of **17**

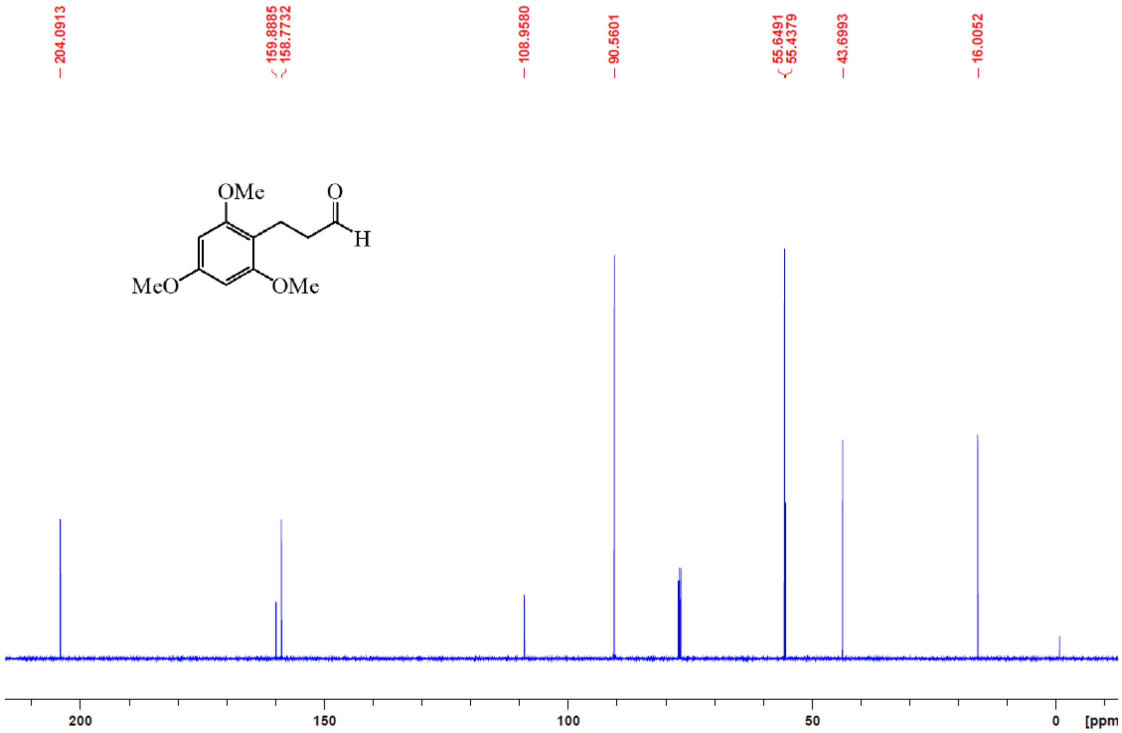

Supplementary Figure 79 ^{13}C NMR ($CDCl_3$, 126 MHz) spectrum of **17**

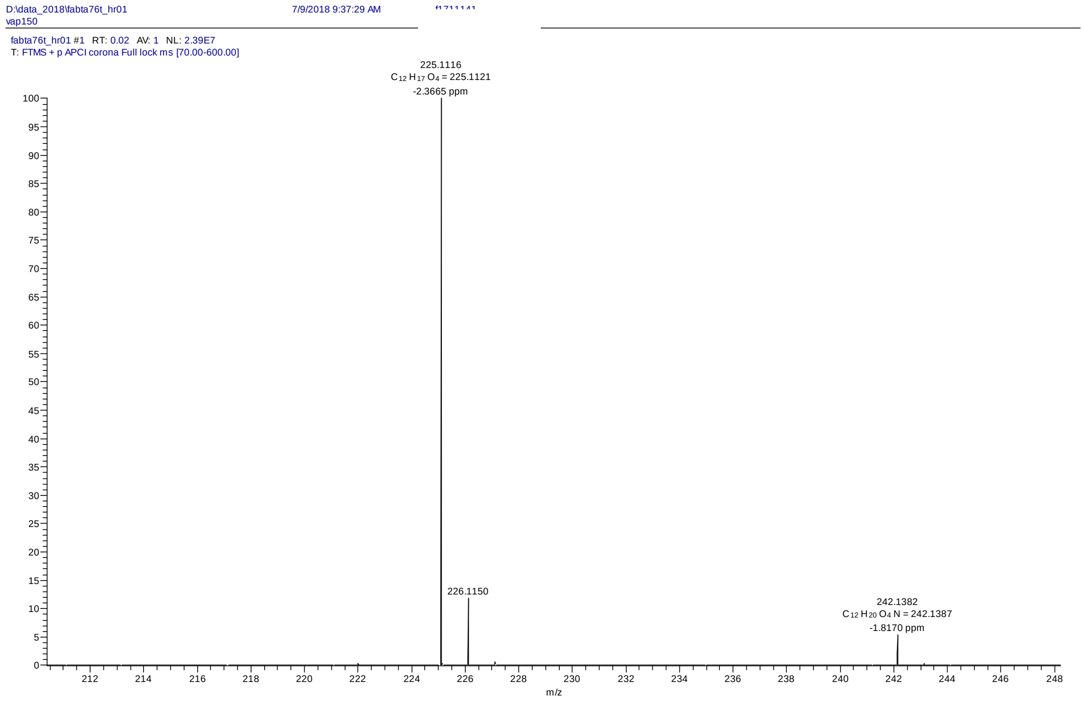

Supplementary Figure 80 ESI-MS spectra of **17**

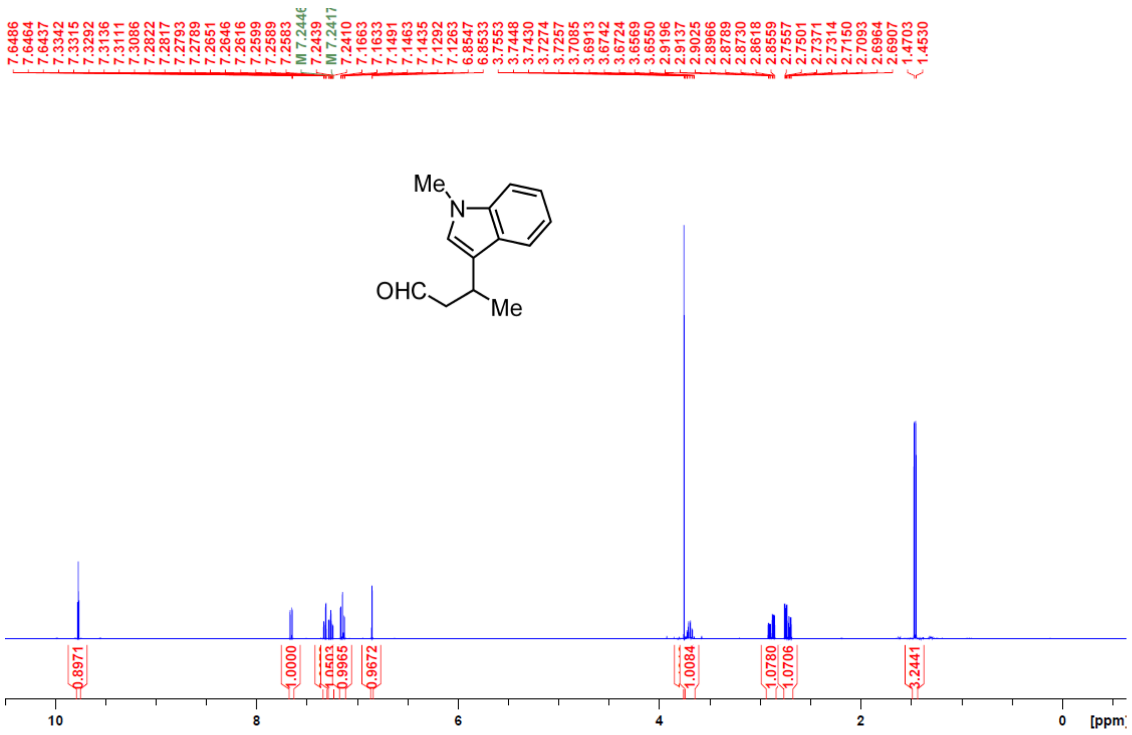

Supplementary Figure 81 ^{1}H NMR ($CDCl_3$, 400 MHz) spectrum of **18**

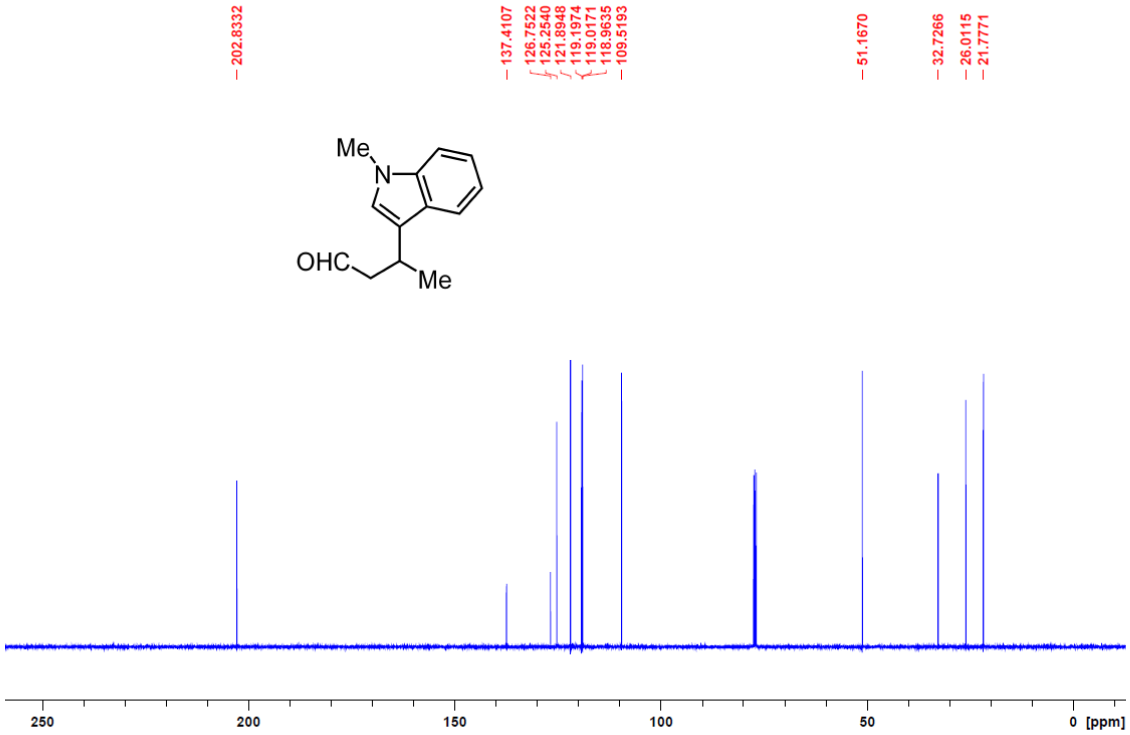

Supplementary Figure 82 ^{13}C NMR ($CDCl_3$, 100 MHz) spectrum of **18**

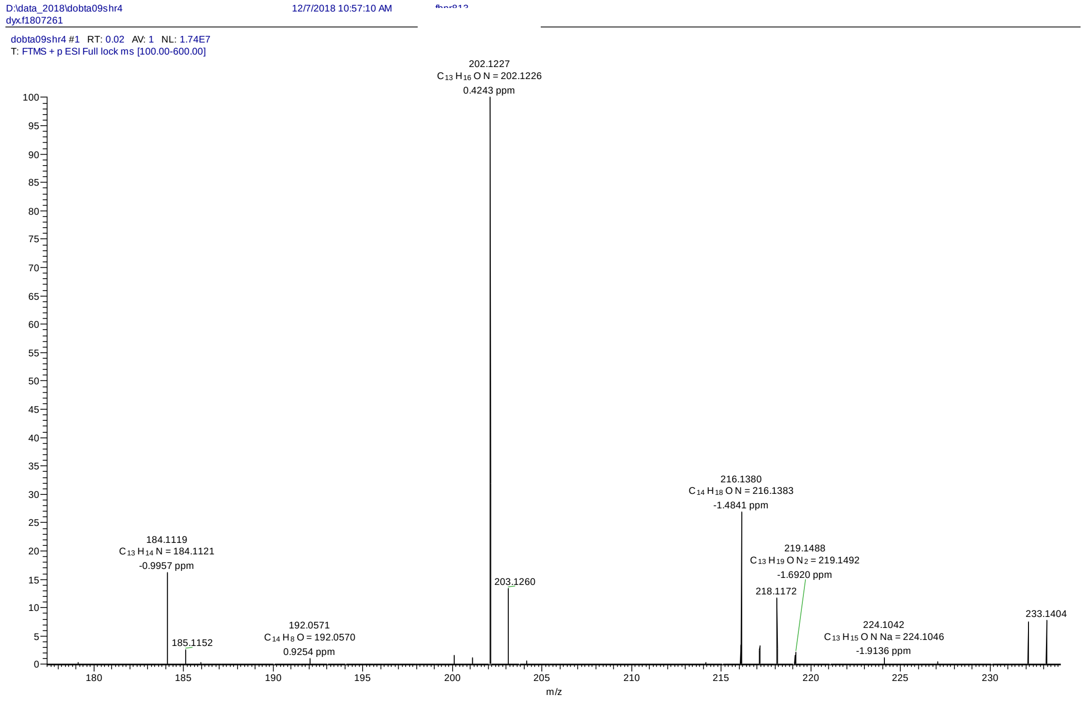

Supplementary Figure 83 ESI-MS spectra of **18**

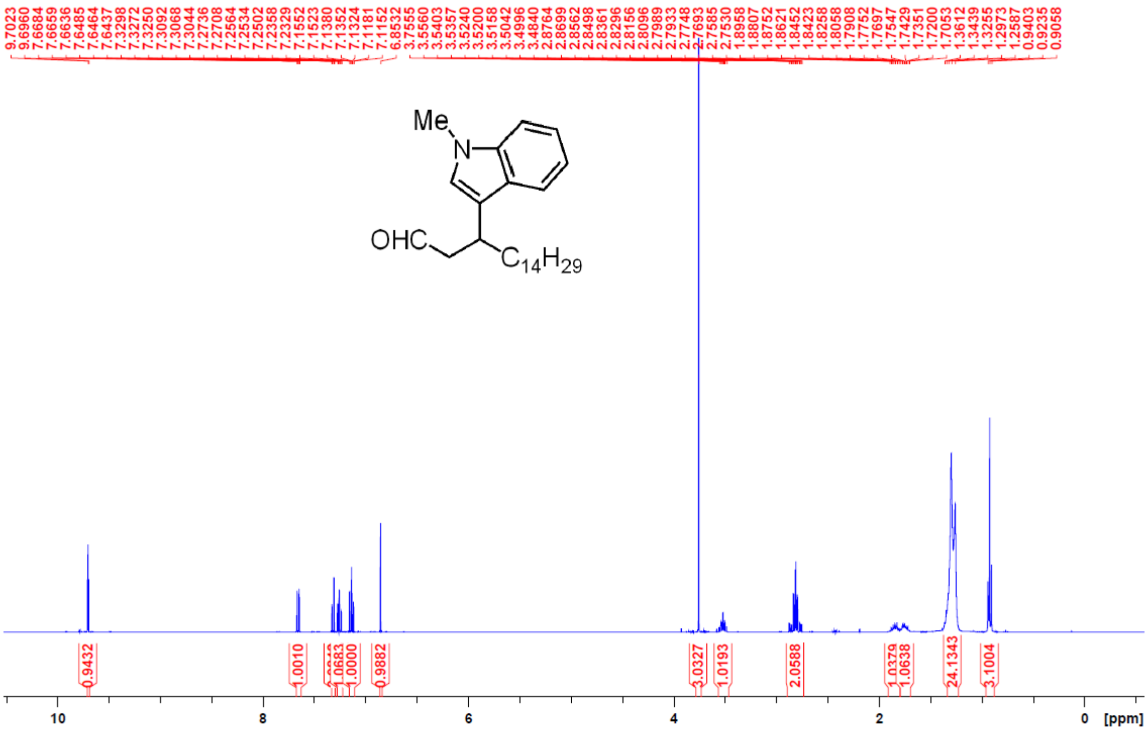

Supplementary Figure 84 ^{1}H NMR ($CDCl_3$, 400 MHz) spectrum of **19**

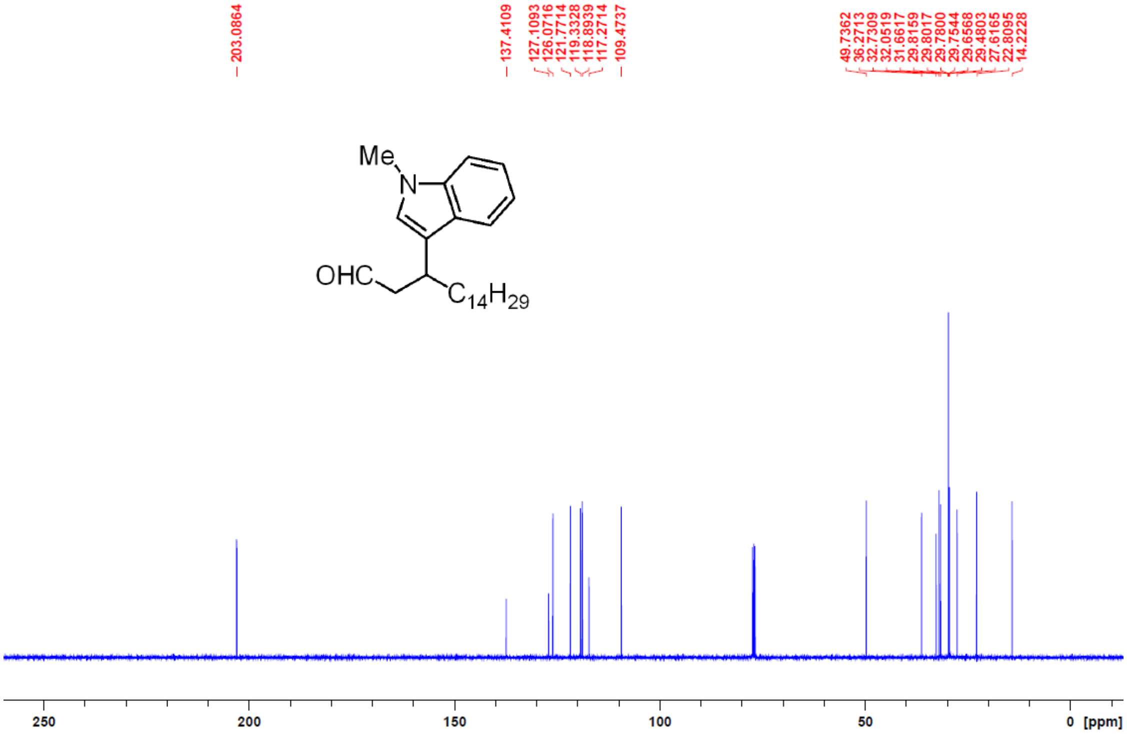

Supplementary Figure 85 ^{13}C NMR ($CDCl_3$, 100 MHz) spectrum of **19**

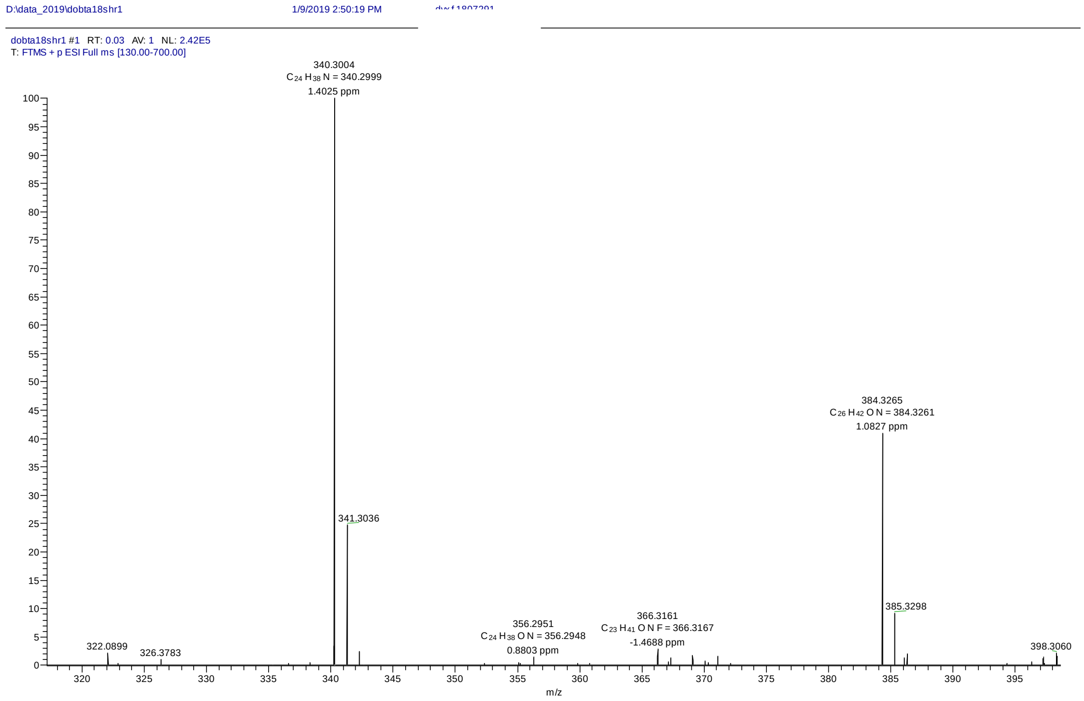

Supplementary Figure 86 ESI-MS spectra of **19**

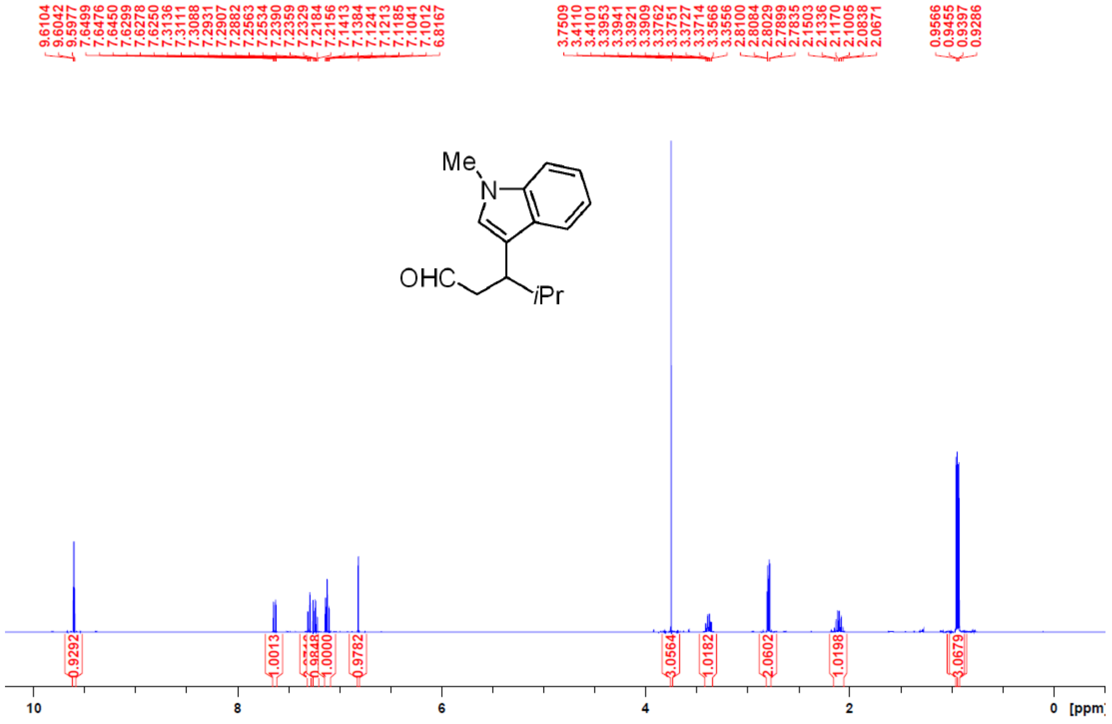

Supplementary Figure 87 ^{1}H NMR ($CDCl_3$, 400 MHz) spectrum of **20**

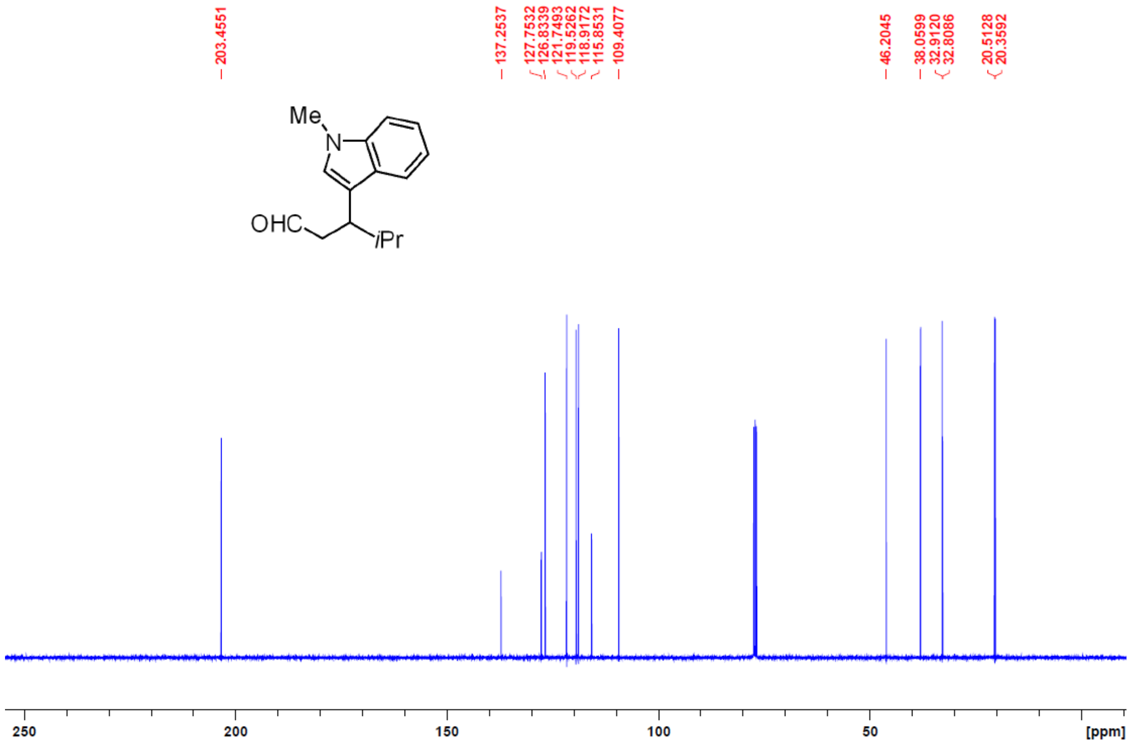

Supplementary Figure 88 ^{13}C NMR ($CDCl_3$, 100 MHz) spectrum of **20**

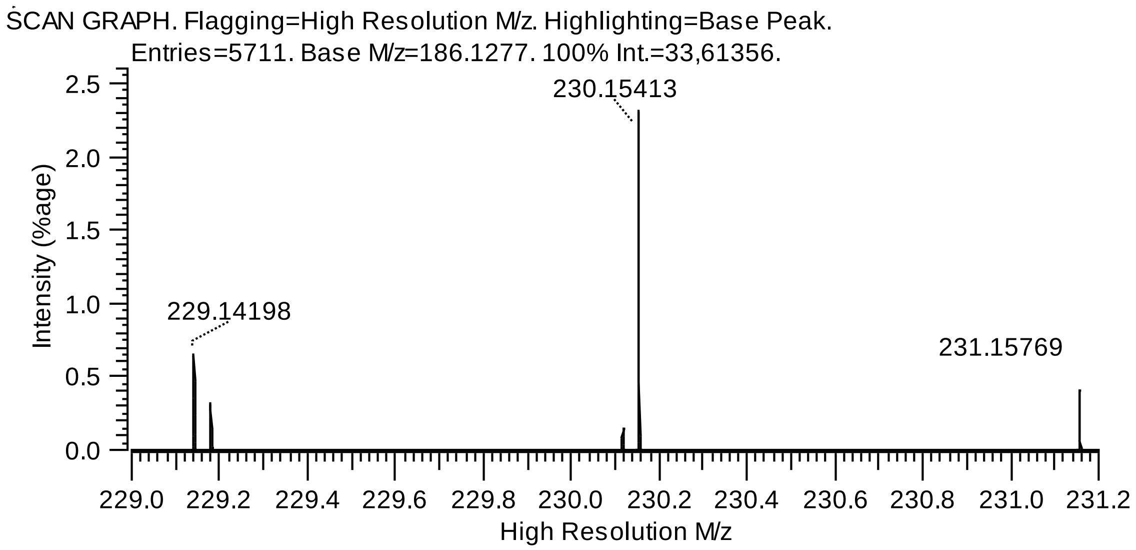

Supplementary Figure 89 ESI-MS spectra of **20**

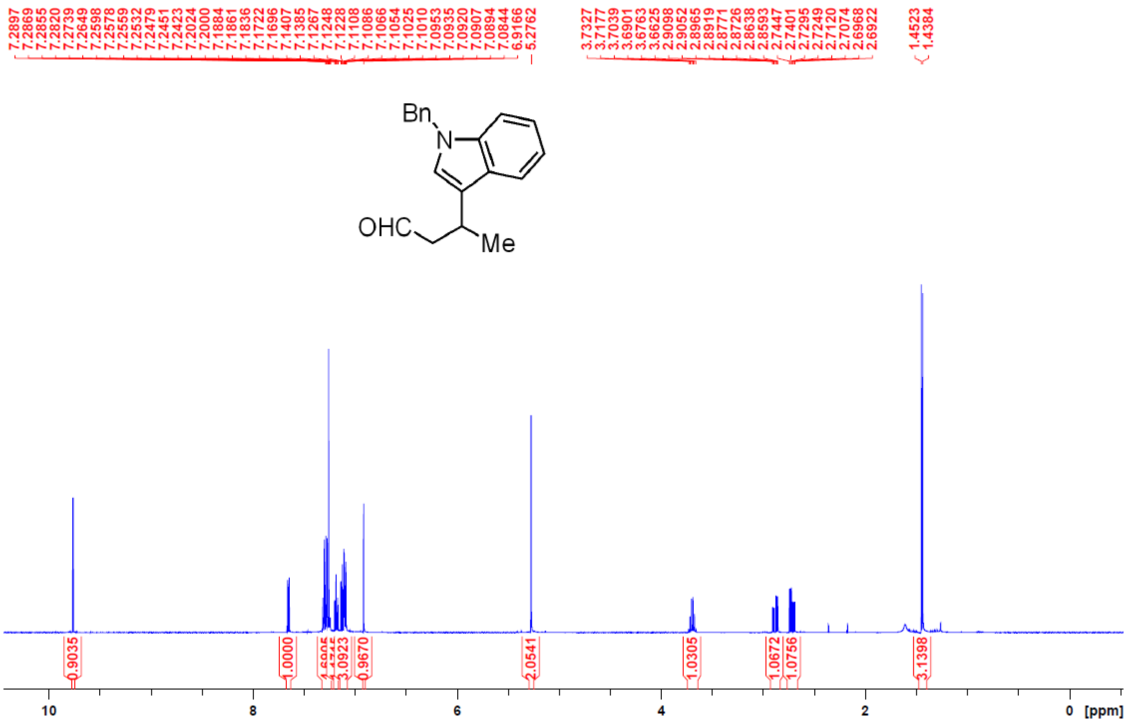

Supplementary Figure 90 ^{1}H NMR ($CDCl_3$, 500 MHz) spectrum of **21**

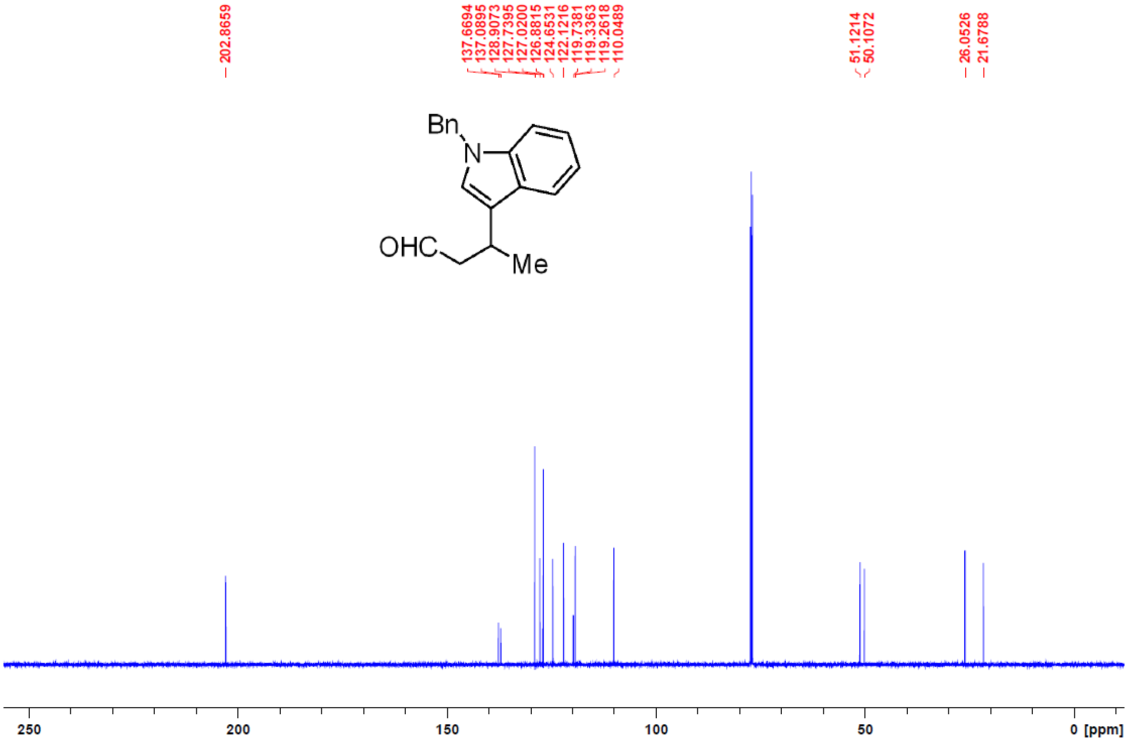

Supplementary Figure 91 ^{13}C NMR ($CDCl_3$, 126 MHz) spectrum of **21**

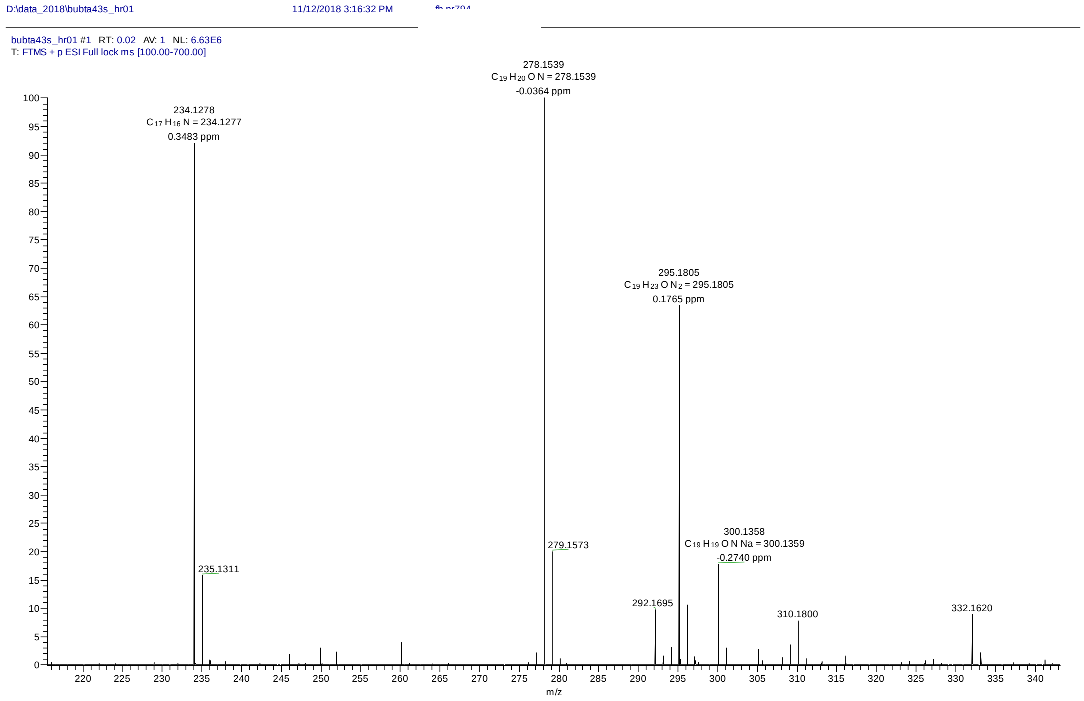

Supplementary Figure 92 ESI-MS spectra of **21**

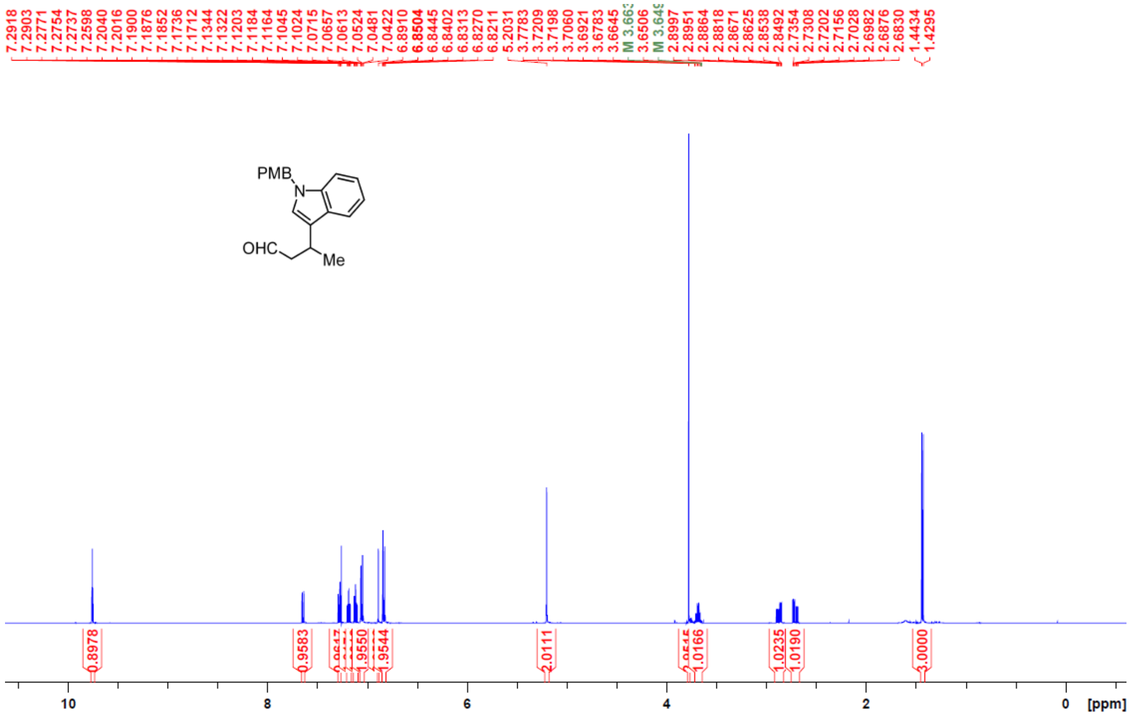

Supplementary Figure 93 ^{1}H NMR ($CDCl_3$, 500 MHz) spectrum of **22**

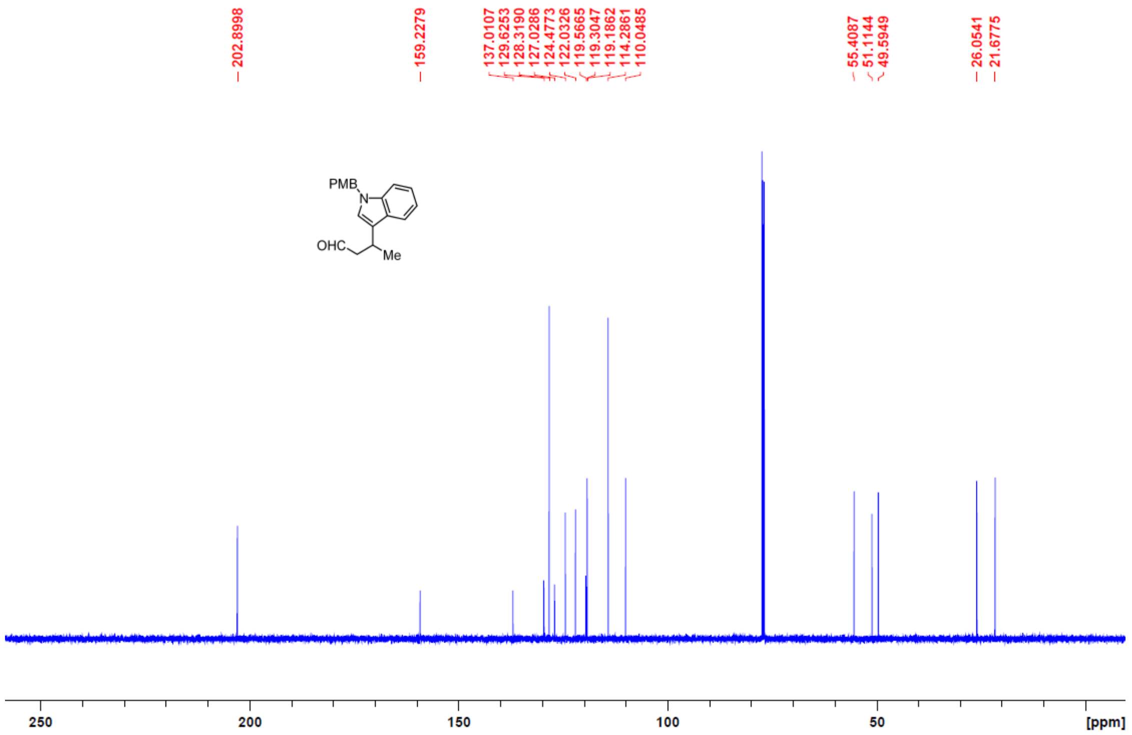

Supplementary Figure 94 ^{13}C NMR ($CDCl_3$, 126 MHz) spectrum of **22**

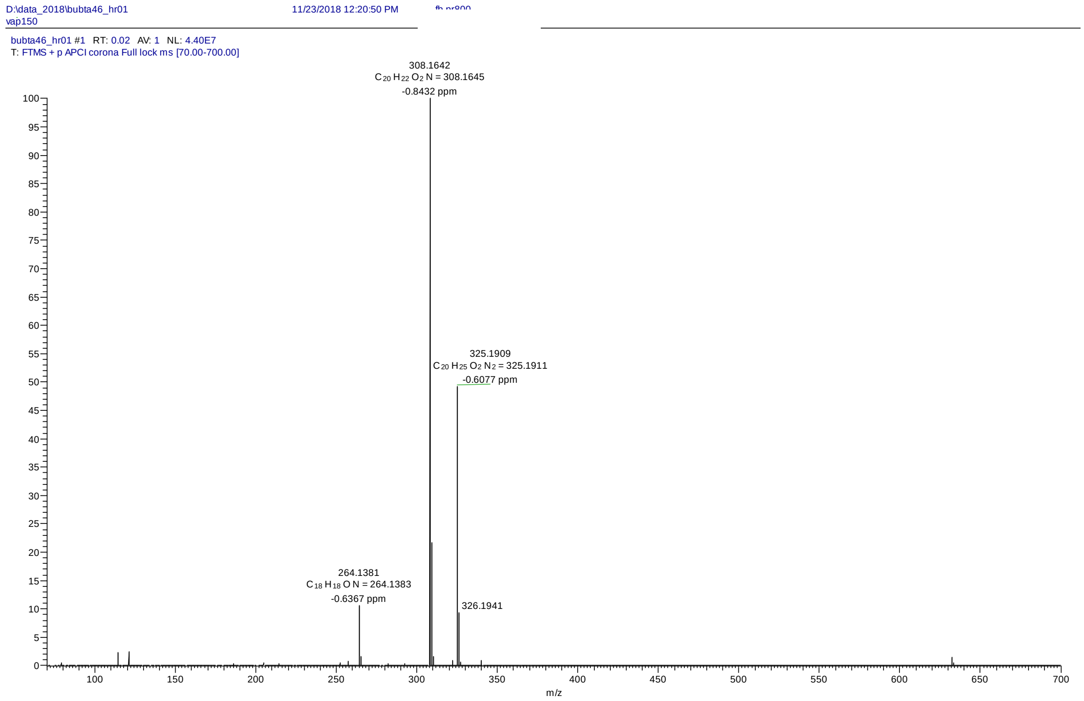

Supplementary Figure 95 ESI-MS spectra of **22**

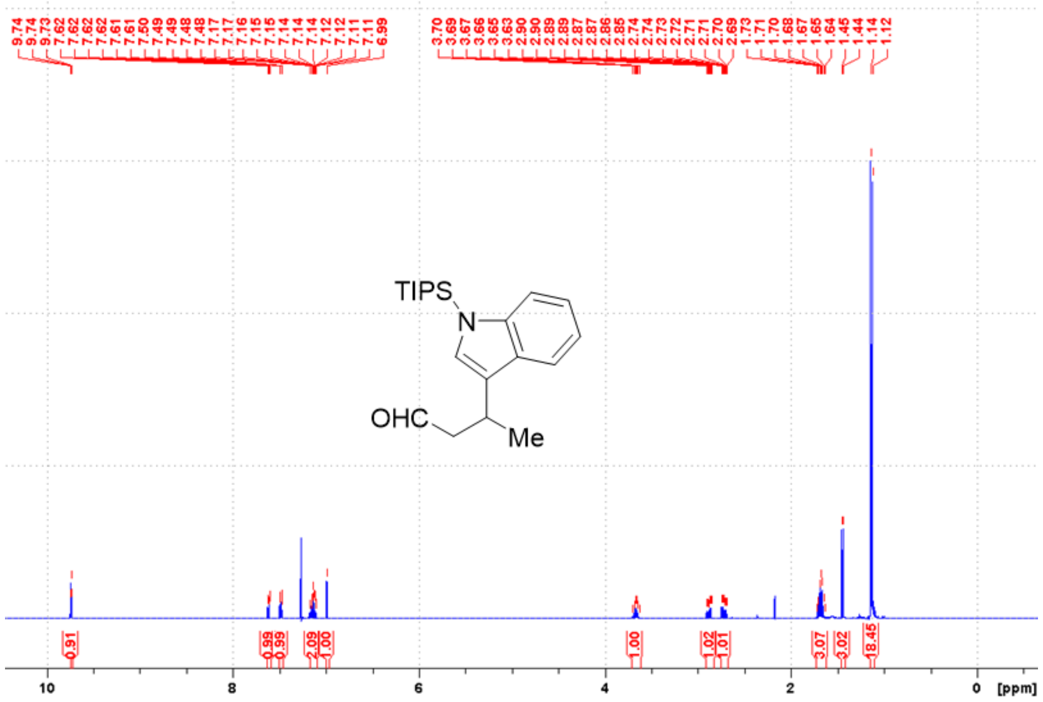

Supplementary Figure 96 ^{1}H NMR ($CDCl_3$, 500 MHz) spectrum of **23**

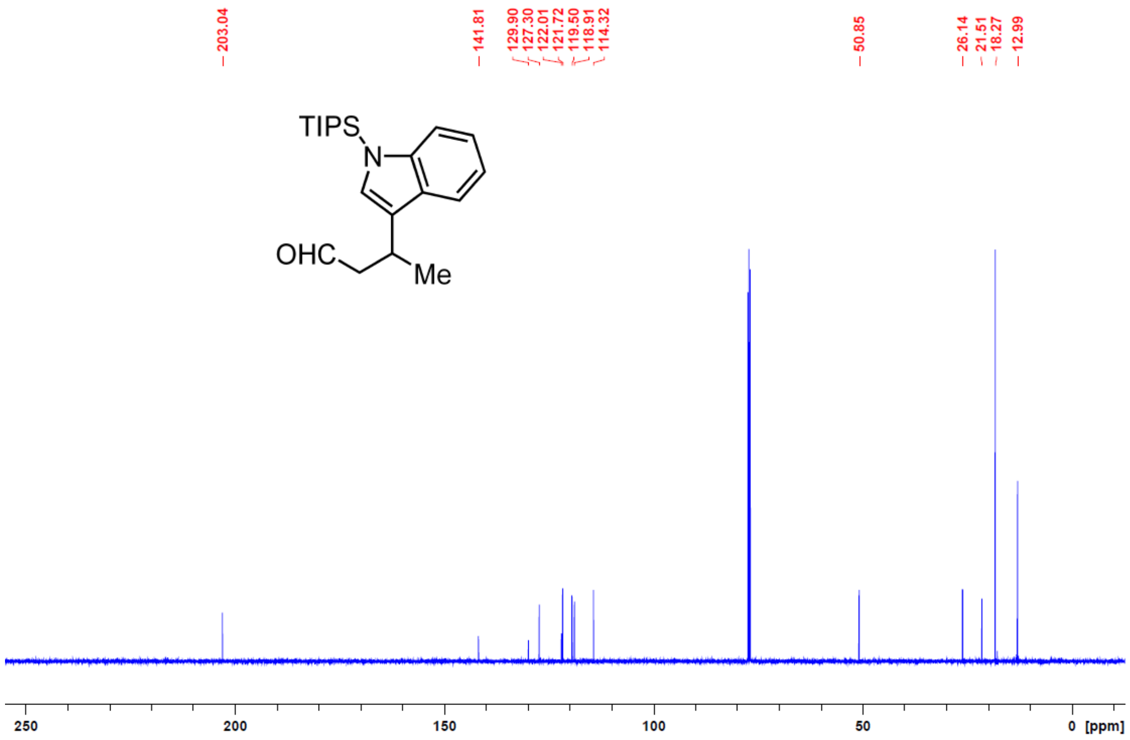

Supplementary Figure 97 ^{13}C NMR ($CDCl_3$, 126 MHz) spectrum of **23**

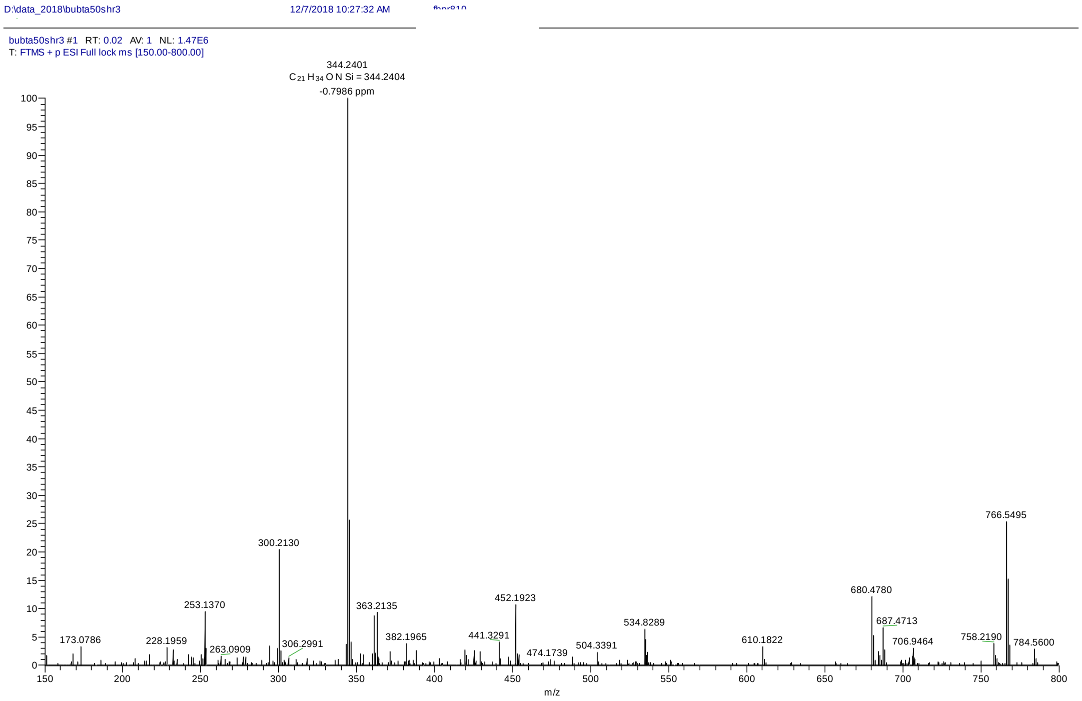

Supplementary Figure 98 ESI-MS spectra of **23**

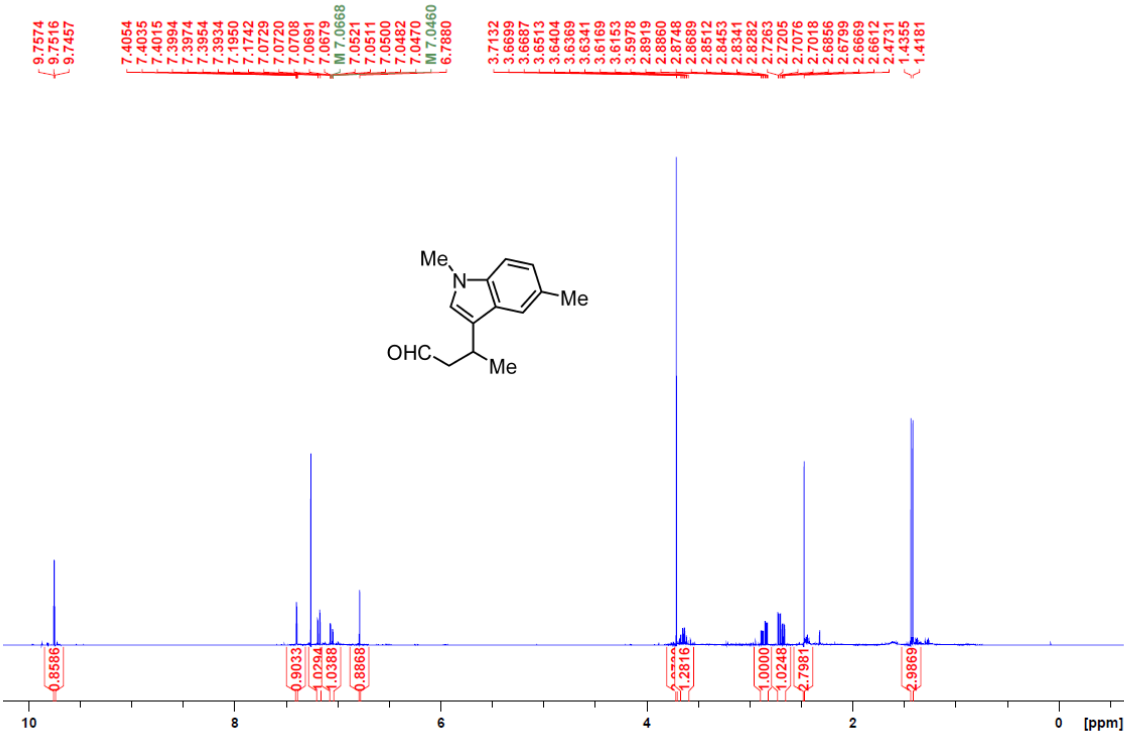

Supplementary Figure 99 ^{1}H NMR ($CDCl_3$, 400 MHz) spectrum of **25**

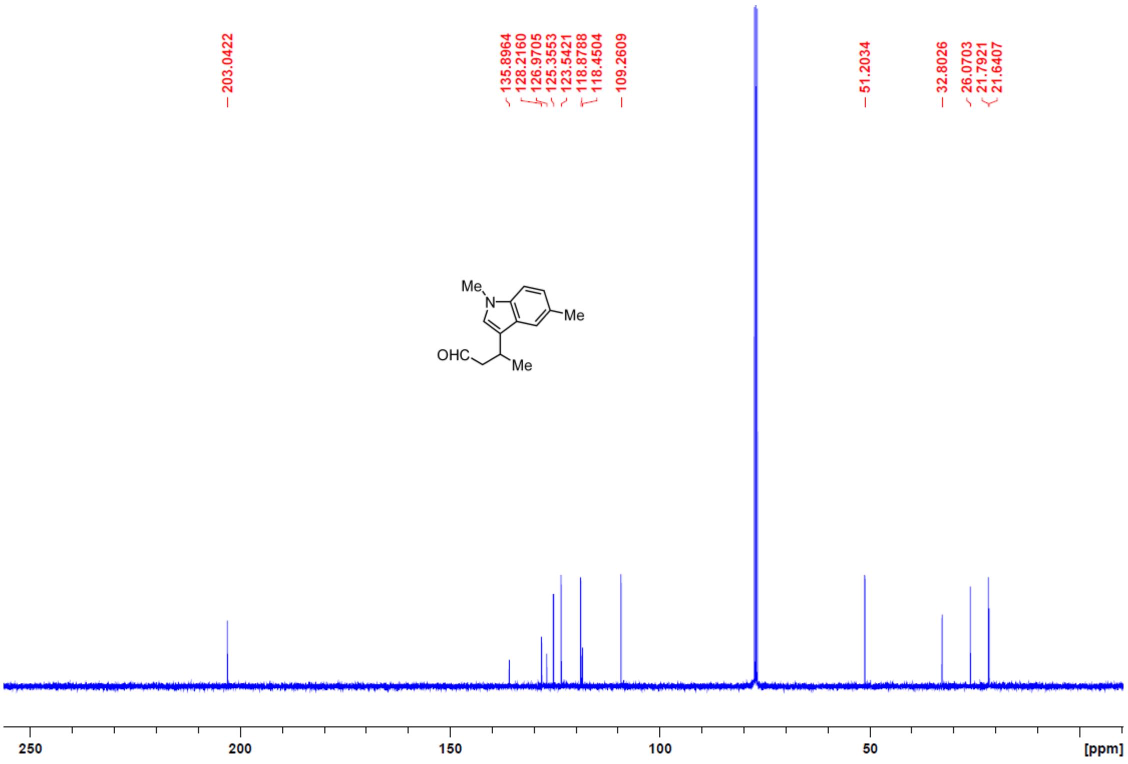

Supplementary Figure 100 ^{13}C NMR ($CDCl_3$, 100 MHz) spectrum of **25**

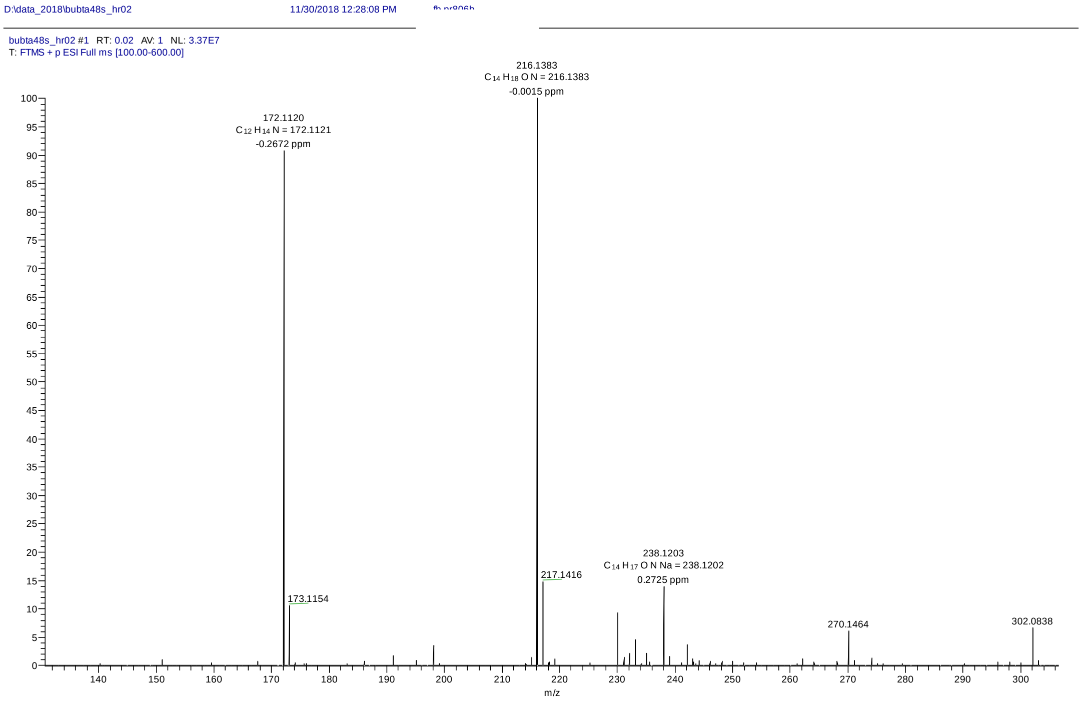

Supplementary Figure 101 ESI-MS spectra of **25**

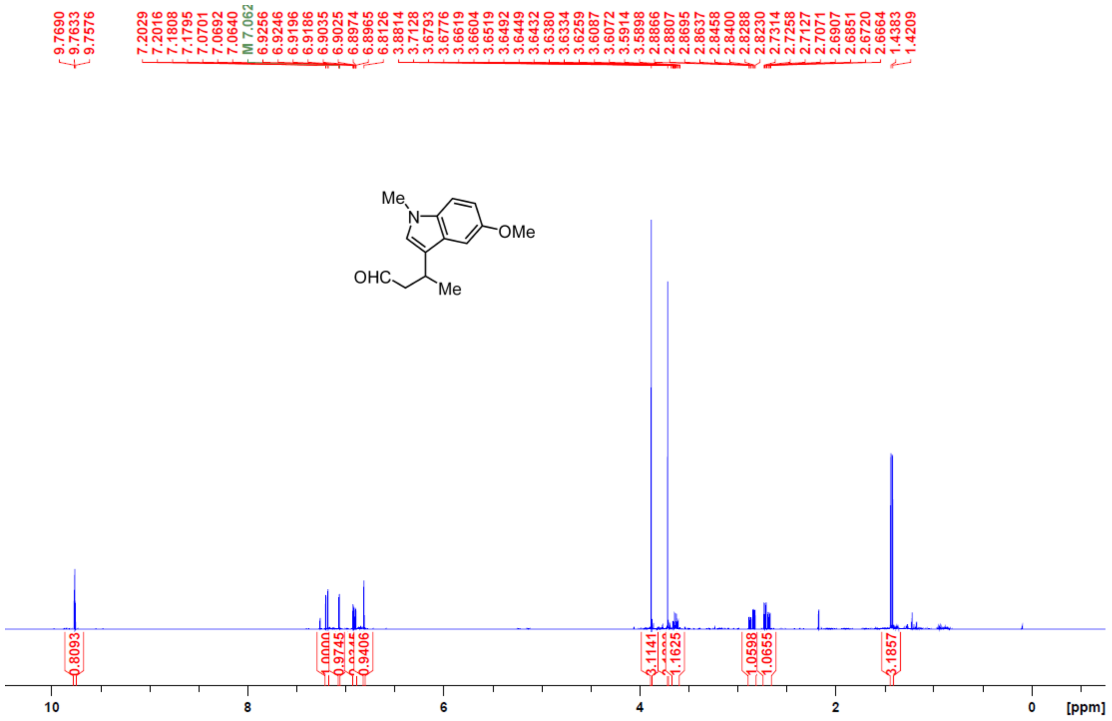

Supplementary Figure 102 ^{1}H NMR ($CDCl_3$, 500 MHz) spectrum of **26**

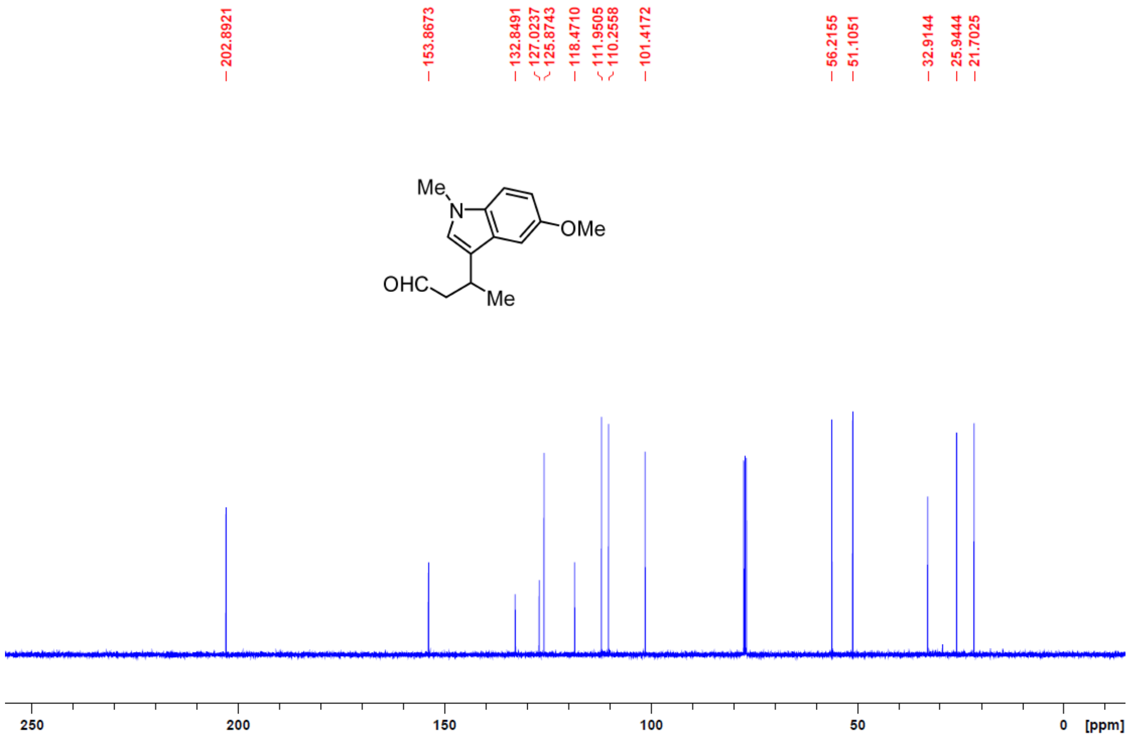

Supplementary Figure 103 ^{13}C NMR ($CDCl_3$, 126 MHz) spectrum of **26**

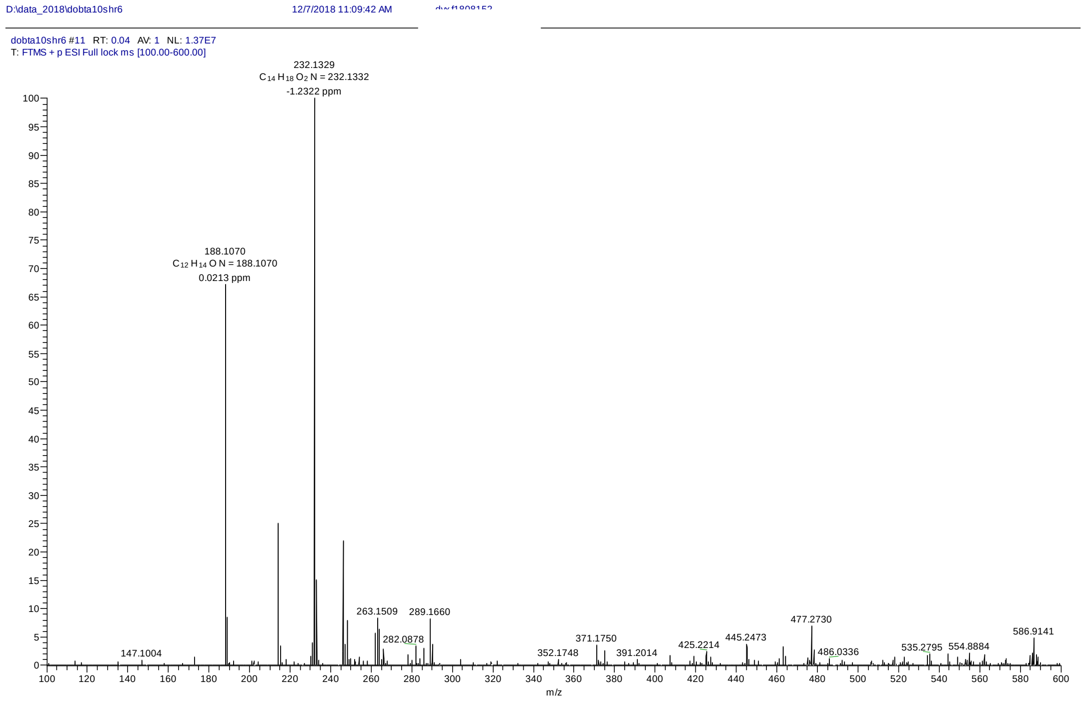

Supplementary Figure 104 ESI-MS spectra of **26**

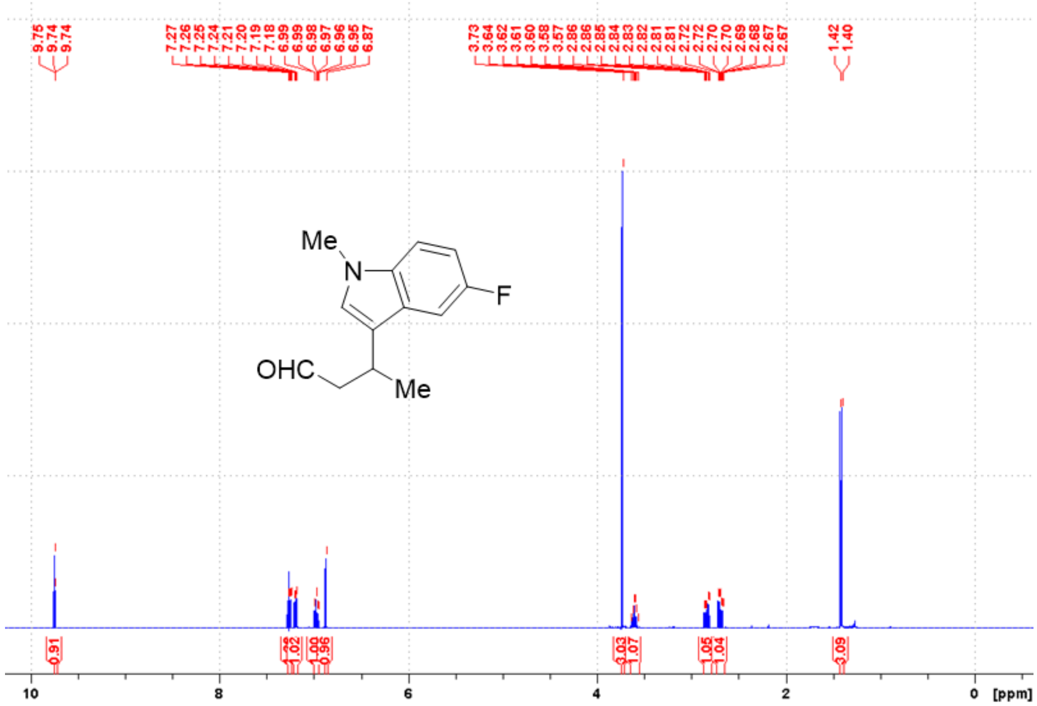

Supplementary Figure 105 ^{1}H NMR ($CDCl_3$, 500 MHz) spectrum of **27**

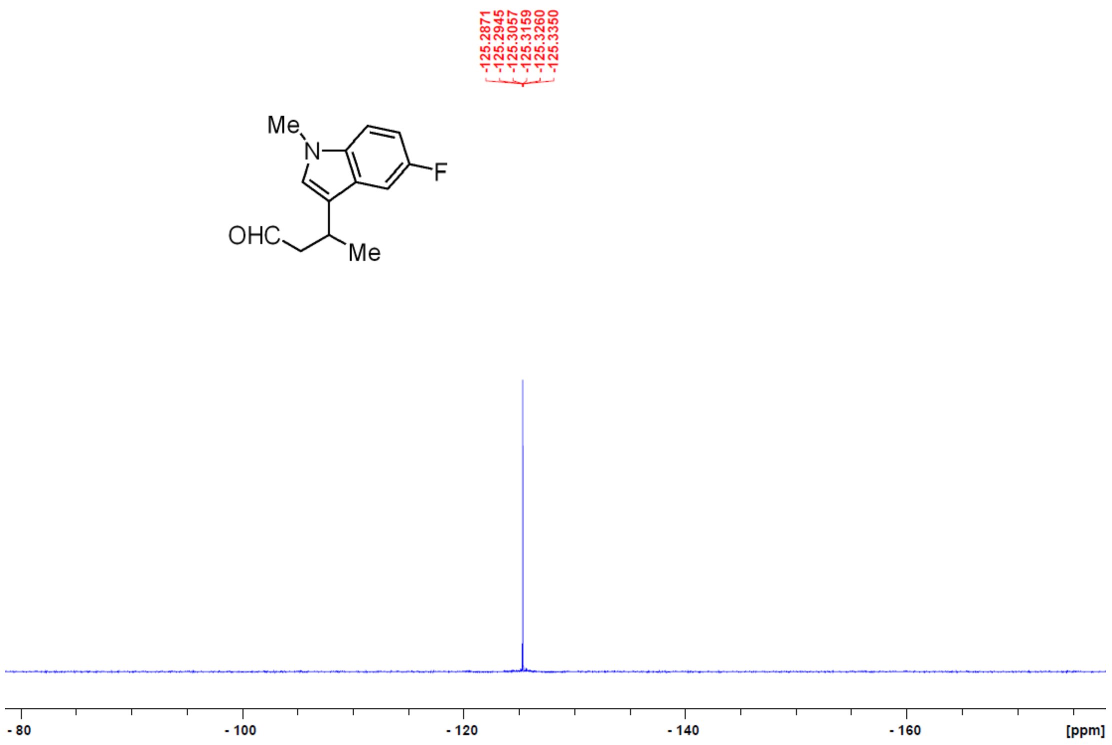

Supplementary Figure 106 ^{19}F NMR ($CDCl_3$, 470 MHz) spectrum of **27**

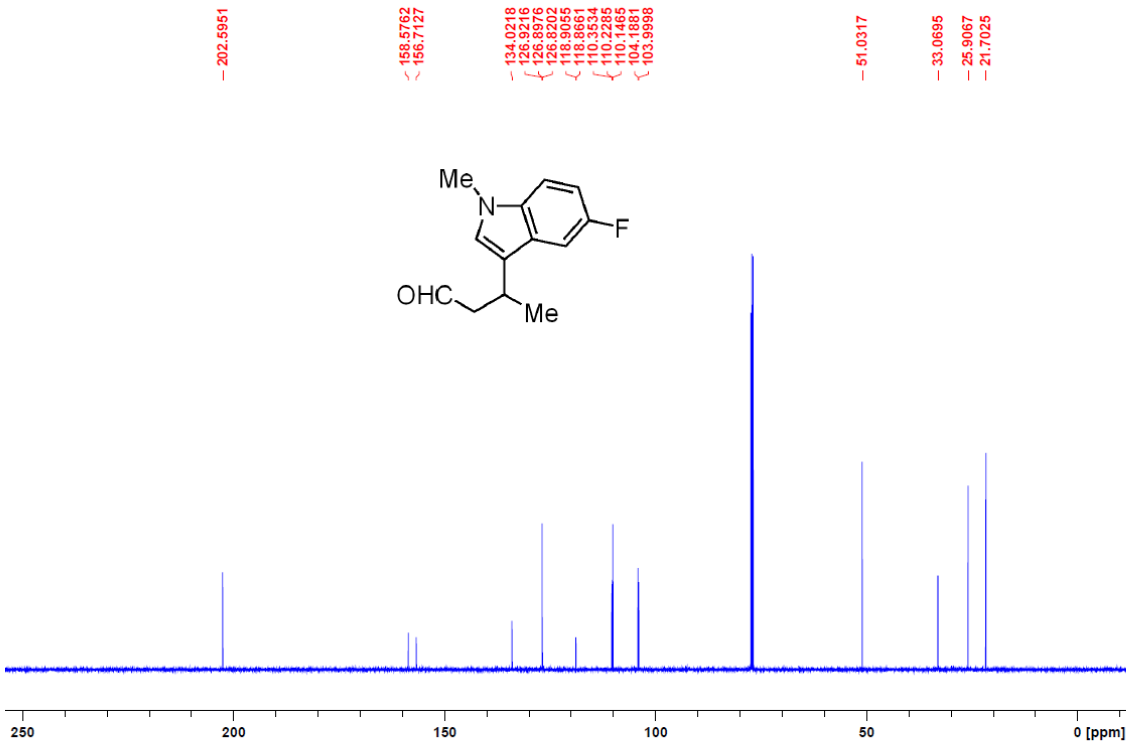

Supplementary Figure 107 ^{13}C NMR ($CDCl_3$, 126 MHz) spectrum of **27**

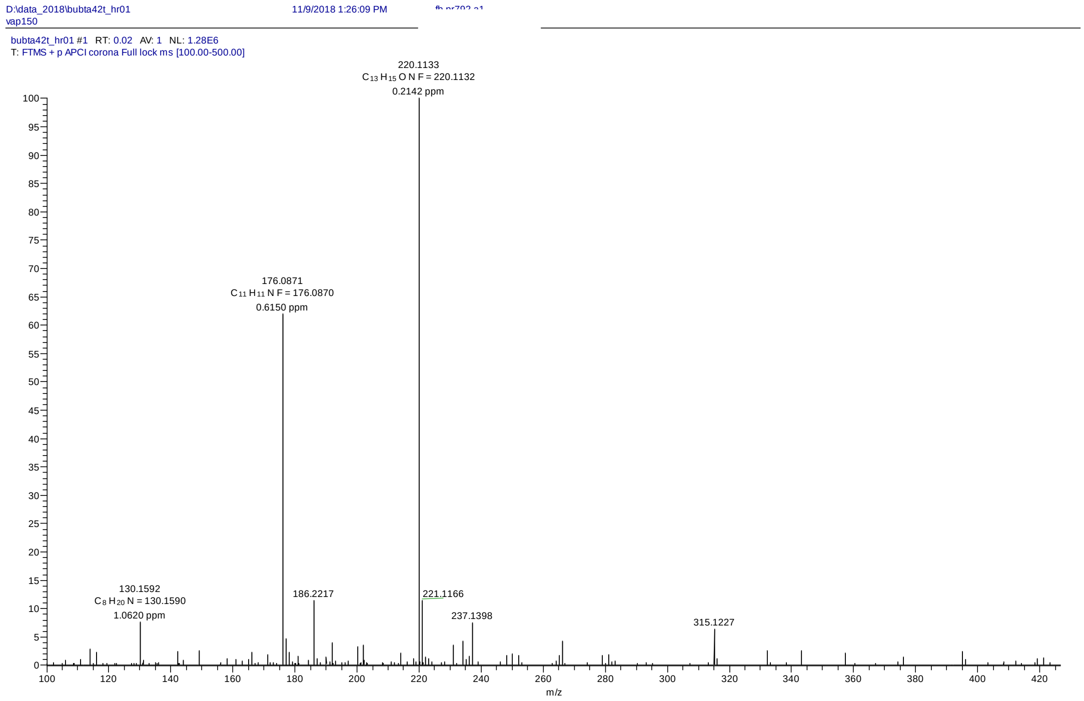

Supplementary Figure 108 ESI-MS spectra of **27**

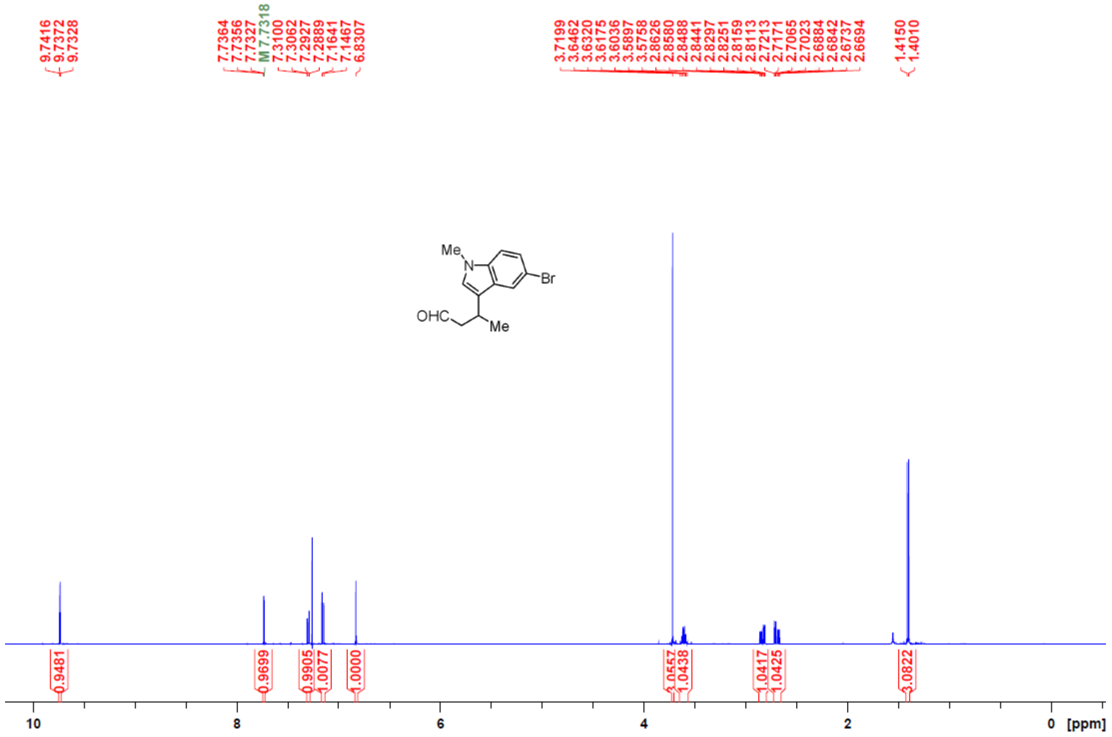

Supplementary Figure 109 ^{1}H NMR ($CDCl_3$, 500 MHz) spectrum of **28**

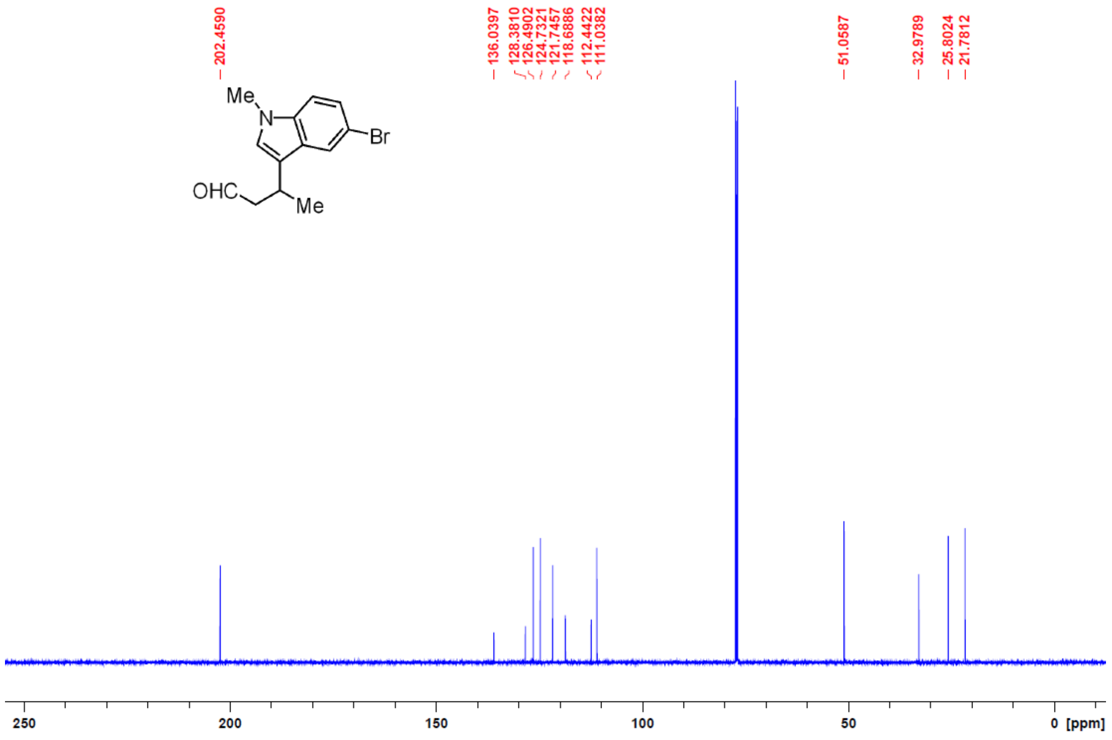

Supplementary Figure 110 ^{13}C NMR ($CDCl_3$, 126 MHz) spectrum of **28**

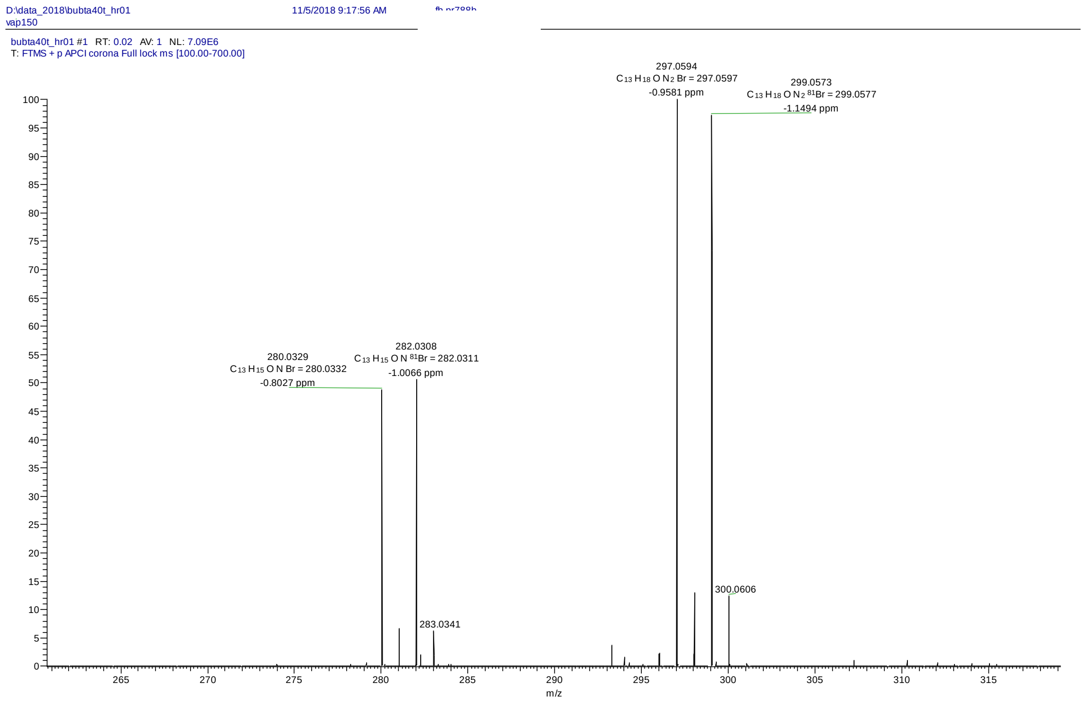

Supplementary Figure 111 ESI-MS spectra of **28**

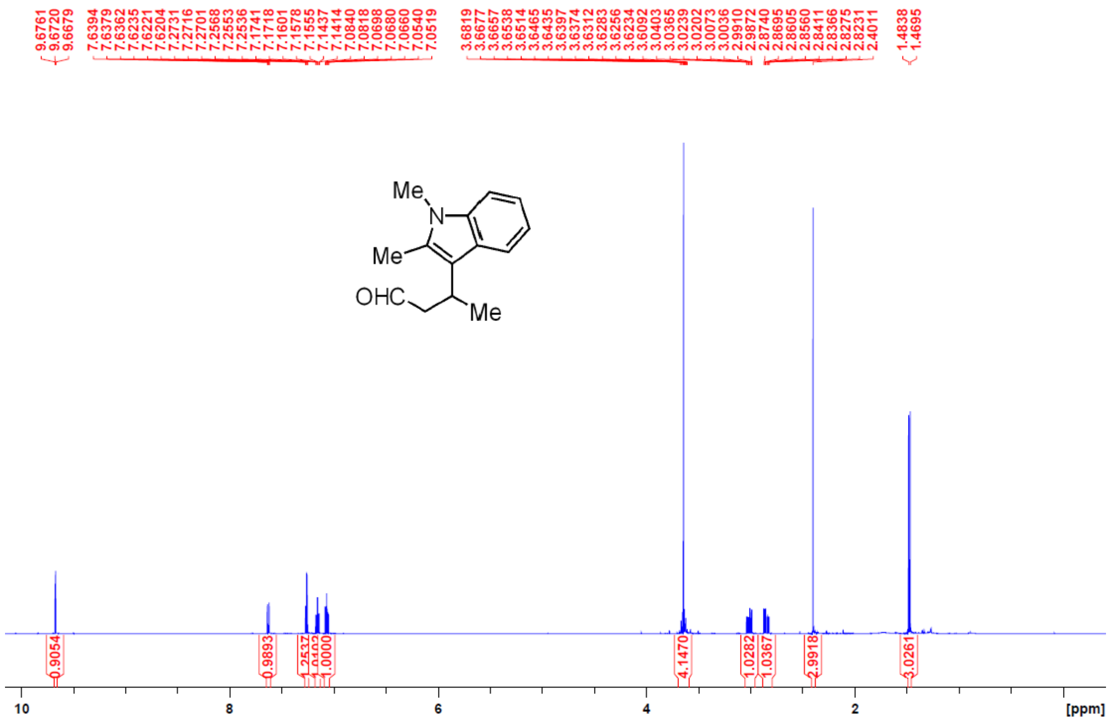

Supplementary Figure 112 ^{1}H NMR ($CDCl_3$, 500 MHz) spectrum of **29**

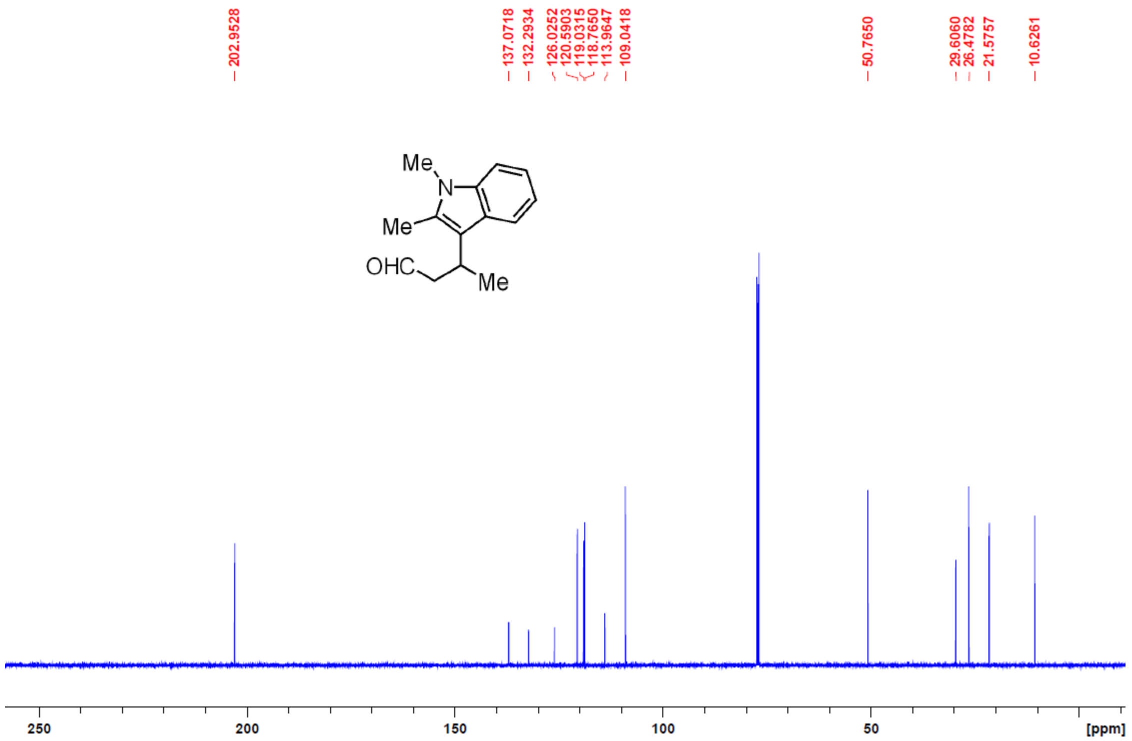

Supplementary Figure 113 ^{13}C NMR ($CDCl_3$, 126 MHz) spectrum of **29**

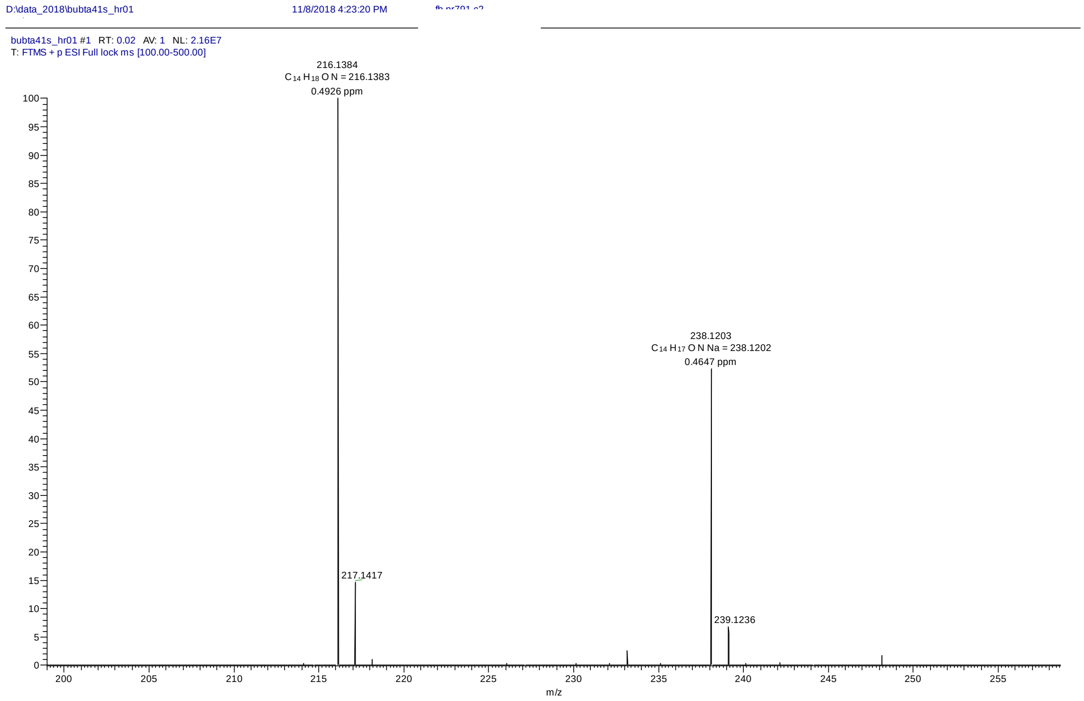

Supplementary Figure 114 ESI-MS spectra of **29**

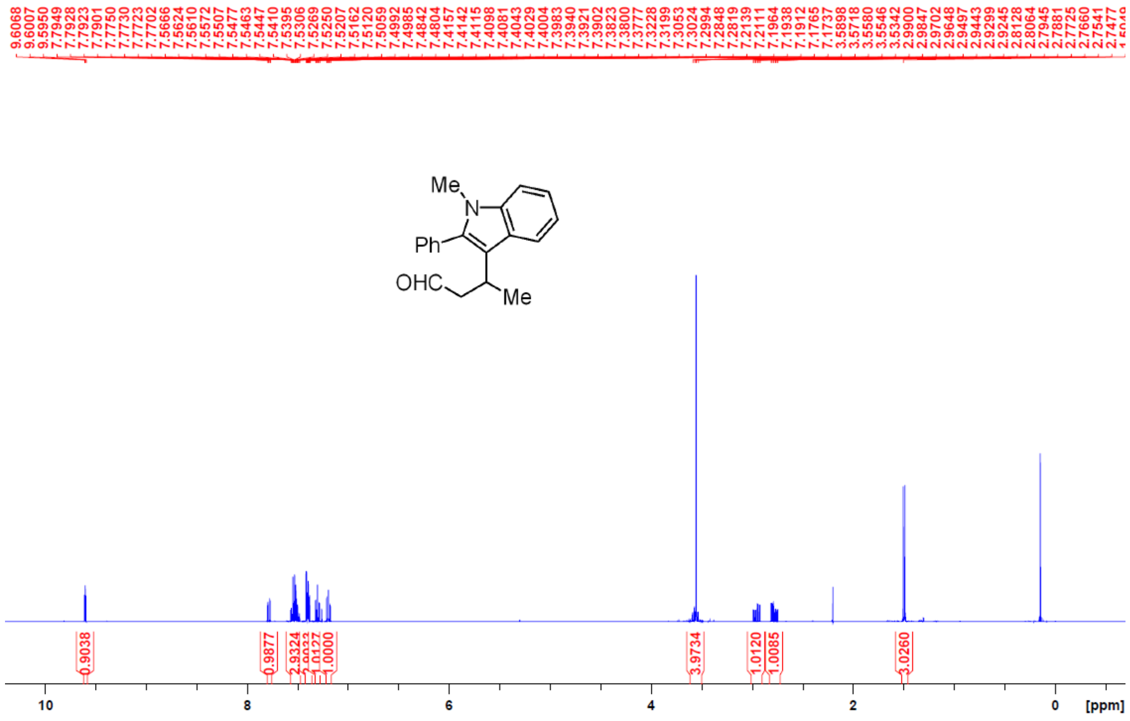

Supplementary Figure 115 ^{1}H NMR ($CDCl_3$, 400 MHz) spectrum of **30**

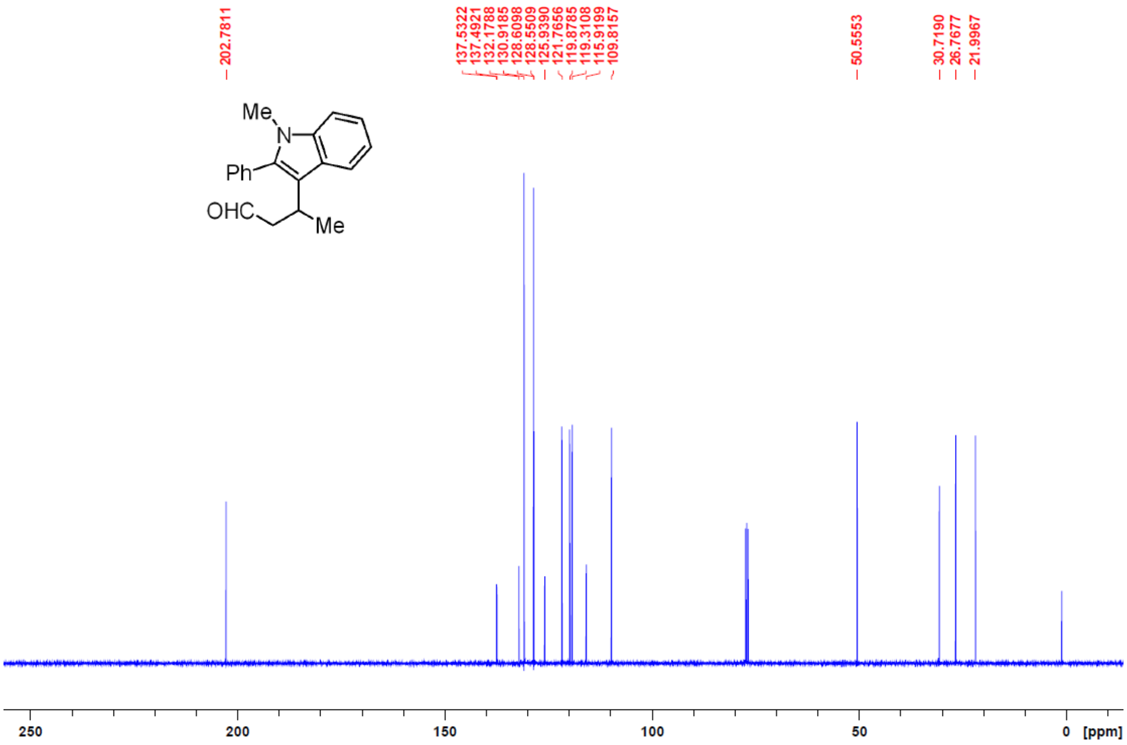

Supplementary Figure 116 ^{13}C NMR ($CDCl_3$, 100 MHz) spectrum of **30**

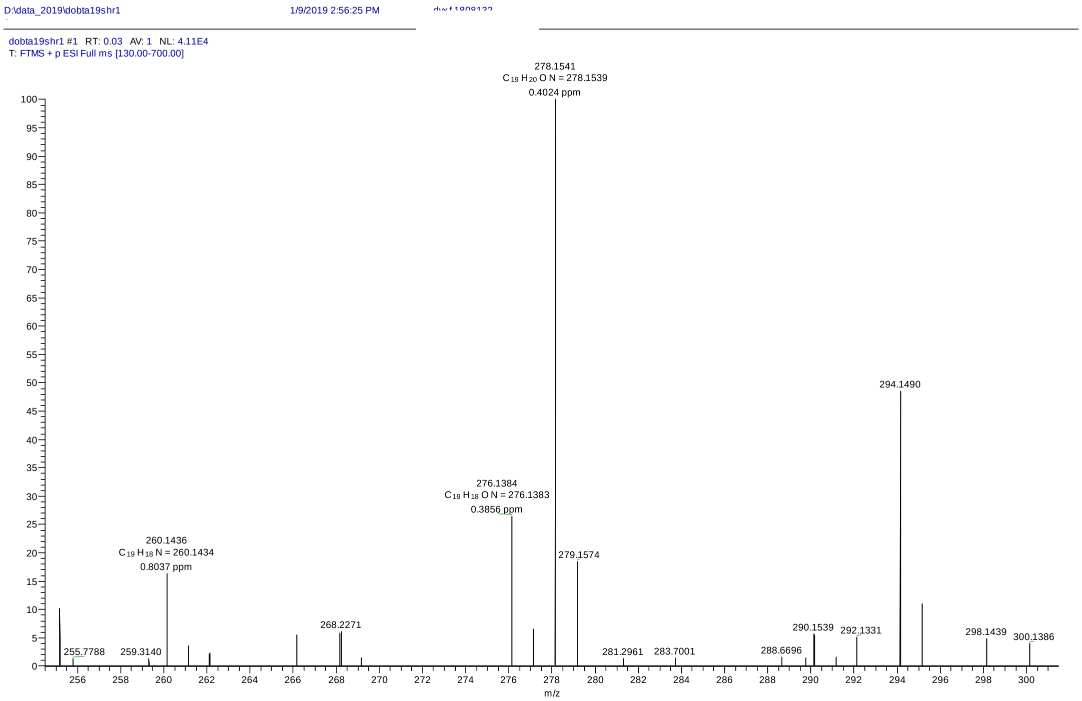

Supplementary Figure 117 ESI-MS spectra of **30**

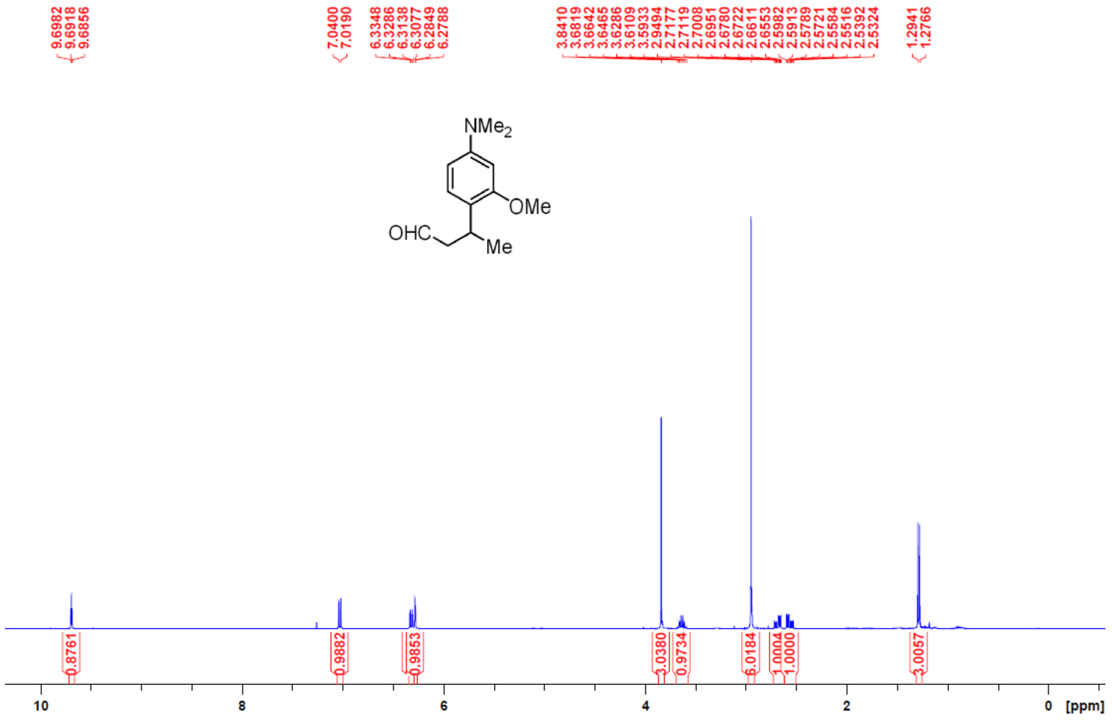

Supplementary Figure 118 ^{1}H NMR ($CDCl_3$, 400 MHz) spectrum of **31**

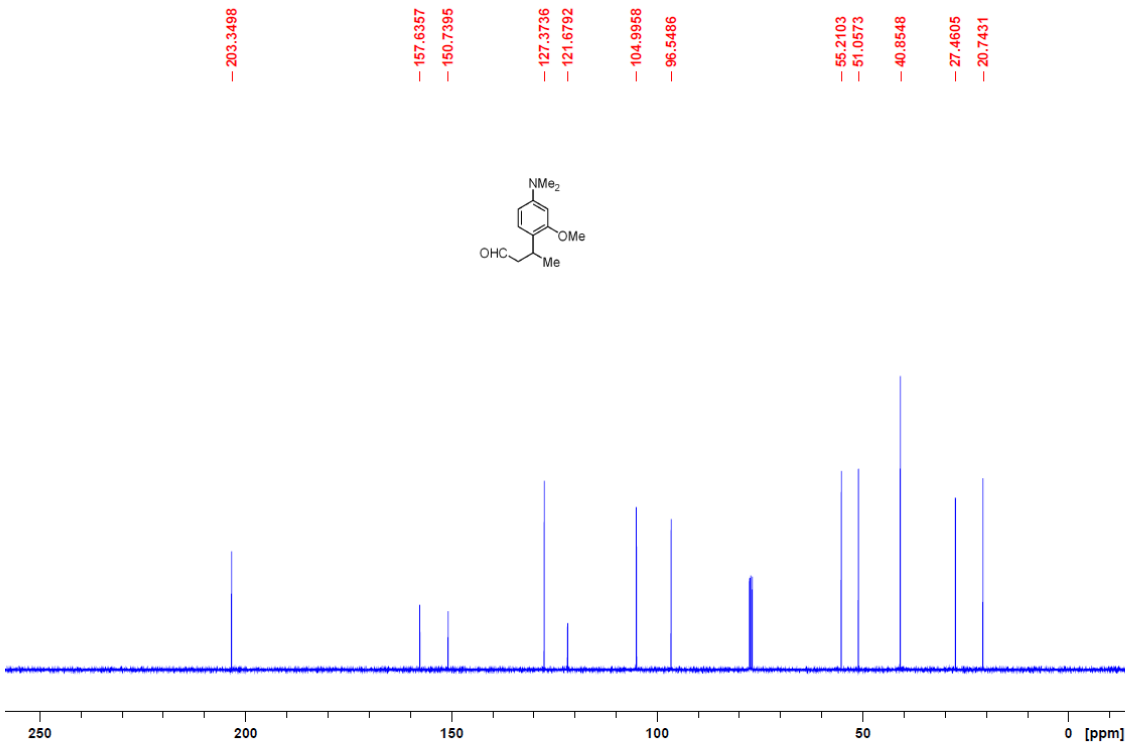

Supplementary Figure 119 ^{13}C NMR ($CDCl_3$, 100 MHz) spectrum of **31**

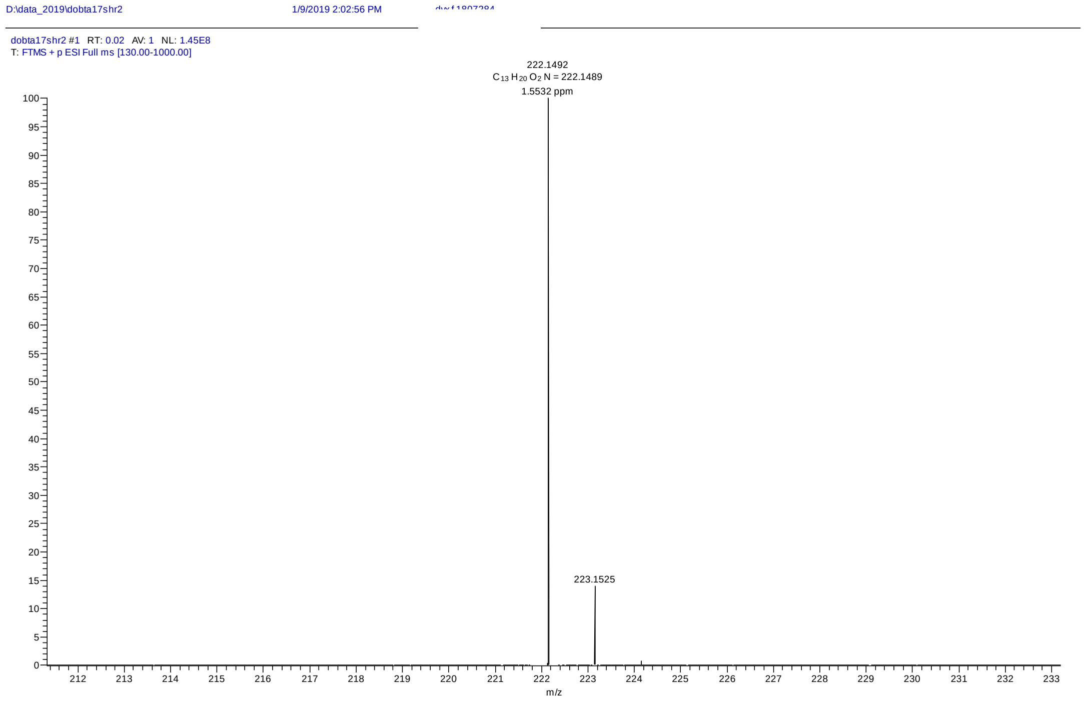

Supplementary Figure 120 ESI-MS spectra of **31**

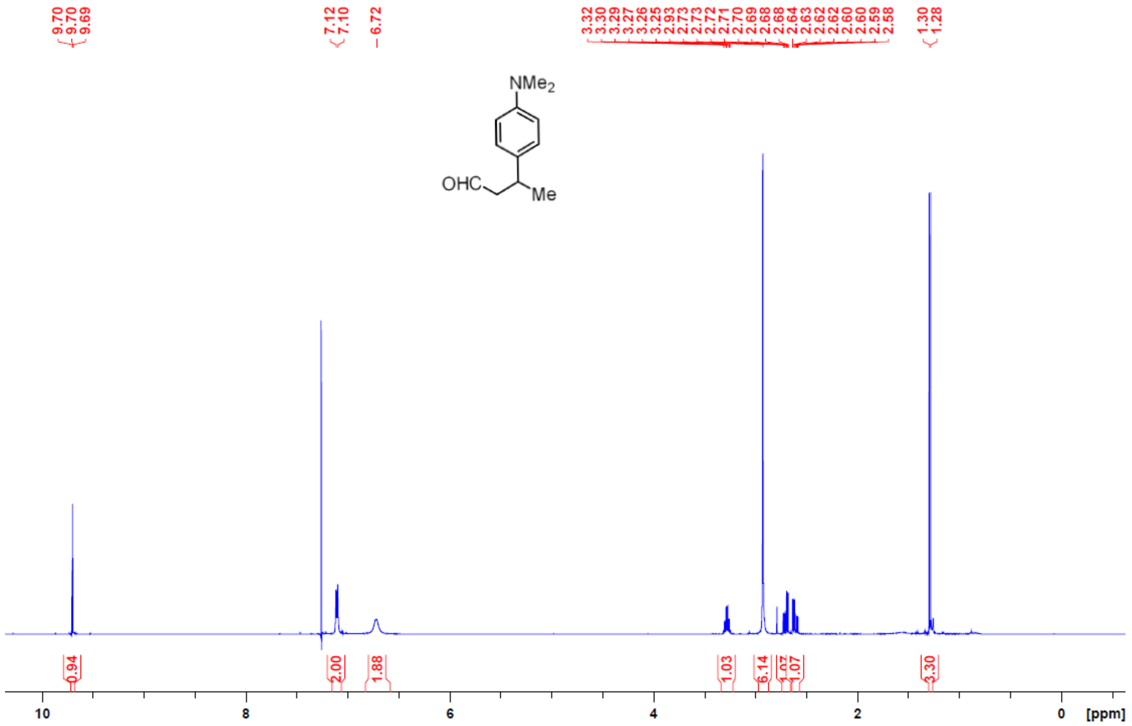

Supplementary Figure 121 ^{1}H NMR ($CDCl_3$, 500 MHz) spectrum of **32**

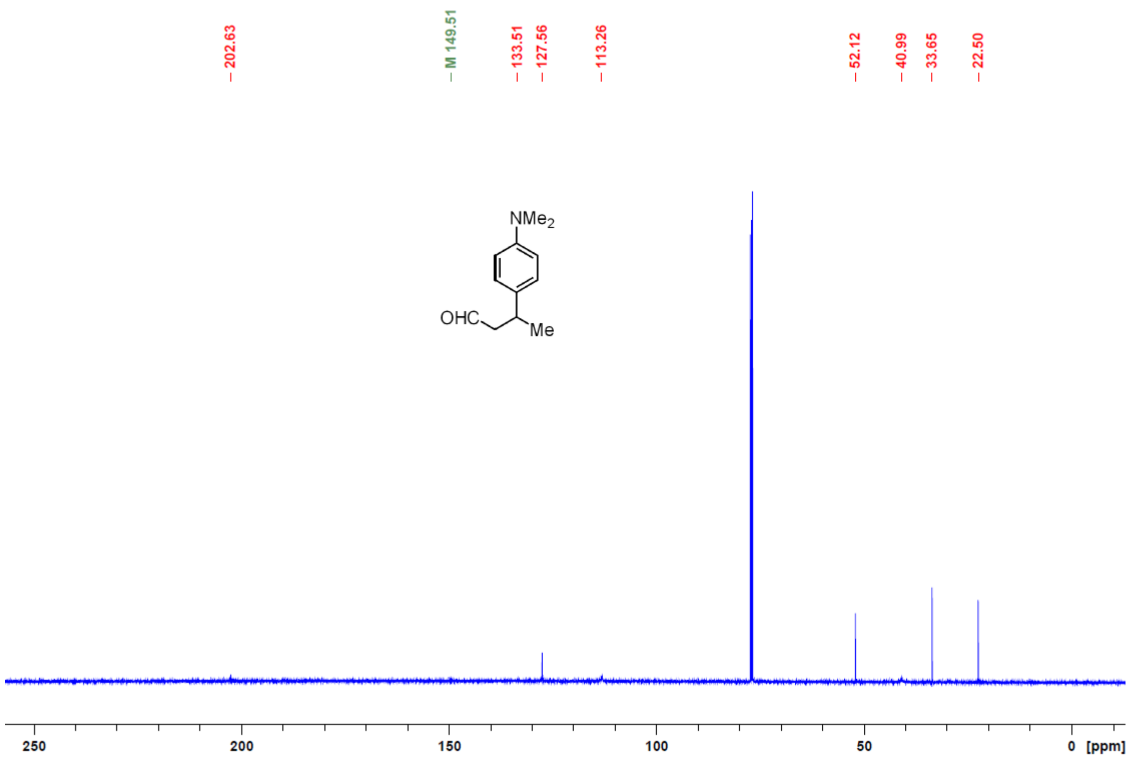

Supplementary Figure 122 ^{13}C NMR ($CDCl_3$, 126 MHz) spectrum of **32**

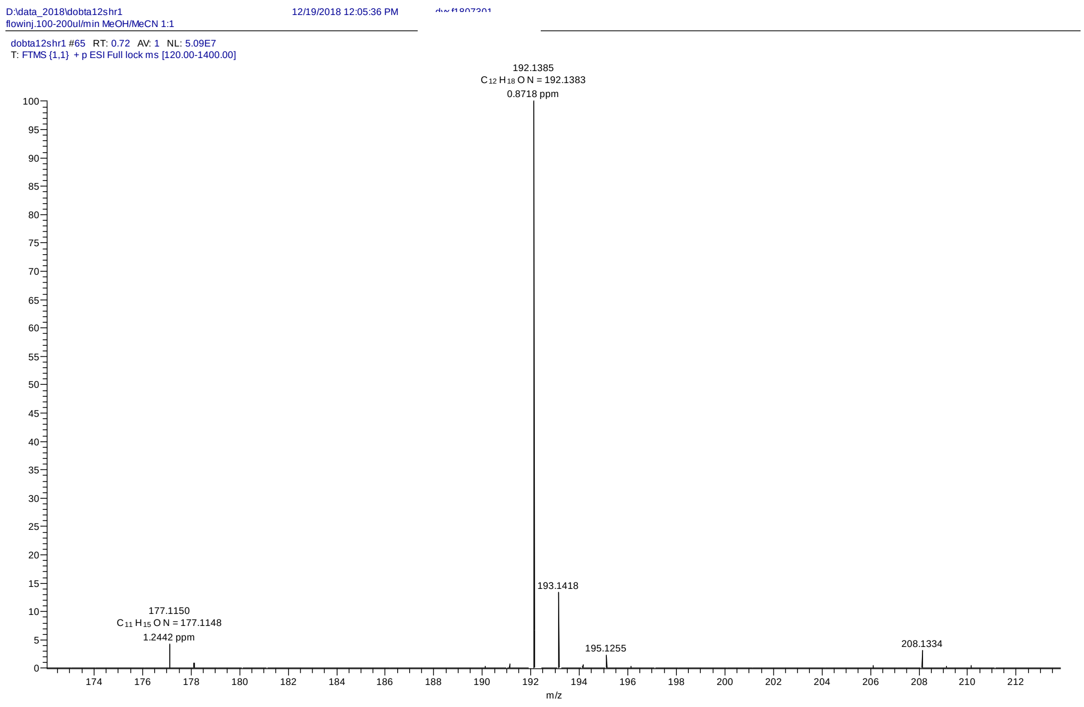

Supplementary Figure 123 ESI-MS spectra of **32**

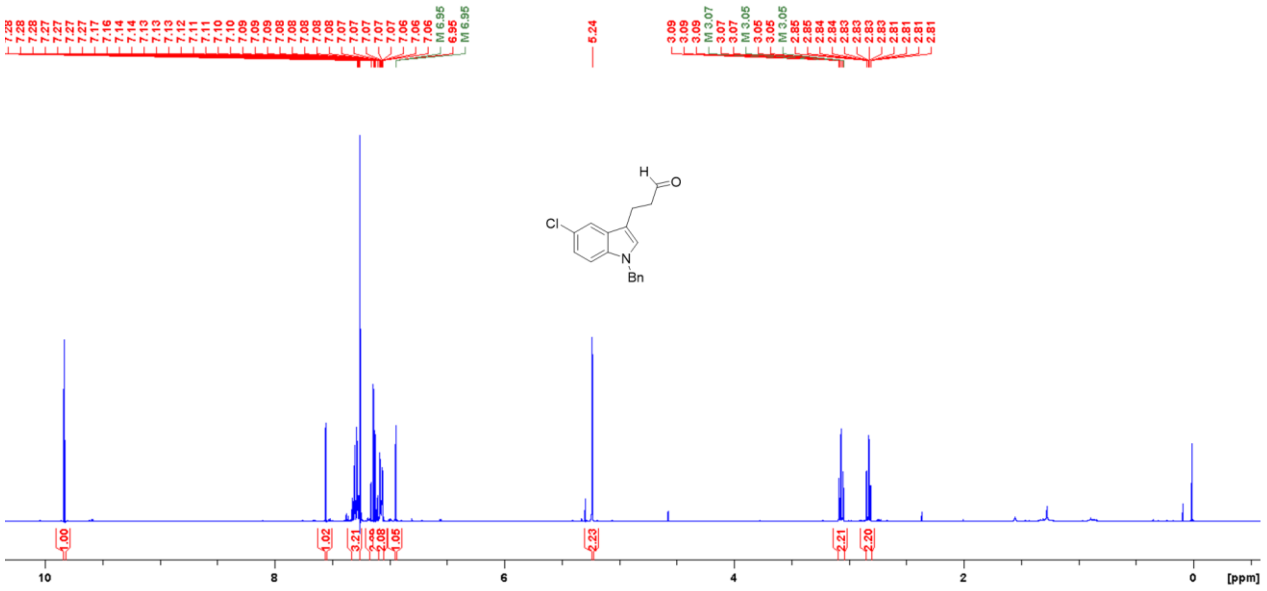

Supplementary Figure 124 ^{1}H NMR ($CDCl_3$, 400 MHz) spectrum of **34a**

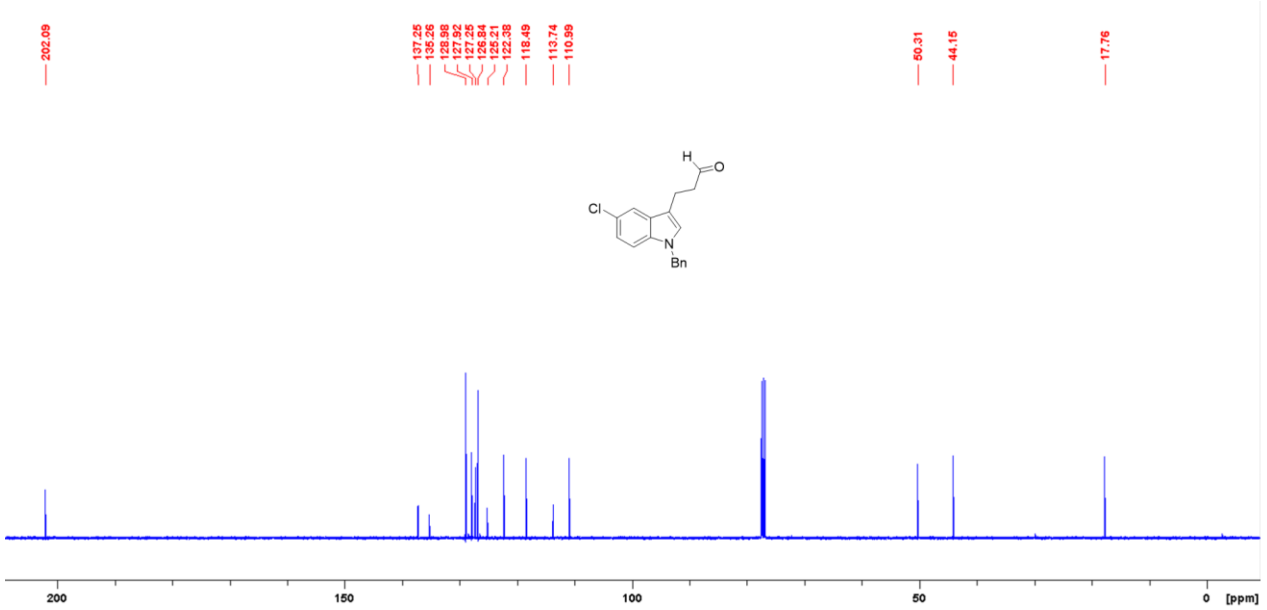

Supplementary Figure 125 ^{13}C NMR ($CDCl_3$, 100 MHz) spectrum of **34a**

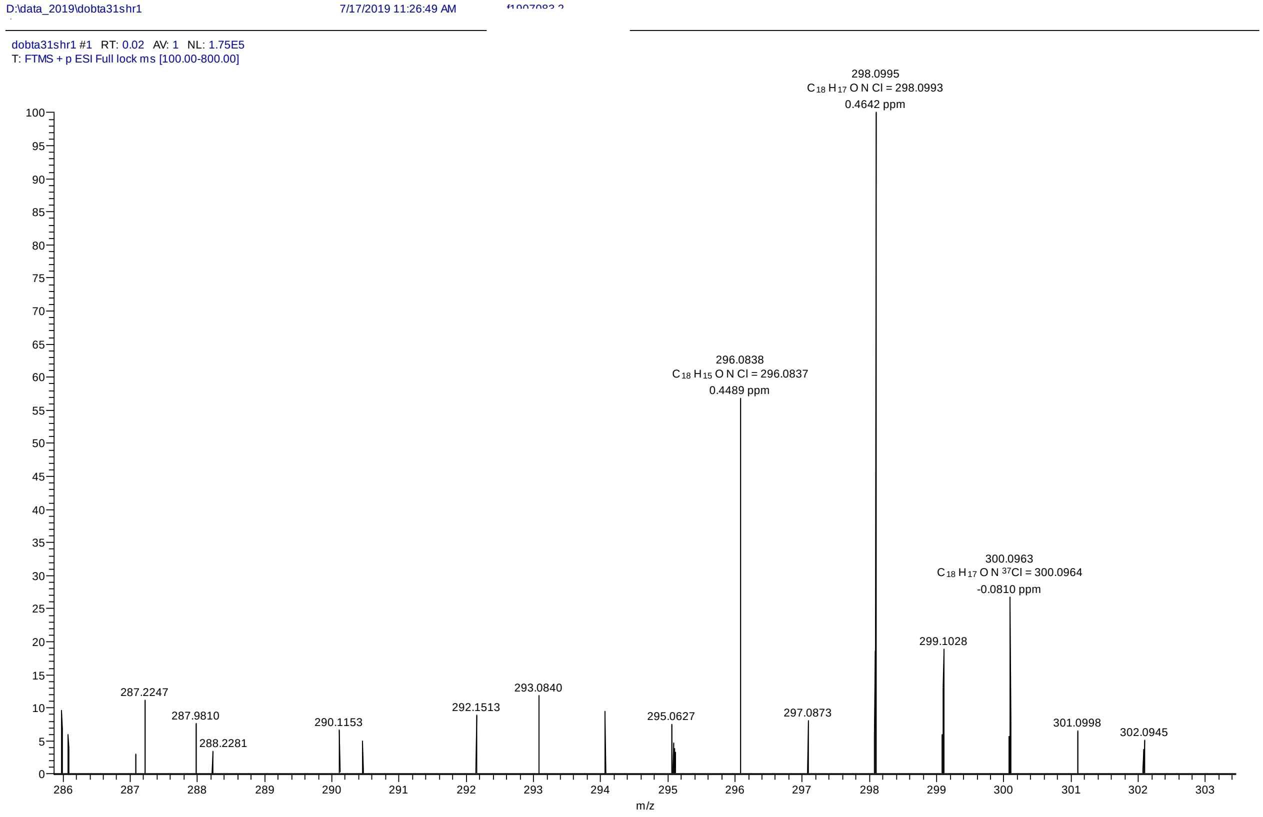

Supplementary Figure 126 ESI-MS spectra of **34a**

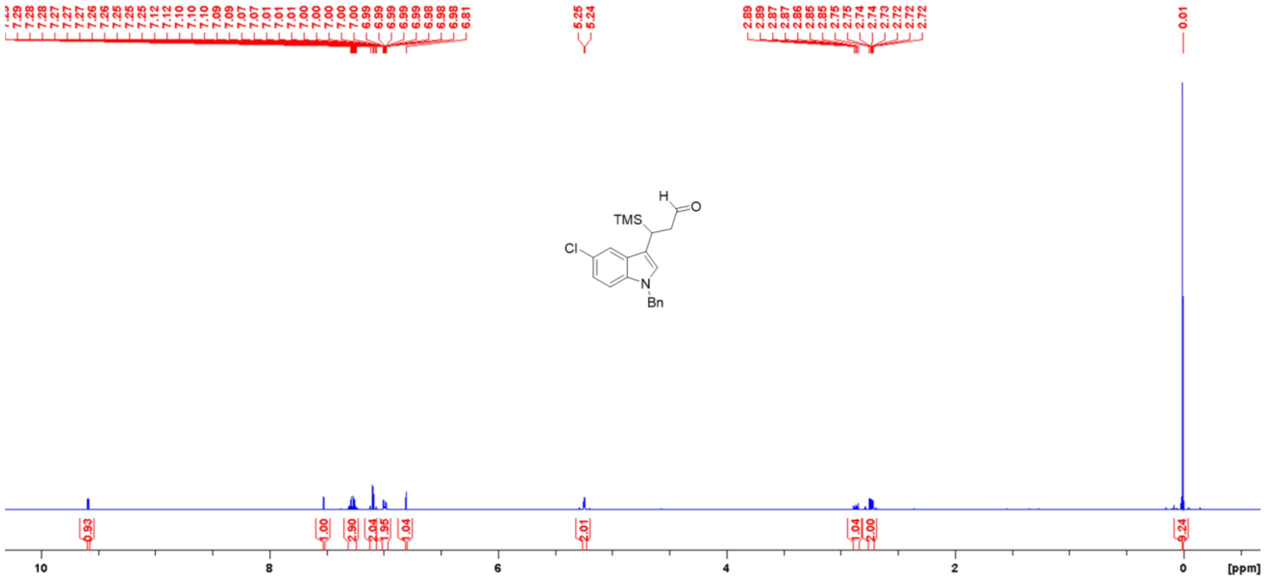

Supplementary Figure 127 ^{1}H NMR ($CDCl_3$, 400 MHz) spectrum of **34b**

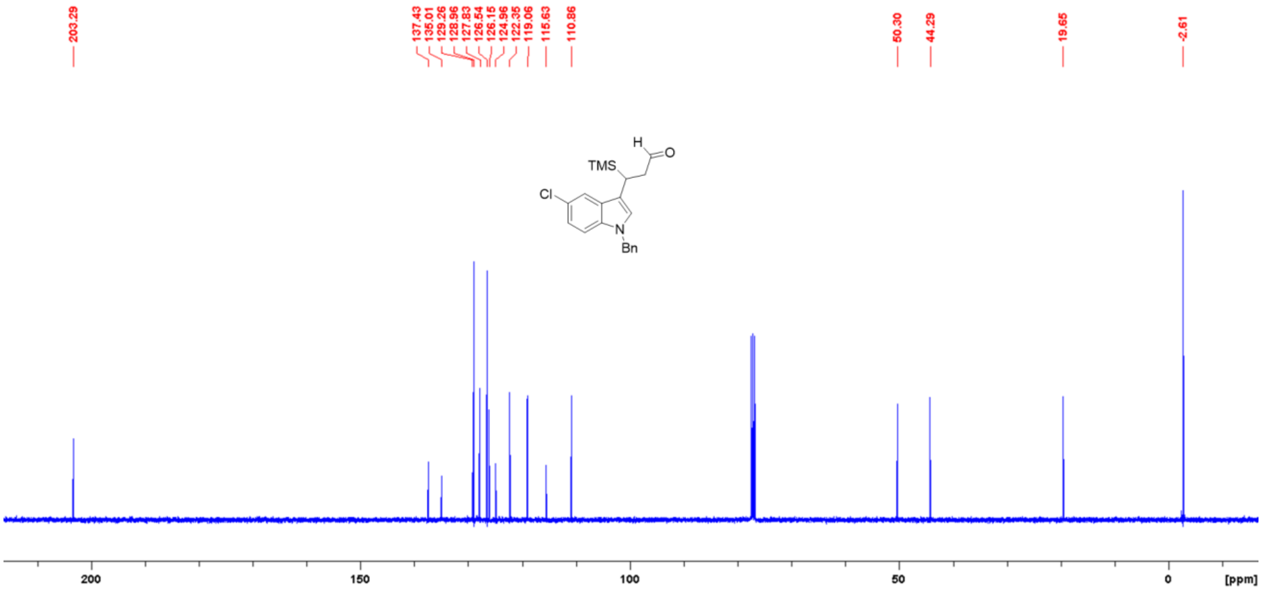

Supplementary Figure 128 ^{13}C NMR ($CDCl_3$, 100 MHz) spectrum of **34b**

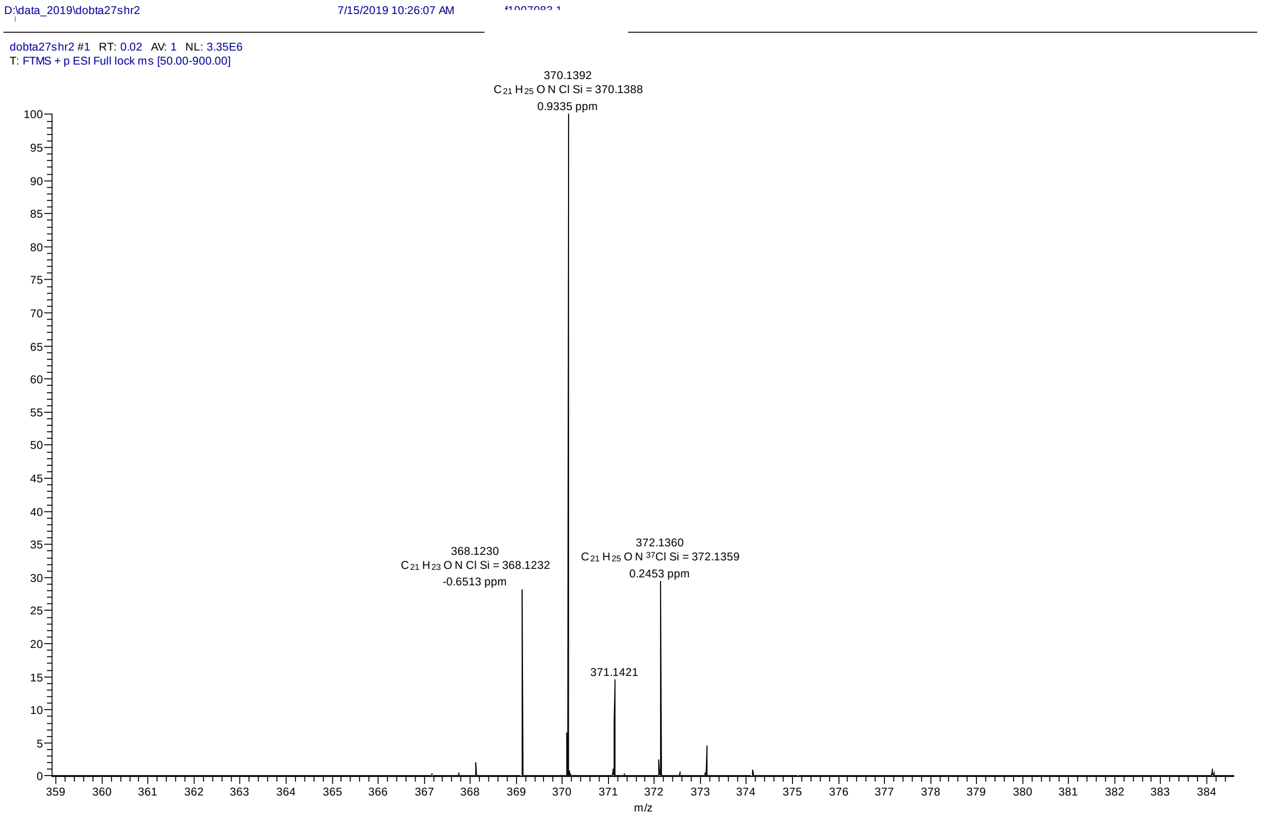

Supplementary Figure 129 ESI-MS spectra of **34b**

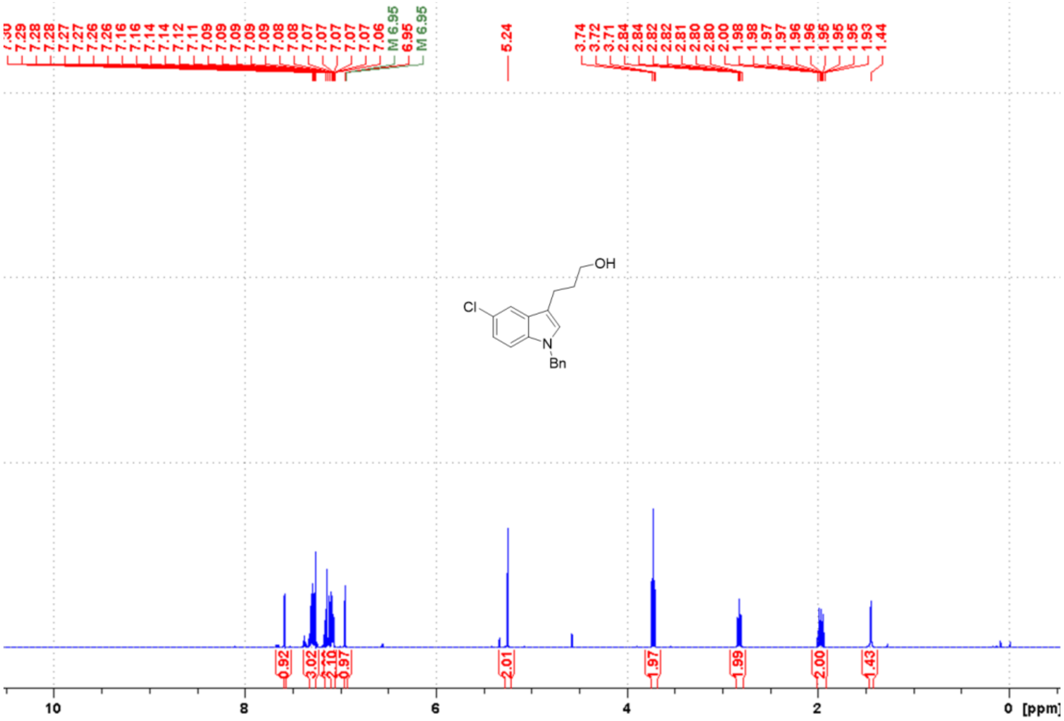

Supplementary Figure 130 ^{1}H NMR ($CDCl_3$, 400 MHz) spectrum of **35a**

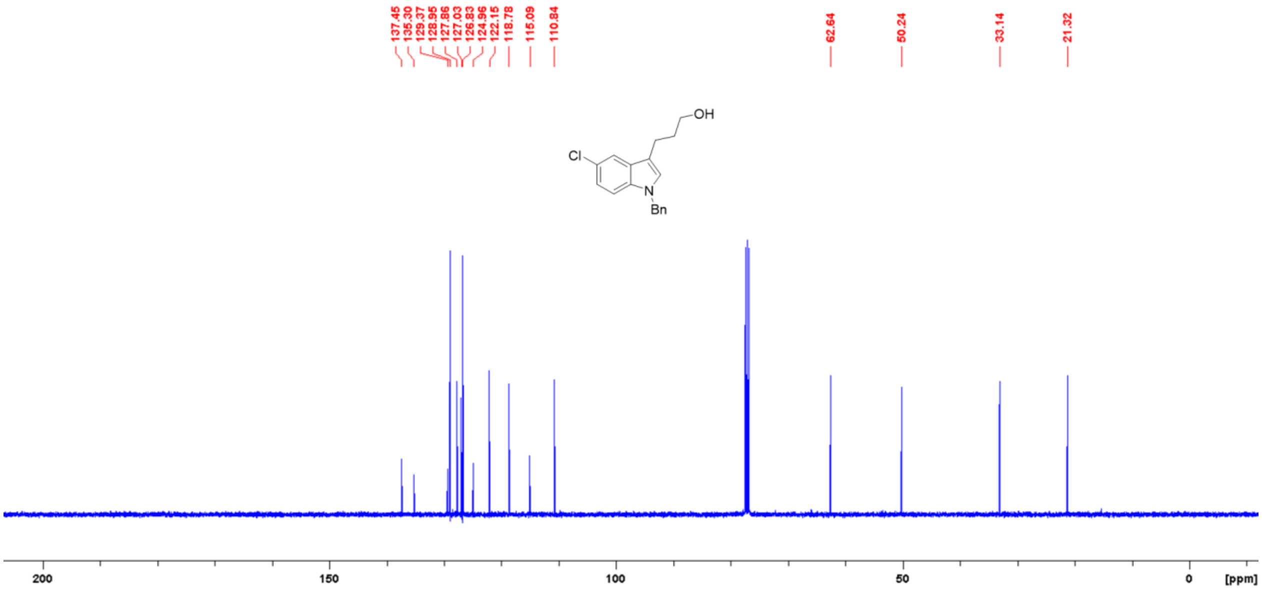

Supplementary Figure 131 ^{13}C NMR ($CDCl_3$, 100 MHz) spectrum of **35a**

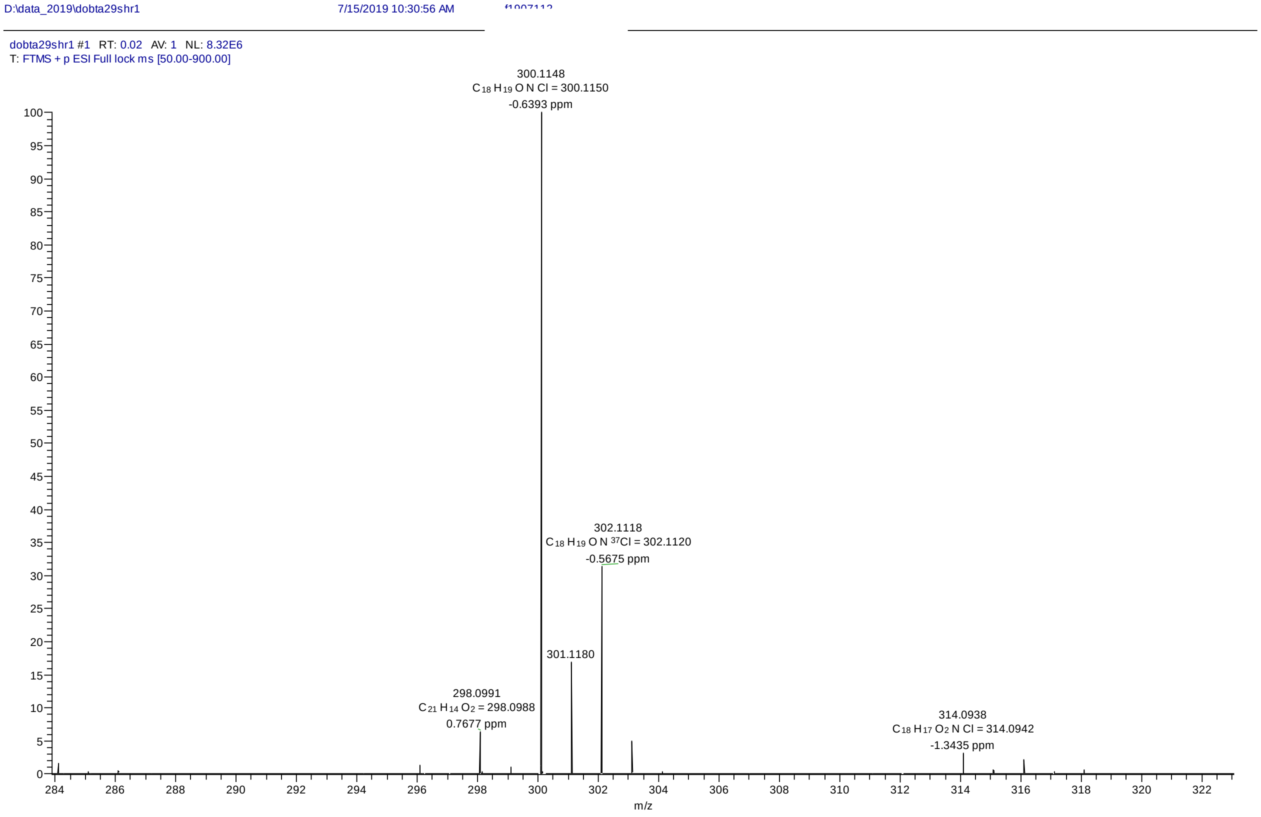

Supplementary Figure 132 ESI-MS spectra of **35a**

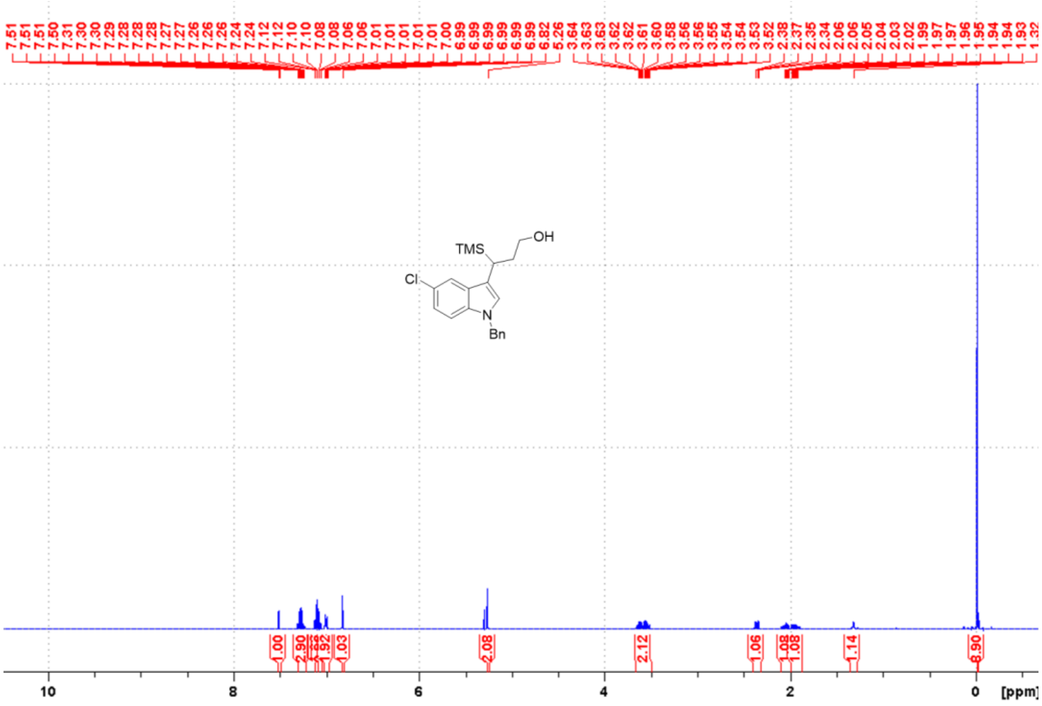

Supplementary Figure 133 ^{1}H NMR ($CDCl_3$, 400 MHz) spectrum of **35b**

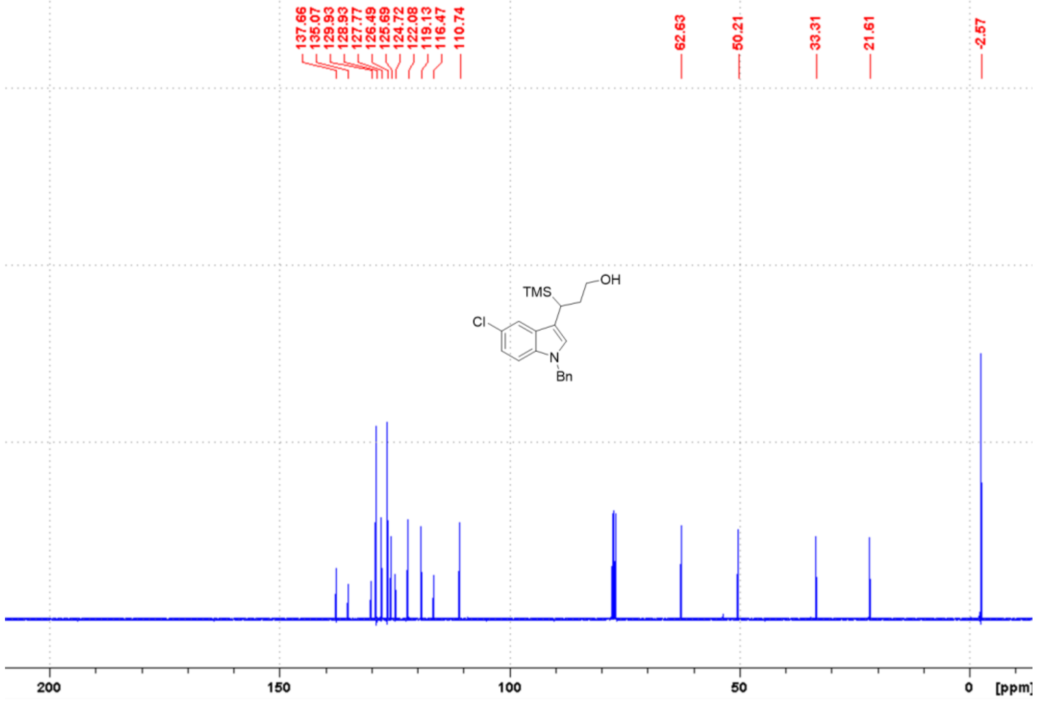

Supplementary Figure 134 ^{13}C NMR ($CDCl_3$, 100 MHz) spectrum of **35b**

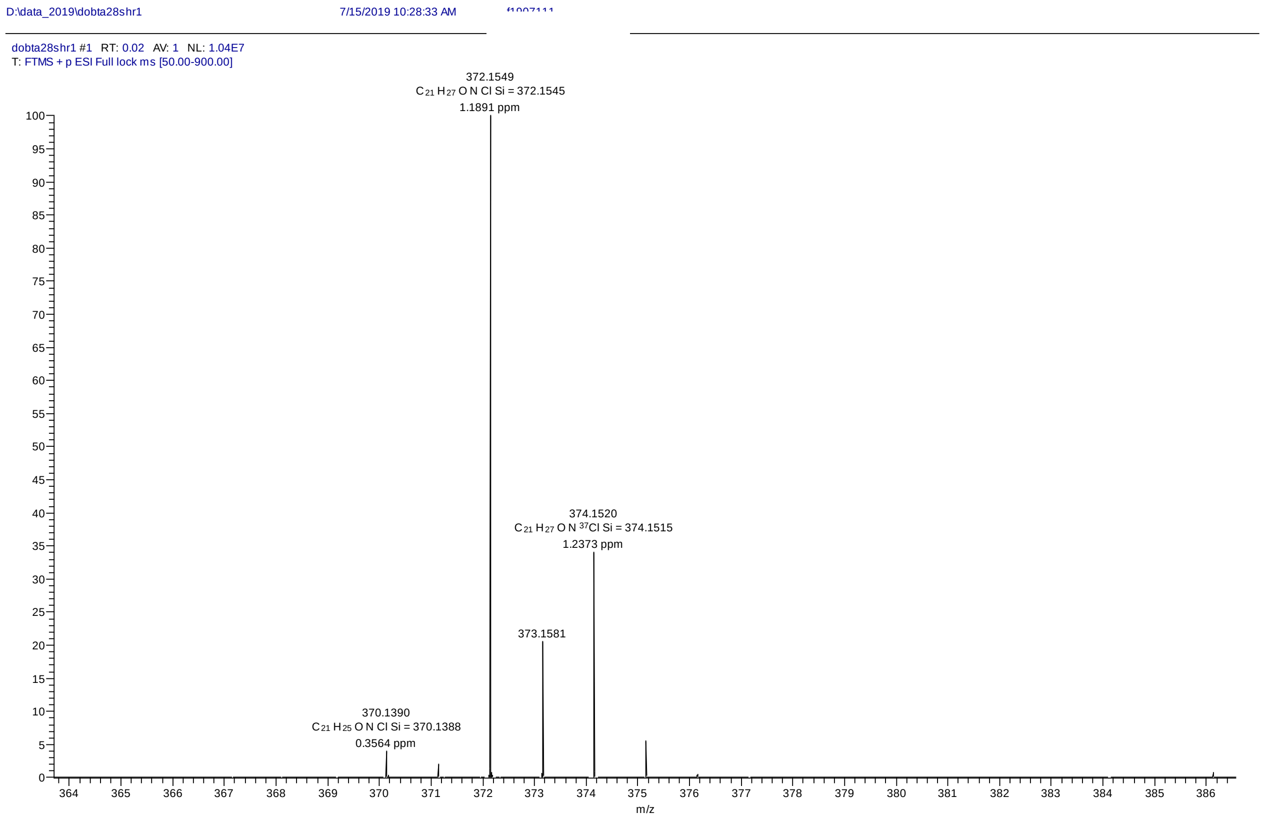

Supplementary Figure 135 ESI-MS spectra of **35b**

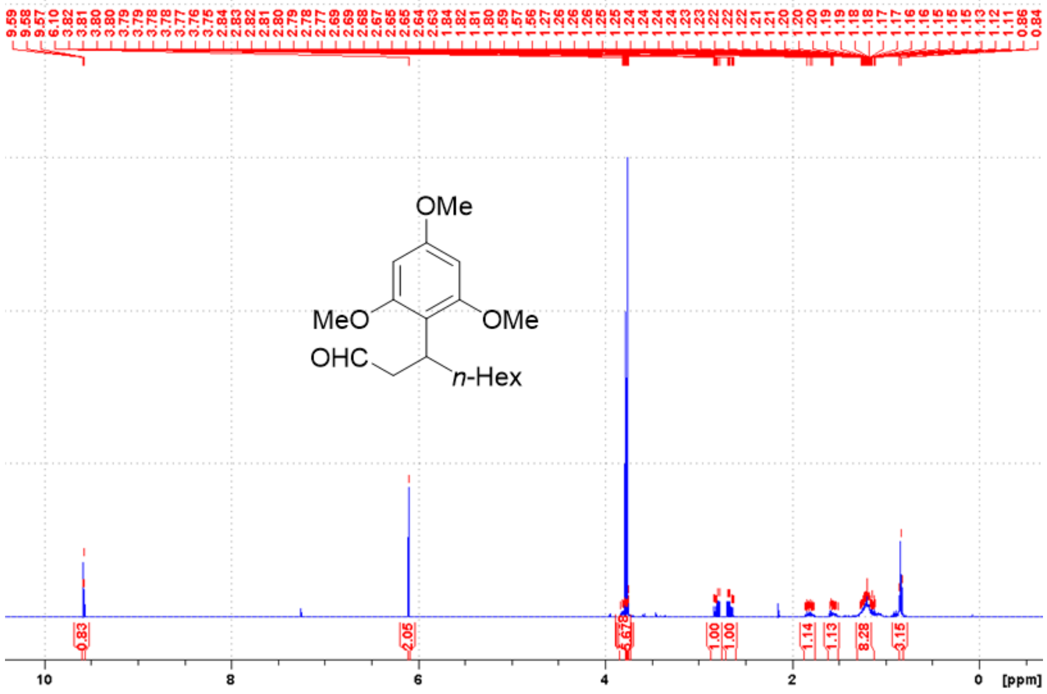

Supplementary Figure 136 ^{1}H NMR ($CDCl_3$, 400 MHz) spectrum of **6** formed from **S37** and TMB

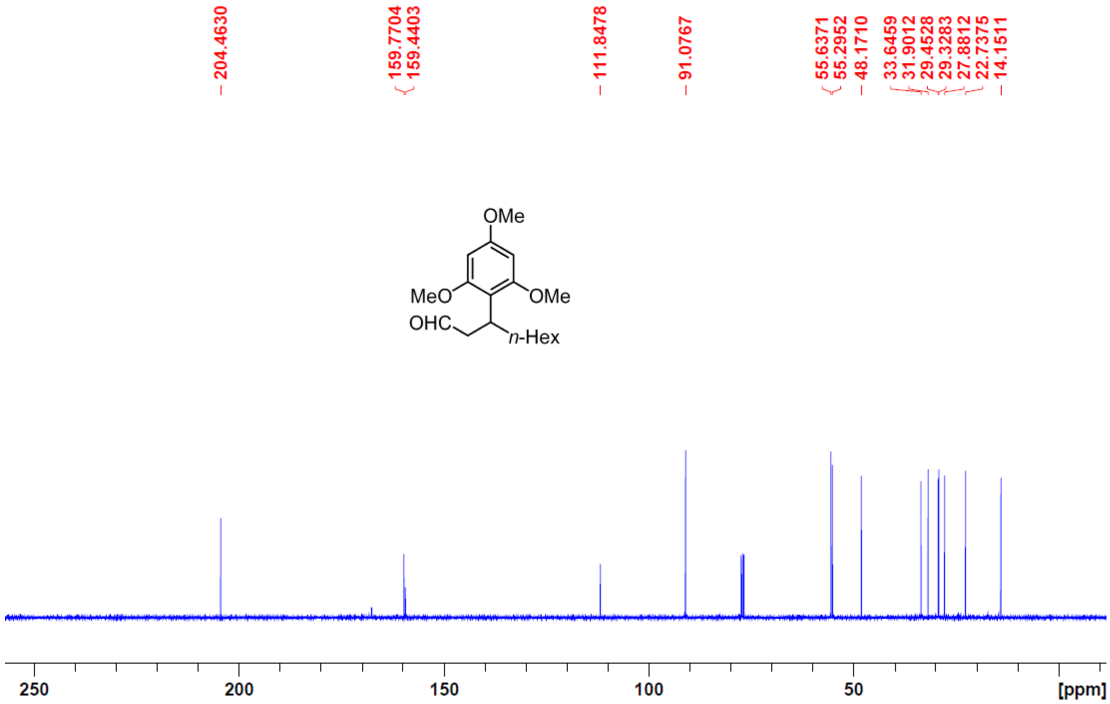

Supplementary Figure 137 ^{13}C NMR ($CDCl_3$, 100 MHz) spectrum of **6** formed from **S37** and TMB

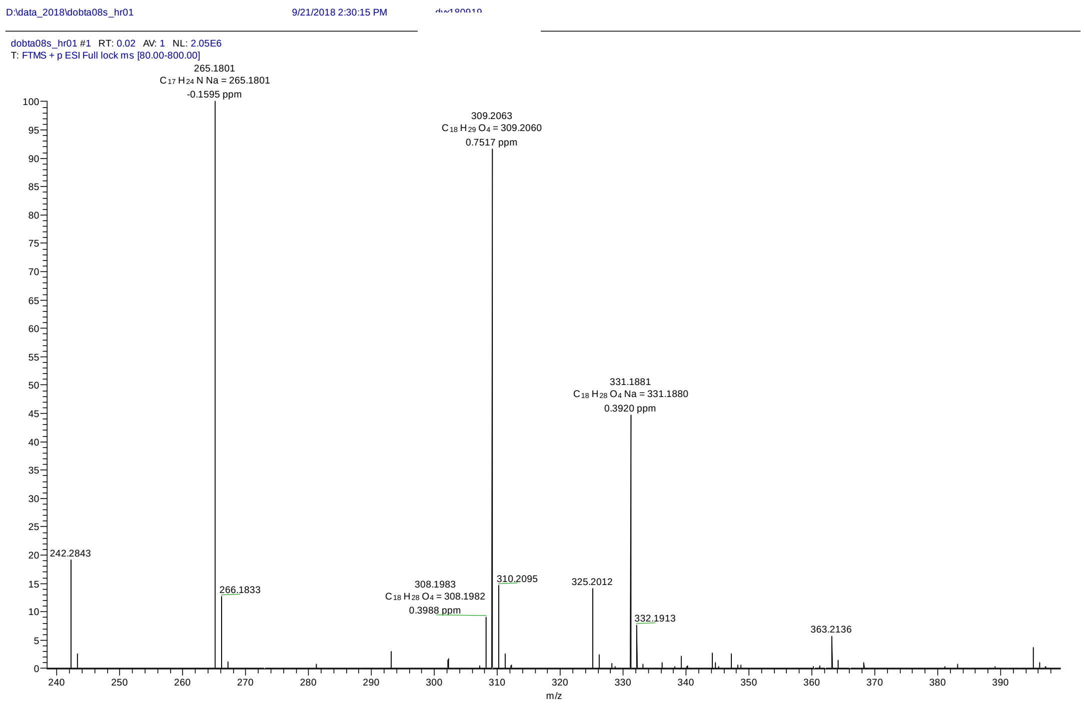

Supplementary Figure 138 ESI-MS spectra of **6** formed f from **S37** and TMB

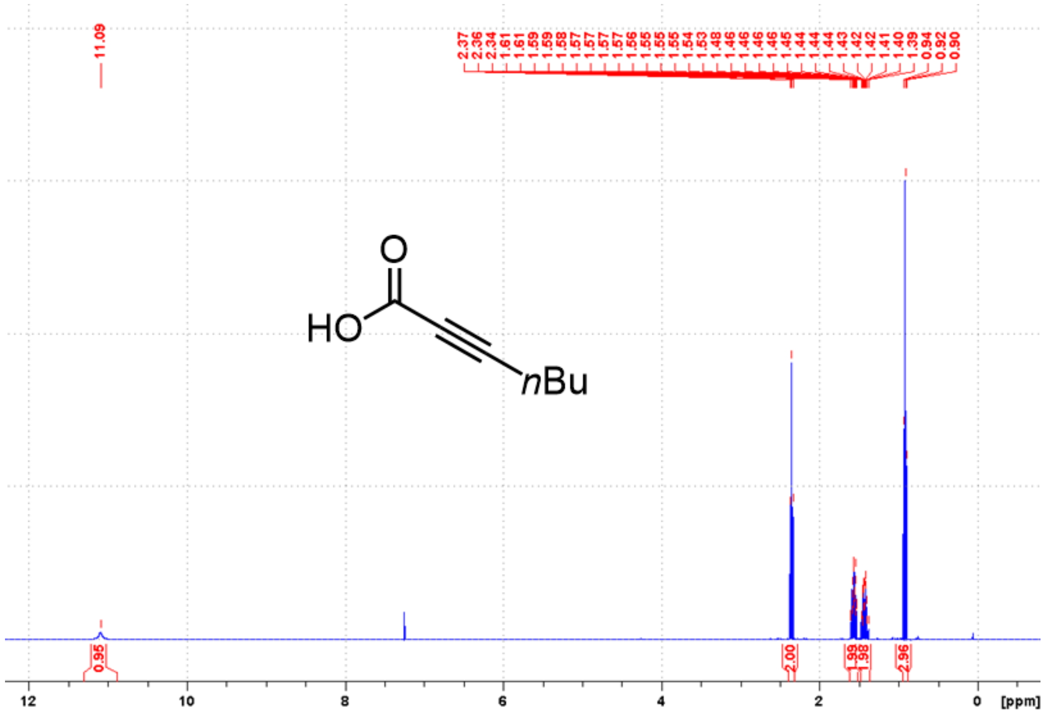

Supplementary Figure 139 ^{1}H NMR ($CDCl_3$, 400 MHz) spectrum of **S4**

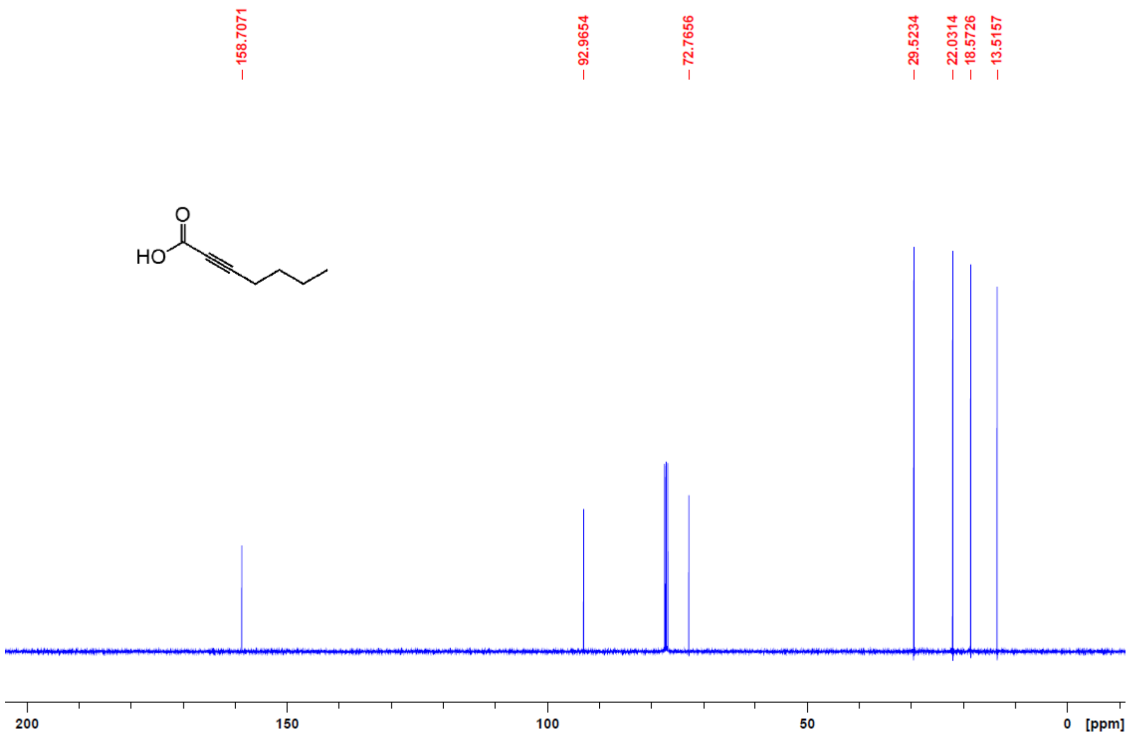

Supplementary Figure 140 ^{13}C NMR ($CDCl_3$, 100 MHz) spectrum of **S4**

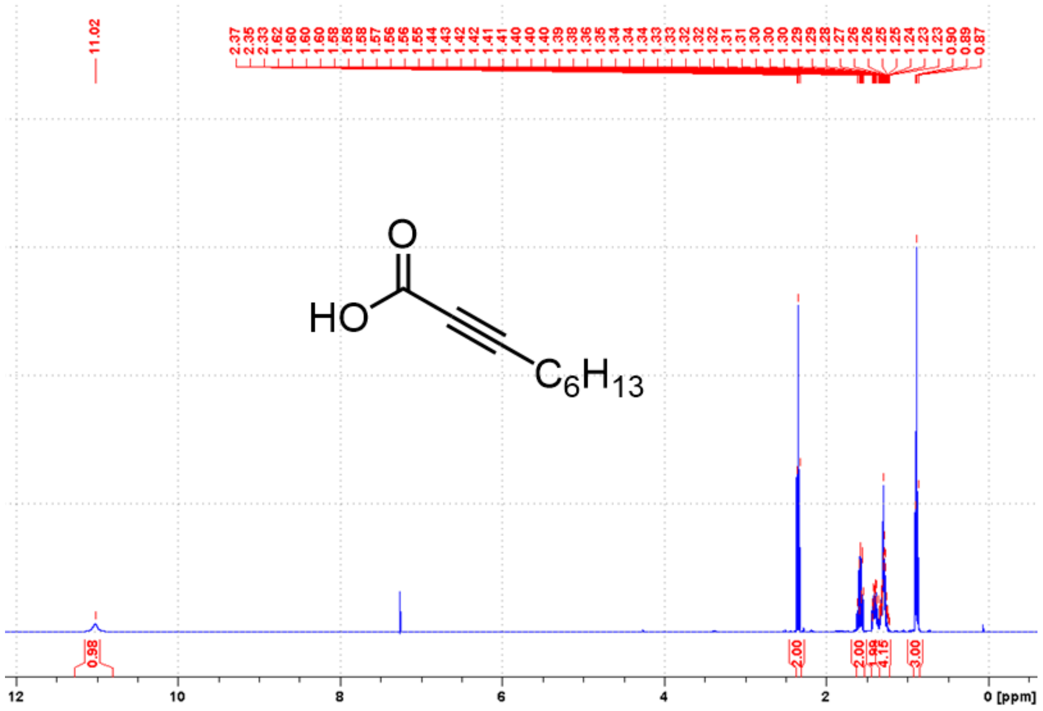

Supplementary Figure 141 ^{1}H NMR ($CDCl_3$, 400 MHz) spectrum of **S6**

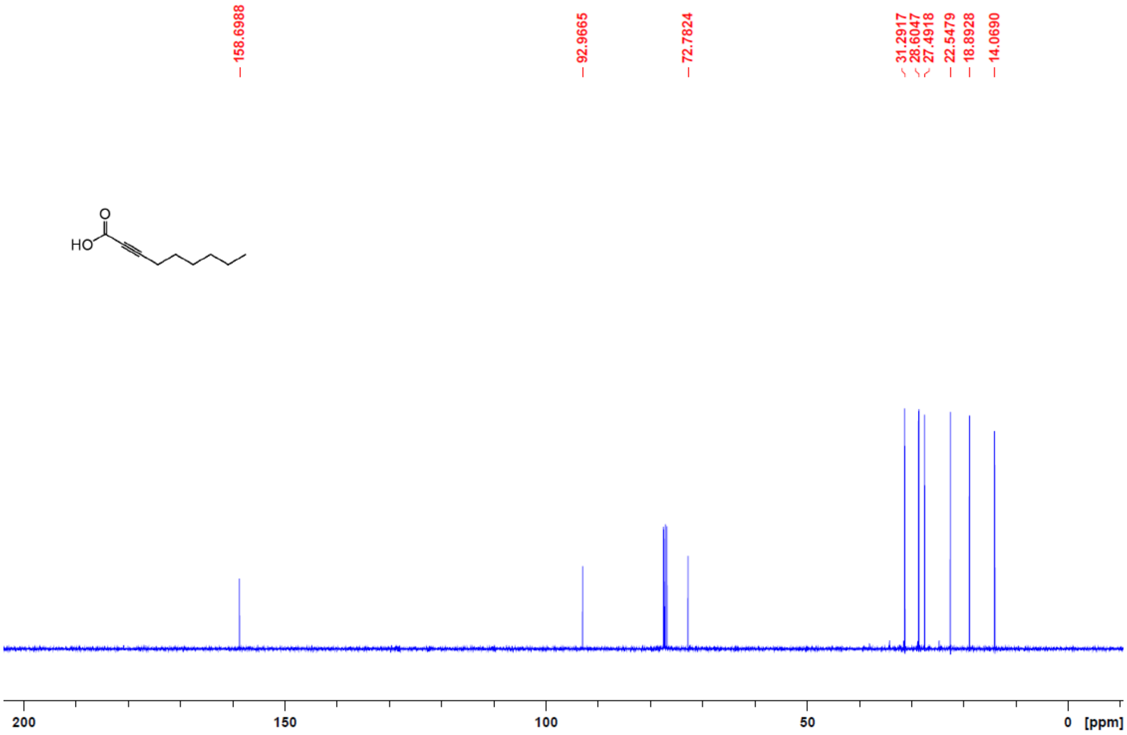

Supplementary Figure 142 ^{13}C NMR ($CDCl_3$, 100 MHz) spectrum of **S6**

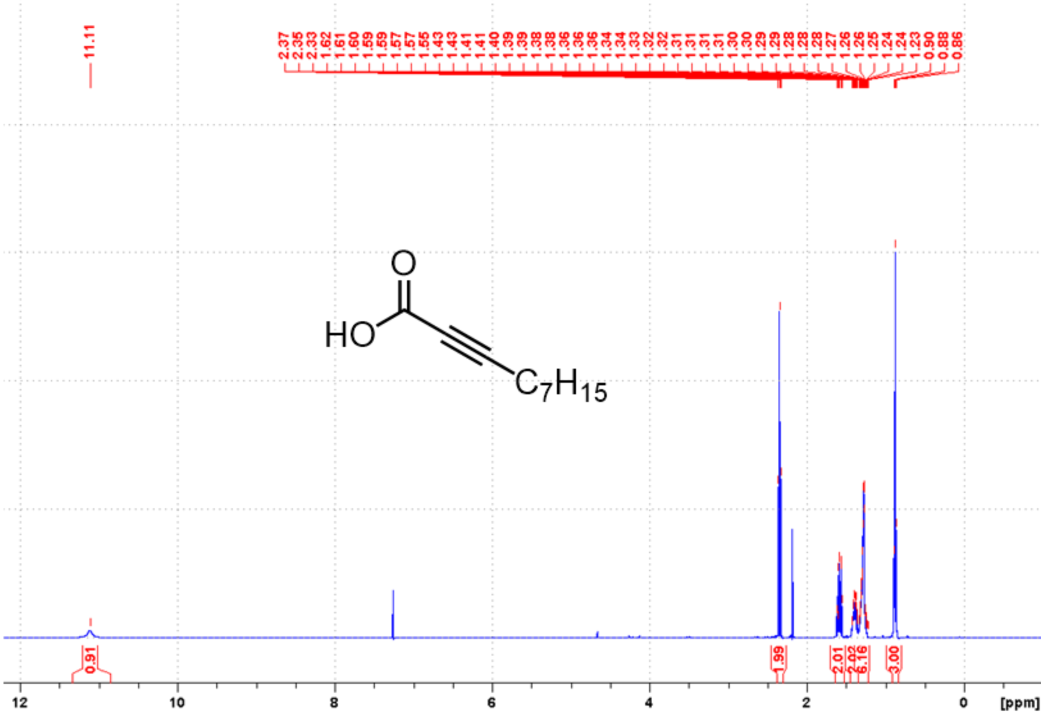

Supplementary Figure 143 ^{1}H NMR ($CDCl_3$, 400 MHz) spectrum of **S7**

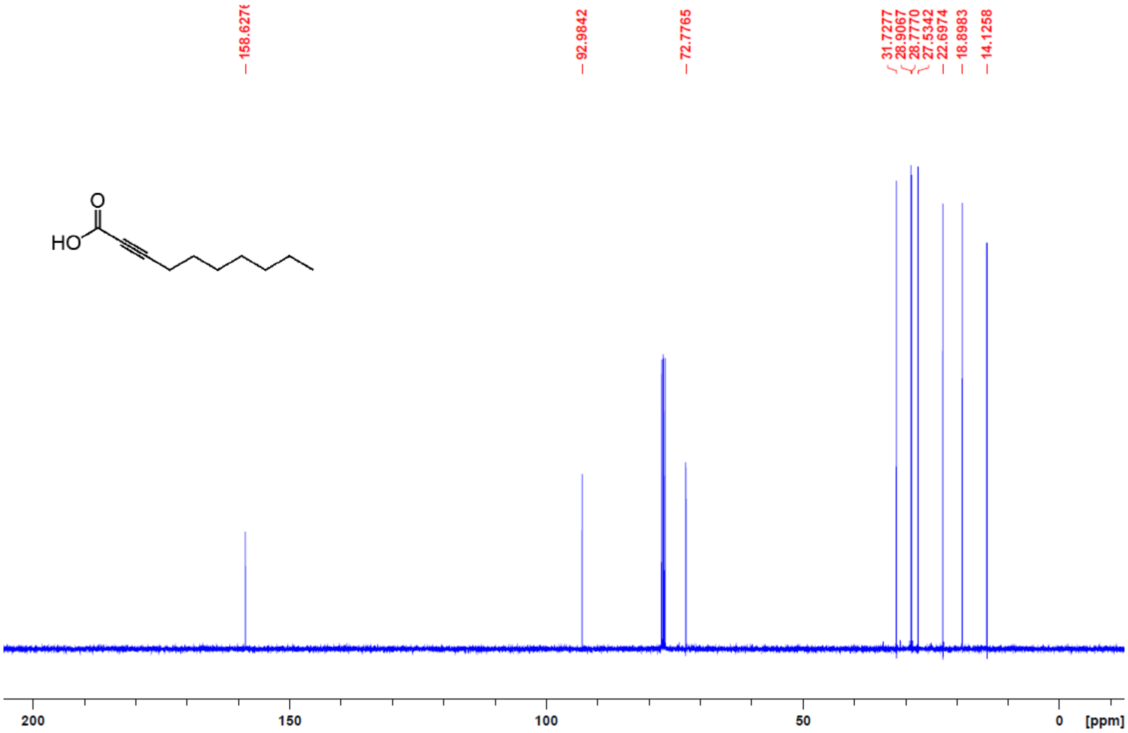

Supplementary Figure 144 ^{13}C NMR ($CDCl_3$, 100 MHz) spectrum of **S7**

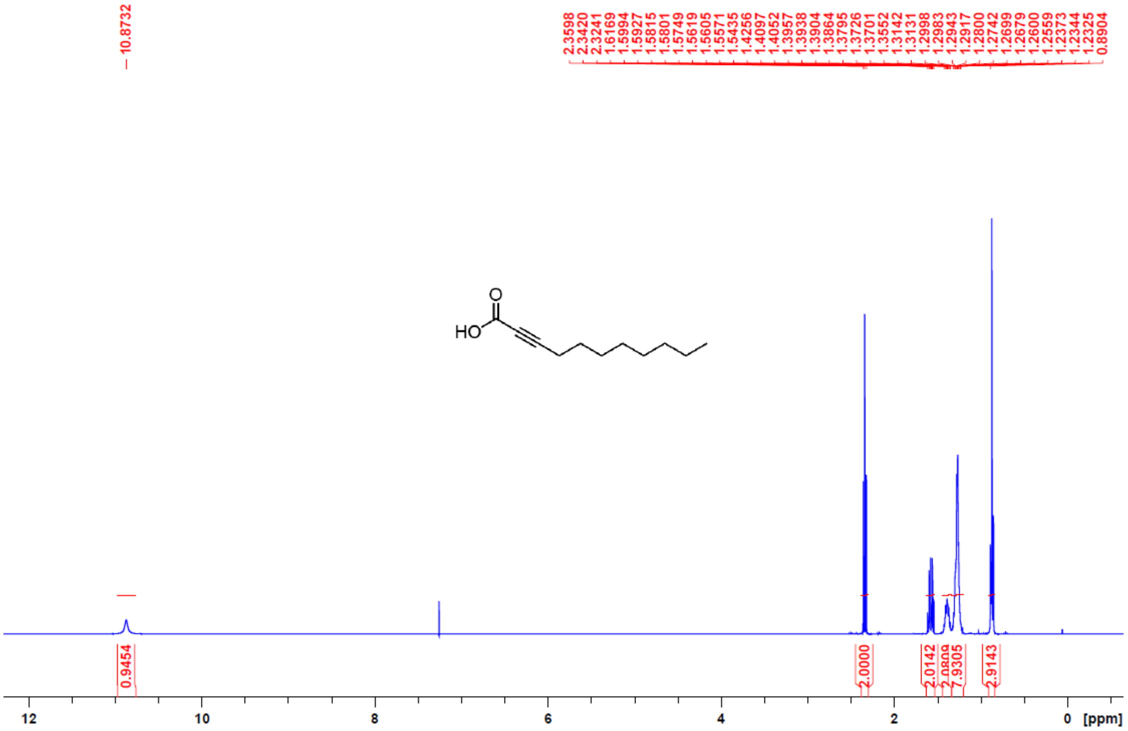

Supplementary Figure 145 ^{1}H NMR ($CDCl_3$, 400 MHz) spectrum of **S8**

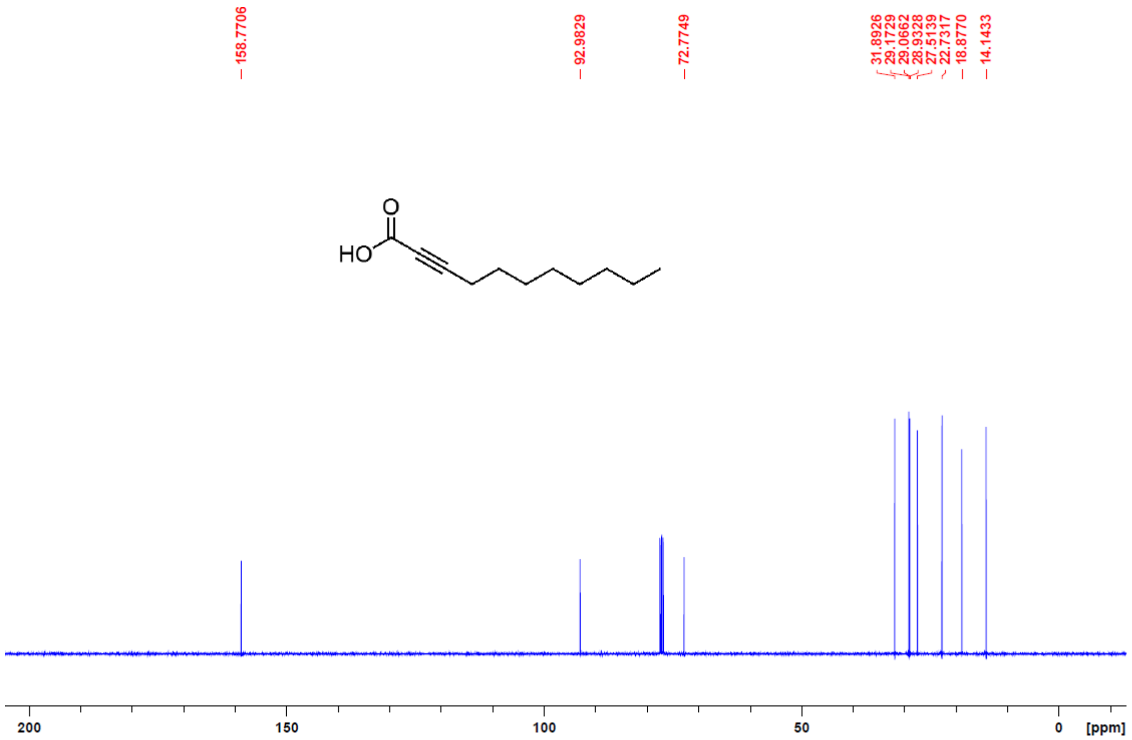

Supplementary Figure 146 ^{13}C NMR ($CDCl_3$, 100 MHz) spectrum of **S8**

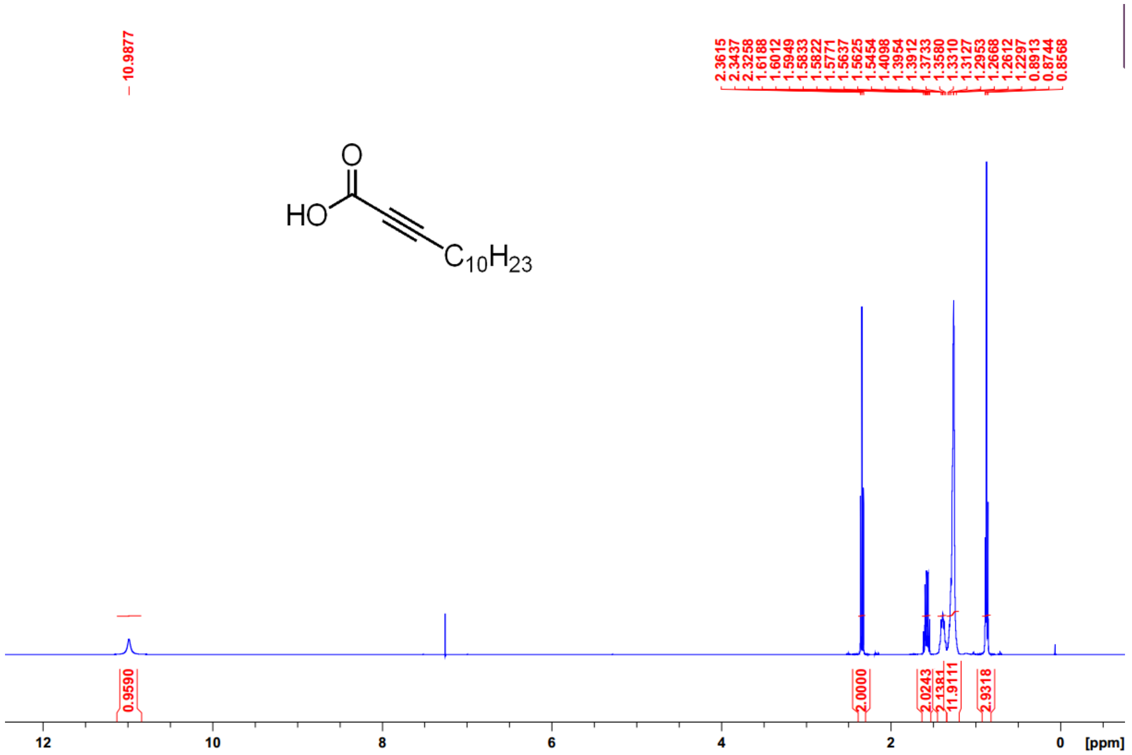

Supplementary Figure 147 ^{1}H NMR ($CDCl_3$, 400 MHz) spectrum of **S9**

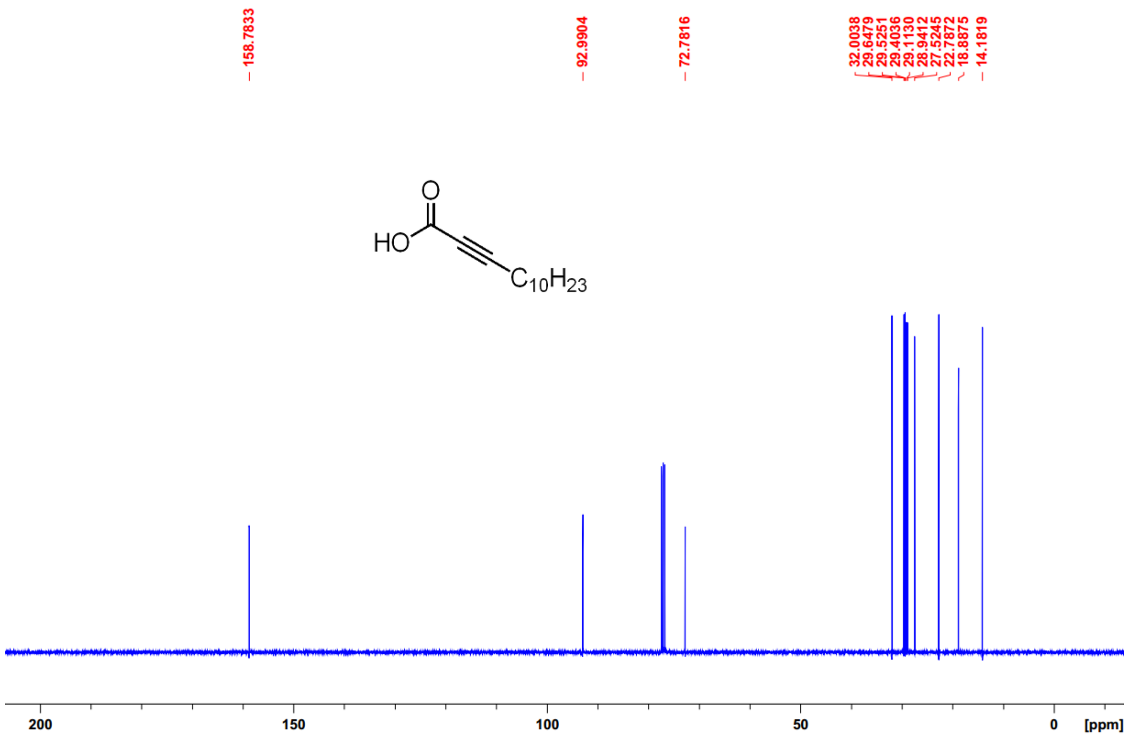

Supplementary Figure 148 ^{13}C NMR ($CDCl_3$, 100 MHz) spectrum of **S9**

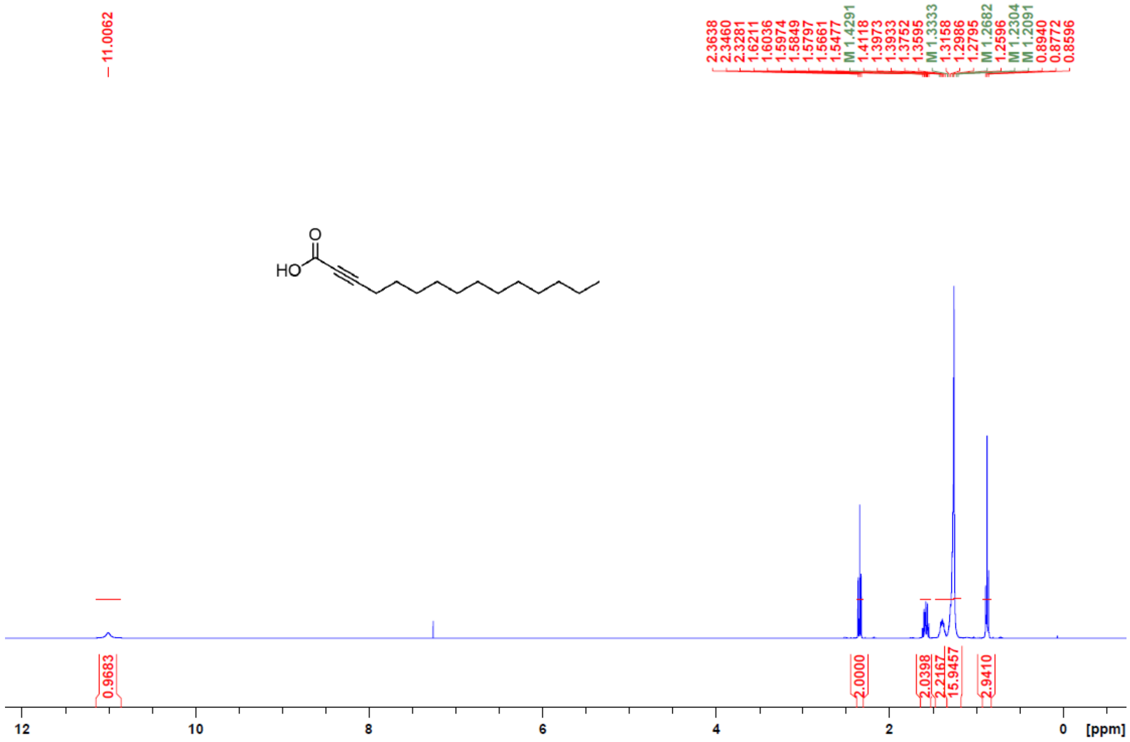

Supplementary Figure 149 ^{1}H NMR ($CDCl_3$, 400 MHz) spectrum of **S10**

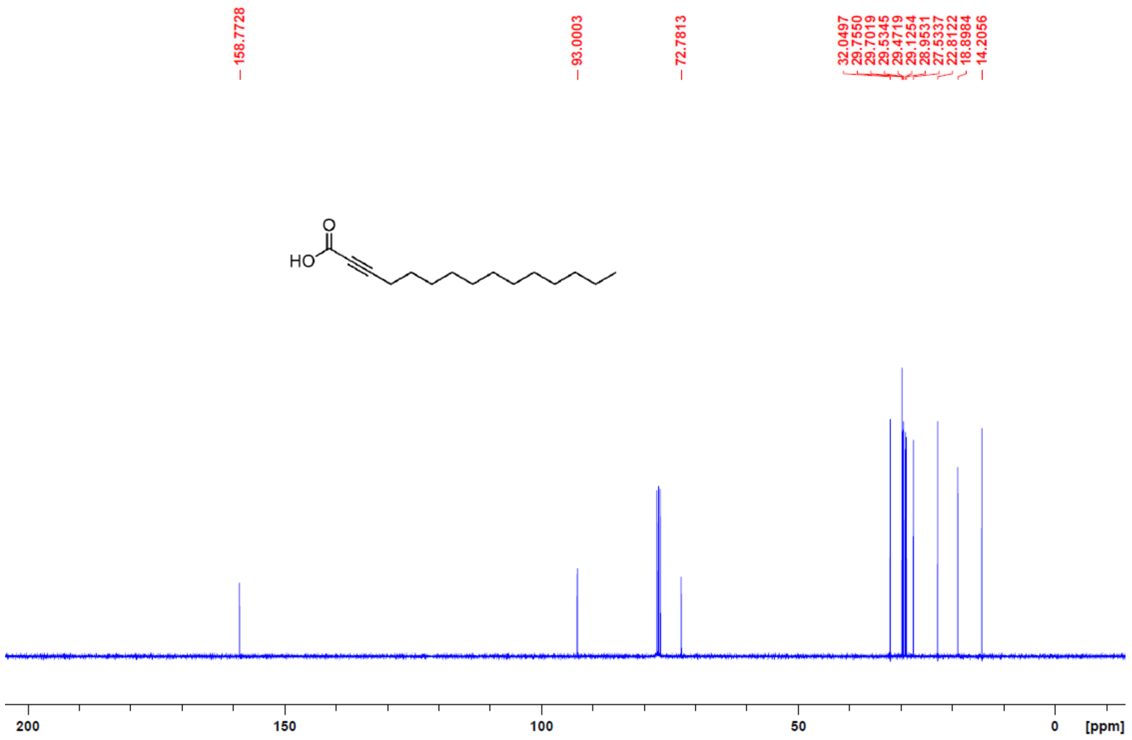

Supplementary Figure 150 ^{13}C NMR ($CDCl_3$, 100 MHz) spectrum of **S10**

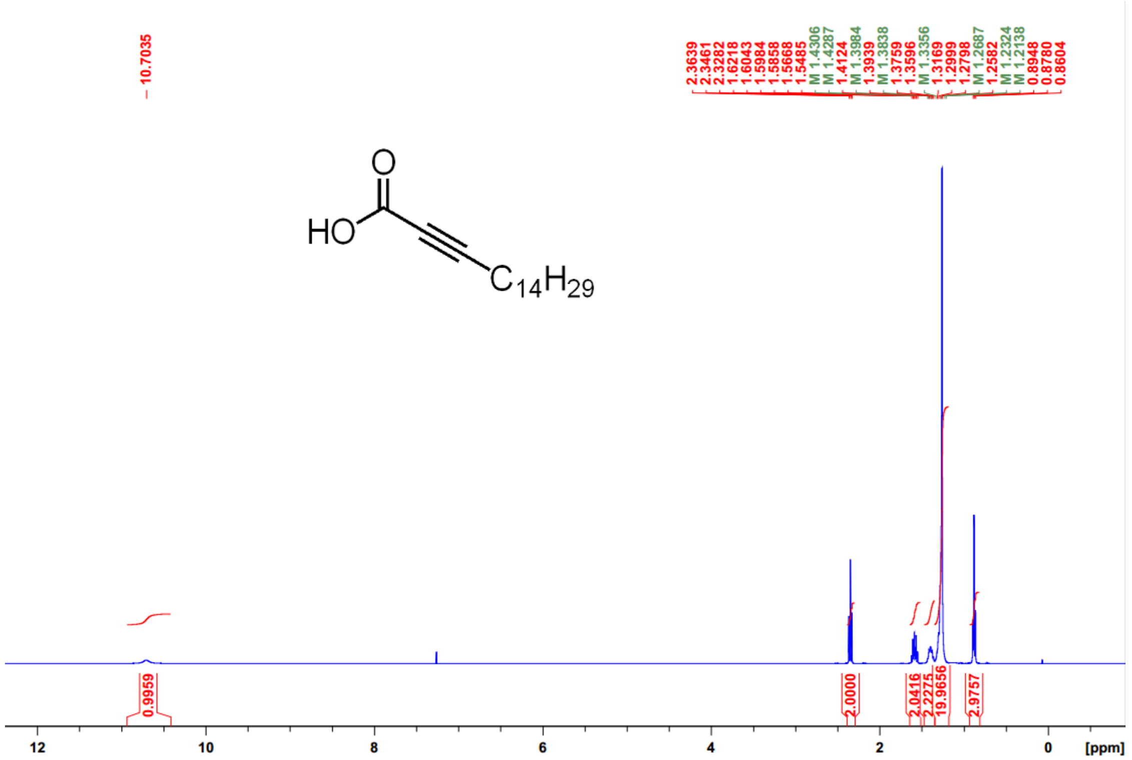

Supplementary Figure 151 ^{1}H NMR ($CDCl_3$, 400 MHz) spectrum of **S11**

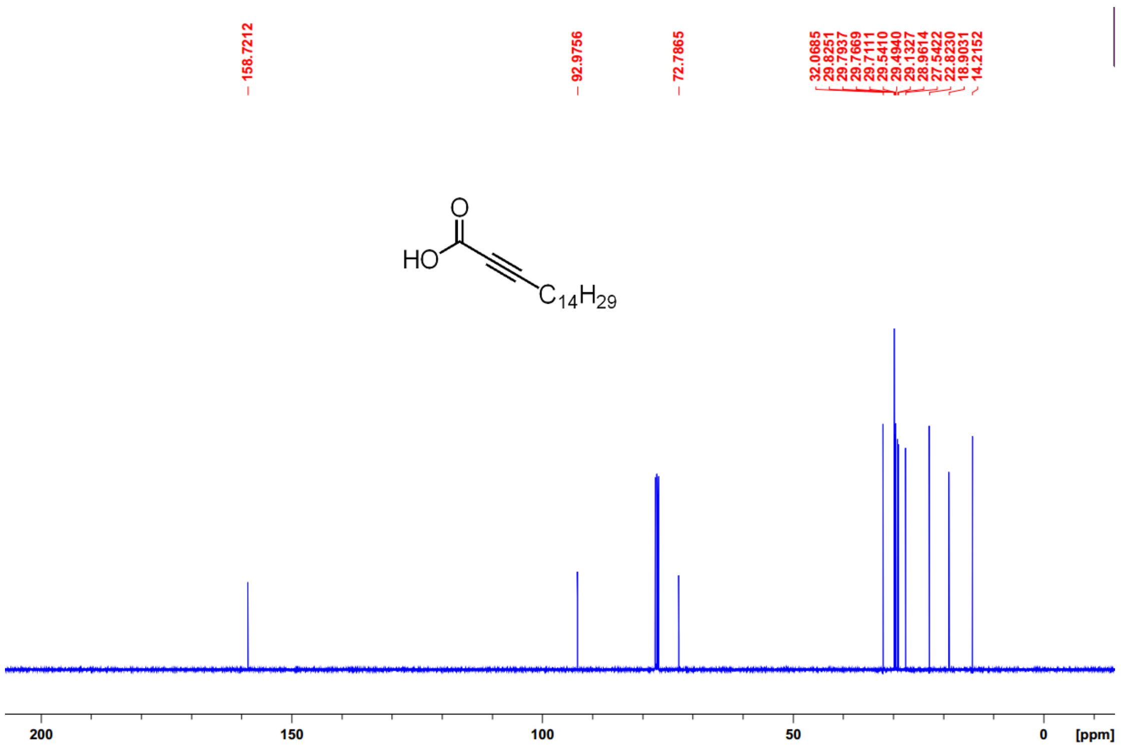

Supplementary Figure 152 ^{13}C NMR ($CDCl_3$, 100 MHz) spectrum of **S11**

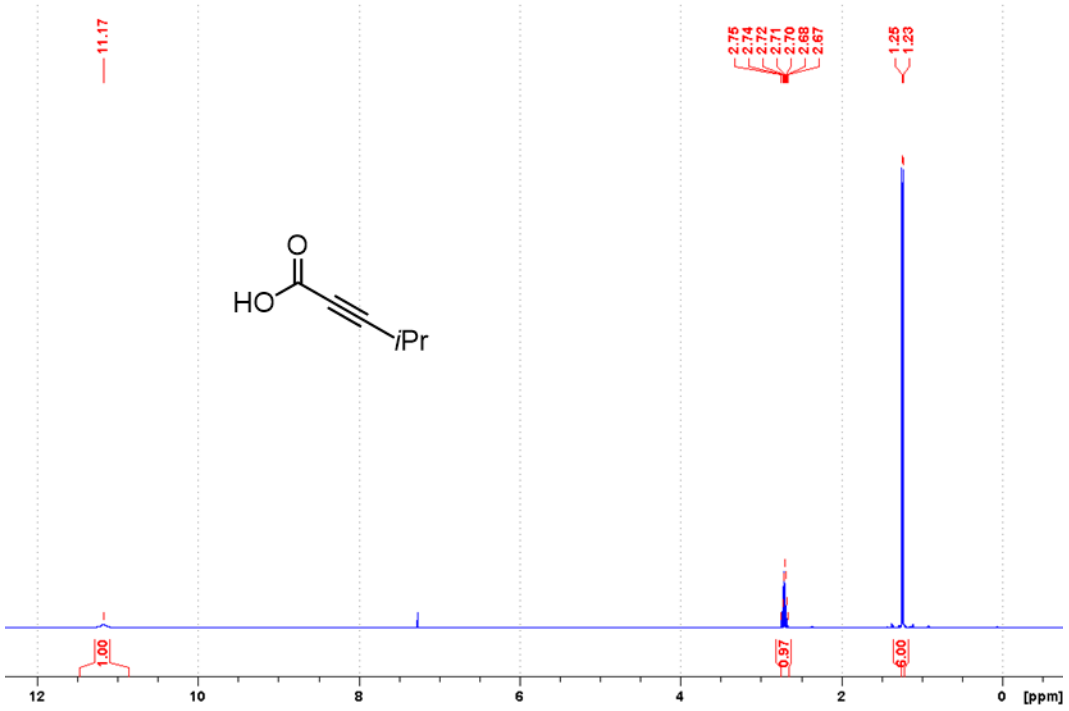

Supplementary Figure 153 ^{1}H NMR ($CDCl_3$, 500 MHz) spectrum of **S12**

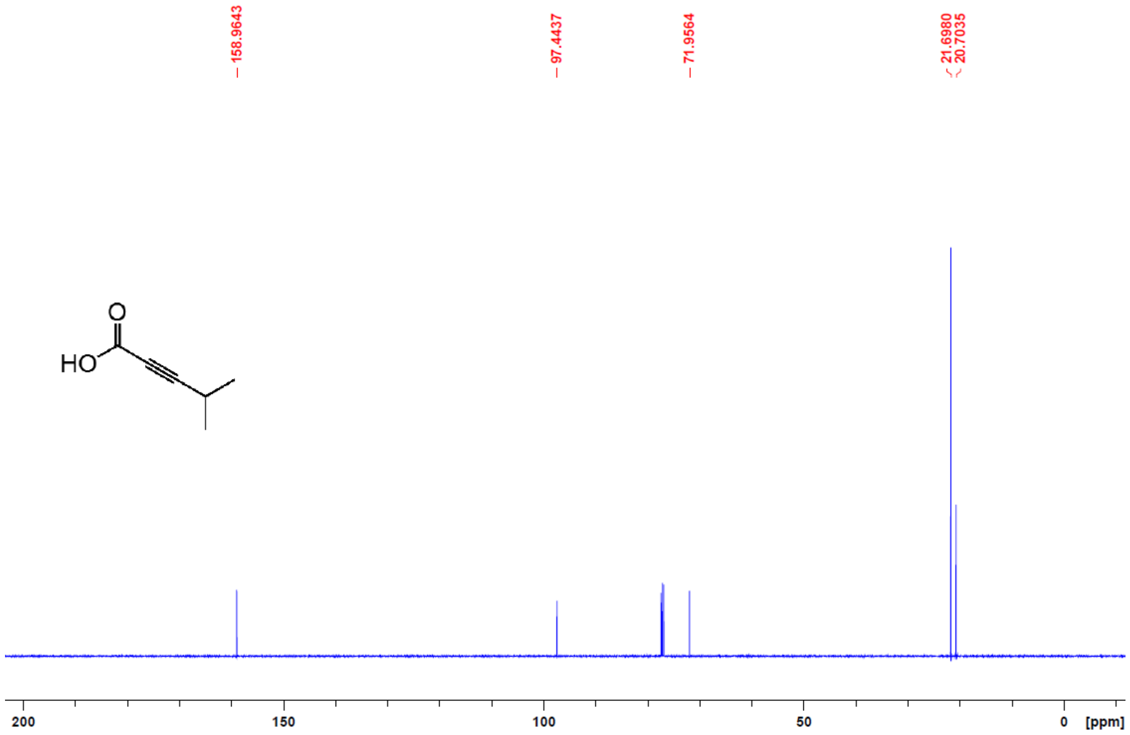

Supplementary Figure 154 ^{13}C NMR ($CDCl_3$, 126 MHz) spectrum of **S12**

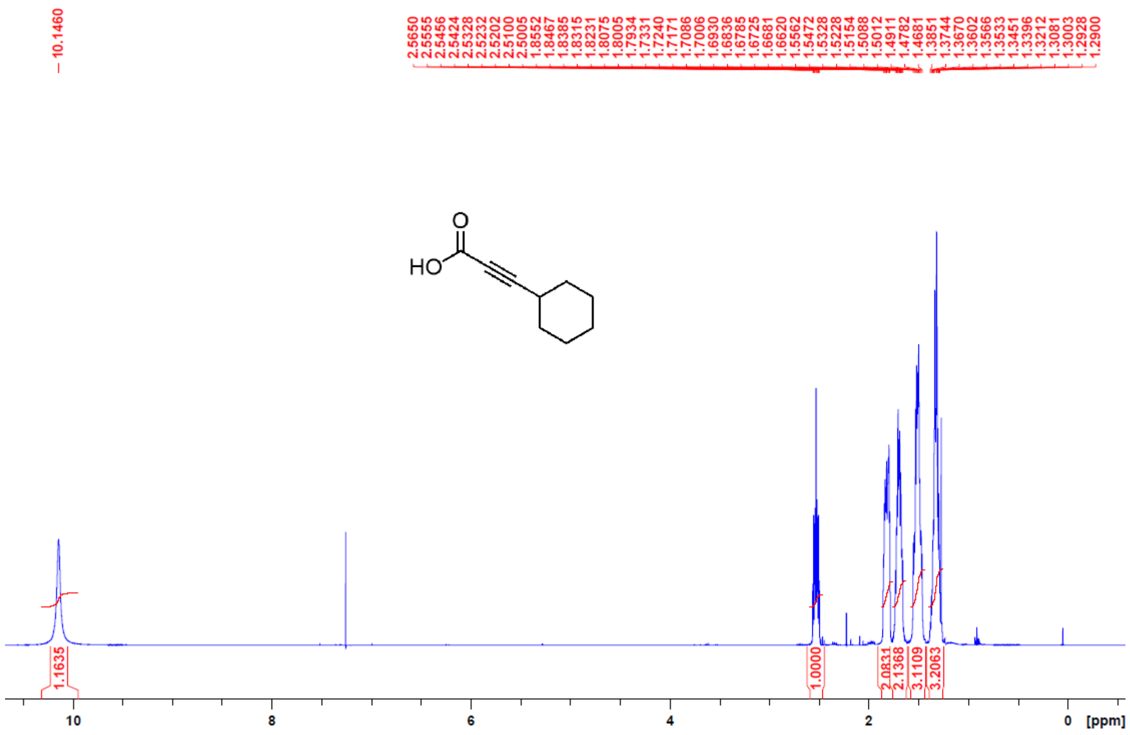

Supplementary Figure 155 ^{1}H NMR ($CDCl_3$, 400 MHz) spectrum of **S13**

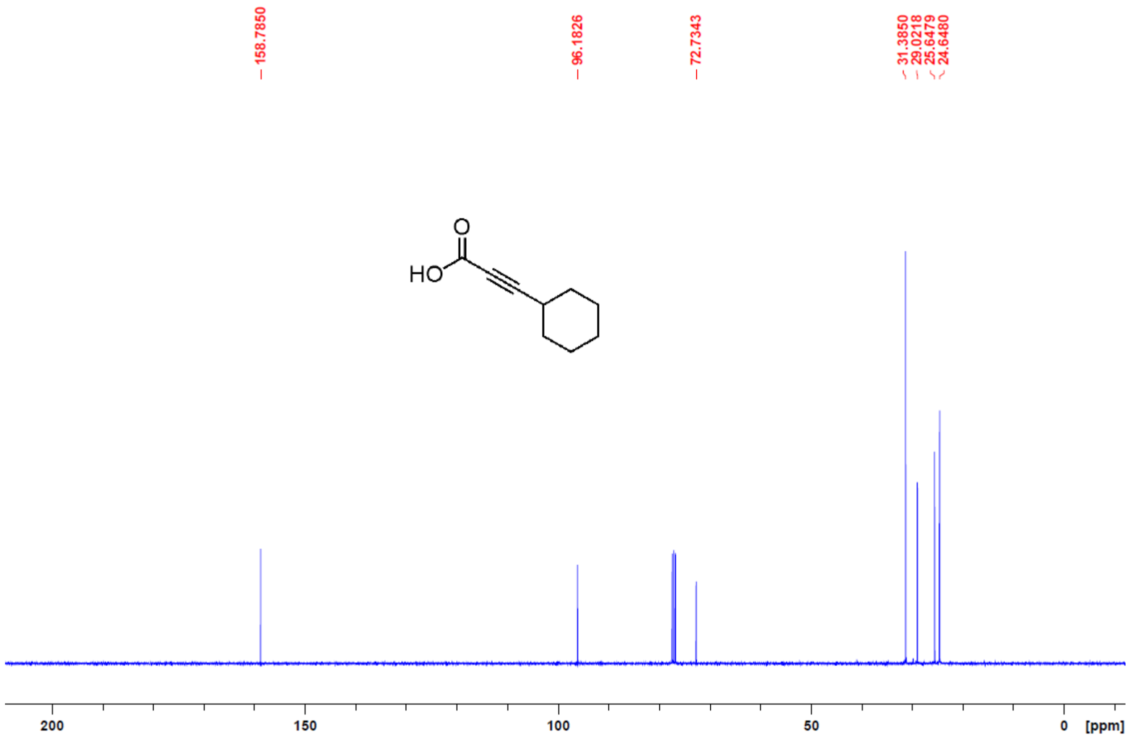

Supplementary Figure 156 ^{13}C NMR ($CDCl_3$, 100 MHz) spectrum of **S13**

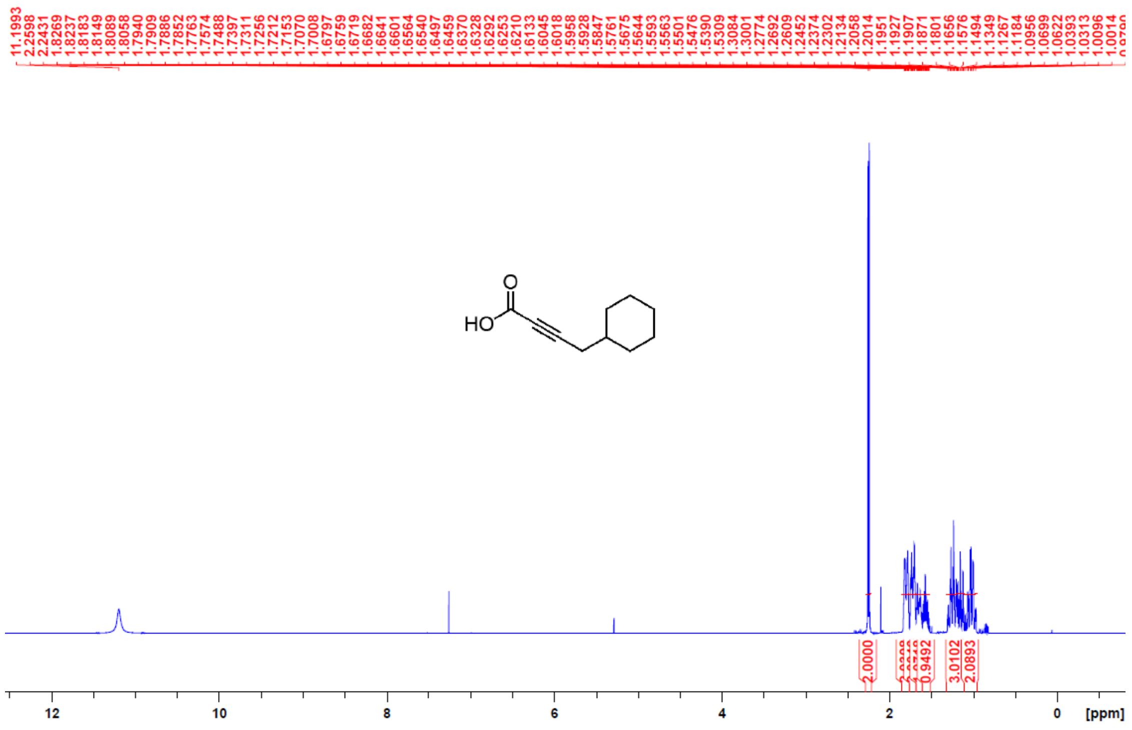

Supplementary Figure 157 ^{1}H NMR ($CDCl_3$, 400 MHz) spectrum of **S14**

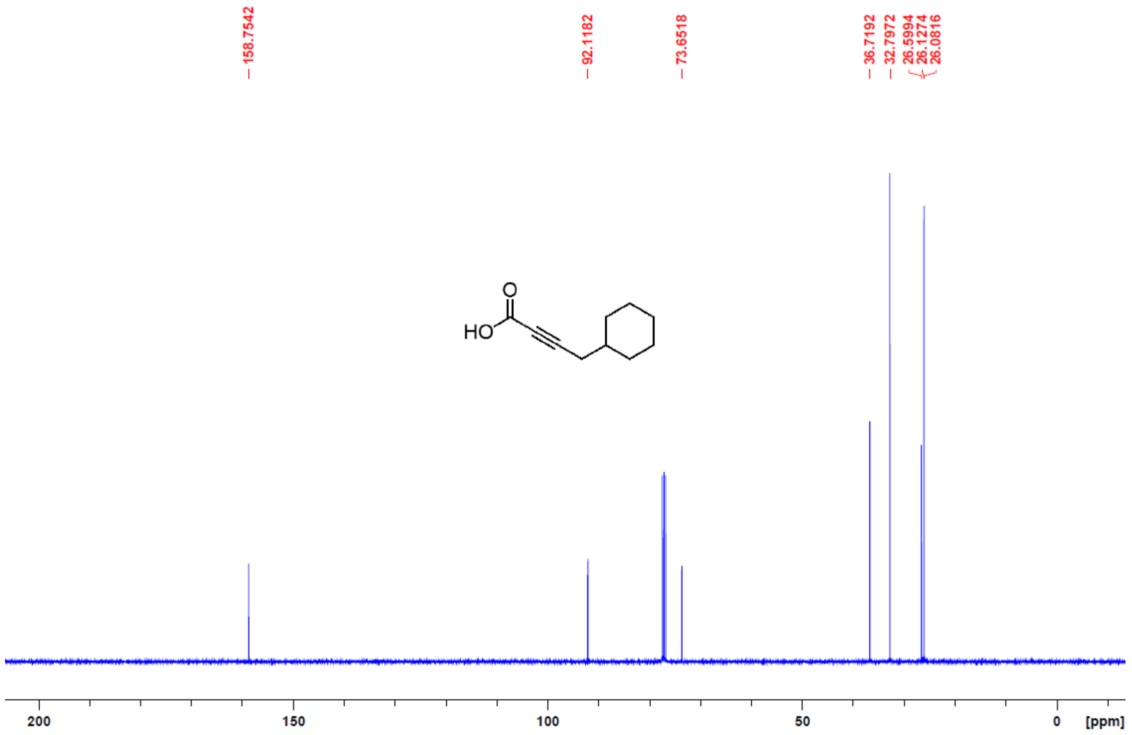

Supplementary Figure 158 ^{13}C NMR ($CDCl_3$, 100 MHz) spectrum of **S14**

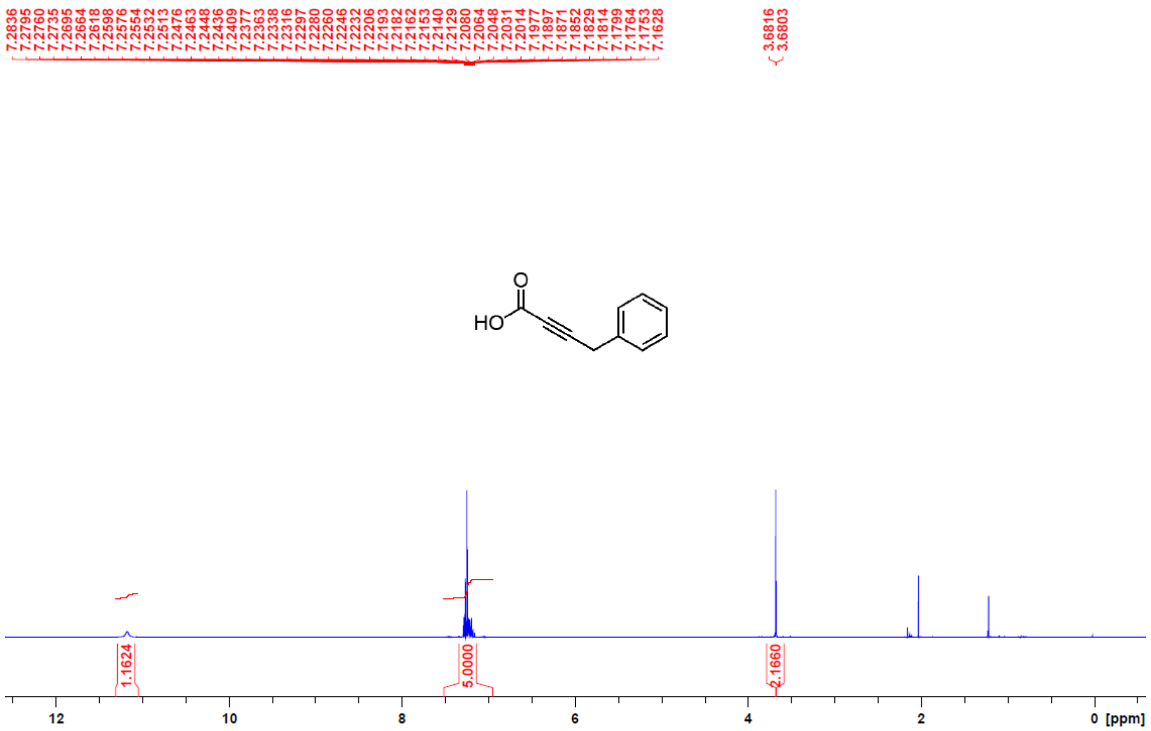

Supplementary Figure 159 ^{1}H NMR ($CDCl_3$, 400 MHz) spectrum of **S15**

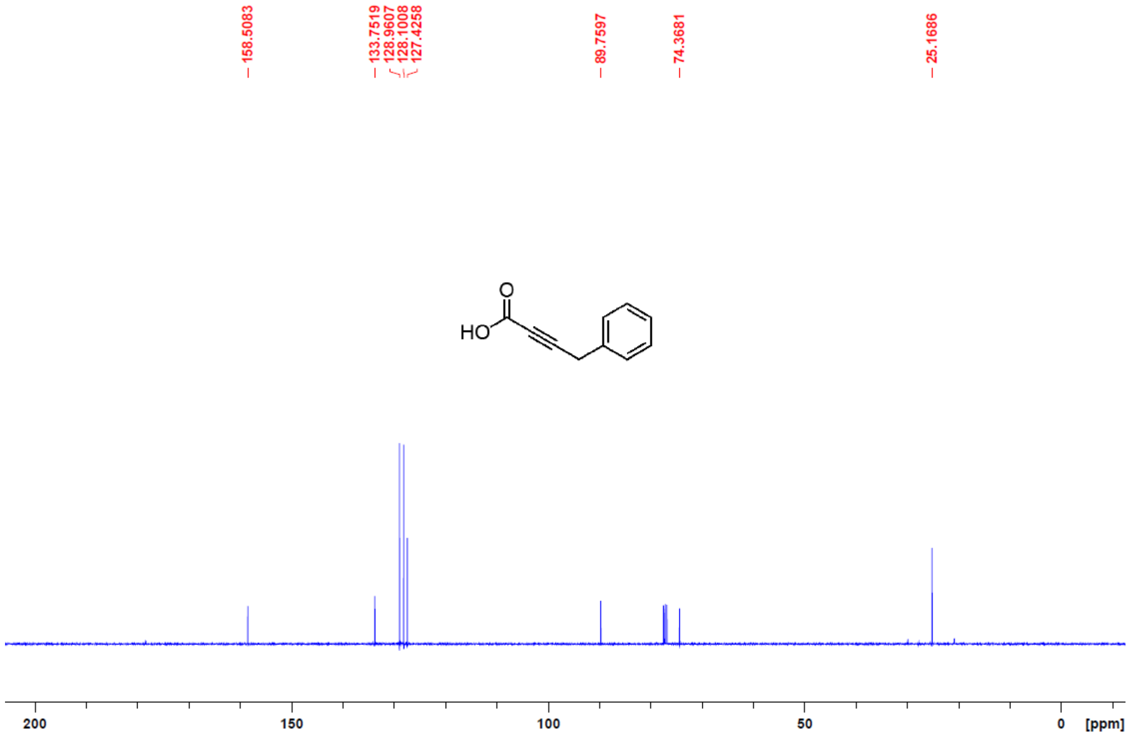

Supplementary Figure 160 ^{13}C NMR ($CDCl_3$, 100 MHz) spectrum of **S15**

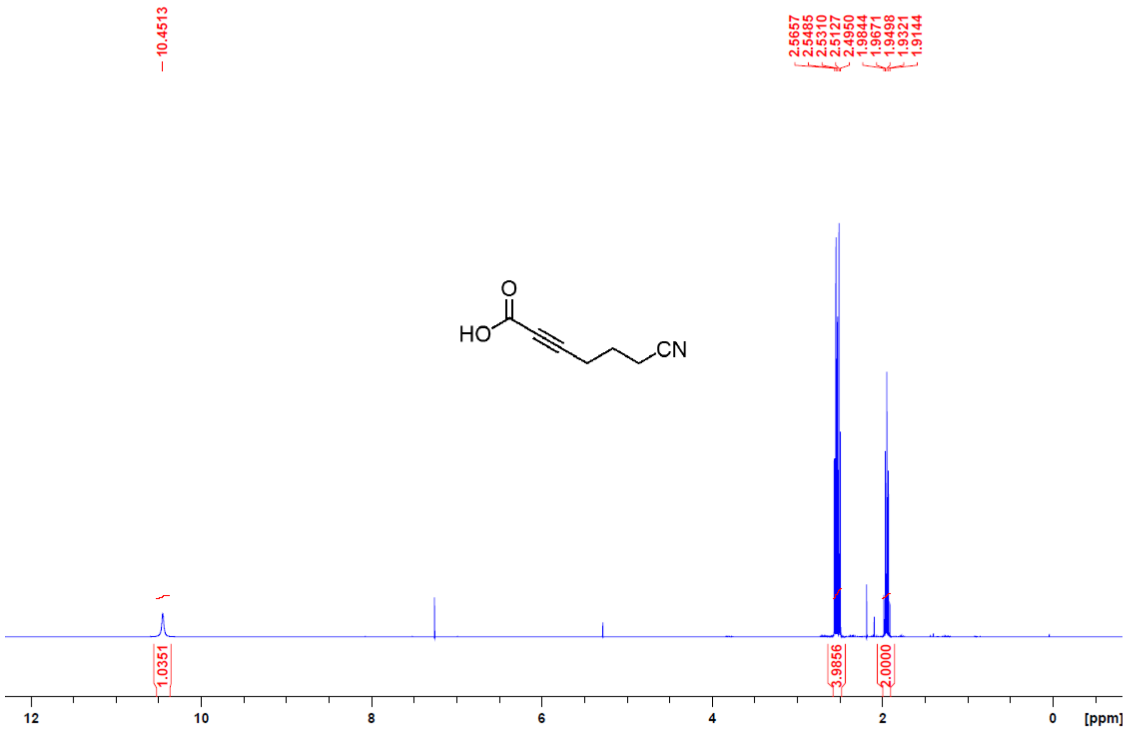

Supplementary Figure 161 ^{1}H NMR ($CDCl_3$, 400 MHz) spectrum of **S16**

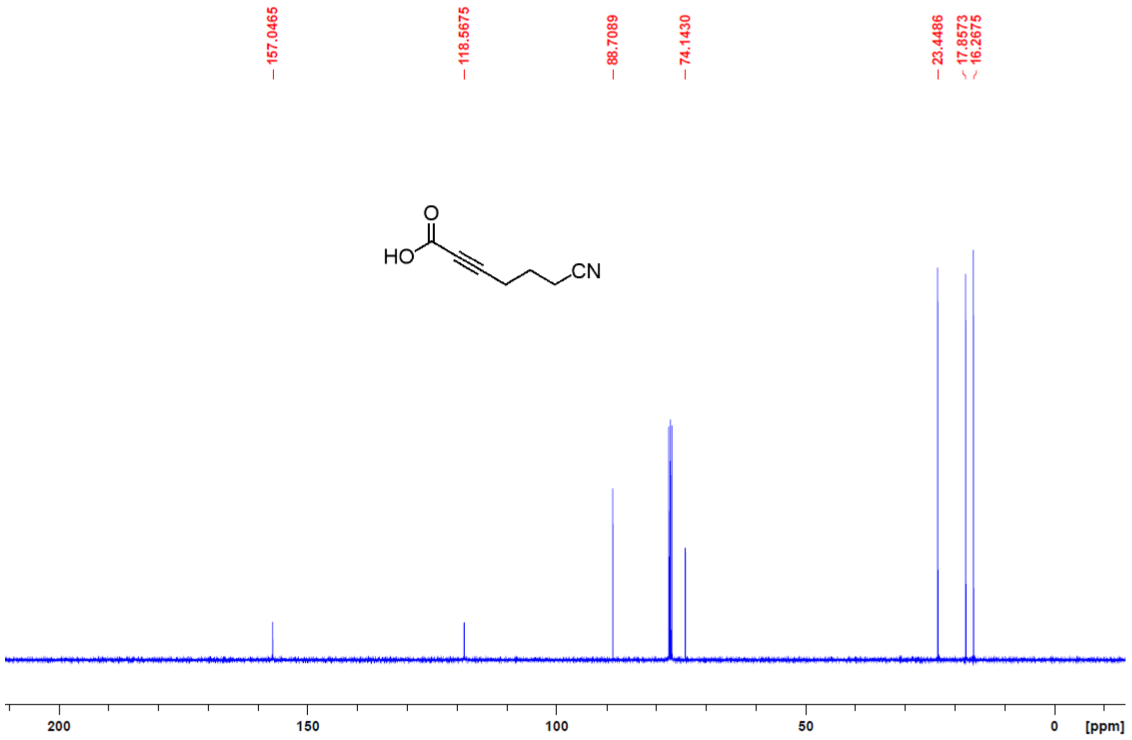

Supplementary Figure 162 ^{13}C NMR ($CDCl_3$, 100 MHz) spectrum of **S16**

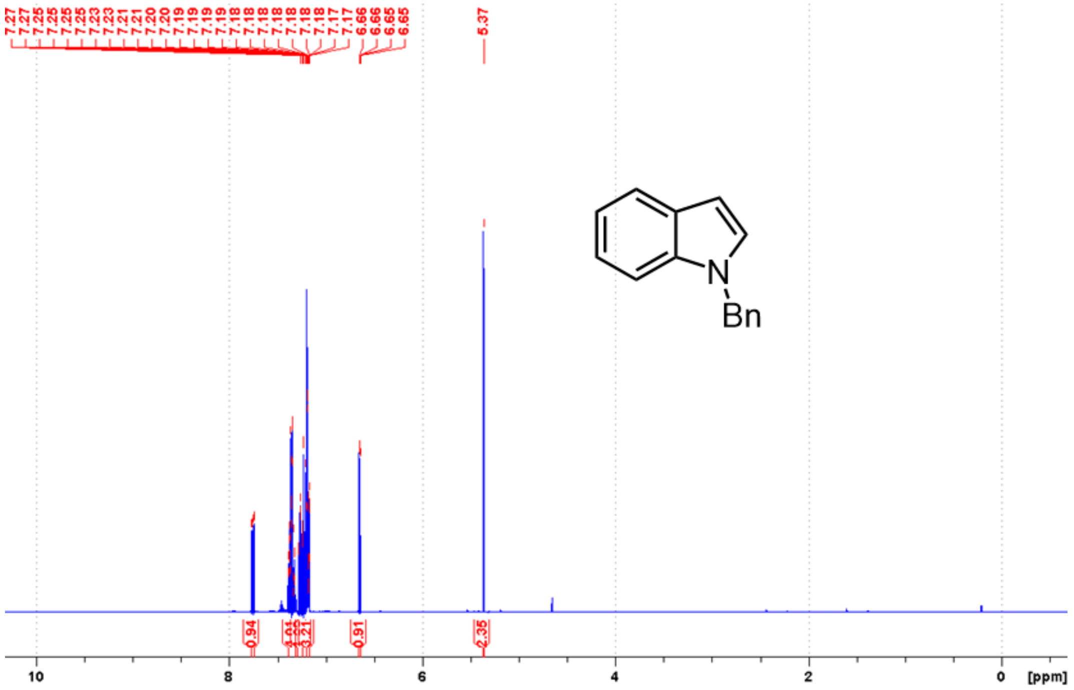

Supplementary Figure 163 ^{1}H NMR ($CDCl_3$, 400 MHz) spectrum of **S21**

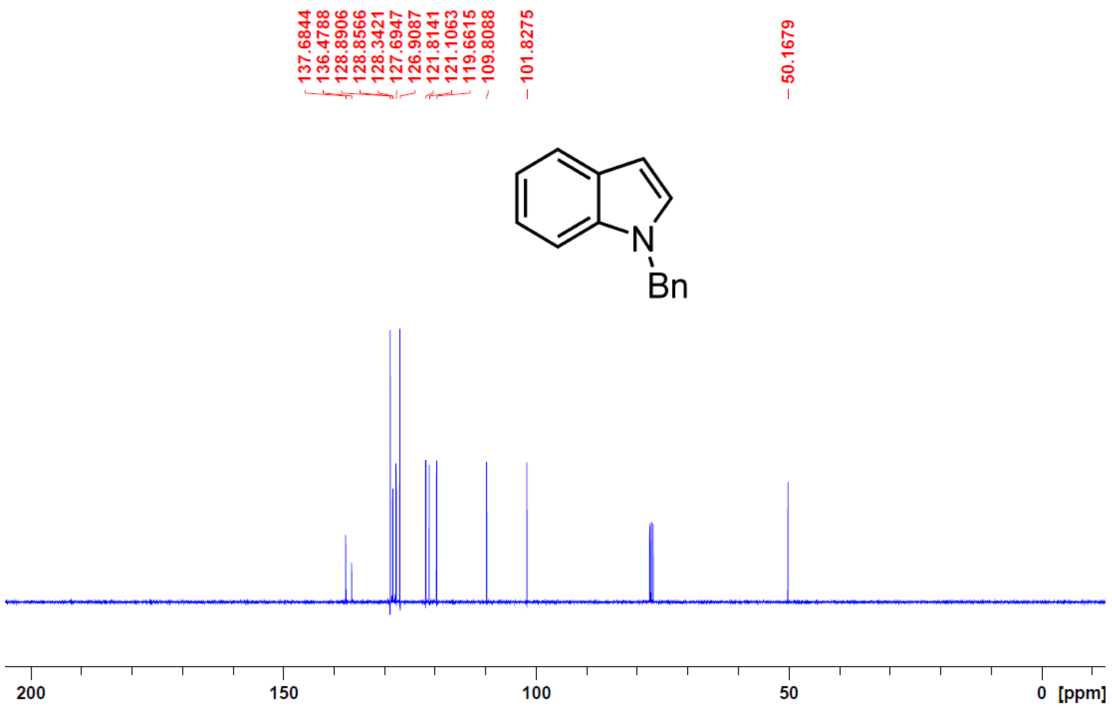

Supplementary Figure 164 ^{13}C NMR ($CDCl_3$, 100 MHz) spectrum of **S21**

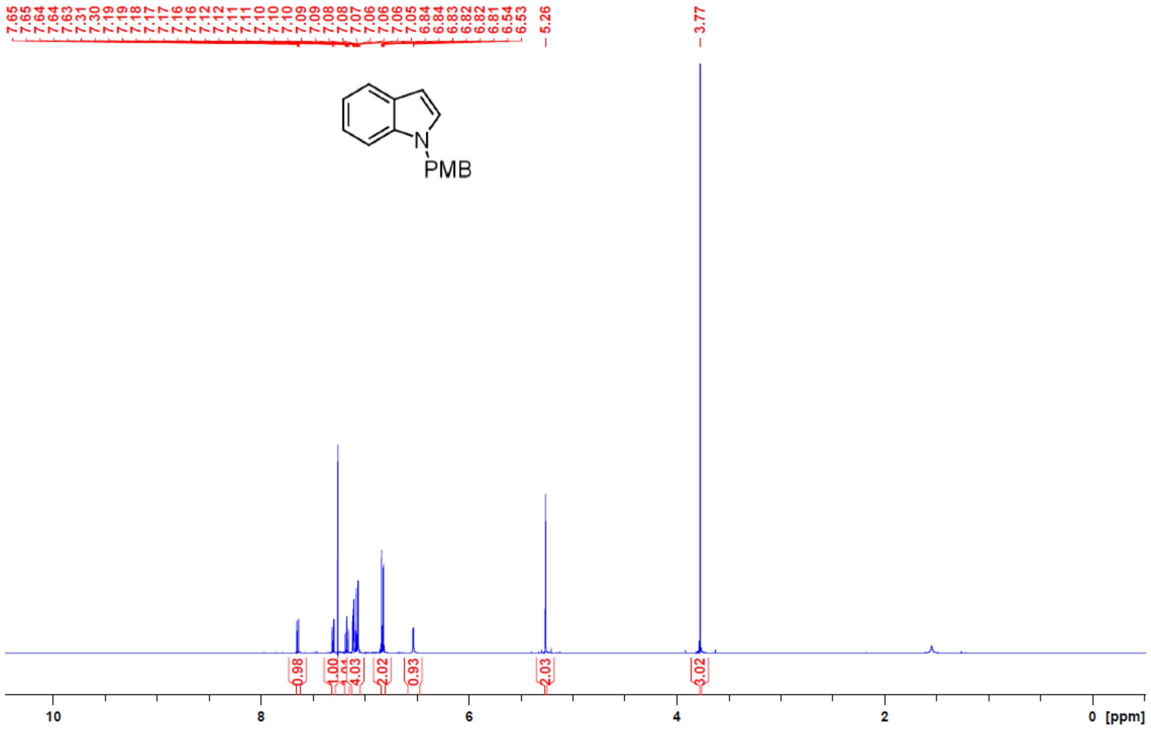

Supplementary Figure 165 ^{1}H NMR ($CDCl_3$, 500 MHz) spectrum of **S22**

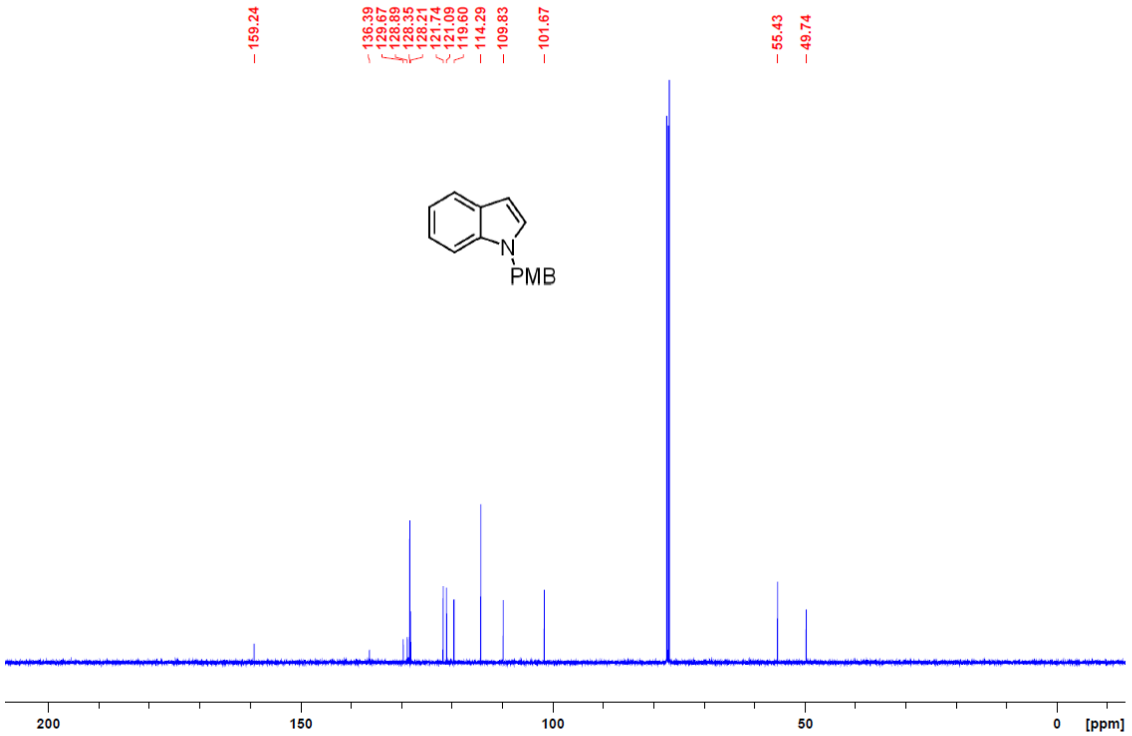

Supplementary Figure 166 ^{13}C NMR ($CDCl_3$, 126 MHz) spectrum of **S22**

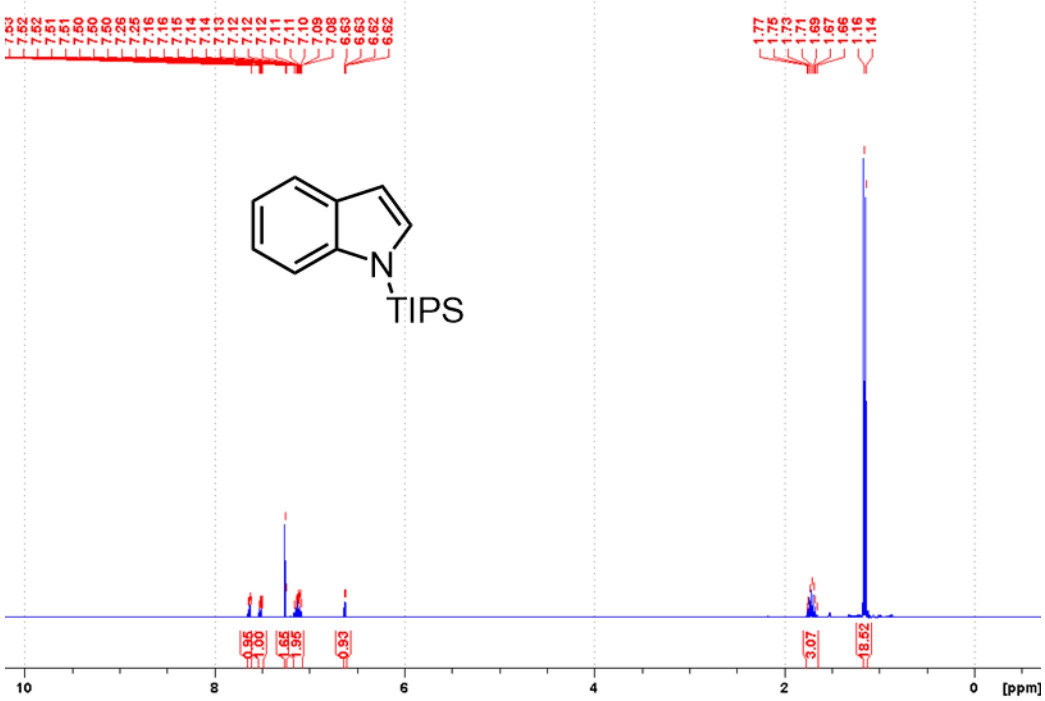

Supplementary Figure 167 ^{1}H NMR ($CDCl_3$, 400 MHz) spectrum of **S23**

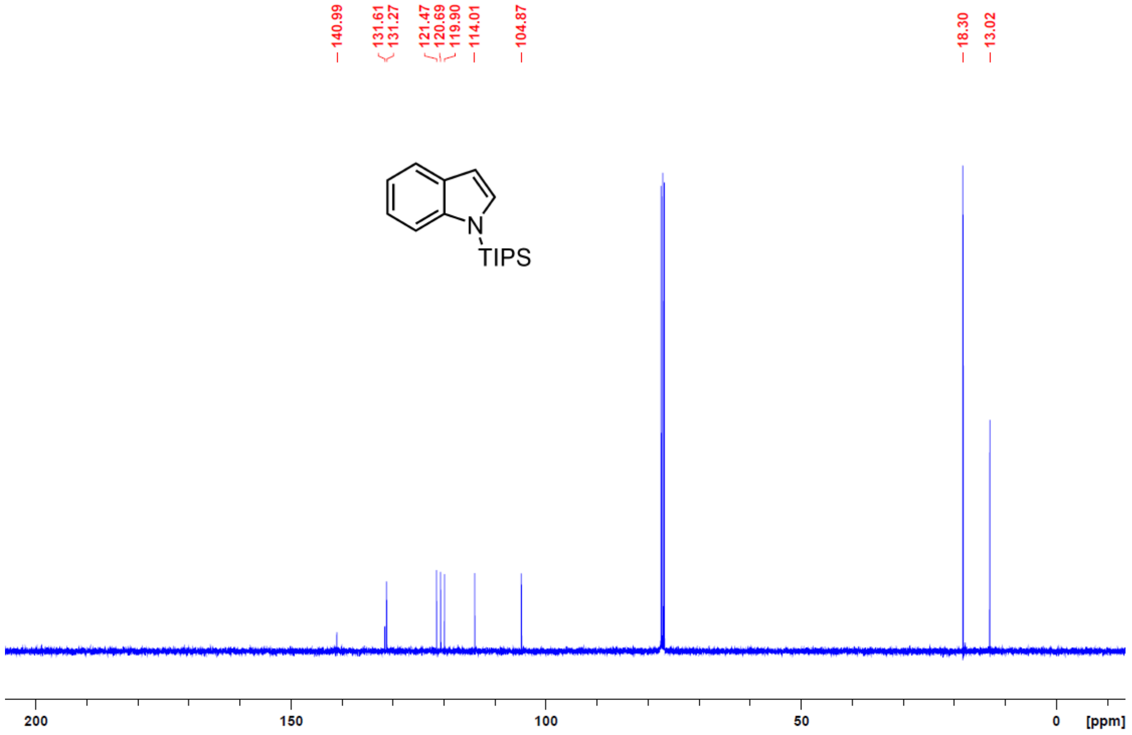

Supplementary Figure 168 ^{13}C NMR ($CDCl_3$, 100 MHz) spectrum of **S23**

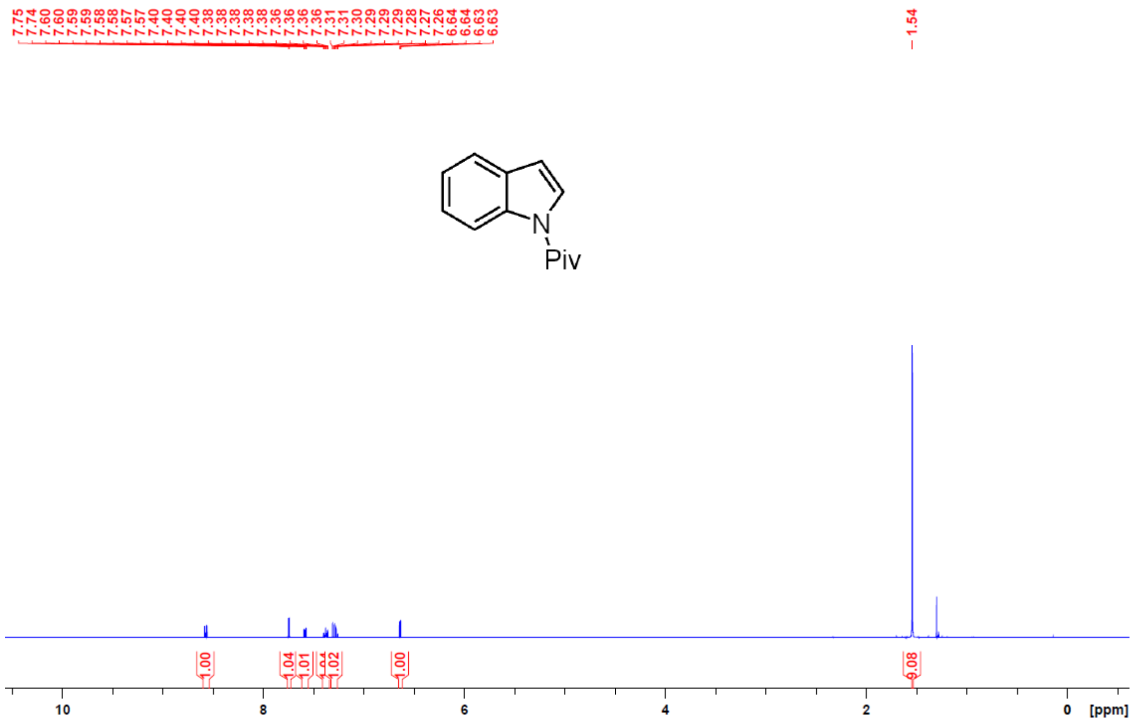

Supplementary Figure 169 ^{1}H NMR ($CDCl_3$, 400 MHz) spectrum of **S24**

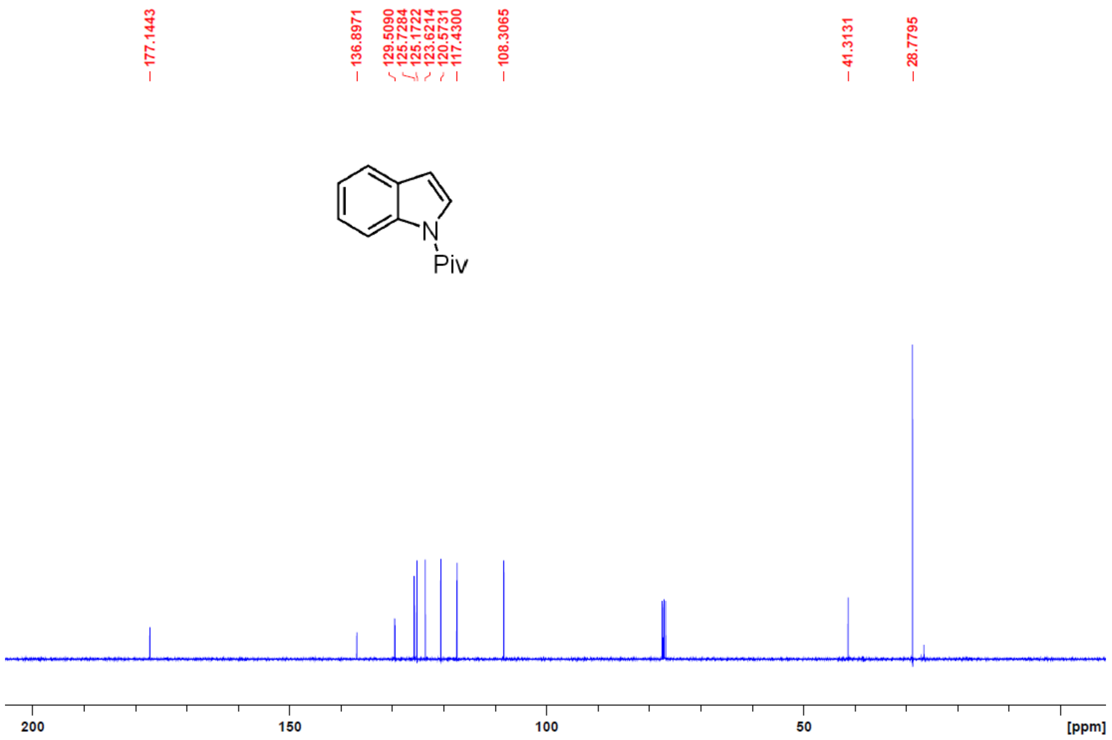

Supplementary Figure 170^{13}C NMR ($CDCl_3$, 100 MHz) spectrum of **S24**

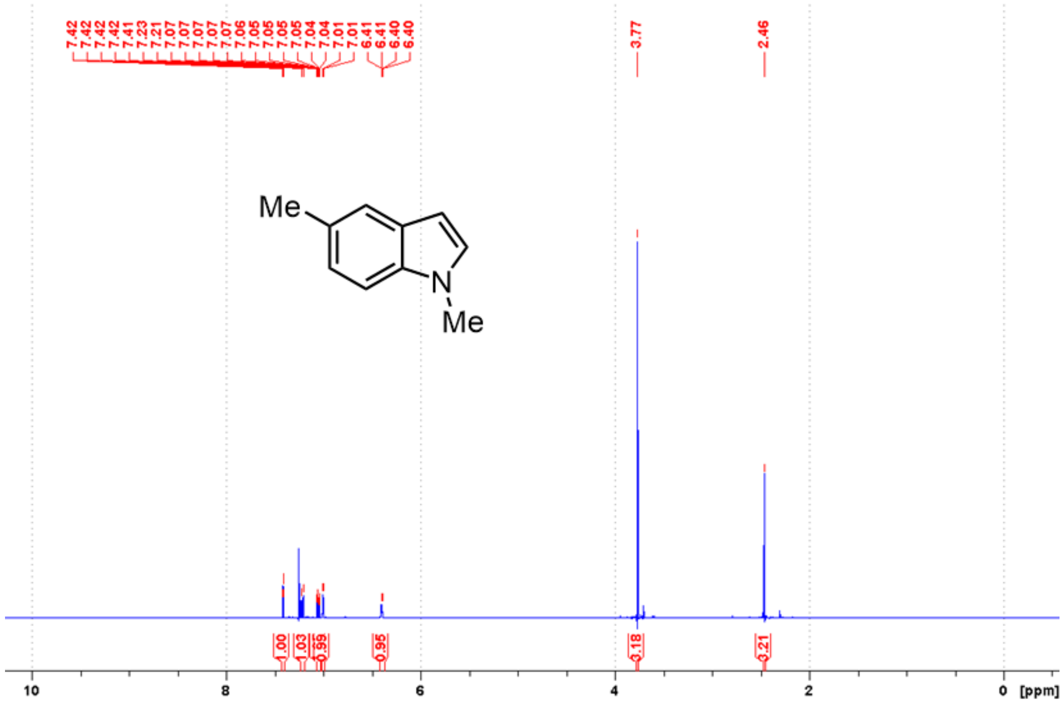

Supplementary Figure 171 ^{1}H NMR ($CDCl_3$, 400 MHz) spectrum of **S25**

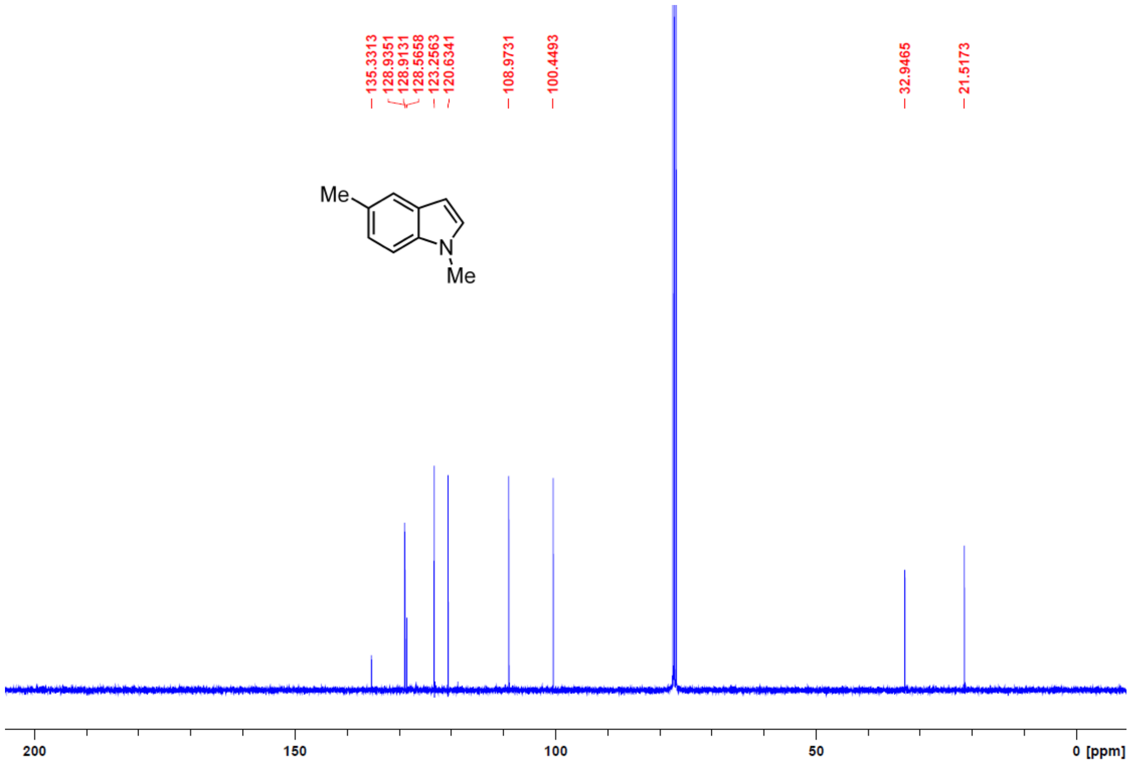

Supplementary Figure 172 ^{13}C NMR ($CDCl_3$, 100 MHz) spectrum of **S25**

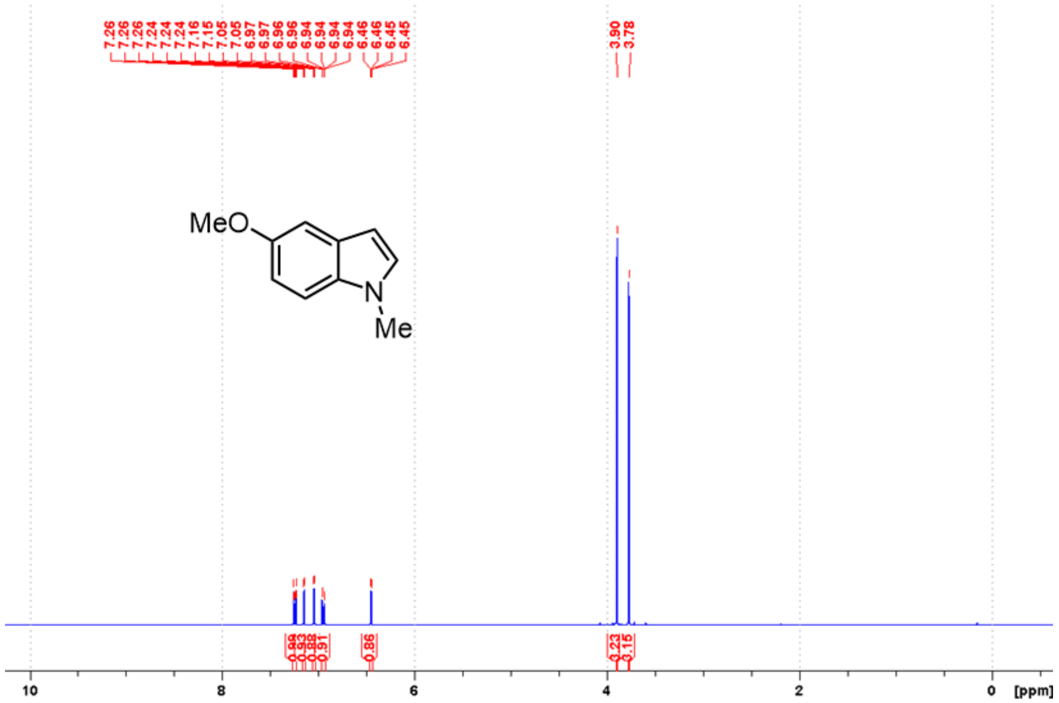

Supplementary Figure 173 ^{1}H NMR ($CDCl_3$, 400 MHz) spectrum of **S26**

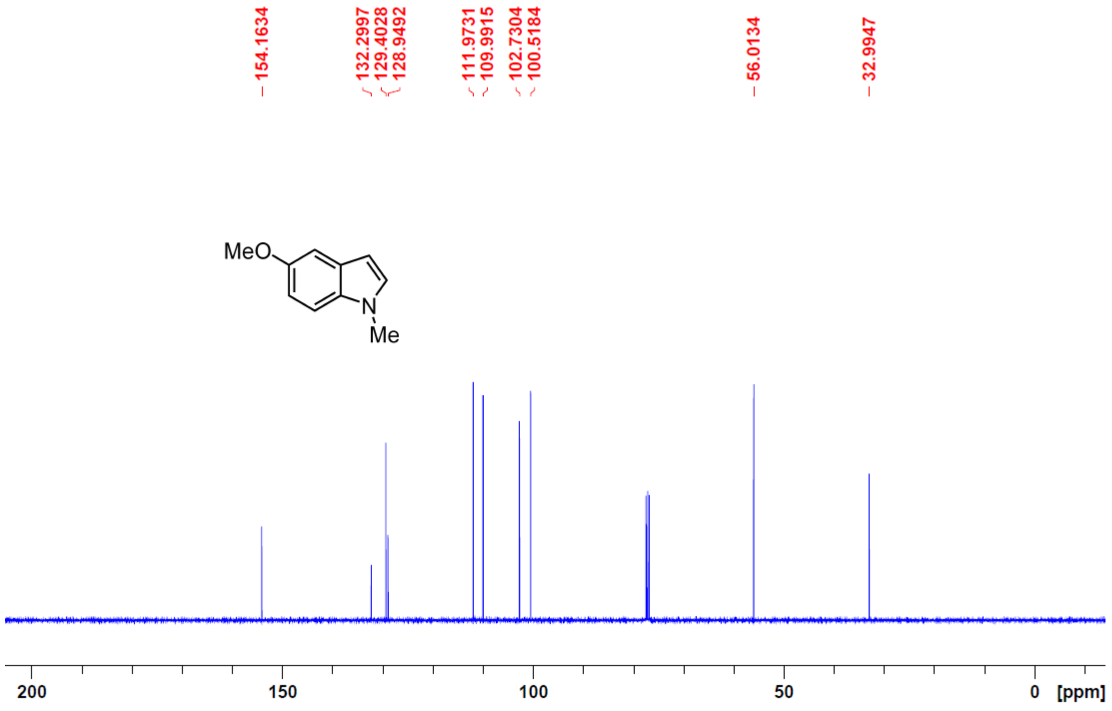

Supplementary Figure 174 ^{13}C NMR ($CDCl_3$, 100 MHz) spectrum of **S26**

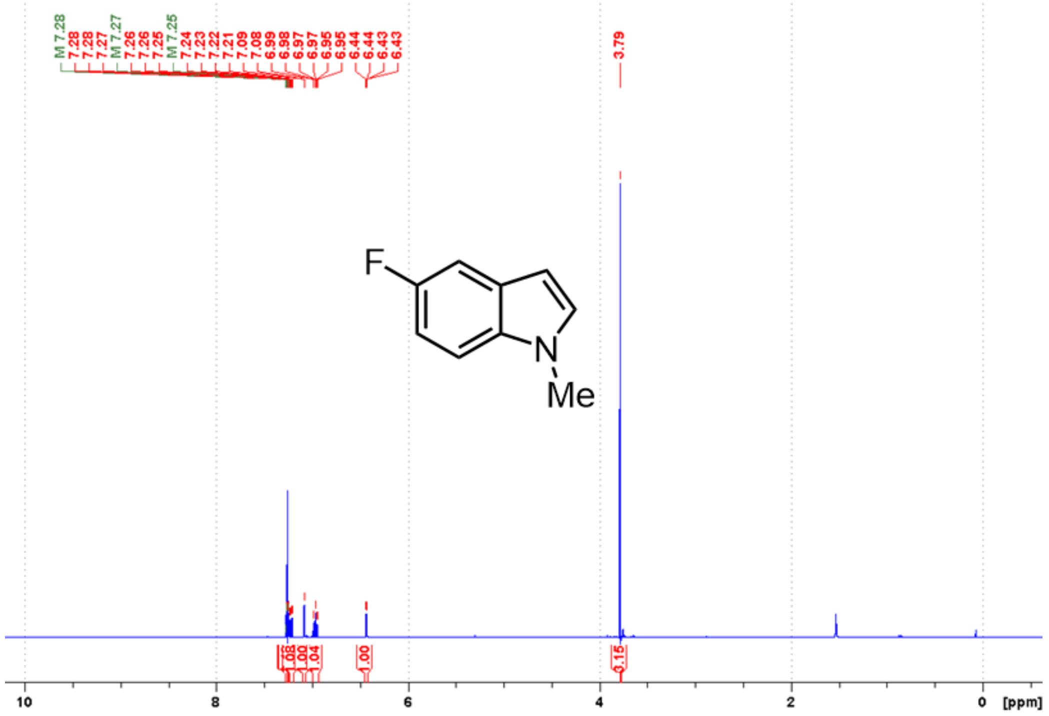

Supplementary Figure 175 ^{1}H NMR ($CDCl_3$, 500 MHz) spectrum of **S27**

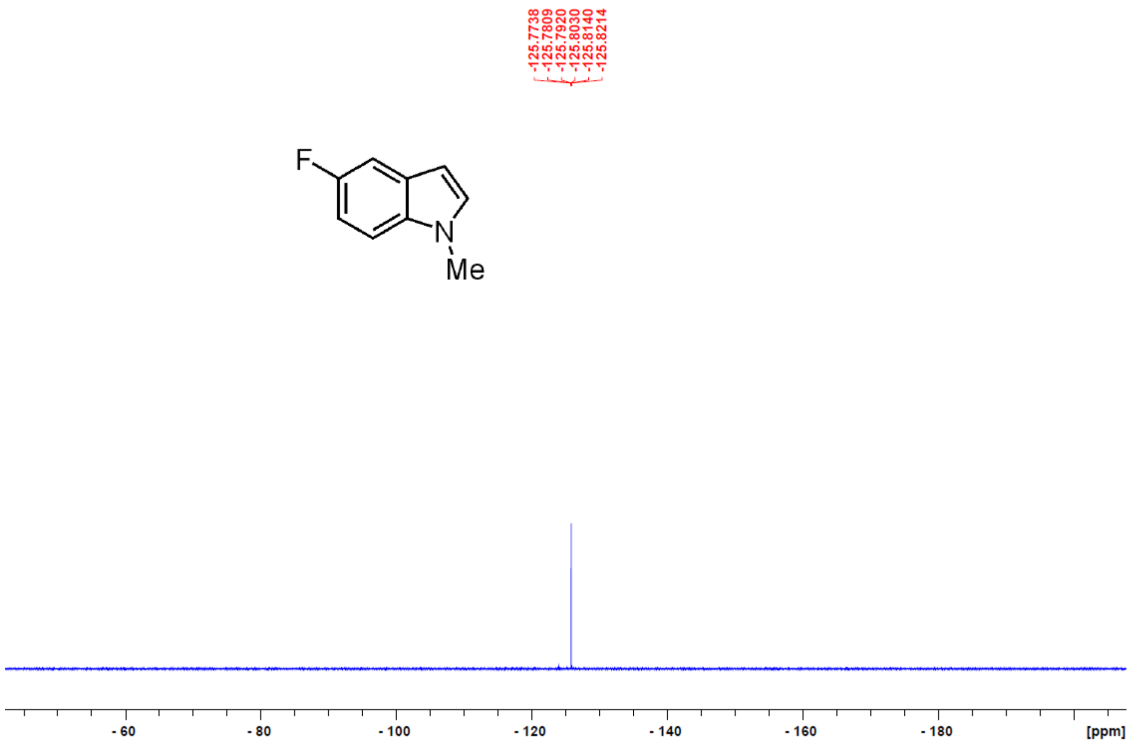

Supplementary Figure 176 ^{19}F NMR ($CDCl_3$, 377 MHz) spectrum of **S27**

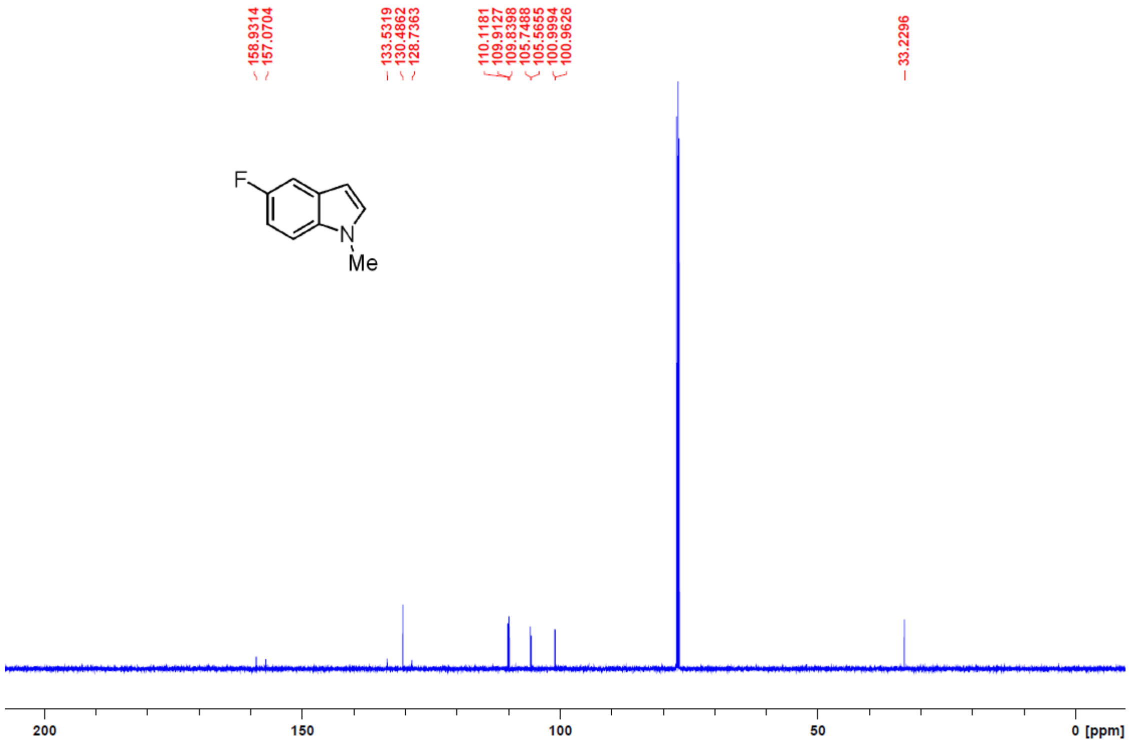

Supplementary Figure 177 ^{13}C NMR ($CDCl_3$, 126 MHz) spectrum of **S27**

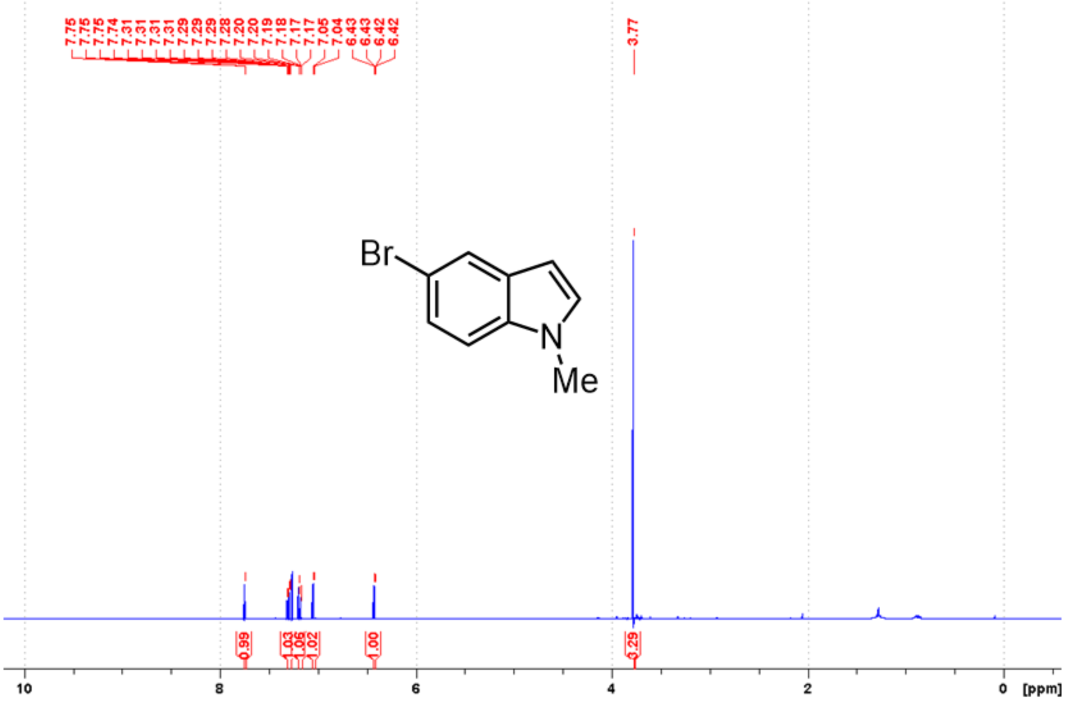

Supplementary Figure 178 ^{1}H NMR ($CDCl_3$, 500 MHz) spectrum of **S28**

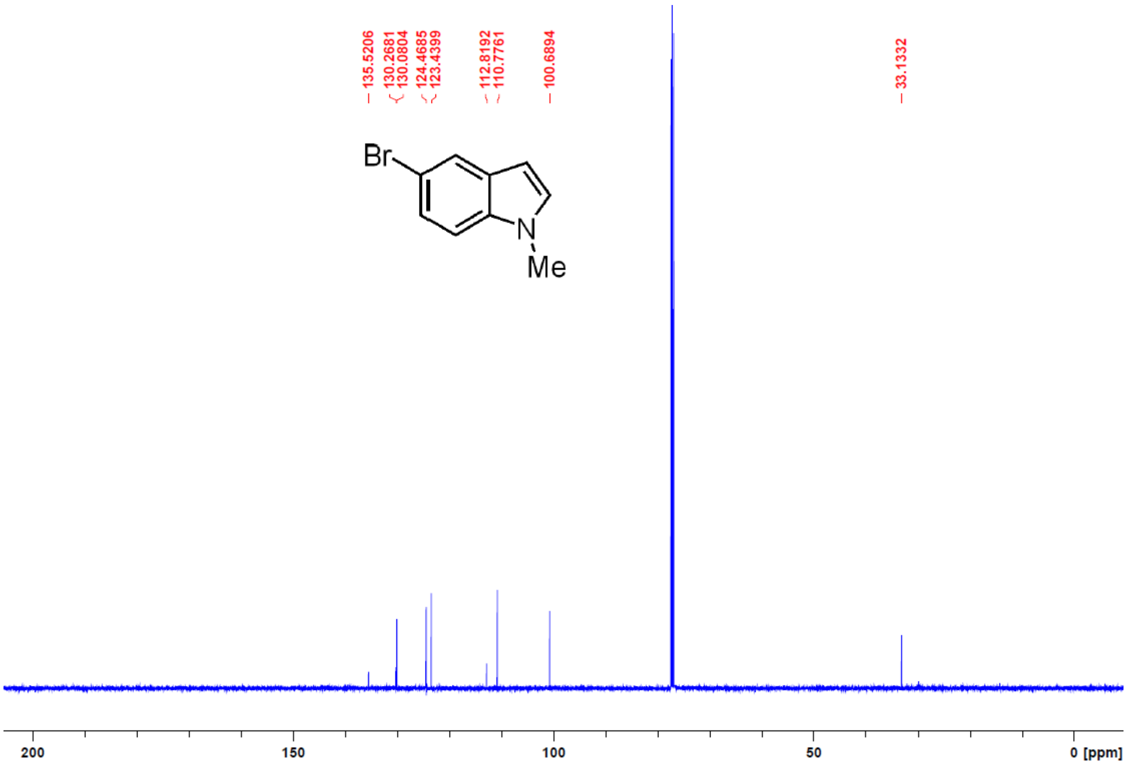

Supplementary Figure 179 ^{13}C NMR ($CDCl_3$, 126 MHz) spectrum of **S28**

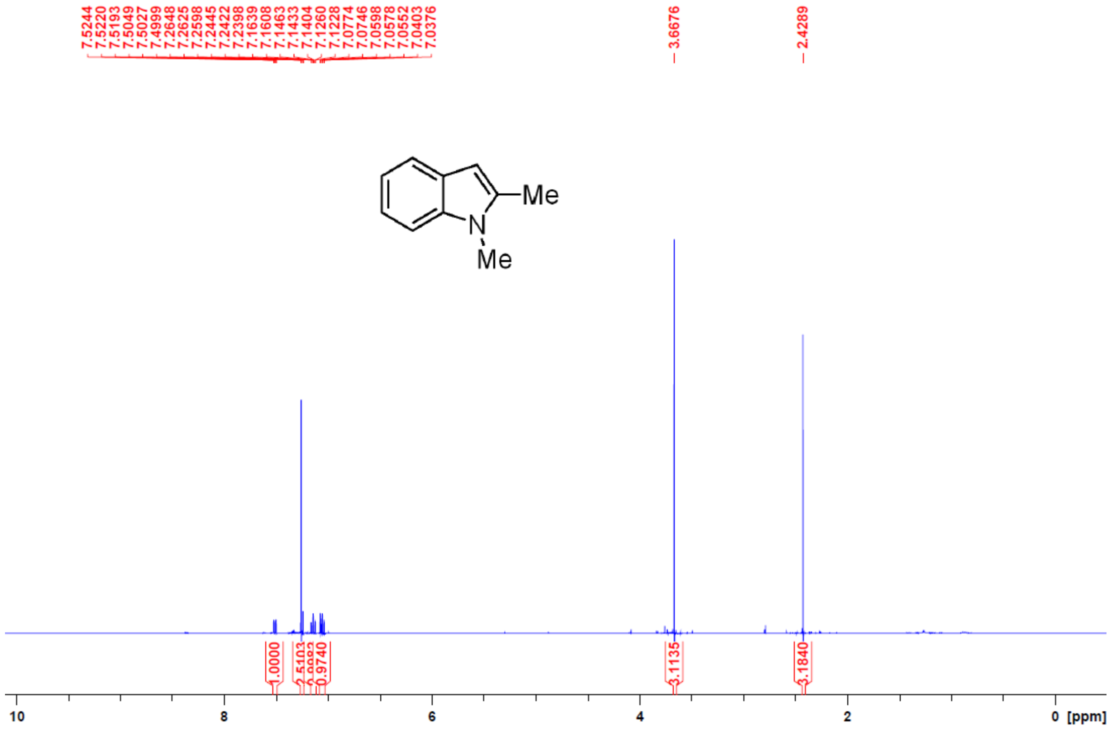

Supplementary Figure 180 ^{1}H NMR ($CDCl_3$, 400 MHz) spectrum of **S29**

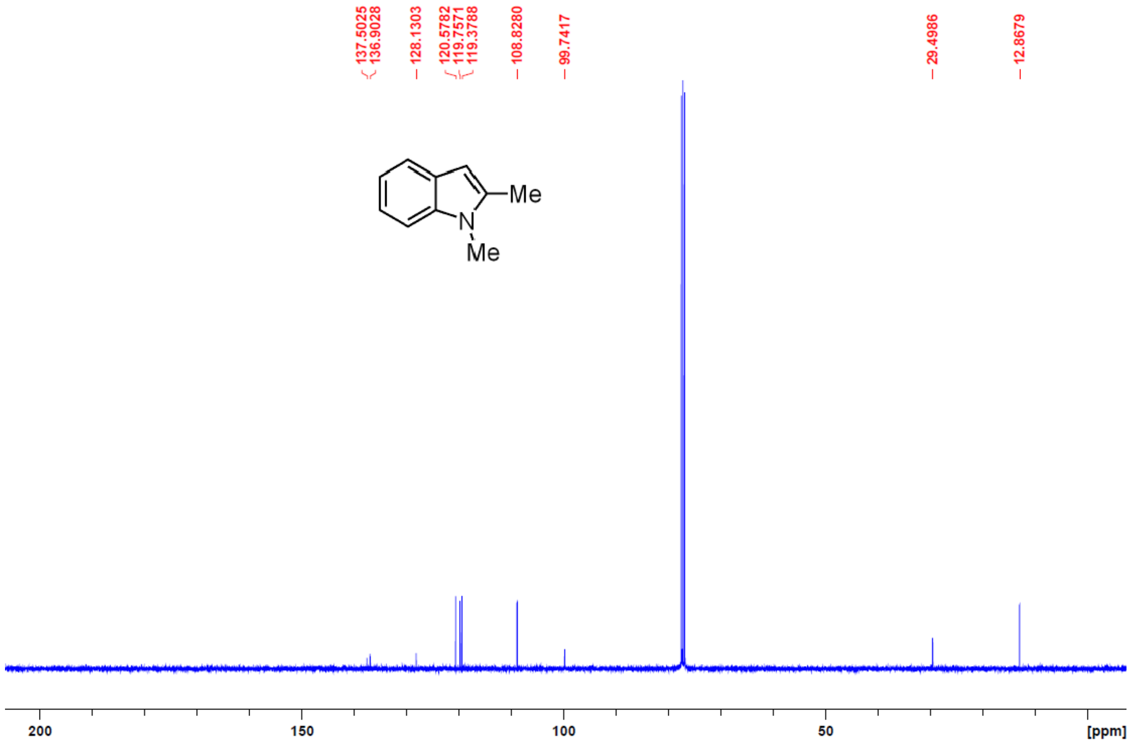

Supplementary Figure 181 ^{13}C NMR ($CDCl_3$, 100 MHz) spectrum of **S29**

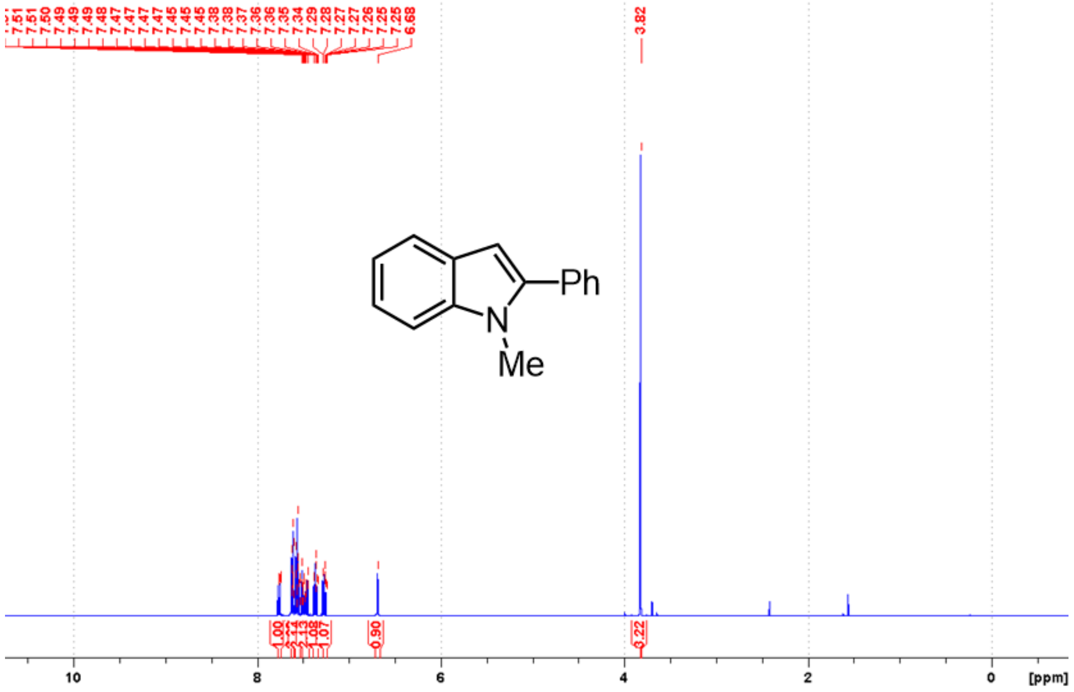

Supplementary Figure 182 ^{1}H NMR ($CDCl_3$, 400 MHz) spectrum of **S30**

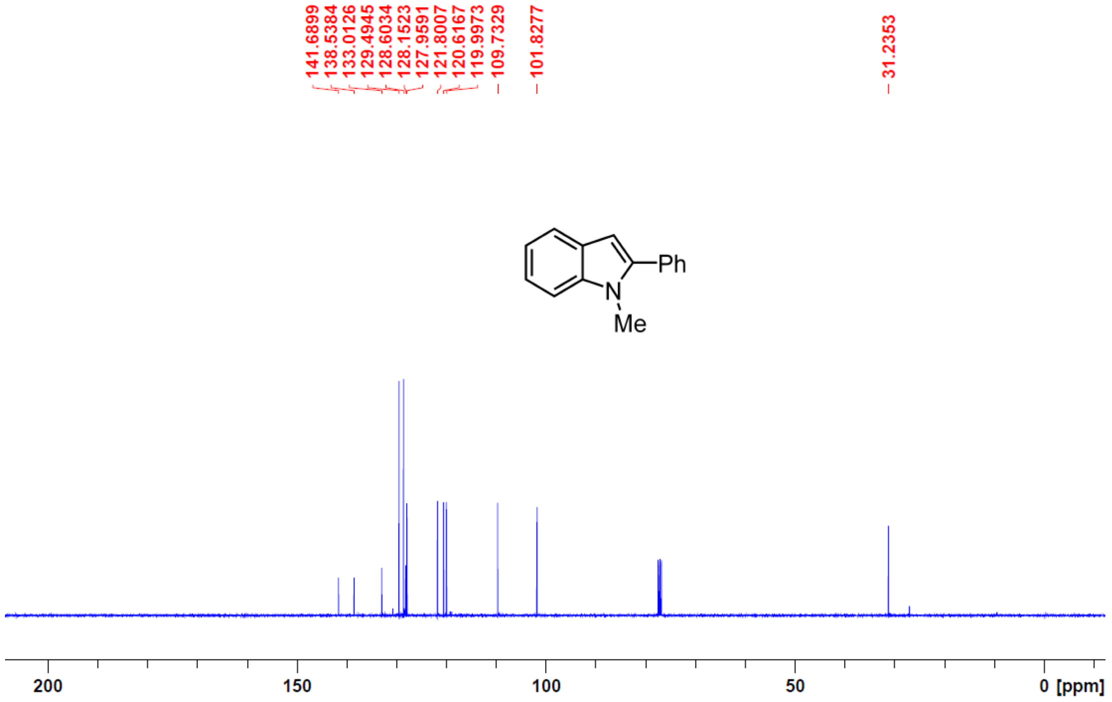

Supplementary Figure 183 ^{13}C NMR ($CDCl_3$, 100 MHz) spectrum of **S30**

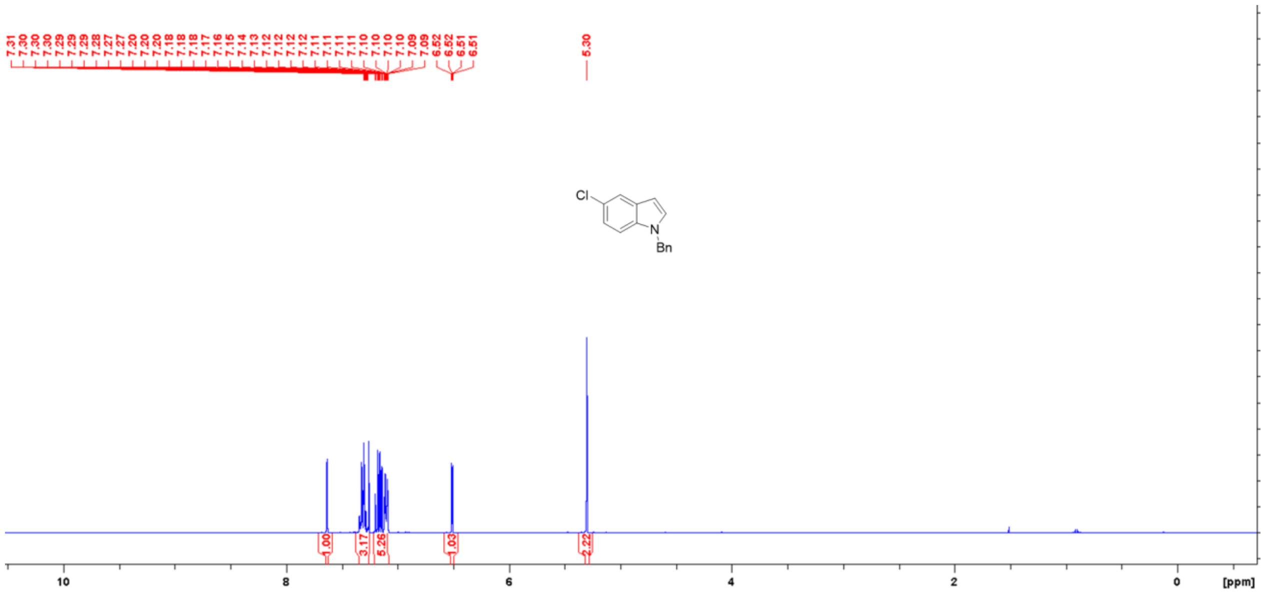

Supplementary Figure 184 ^{1}H NMR ($CDCl_3$, 400 MHz) spectrum of **S34**

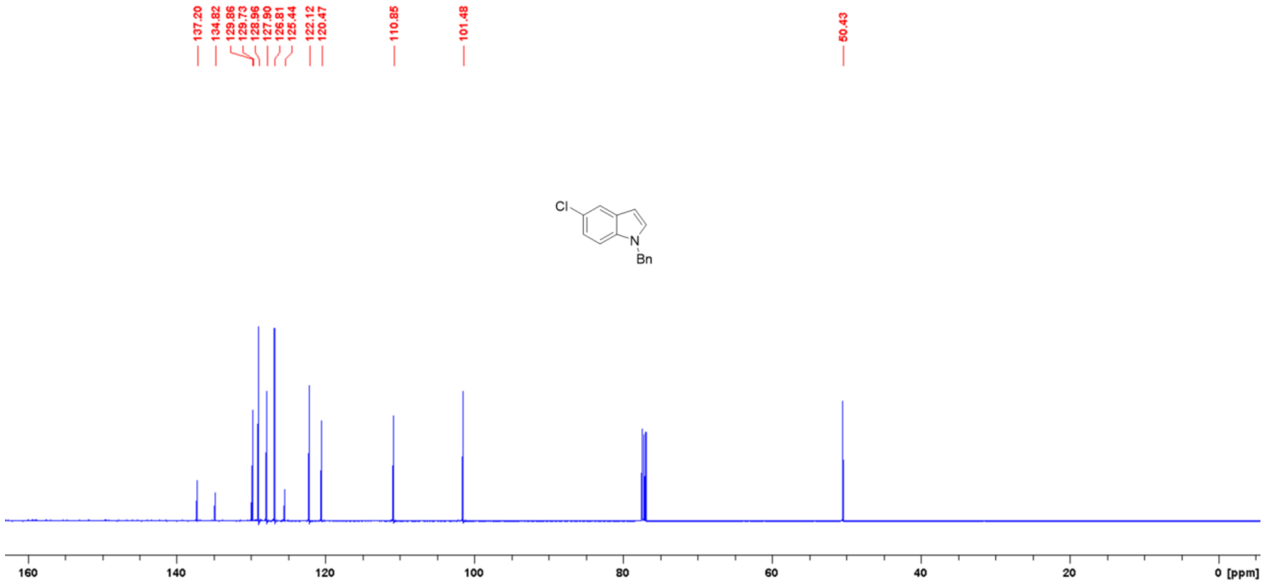

Supplementary Figure 185 ^{13}C NMR ($CDCl_3$, 126 MHz) spectrum of **S34**

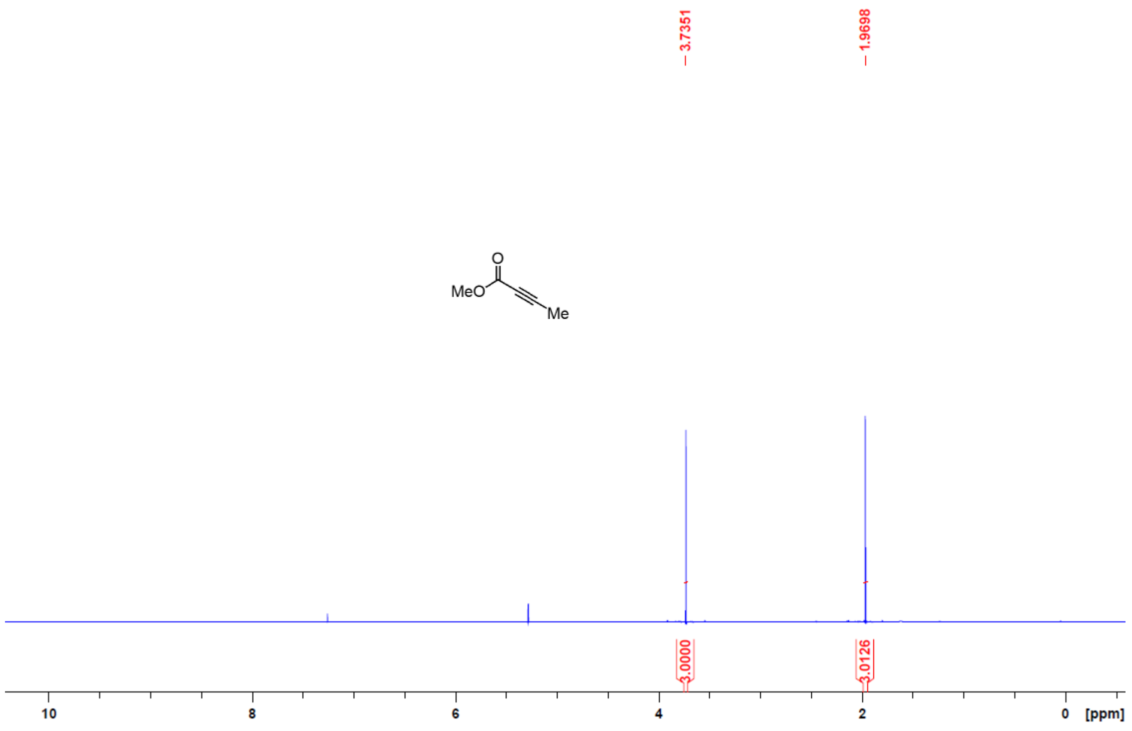

Supplementary Figure 186 ^{1}H NMR ($CDCl_3$, 400 MHz) spectrum of **S36**

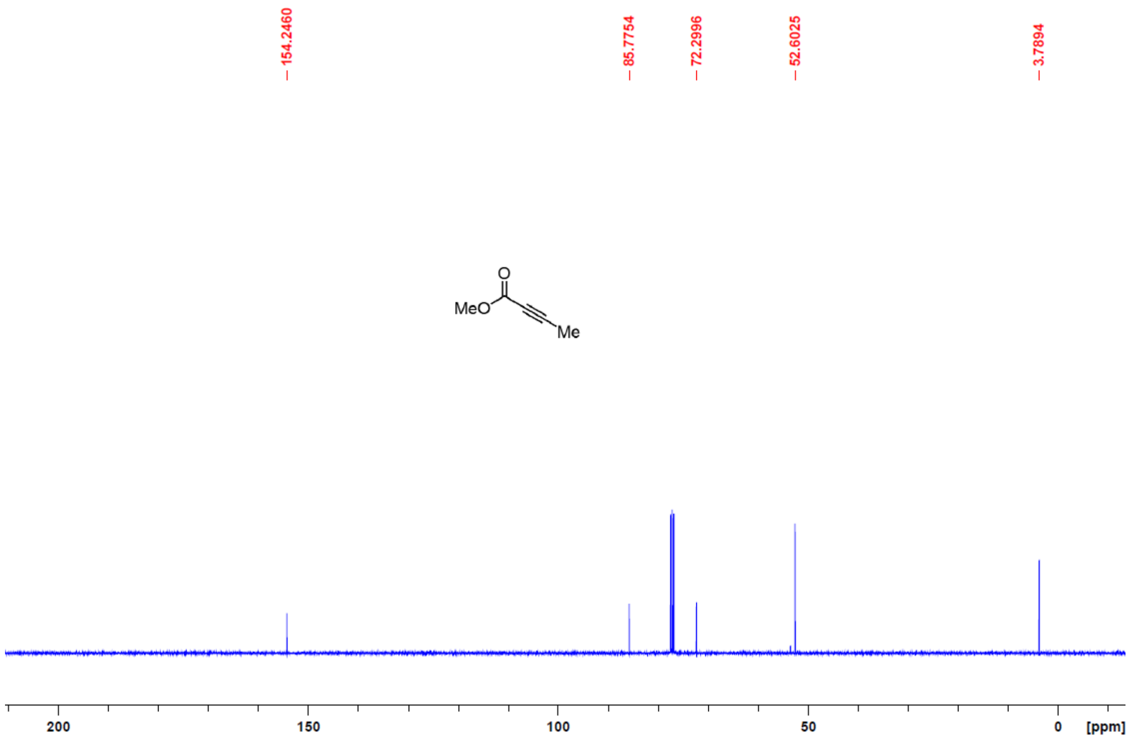

Supplementary Figure 187 ^{13}C NMR ($CDCl_3$, 100 MHz) spectrum of **S36**

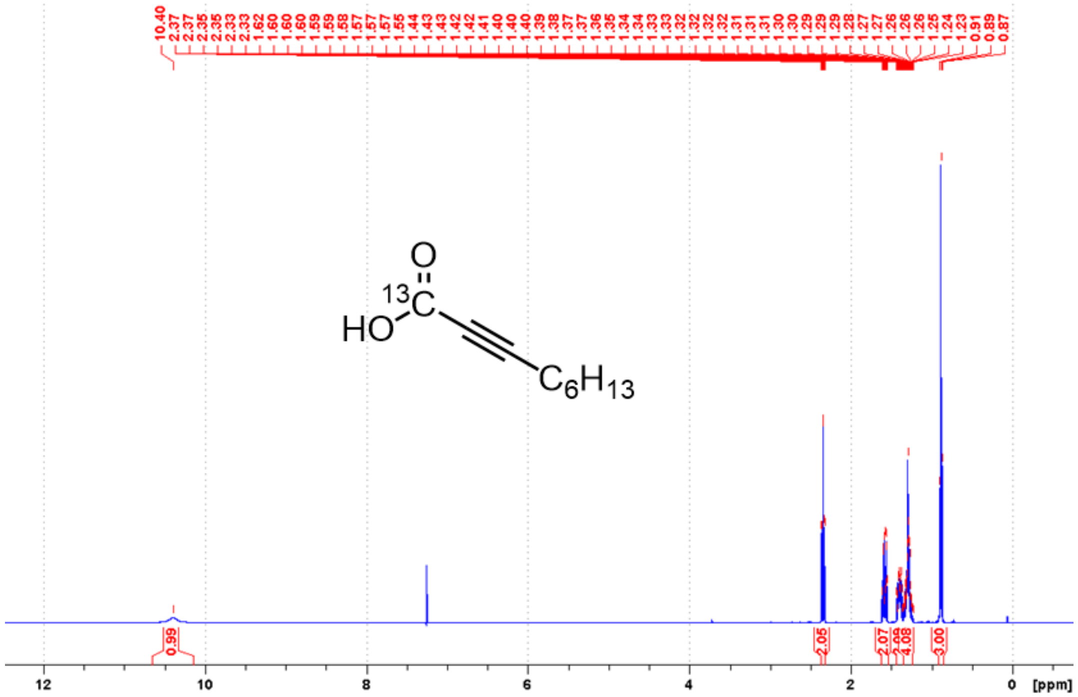

Supplementary Figure 188 ^{1}H NMR ($CDCl_3$, 400 MHz) spectrum of **S37**

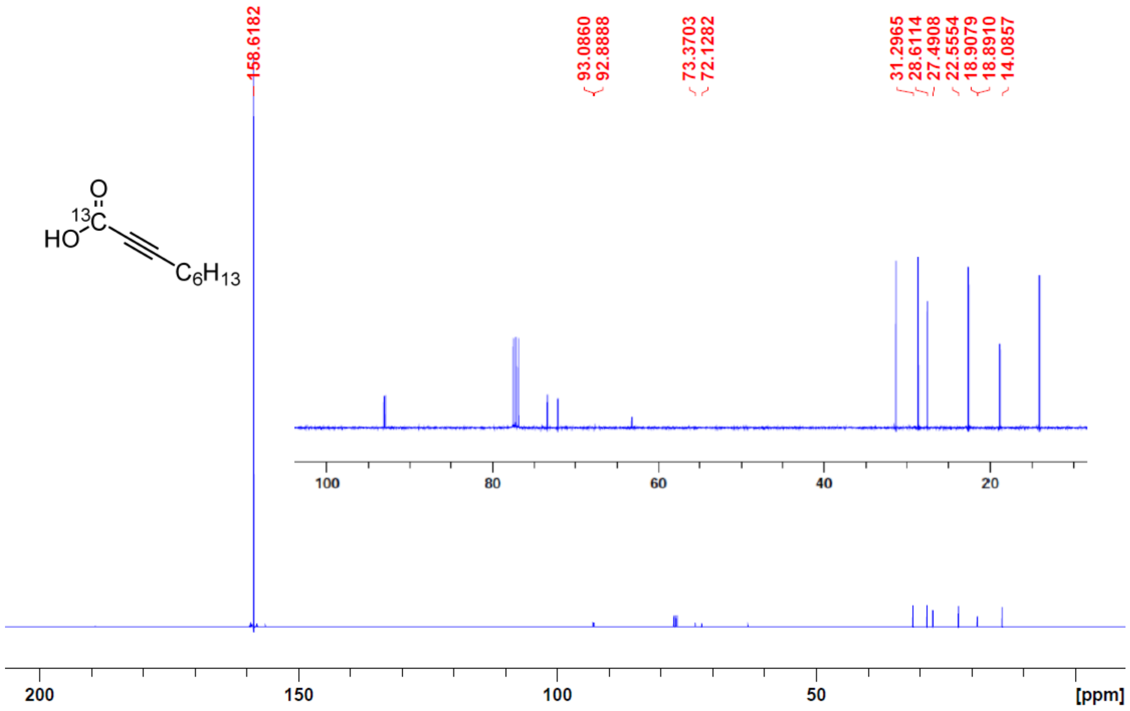

Supplementary Figure 189 ^{13}C NMR ($CDCl_3$, 100 MHz) spectrum of **S37**

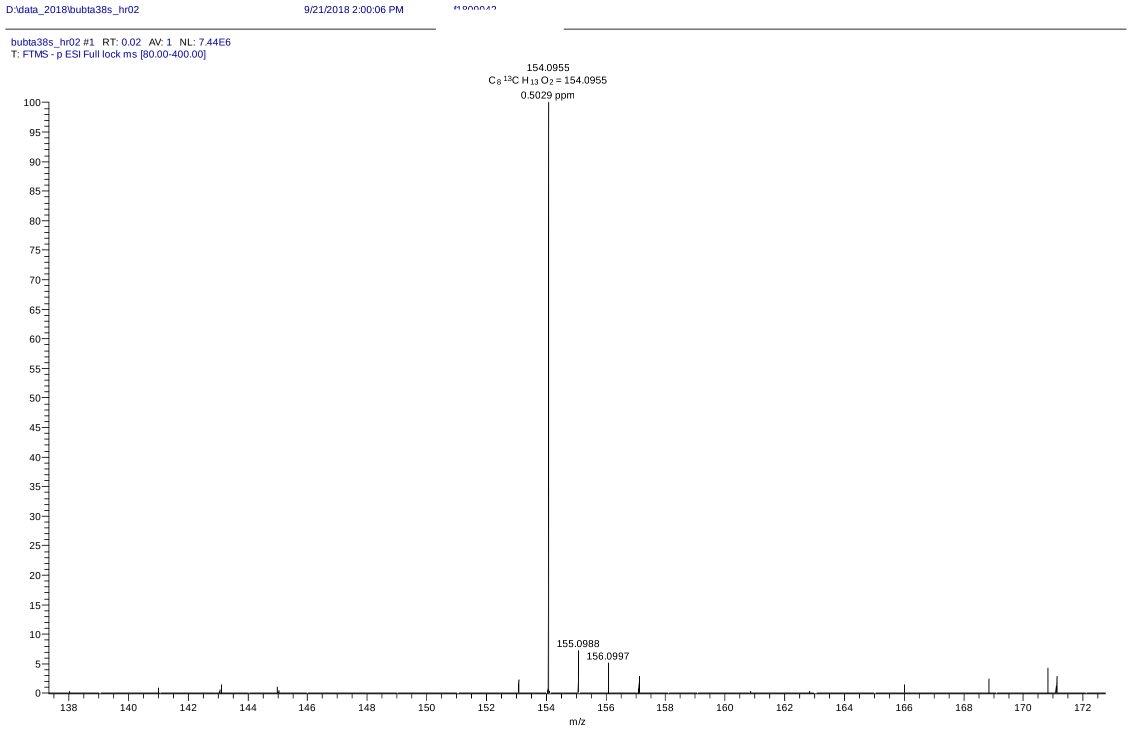

Supplementary Figure 190 ESI-MS spectra of **S37**

Supplementary References

1 Fang, W. & Breit, B. Tandem Regioselective Hydroformylation-Hydrogenation of Internal Alkynes Using a Supramolecular Catalyst. *Angew. Chem. Int. Ed.* **57**, 14817-14821 (2018).

2 Smejkal, T. & Breit, B. A supramolecular catalyst for regioselective hydroformylation of unsaturated carboxylic acids. *Angew. Chem. Int. Ed.* **47**, 311-315 (2008).

3 Conde, N., SanMartin, R., Herrero, M. T. & Domínguez, E. Palladium NNC Pincer Complex as an Efficient Catalyst for the Cycloisomerization of Alkynoic Acids. *Adv. Synth. Catal.* **358**, 3283-3292 (2016).

4 Szamosvári, D. & Böttcher, T. An Unsaturated Quinolone N-Oxide of Pseudomonas aeruginosa Modulates Growth and Virulence of Staphylococcus aureus. *Angew. Chem. Int. Ed.* **56**, 7271-7275 (2017).

5 Alhamadsheh, M. M., Palaniappan, N., DasChouduri, S. & Reynolds, K. A. Modular Polyketide Synthases and cis Double Bond Formation: Establishment of Activated cis-3-Cyclohexylpropenoic Acid as the Diketide Intermediate in Phoslactomycin Biosynthesis. *J. Am. Chem. Soc.* **129**, 1910-1911 (2007).

6 Slack, E. D., Gabriel, C. M. & Lipshutz, B. H. A palladium nanoparticle-nanomicelle combination for the stereoselective semihydrogenation of alkynes in water at room temperature. *Angew. Chem. Int. Ed.* **53**, 14051-14054 (2014).

7 Zheng, L. *et al.* Visible-Light-Mediated Anti-Regioselective Nitrone 1,3-Dipolar Cycloaddition Reaction and Synthesis of Bisindolylmethanes. *Org. Lett.* **19**, 5086-5089 (2017).

8 Das, A., Watanabe, K., Morimoto, H. & Ohshima, T. Boronic Acid Accelerated Three-Component Reaction for the Synthesis of alpha-Sulfanyl-Substituted Indole-3-acetic Acids. *Org. Lett.* **19**, 5794-5797 (2017).

9 Kerr, W. J. *et al.* Site-Selective Deuteration of N-Heterocycles via Iridium-Catalyzed Hydrogen Isotope Exchange. *ACS Catal.* **7**, 7182-7186 (2017).

10 Saxena, P. R. *et al.* Effects of avitriptan, a new 5-HT1B/1D receptor agonist, in experimental models predictive of antimigraine activity and coronary side-effect potential. *Naunyn-Schmiedeberg's Arch. Pharmacol.* **355**, 295-302 (1997).

11 Brodfuehrer, P. R. *et al.* An Efficient Fischer Indole Synthesis of Avitriptan, a Potent 5-HT1DReceptor Agonist. *J. Org. Chem.* **62**, 9192-9202 (1997).

12 Zheng, B., Li, M., Gao, G., He, Y. & Walsh, P. J. Palladium-Catalyzed alpha-Arylation of Methyl Sulfonamides with Aryl Chlorides. *Adv. Synth. Catal.* **358**, 2156-2162 (2016).

13 Hudrlik, P. F., Holmes, P. E. & Hudrlik, A. M. Protiodesilylation reactions of β-and γ-hydroxysilanes: Deuterium labeling and silicon-directed epoxide openings. *Tetrahedron lett.* **29**, 6395-6398 (1988)

14 Frisch, M. J.; Trucks, G. W.; Schlegel, H. B.; Scuseria, G. E.; Robb, M. A.; Cheeseman, J. R.; Scalmani, G.; Barone, V.; Mennucci, B.; Petersson, G. A.; Nakatsuji, H.; Caricato, M.; Li, X.; Hratchian, H. P.; Izmaylov, A. F.; Bloino, J.; Zheng, G.; Sonnenberg, J. L.; Hada, M.; Ehara, M.; Toyota, K.; Fukuda, R.; Hasegawa, J.; Ishida, M.; Nakajima, T.; Honda, Y.; Kitao, O.; Nakai, H.; Vreven, T.; Montgomery, J. A., Jr.; Peralta, J. E.; Ogliaro, F.; Bearpark, M.; Heyd, J. J.; Brothers, E.; Kudin, K. N.; Staroverov, V. N.; Kobayashi, R.; Normand, J.; Raghavachari, K.; Rendell, A.; Burant, J. C.; Iyengar, S. S.; Tomasi, J.; Cossi, M.; Rega, N.; Millam, N. J.; Klene, M.; Knox, J. E.; Cross, J. B.; Bakken, V.; Adamo, C.; Jaramillo, J.; Gomperts, R.; Stratmann, R. E.; Yazyev, O.; Austin, A. J.; Cammi, R.; Pomelli, C.; Ochterski, J. W.; Martin, R. L.; Morokuma, K.; Zakrzewski, V. G.; Voth, G. A.; Salvador, P.; Dannenberg, J. J.; Dapprich, S.; Daniels, A. D.; Farkas, O.; Foresman, J. B.; Ortiz, J. V.; Cioslowski, J.; Fox, D. J. Gaussian 09, rev. B.01; Gaussian, Inc.: Wallingford, CT, (2010).

15 Becke, A. D. Density-functional exchange-energy approximation with correct asymptotic behavior. *Phys. Rev. A* **38**, 3098−3100 (1988).

16 Perdew, J. P. Density-functional approximation for the correlation energy of the inhomogeneous electron gas. *Phys. Rev. B* **33**, 8822−8824 (1986).

17 Weigend, F., Ahlrichs, R. Balanced basis sets of split valence, triple zeta valence and quadruple zeta valence quality for H to Rn: Design and assessment of accuracy. *Phys. Chem. Chem. Phys.* **7**, 3297−3305 (2005).

.
